# Comprehensive Effects of Melatonin Supplementation on Cardiometabolic Risk Factors: A Systematic Review and Dose–Response Meta-Analysis

**DOI:** 10.3390/nu18010134

**Published:** 2025-12-31

**Authors:** Shooka Mohammadi, Damoon Ashtary-Larky, Mahsa Erfanian-Salim, Navid Alaghemand, Mojtaba Yousefi, Pouyan Sanjari Pirayvatlou, Mohammadreza Mirkarimi, Sara Ayazian Mavi, Ilnaz Alavi, Yeganeh Ettehad, Milad Mehrbod, Omid Asbaghi, Katsuhiko Suzuki, Russel J. Reiter

**Affiliations:** 1 Department of Social and Preventive Medicine, Faculty of Medicine, University of Malaya, Kuala Lumpur 50603, Malaysia; shooka.mohammadi@gmail.com; 2 Nutrition and Metabolic Diseases Research Center, Ahvaz Jundishapur University of Medical Sciences, Ahvaz 6135715794, Iran; n.alaghemand@gmail.com; 3 Department of Pediatric Cardiology, Children’s Medical Center, Tehran University of Medical Sciences, Tehran 1416753955, Iran; merfaniansalim@gmail.com; 4 Department of Cardiology, Faculty of Medicine, Ahvaz Jundishapur University of Medical Sciences, Ahvaz 6135715794, Iran; mojtaba657@gmail.com; 5 Department of Internal Medicine, Faculty of Medicine, Alborz University of Medical Sciences, Karaj 3149969415, Iran; dr.pouyan.sanjari.p@gmail.com (P.S.P.); yeganehettehad@gmail.com (Y.E.); 6 Department of Pediatrics, Faculty of Medicine, Ahvaz Jundishapur University of Medical Sciences, Ahvaz 6135715794, Iran; mirkarimi-smr@ajums.ac.ir; 7 Department of Community Medicine, Faculty of Medicine, Ahvaz Jundishapur University of Medical Sciences, Ahvaz 6135715794, Iran; sayazianm1979@gmail.com; 8 Division of Cardiovascular Medicine, School of Medicine, University of Louisville, Louisville, KY 40222, USA; i0alav01@louisville.edu; 9 Department of Internal Medicine, Mercy San Juan Medical Center, Carmichael, CA 95608, USA; milad.mehrbod@commonspirit.org; 10 Cancer Research Center, Shahid Beheshti University of Medical Sciences, Tehran 1985717443, Iran; omid.asbaghi@gmail.com; 11 Faculty of Sport Sciences, Waseda University, Tokorozawa 359-1192, Japan; 12 Department of Cell Systems and Anatomy, Long School of Medicine, UT Health San Antonio, San Antonio, TX 78229, USA; reiter@uthscsa.edu

**Keywords:** melatonin, oxidative stress, inflammation, cardiometabolic risks, lipid profile, blood pressure, glycemic control

## Abstract

**Background/Objectives**: There is no definitive consensus regarding the effects of melatonin on cardiometabolic risk factors (CMRFs). This systematic review and dose–response meta-analysis of randomized controlled trials (RCTs) evaluated the impacts of melatonin supplementation on CMRFs, including anthropometric, lipid, glycemic, inflammatory, oxidative, and liver function parameters. **Methods**: A systematic search across multiple databases retrieved 63 eligible RCTs published up to October 2025. **Results**: This random-effects meta-analysis indicated that melatonin supplementation significantly reduced hip circumference (weighted mean difference (WMD): −1.18 cm, 95% confidence interval (CI): −2.28, −0.08), systolic blood pressure (WMD: −2.34 mmHg, 95% CI: −4.13, −0.55), fasting blood glucose (WMD: −11.63 mg/dL, 95% CI: −19.16, −4.10), low-density lipoprotein cholesterol (WMD: −6.28 mg/dL, 95% CI: −10.53, −2.03), total cholesterol (WMD: −6.97 mg/dL, 95% CI: −12.20, −1.74), C-reactive protein (WMD: −0.59 mg/L, 95% CI: −0.94, −0.23), malondialdehyde (WMD: −1.54 μmol/L, 95% CI: −2.07, −1.01), tumor necrosis factor-alpha (WMD: −1.61 pg/mL, 95% CI: −2.31, −0.90), interleukin-6 (WMD: −6.43 pg/mL, 95% CI: −10.72, −2.15), and alanine aminotransferase (WMD: −2.61 IU/L, 95% CI: −4.87, −0.34). Supplementation with melatonin substantially increased serum total antioxidant capacity (WMD: 0.15 mmol/L, 95% CI: 0.08, 0.22) and high-density lipoprotein cholesterol (WMD: 2.04 mg/dL, 95% CI: 0.50, 3.57). No significant effects of melatonin were observed on body weight, waist circumference, body fat percentage, body mass index, fasting insulin, homeostasis model assessment of insulin resistance, hemoglobin A1c, triglycerides, diastolic blood pressure, aspartate aminotransferase, or gamma-glutamyl transferase. **Conclusions**: Melatonin supplementation significantly ameliorated multiple CMRFs.

## 1. Introduction

Cardiometabolic risk (CMR) refers to the interrelationship between cardiovascular and metabolic conditions, emphasizing the risk factors that contribute to diseases such as hypertension (HTN), cardiovascular disease (CVD), dyslipidemia, and diabetes mellitus (DM) [1]. Inflammation and oxidative stress (OS) are interconnected biological processes that profoundly affect cardiometabolic health (CMH) [2,3]. Elevated generation of reactive oxygen species (ROS) disrupts antioxidant defenses and promotes cellular injury [4], while chronic inflammation contributes to the pathogenesis of several diseases [5,6]. Their interplay accelerates the development of cardiometabolic diseases (CMDs) [3], promoting conditions such as obesity [7], insulin resistance (IR) [8], and CVD [9]. Elevated liver enzymes are also associated with OS and inflammation and are linked to CMR [10]. Given these mechanisms, antioxidant supplementation has emerged as a potential strategy for reducing CMR by attenuating oxidative and inflammatory damage [11,12,13,14,15,16,17]. Among various antioxidants, melatonin has attracted considerable interest due to its potent free radical scavenging activity and broad physiological effects [18,19,20].

Melatonin (N-acetyl-5-methoxytryptamine) is a hormone primarily synthesized by the pineal gland in a circadian pattern and regulates the circadian rhythm [21,22]. However, it is also produced in the mitochondria of nearly all somatic cells, where it contributes to the cellular redox balance [23,24]. It is also present in several dietary sources, including a variety of plant-derived foods (e.g., nuts, cereals, mushrooms) and some animal products (e.g., eggs, milk, fish) [25]. It acts as a potent antioxidant [26] and influences diverse physiological processes [27,28] through receptor-mediated and receptor-independent mechanisms [22,29,30,31,32].

Melatonin readily crosses biological membranes, scavenges free radicals [33], enhances antioxidant enzyme activity [34], and protects tissues from oxidative injuries [35]. Beyond sleep regulation [36], it has been associated with improved immune function [37] and possible protective effects against various diseases, including neurodegenerative [38] and gastrointestinal diseases [39]. Experimental and clinical studies suggest that melatonin supplementation may decrease inflammation and OS [40,41,42], reduce blood pressure (BP) [43], provide cardiac protection [44], and improve lipid and glucose metabolism [45,46,47,48]. However, clinical findings remain inconsistent across trials because of substantial variations in melatonin dosage, formulation, and intervention duration [49].

Several systematic reviews and meta-analyses have examined the impacts of melatonin supplementation on specific human health outcomes, including glycemic indices [50,51,52,53], BP [54,55,56,57,58], anthropometrics [55,59,60,61], lipid profile [61,62], liver enzymes [63], OS markers [64,65,66], and inflammatory parameters [40,41,67]. Although these studies provided valuable insights, their findings were inconsistent and mostly focused on single or limited cardiometabolic domains. Importantly, no prior meta-analysis has synthesized the comprehensive, multidimensional impact of melatonin on the full spectrum of integrated cardiometabolic risk factors (CMRFs), including anthropometric, glycemic, lipid, inflammatory, OS, and liver function parameters. Moreover, despite the large body of literature, there is still no definitive consensus on the overall effects of melatonin on CMH.

Although a few previous meta-analyses have explored dose–response relationships for selected individual outcomes, none have provided an integrated assessment across the major CMRFs. Given the substantial variability in melatonin dosage and treatment duration across trials, comprehensive evaluations of these dose–response and duration–response relationships are clinically important for identifying optimal supplementation strategies and explaining inconsistent findings in the literature. Therefore, this systematic review and dose–response meta-analysis of randomized controlled trials (RCTs) comprehensively evaluated the effects of melatonin supplementation on major CMRFs, providing updated and robust insights into these interconnected domains.

## 2. Materials and Methods

This systematic review and meta-analysis adhered to the PRISMA (Preferred Reporting Items for Systematic Reviews and Meta-Analyses) 2020 framework [68] and the Cochrane Handbook for Systematic Reviews of Interventions. Its protocol was registered in PROSPERO (International Prospective Register of Systematic Reviews) (registration number: CRD420251115809).

### 2.1. Search Strategy

A systematic search was performed by two investigators to identify RCTs published up to October 2025 in several databases (Scopus, Web of Science, and PubMed/MEDLINE). A gray literature search was conducted using Google Scholar and major clinical trial registries to identify relevant trials. The reference lists of related systematic reviews and all included trials were screened to capture any further eligible RCTs. No language or date restrictions were applied for searching and selecting RCTs.

The search strategy was structured according to the PICOS framework, encompassing the following components: population (adults), intervention (melatonin supplementation), comparator (control or placebo), outcomes (anthropometric measurements, BP, glycemic and lipid profiles, OS markers, inflammatory parameters, and liver function indicators), and study design. Anthropometric measurements included body mass index (BMI), hip circumference (HC), body fat percentage (BFP), waist circumference (WC), and body weight (BW). Lipid profile included high-density lipoprotein cholesterol (HDL-C), triglyceride (TG), low-density lipoprotein cholesterol (LDL-C), and total cholesterol (TC) levels. Glycemic control parameters were hemoglobin A1c (HbA1c), homeostatic model assessment of insulin resistance (HOMA-IR), fasting blood glucose (FBG), and fasting insulin (FI). BP was reported as diastolic (DBP) and systolic (SBP) blood pressure. Indicators of OS were malondialdehyde (MDA) and total antioxidant capacity (TAC). Inflammatory parameters were serum levels of interleukin-6 (IL-6), tumor necrosis factor-alpha (TNF-α), and C-reactive protein (CRP). Liver function parameters were gamma-glutamyl transferase (GGT), aspartate aminotransferase (AST), and alanine aminotransferase (ALT).

Search strategies were tailored for each database using Medical Subject Headings (MeSH) and non-MeSH keywords. Boolean operators (OR, AND) were applied to combine terms effectively and enhance the sensitivity and precision of the search. Table S1 presents the search strategy applied in PubMed.

### 2.2. Eligibility Criteria

EndNote reference management software was used to import and organize citations obtained from the databases. Two independent reviewers assessed and selected the RCTs based on the predefined inclusion criteria. Disagreements in study selection were addressed through discussion and consensus with a third investigator. The current systematic review and meta-analysis included RCTs that assessed the effects of melatonin supplementation on CMRFs by comparing the melatonin-treated group with the control or placebo group. Eligible studies employed parallel or crossover designs, had an intervention duration of at least two weeks, and reported baseline and post-intervention data for at least one CMRF using a pre–post design in both groups. RCTs in which melatonin was administered as part of a multi-component supplement were excluded, along with non-randomized studies, trials without a control or placebo group, and studies that included participants younger than 18 years or pregnant women.

### 2.3. Data Extraction

Two investigators independently extracted data from the included full-text articles, and any differences were addressed through discussion with the third investigator. Data were extracted using a standardized Excel-based extraction form developed and pilot-tested to ensure consistency between reviewers. The extracted information included several trial characteristics, such as the trial setting, publication year, study design, sample size, trial duration, first author’s name, and melatonin dosage. In addition, participants’ demographic variables, including sex, mean BMI, and age, were collected. The outcome measures were documented at the beginning and end of each intervention period. When full texts were not accessible or when outcome data were unclear or missing, the corresponding authors were contacted to obtain the required information.

### 2.4. Risk of Bias Assessment

Two researchers independently assessed the included RCTs using the Cochrane Risk of Bias tool (RoB 2) [69]. Any disagreements during the evaluation process were resolved through discussions with the third researcher. The tool evaluated five domains: detection bias, performance bias, randomization bias, attrition bias, and reporting bias. It categorized the RoB in each domain as high, low, or unclear [69].

### 2.5. Certainty Assessment

The certainty of the evidence was assessed using the Grading of Recommendations Assessment, Development, and Evaluation (GRADE) framework, which classified evidence into four levels: very low, low, moderate, and high quality. The assessment considered the five standard GRADE domains (publication bias, inconsistency, RoB, imprecision, and indirectness).

### 2.6. Statistical Analysis

STATA software (version 17) was used for statistical analyses. The mean values and standard deviations (SDs) reported in the RCTs were extracted and used to compute effect sizes as mean differences [70]. Effect estimates were presented as weighted mean differences (WMDs) with 95% confidence intervals (CIs) representing changes from baseline to post-intervention in both the melatonin and placebo groups. A random-effects model (DerSimonian and Laird method) was applied to pool effect sizes [70]. Heterogeneity among RCTs was examined using Cochran’s Q test and quantified with the I^2^ statistic, categorized as ≤25% (low), 26–50% (moderate), 51–75% (high), and >75% (very high) [71].

Subgroup analyses were performed to determine the potential sources of heterogeneity across the included studies. Baseline SBP, DBP, serum HDL-C, FBG, TG, LDL-C, and TC levels were categorized. The melatonin supplementation dose was classified into two categories (≤12 mg/day and >12 mg/day), and the intervention duration was divided into two periods (≤6 and >6 weeks). Participants were also grouped by baseline BMI (obesity, overweight, or normal) and sex (male, female, or both sexes). Leave-one-out sensitivity analyses were conducted to examine the impact of individual studies on the stability and robustness of the pooled estimates. Potential publication bias was assessed using Egger’s and Begg’s tests [72,73] and a visual inspection of funnel plots. A fractional polynomial model was applied to assess potential nonlinear dose–response relationships between melatonin dosage or study duration and changes in outcome measures. Meta-regression analyses were performed to explore possible linear associations between melatonin dosage or intervention duration and variations in the outcomes [74]. A *p*-value below 0.05 was considered statistically significant.

## 3. Results

### 3.1. Study Selection

**Figure 1:**
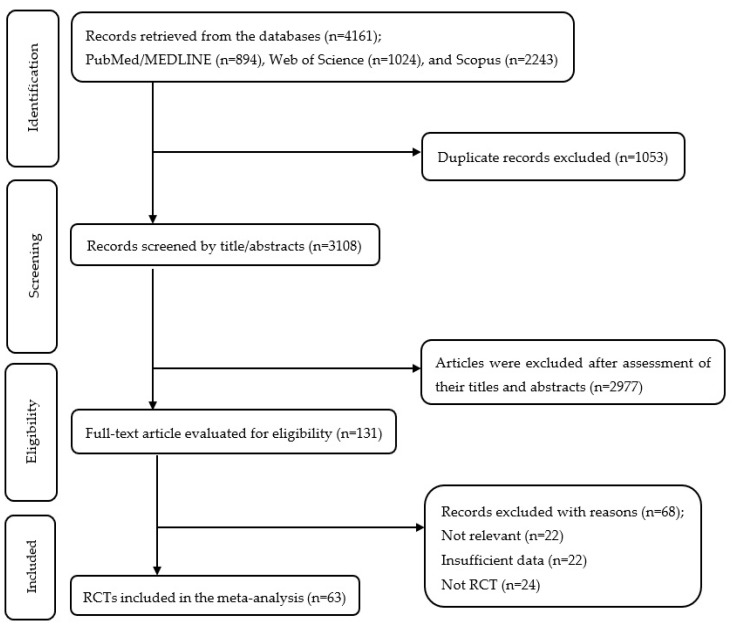
Flow diagram of study selection.

A systematic search across several databases identified 4161 records for screening. After removing 1053 duplicate entries, the abstracts and titles of 3108 records were screened, resulting in the exclusion of 2977 references. Subsequently, after assessing 131 full-text articles for eligibility, 63 RCTs were included in this meta-analysis. Figure 1 displays the flow diagram of trial selection and screening procedures.

### 3.2. Study Characteristics

The present systematic review and meta-analysis included 63 RCTs [75,76,77,78,79,80,81,82,83,84,85,86,87,88,89,90,91,92,93,94,95,96,97,98,99,100,101,102,103,104,105,106,107,108,109,110,111,112,113,114,115,116,117,118,119,120,121,122,123,124,125,126,127,128,129,130,131,132,133,134,135,136,137]. The trial characteristics are shown in Table 1. Of these trials, 60 employed a parallel [75,76,77,78,79,80,81,82,83,84,85,86,87,88,89,90,91,92,93,94,95,96,97,98,99,100,101,102,103,104,105,106,107,108,109,110,112,113,114,115,116,117,118,119,120,121,122,123,125,127,128,129,130,131,132,133,134,135,136,137], and three used a crossover design [111,124,126]. A total of 3157 participants were included (melatonin group, n = 1606; control group, n = 1551). The sample sizes of the trials varied between 14 and 158 participants. Their mean age ranged between 19 and 85 years, and their BMI ranged between 21.7 and 43 kg/m^2^. Among these trials, 41 involved mixed-sex participants [76,78,81,83,85,86,88,89,90,93,95,96,97,98,99,100,101,102,103,104,105,106,107,114,115,116,119,121,122,123,124,125,126,127,128,129,132,133,134,136,137], 16 included only female participants [75,77,79,80,82,91,92,94,108,109,110,113,117,118,131,135], and 6 consisted exclusively of male participants [84,87,111,112,120,130].

Trials have been conducted on diverse patients, including those with metabolic syndrome (MetS) [75,86,99,101], chronic kidney disease (CKD) [78,128], and polycystic ovary syndrome (PCOS) [80,117]. Additional studies have involved patients with coronavirus disease 2019 (COVID-19) [81,98,104], multiple sclerosis (MS) [106,129], type 2 diabetes mellitus (T2DM) with periodontal disease [83,88,133,135], and T2DM [89,96,111,124]. 

Patients with coronary artery disease (CAD) [123], chronic obstructive pulmonary disease (COPD) [119], heart failure with reduced ejection fraction (HFrEF) [105], ulcerative colitis (UC) [93], rheumatoid arthritis (RA) [95,97], nonalcoholic steatohepatitis (NASH) [100], nonalcoholic fatty liver disease (NAFLD) [85,90,121], and nocturnal HTN [102] were also included. Studies have also involved patients who underwent coronary artery bypass grafting (CABG) [107,137], and individuals with schizophrenia [115].

The RCTs have included patients with systemic lupus erythematosus (SLE) [118], renal ischemia–reperfusion injury (IRI) in transplant recipients [122], hypercholesterolemia [126], and diabetic nephropathy (DN) [132]. The participants were elderly patients with sarcopenia [127], hemodialysis patients [114,136], and patients treated with antipsychotic medications [76,134]. Furthermore, the trials were conducted among high-intensity trained athletes [120], resistance-trained athletes [112], women with comorbid conditions (such as overweight status, depression, and sleep disturbances) [77], individuals with overweight (OW) or obesity (OB) [79,116], postmenopausal women [82,91,92] with T2DM [108], perimenopausal women [110], shift workers [103,113], methamphetamine-dependent men [84], sedentary young men [87], women in the menopausal transition [94], women with insomnia [109], adults with obesity who participated in BW reduction programs [125], healthy men [130], and individuals with obesity who adhered to calorie-restricted diets [131].

The articles were published between 1997 and 2025. The RCTs were carried out in multiple countries, including Iran [76,77,79,80,81,84,85,86,88,89,95,96,98,99,105,107,108,115,116,117,118,121,122,124,128,132,133,135,136,137], Poland [90,91,92,93,100,123,131], Brazil [113,119,130], Italy [94,125,127], the United States of America (USA) [101,110,126], Iraq [75,78,104], Mexico [129,134], Tunisia [106,114], Denmark [82,111], Spain [112,120], Romania [83], India [87], the United Kingdom (UK) [97], Israel [102], South Korea [109], and Germany [103]. The length of these trials ranged from 2 to 56 weeks, and the daily melatonin supplementation doses ranged from 0.3 mg to 100 mg/day.

### 3.3. Meta-Analysis

**Figure 2:**
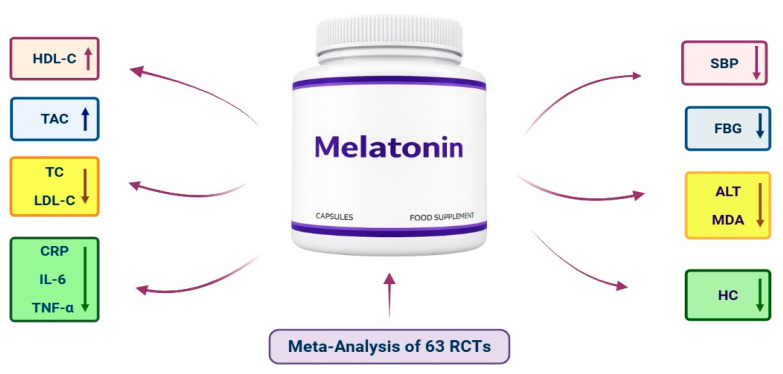
Summary of the effects of melatonin supplementation on cardiometabolic risk factors based on the meta-analysis of 63 RCTs. Arrows indicate the direction of effect (↑increase,↓ decrease).

The summary of the meta-analysis findings is shown in Figure 2 and Table 2. Various outcomes were assessed, including anthropometric (n = 5), glycemic (n = 4), lipid (n = 4), BP (n = 2), OS (n = 2), inflammatory (n = 3), and liver function parameters (n = 3).

#### 3.3.1. Impacts of Melatonin Supplementation on Anthropometric Parameters

**Figure 3:**
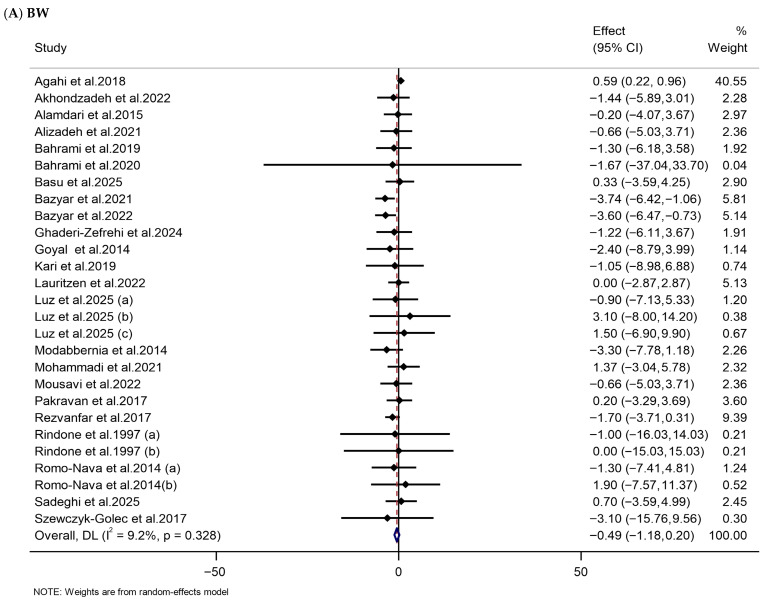

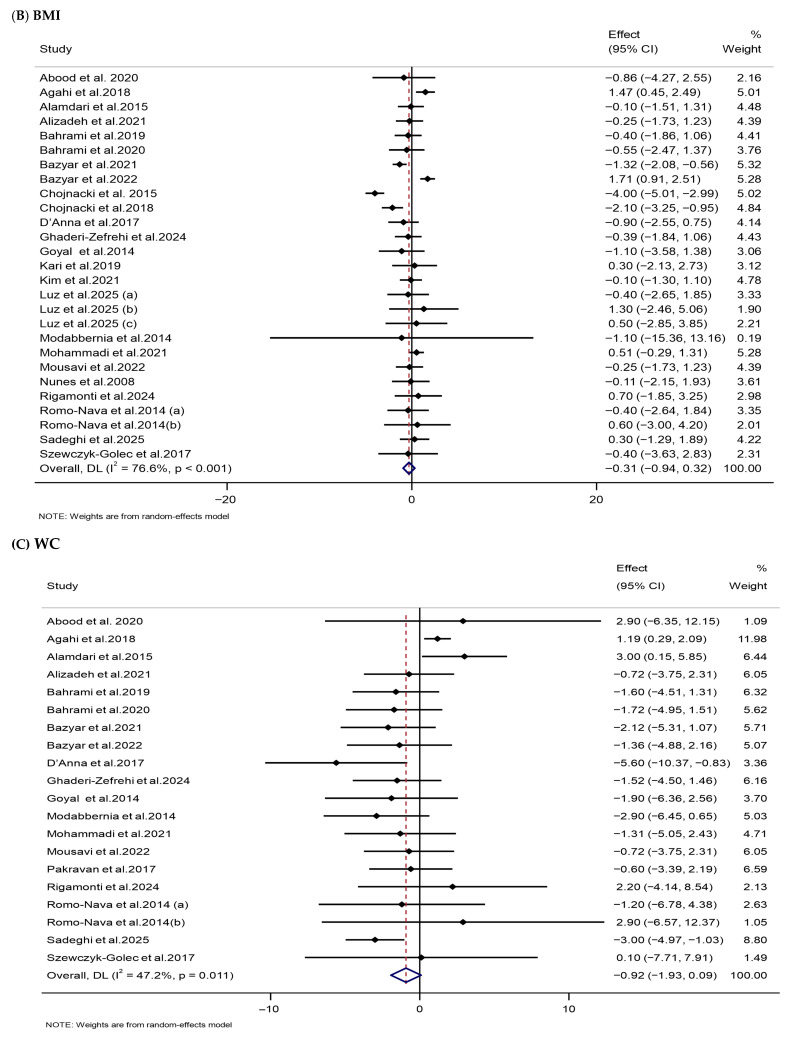

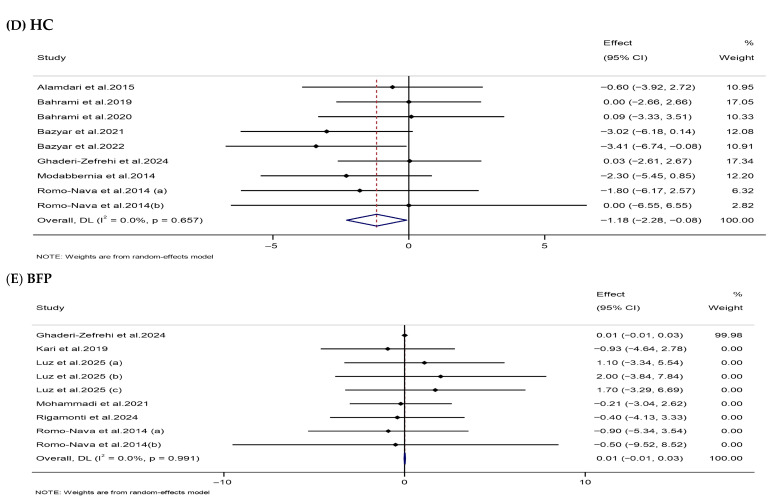
Forest plots illustrate weighted mean differences (WMDs) and 95% confidence intervals (CIs) for the effects of melatonin supplementation on anthropometric parameters, including (**A**) BW (kg), (**B**) BMI (kg/m^2^), (**C**) WC (cm), (**D**) HC (cm), and (**E**) BFP (%) [75,76,77,79,80,85,86,87,89,91,92,94,99,101,108,109,111,113,115,116,117,119,121,124,125,126,128,131,134,135].

This meta-analysis of RCTs revealed that melatonin supplementation substantially reduced HC in the melatonin-treated group compared to the placebo group (WMD: −1.18 cm, 95% CI: −2.28, −0.08). However, no statistically substantial impacts were observed on BW (WMD: −0.49 kg, 95% CI: −1.18, 0.20), BMI (WMD: −0.31 kg/m^2^, 95% CI: −0.94, 0.32), WC (WMD: −0.92 cm, 95% CI: −1.93, 0.09), and BFP (WMD: 0.01%, 95% CI: −0.01, 0.03) (Table 2, Figure 3).

Subgroup analyses indicated that melatonin supplementation substantially decreased BW in OW participants and reduced BMI of individuals in long-term trials (>12 weeks). In addition, WC declined following long-term supplementation with high melatonin doses (>6 mg/day) in OW participants. In contrast, HC decreased following short-term supplementation trials (≤12 weeks) with low melatonin doses (≤6 mg/day) in OW participants (Table S2).

#### 3.3.2. Impacts of Melatonin Supplementation on Glycemic Parameters

**Figure 4:**
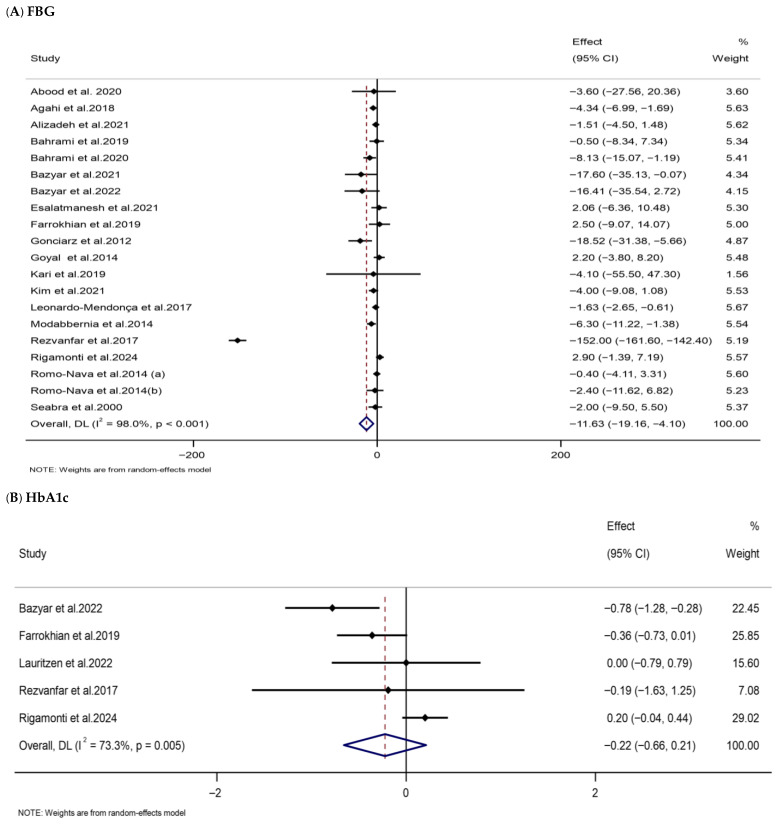

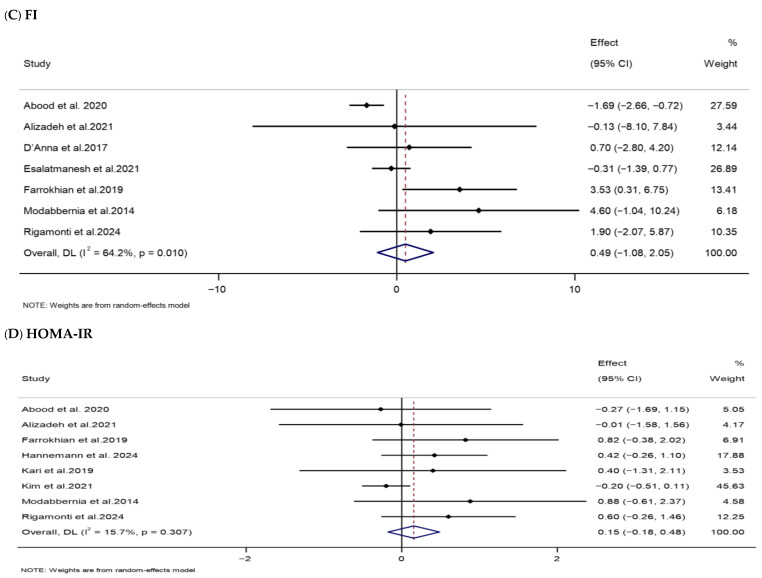
Forest plots illustrate weighted mean differences (WMDs) and 95% confidence intervals (CIs) for the effects of melatonin supplementation on glycemic parameters, including (**A**) FBG (mg/dL), (**B**) HbA1c (%), (**C**) FI (µIU/mL), and (**D**) HOMA-IR [75,76,80,85,86,89,94,95,96,100,101,103,108,109,111,112,115,124,125,134,135].

The pooled analysis displayed that melatonin supplementation substantially lowered serum FBG levels in the melatonin group compared with the placebo group (WMD: −11.63 mg/dL, 95% CI: −19.16, −4.10). However, there was a high degree of heterogeneity among the included trials (Table 2, Figure 4). No significant effects of melatonin were found on HOMA-IR (WMD: 0.15, 95% CI: −0.18, 0.48), serum FI levels (WMD: 0.49 µIU/mL, 95% CI: −1.08, 2.05), or HbA1c (WMD: −0.22%, 95% CI: −0.66, 0.21).

Subgroup analyses indicated that serum FBG levels were significantly reduced in both short- and long-duration trials (≤12 and >12 weeks) with low melatonin doses (≤6 mg/day) among male or mixed-sex participants with baseline FBG ≤100 mg/dL and normal BMI. Serum FI levels significantly decreased with high melatonin doses (>6 mg/day) in female participants. In addition, serum HbA1c levels were reduced in OW and female subgroups, whereas HOMA-IR decreased in mixed-sex participants (Table S2).

#### 3.3.3. Impacts of Melatonin Supplementation on Lipid Parameters

**Figure 5:**
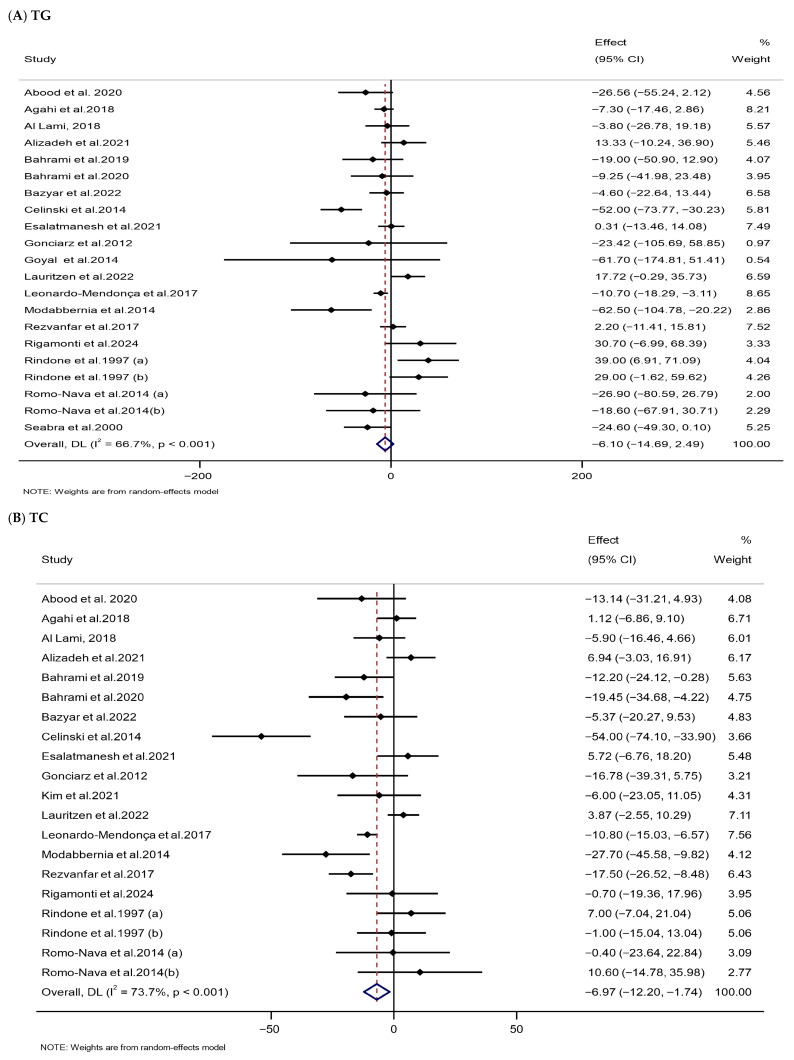

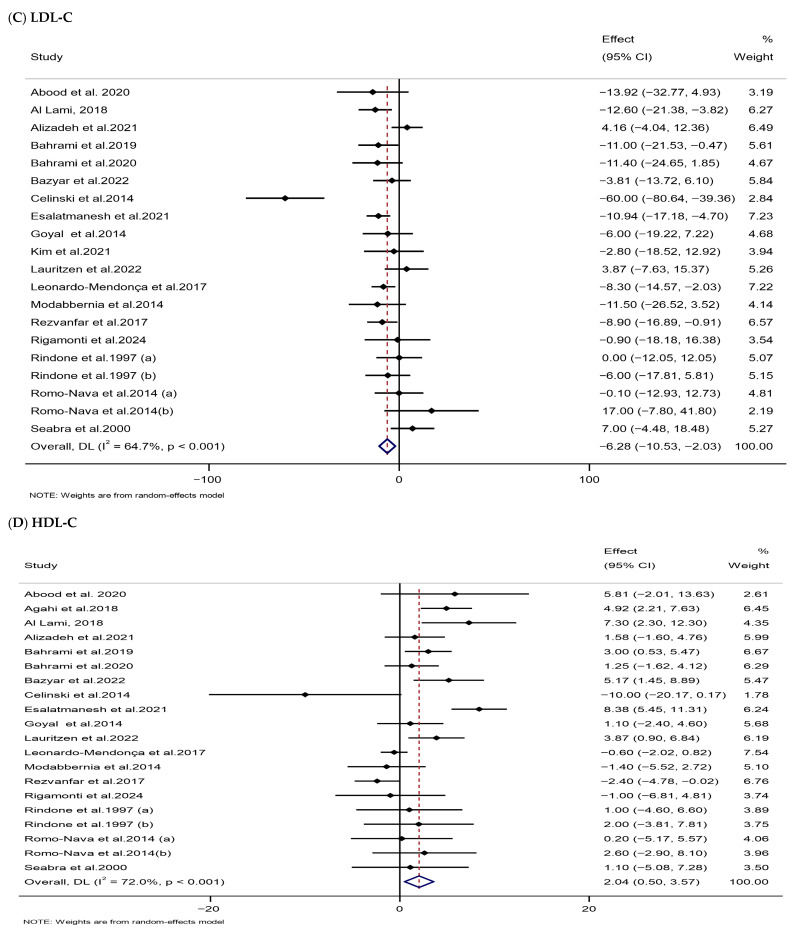
Forest plots illustrate weighted mean differences (WMDs) and 95% confidence intervals (CIs) for the effects of melatonin supplementation on lipid parameters, including (**A**) TG (mg/dL), (**B**) TC (mg/dL), (**C**) LDL-C (mg/dL), and (**D**) HDL-C (mg/dL) [75,76,78,80,85,86,90,95,100,101,109,111,112,115,124,125,126,130,134,135].

The meta-analysis indicated that melatonin supplementation significantly reduced serum TC (WMD: −6.97 mg/dL, 95% CI: −12.20, −1.74) and LDL-C levels (WMD: −6.28 mg/dL, 95% CI: −10.53, −2.03) in the melatonin group compared with the control group. HDL-C levels were substantially increased (WMD: 2.04 mg/dL, 95% CI: 0.50, 3.57) following melatonin supplementation. However, melatonin had no significant effect on TG levels (WMD: −6.10 mg/dL, 95% CI: −14.69, 2.49). Significant heterogeneity was observed among the included RCTs (Table 2 and Figure 5).

Subgroup analyses revealed that serum TG levels considerably decreased in long-duration trials (>12 weeks) with high melatonin doses (>6 mg/day) among participants with normal BMI. In addition, serum TC levels were significantly reduced in short-duration trials (≤12 weeks) with high melatonin doses (>6 mg/day) among mixed-sex participants with baseline TC >200 mg/dL and normal BMI or OB. Furthermore, serum LDL-C levels decreased in both short- and long-duration trials with low melatonin doses (≤6 mg/day) among mixed-sex participants with baseline LDL-C >100 mg/dL and normal BMI or OB. Additionally, serum HDL-C levels increased in short-duration trials with low melatonin doses in OW, OB, and female participants, as well as in those with baseline HDL-C levels ≤50 mg/dL (Table S2).

#### 3.3.4. Impacts of Melatonin Supplementation on Blood Pressure

**Figure 6:**
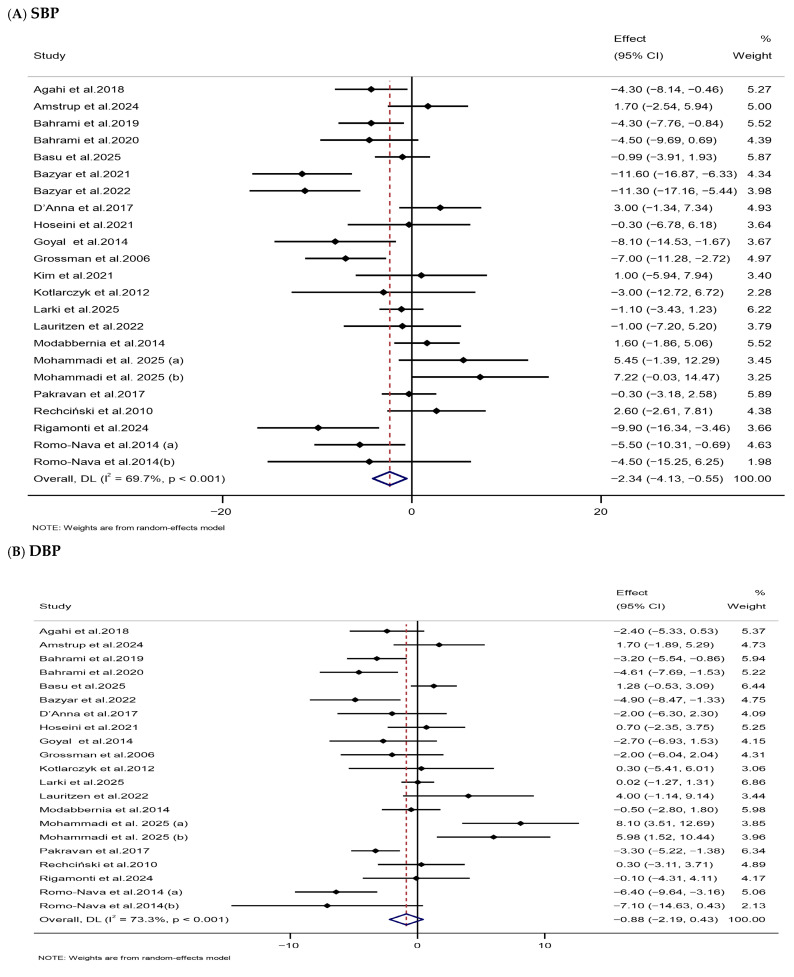
Forest plots illustrate weighted mean differences (WMDs) and 95% confidence intervals (CIs) for the effects of melatonin supplementation on blood pressure, including (**A**) SBP (mmHg) and (**B**) DBP (mmHg) [76,82,85,86,87,89,94,101,102,105,109,110,111,115,121,123,125,134,135,136,137].

The pooled analysis revealed that melatonin supplementation significantly reduced SBP (WMD: −2.34 mmHg; 95% CI: −4.13, −0.55), whereas no significant change was observed in DBP (WMD: −0.88 mmHg, 95% CI: −2.19, 0.43) in the melatonin-treated group versus the control group (Table 2, Figure 6). Heterogeneity among studies was significant for both SBP (*I*^2^ = 69.7%, *p* < 0.001) and DBP (*I*^2^ = 73.3%, *p* < 0.001) measurements. Subgroup analyses revealed that short-term supplementation (≤12 weeks) with low-dose melatonin (≤6 mg/day) substantially decreased SBP in mixed-sex participants with OB and baseline SBP > 130 mmHg. Melatonin also reduced DBP in low-dose trials among participants with OB (Table S2).

#### 3.3.5. Impacts of Melatonin Supplementation on Oxidative Stress Parameters

**Figure 7:**
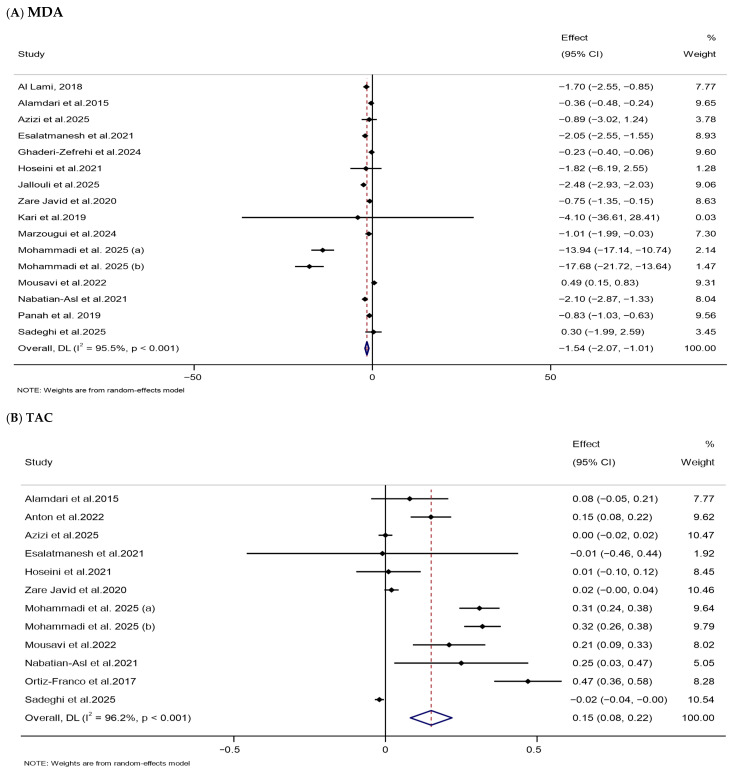
Forest plots illustrate weighted mean differences (WMDs) and 95% confidence intervals (CIs) for the effects of melatonin supplementation on oxidative stress parameters, including (**A**) MDA (μmol/L) and (**B**) TAC (mmol/L) [78,79,83,84,95,99,105,106,108,114,117,118,120,122,128,133,137].

The meta-analysis indicated that melatonin supplementation considerably lowered serum MDA levels (WMD: −1.54 μmol/L, 95% CI: −2.07, −1.01) and markedly increased serum TAC levels (WMD: 0.15 mmol/L, 95% CI: 0.08, 0.22) in the melatonin-treated group versus the untreated group. The included studies exhibited substantial heterogeneity (Table 2 and Figure 7).

Subgroup analyses revealed that melatonin supplementation significantly reduced serum MDA levels following short-term supplementation (≤12 weeks) with both low (≤6 mg/day) and high (>6 mg/day) doses in mixed-sex participants across different BMI categories (normal, OW, and OB). It increased serum TAC levels in short-term trials with low and high melatonin doses among female or mixed-sex participants with OW (Table S2).

#### 3.3.6. Impacts of Melatonin Supplementation on Inflammatory Parameters

**Figure 8:**
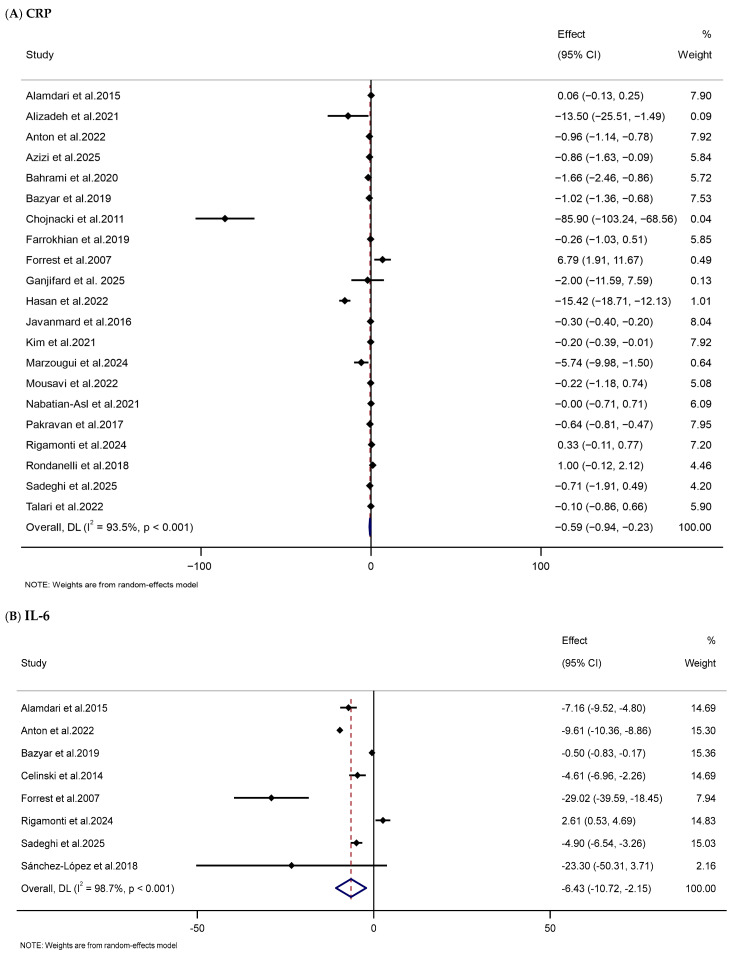

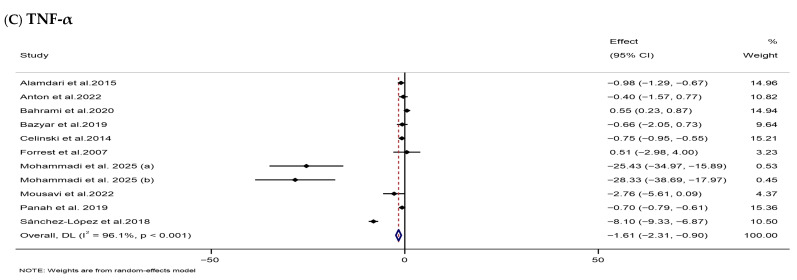
Forest plots illustrate weighted mean differences (WMDs) and 95% confidence intervals (CIs) for the effects of melatonin supplementation on inflammatory parameters, including (**A**) CRP (mg/L), (**B**) IL-6 (pg/mL), and **(C)** TNF-α (pg/mL) [79,81,83,84,85,88,90,93,96,97,98,104,107,109,114,117,118,121,122,125,127,128,129,132,137].

This meta-analysis indicated that melatonin supplementation substantially decreased serum levels of CRP (WMD: −0.59 mg/L, 95% CI: −0.94, −0.23), IL-6 (WMD: −6.43 pg/mL, 95% CI: −10.72, −2.15), and TNF-α (WMD: −1.61 pg/mL, 95% CI: −2.31, −0.90) in the melatonin-treated group compared to those in the control group. However, the trials exhibited substantial heterogeneity (Table 2 and Figure 8).

Subgroup analyses revealed that melatonin supplementation considerably reduced serum CRP levels in both long- and short-term supplementation (>12 and ≤12 weeks) with high (>6 mg/day) and low (≤6 mg/day) doses among male or mixed-sex participants with normal BMI or OW. In addition, serum IL-6 levels decreased after supplementation with high melatonin doses (>6 mg/day) in female or mixed-sex participants. Furthermore, serum TNF-α levels were reduced after short-term supplementation with high melatonin doses (>6 mg/day) in mixed-sex participants with normal BMI or OW (Table S2).

#### 3.3.7. Impacts of Melatonin Supplementation on Liver Function Markers

**Figure 9:**
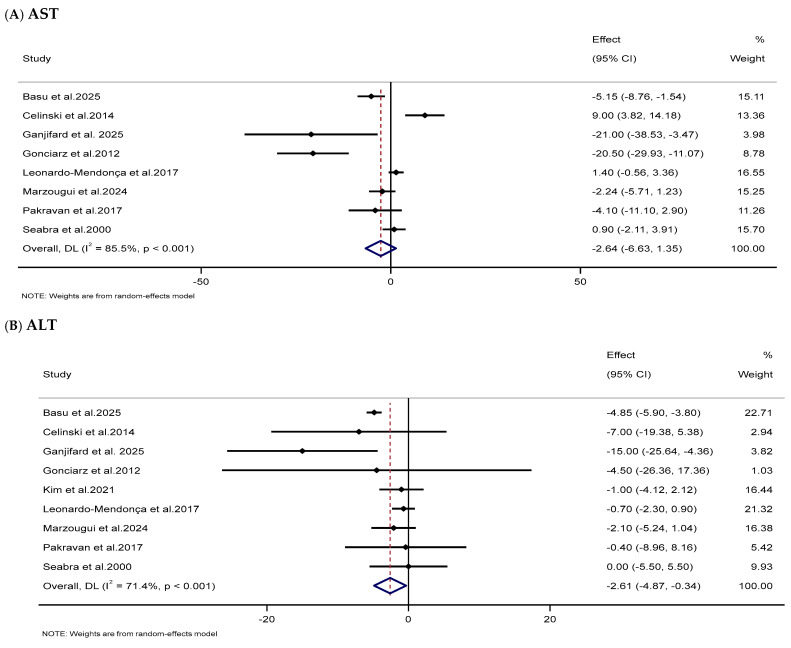

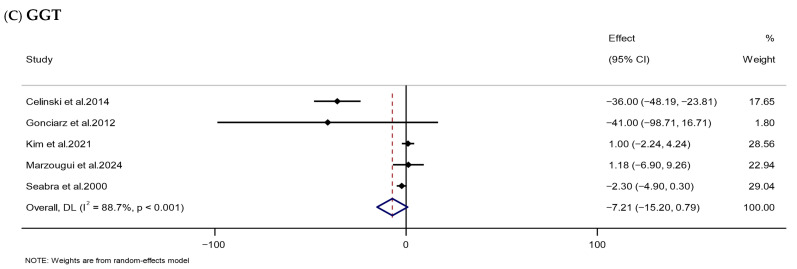
Forest plots illustrate weighted mean differences (WMDs) and 95% confidence intervals (CIs) for the effects of melatonin supplementation on liver function markers, including (**A**) AST (IU/L), (**B**) ALT (IU/L), and (**C**) GGT (IU/L) [87,90,98,100,109,112,114,121,130].

The meta-analysis revealed that melatonin supplementation substantially reduced serum ALT (WMD: −2.61 IU/L, 95% CI: −4.87, −0.34) levels in the melatonin-treated group compared to the untreated group. However, no substantial impact was found on AST levels (WMD: −2.64 IU/L, 95% CI: −6.63, 1.35) and GGT (WMD: −7.21 IU/L, 95% CI: −15.20, 0.79) (Table 2 and Figure 9).

Subgroup analyses revealed that short-term supplementation with low melatonin doses (≤6 mg/day) significantly reduced serum ALT concentrations in participants with normal baseline BMI. In addition, reductions in serum AST levels were observed with low melatonin doses (≤6 mg/day), and serum GGT levels significantly decreased after long-term melatonin administration (Table S2).

### 3.4. Risk of Bias Evaluation

The RoB of the included RCTs is summarized in Table S3. Ten trials [78,81,87,90,92,94,104,108,123,126] were considered to have high RoB, primarily due to issues with randomization and deviations from the intended intervention. In contrast, 47 trials [76,77,79,80,82,83,84,85,86,88,89,91,93,95,96,97,98,99,101,102,103,105,106,107,109,111,112,113,114,115,116,117,118,119,120,121,122,125,127,128,129,131,133,134,135,136,137] were deemed low RoB, while six trials [75,100,110,124,130,132] raised some concerns.

### 3.5. GRADE

Table S4 presents the GRADE of outcomes. High-certainty evidence was detected for the effects of melatonin on WC, HOMA-IR, and BFP. The evidence for BW, HC, BMI, HbA1c, and CRP was rated as moderate quality. Additionally, FBG, FI, TG, HDL-C, SBP, TC, DBP, TAC, LDL-C, TNF-α, AST, IL-6, GGT, and ALT outcomes were downgraded to low-quality evidence. Furthermore, the GRADE score for the impact of melatonin on MDA levels was very low.

### 3.6. Sensitivity Analysis

Sensitivity analyses revealed that the results remained robust after excluding specific trials for BMI, FI, BFP, HDL-C, HOMA-IR, TG, FBG, TC, LDL-C, SBP, MDA, TAC, IL-6, CRP, and TNF-α. However, exclusion of certain trials altered the outcomes for BW [76], HbA1c [125], AST [90], ALT [87,98], DBP [137], GGT [114], WC [76,79], and HC [89,115,135].

### 3.7. Publication Bias

Funnel plot inspection revealed asymmetry for all outcomes (Figure S1). Egger’s test revealed publication bias for BW, TAC, and MDA. In contrast, no bias was detected for the other outcomes using Egger’s or Begg’s test.

### 3.8. Linear and Nonlinear Dose–Response Associations

No linear (Figures S4 and S5) or nonlinear (Figures S2 and S3) associations were observed between melatonin doses or trial durations and mean changes in BW, HC, FBG, HbA1c, FI, HOMA-IR, DBP, MDA, and TNF-α levels. Nonlinear dose–response relationships were identified between melatonin doses and mean changes in BFP (−0.04, *p* = 0.028), TG (−13.23, *p* = 0.032), SBP (−2.97, *p* = 0.036), TAC (0.11, *p* < 0.001), CRP (−2.33, *p* = 0.001), AST (−1.72, *p* = 0.004), and IL-6 (−1.65, *p* < 0.001) (Figure S2), as well as between trial durations and changes in BMI (−0.11, *p* = 0.027) and ALT (−2.06, *p* = 0.015) (Figure S3). Linear meta-regression analyses revealed that trial durations were associated with mean changes in BMI (−4.33, *p* = 0.003), WC (−1.28, *p* = 0.002), TC (−0.41, *p* = 0.010), LDL-C (−0.53, *p* = 0.001), HDL-C (−1.27, *p* = 0.028), CRP (−0.33, *p* = 0.017), and GGT (−1.25, *p* = 0.017) levels (Figure S5).

## 4. Discussion

### 4.1. Summary of Findings

This meta-analysis of 63 RCTs revealed that melatonin supplementation substantially reduced HC, SBP, and serum levels of FBG, TC, LDL-C, CRP, MDA, TNF-α, IL-6, and ALT. It also significantly increased serum TAC and HDL-C levels. No significant effects of melatonin were observed on BW, WC, BFP, BMI, FI, HOMA-IR, HbA1c, TG, DBP, GGT, or AST.

Subgroup analyses revealed that melatonin supplementation significantly decreased BW in participants with OW and reduced BMI of individuals in long-term trials (>12 weeks). WC declined following long-term supplementation with high melatonin doses (>6 mg/day) in OW participants, whereas HC decreased in short-term melatonin supplementation (≤12 weeks) with low doses (≤6 mg/day) in OW participants. Serum FBG levels were substantially reduced with low melatonin doses in male or mixed-sex participants with baseline FBG ≤100 mg/dL and normal BMI. FI levels significantly decreased with high melatonin doses in female participants, HbA1c levels were reduced in those with OW and female subgroups, and HOMA-IR decreased in mixed-sex participants. Serum TG levels were significantly reduced in long-duration trials with high melatonin doses among participants with normal BMI. Serum TC values decreased in short-duration trials with high doses among mixed-sex participants with baseline TC >200 mg/dL and normal BMI or OB. Serum LDL-C decreased with low melatonin doses in mixed-sex participants with baseline LDL-C >100 mg/dL and normal BMI or OB, while HDL-C increased following short-duration trials with low doses in female participants and those with OW, OB, and baseline HDL-C ≤50 mg/dL.

Subgroup analyses also showed that short-term low-dose melatonin supplementation decreased SBP in mixed-sex participants with OB and baseline SBP > 130 mmHg, and it reduced DBP following low-dose trials in participants with OB. It significantly reduced serum MDA levels in participants following short-term trials and increased serum TAC levels in short-term trials among female or mixed-sex participants with OW. Melatonin supplementation substantially reduced serum CRP levels in male participants and those with normal BMI or OW. Serum IL-6 levels decreased following high-dose melatonin supplementation in female or mixed-sex participants, while TNF-α decreased in short-term high-dose trials among mixed-sex participants with OW or normal BMI. Short-term supplementation with low melatonin doses significantly reduced ALT in participants with normal BMI. Serum AST levels of participants were also reduced with low doses of melatonin, and GGT levels significantly decreased after long-term melatonin supplementation.

Dose–response analyses revealed nonlinear associations between melatonin doses and changes in BFP, TG, SBP, CRP, AST, IL-6, and TAC. Nonlinear relationships were also observed between trial durations and changes in BMI and ALT. Linear meta-regression analyses further showed associations between trial durations and changes in BMI, WC, TC, LDL-C, CRP, HDL-C, and GGT.

### 4.2. Findings in the Context of Existing Literature

Previous meta-analyses have reported inconsistent results regarding the impacts of melatonin supplementation on anthropometric parameters. A meta-analysis of 23 studies indicated that melatonin supplementation substantially decreased BW [59]. However, another meta-analysis of seven trials found no effect on BW [60]. A meta-analysis found no significant effects of melatonin on anthropometric parameters [61]. One meta-analysis reported that melatonin supplementation substantially lowered HC but had no significant impact on other anthropometric parameters [55], which was similar to the findings of the present study. Additionally, a review suggested that melatonin may influence energy metabolism by regulating glycemic homeostasis, lipid metabolism, mitochondrial function, and adipose tissue remodeling, thereby reducing adiposity [138].

Prior evidence related to the effects of melatonin on glycemic control has also been inconsistent. A meta-analysis of 16 RCTs indicated that melatonin supplementation decreased FBG, HbA1c, and IR [50]. Another meta-analysis of eight RCTs reported improvements in hyperinsulinemia, IR, and insulin sensitivity following melatonin supplementation [51]. However, a systematic review of 15 RCTs and 18 animal studies suggested that melatonin supplementation may reduce HOMA-IR or FI levels without affecting FBG [52]. Moreover, a recent meta-analysis of nine RCTs revealed that melatonin supplementation substantially decreased HbA1c levels in patients with T2DM but had no substantial effect on FBG [53]. In contrast, the present meta-analysis found a significant decrease in FBG levels and no substantial effects on HOMA-IR, FI, and HbA1c. This discrepancy may arise because the current analysis included a larger proportion of adults without advanced T2DM, whose glycemic physiology may be more responsive to melatonin in the fasting state but less susceptible to changes in long-term markers such as HbA1c or insulin dynamics.

In terms of lipid profile, a meta-analysis of eight RCTs reported that melatonin supplementation considerably lowered TG and TC levels [62], particularly at higher doses and longer durations in participants with elevated baseline TC [62]. Another meta-analysis of 12 trials reported that melatonin substantially decreased LDL-C and TG levels, although no significant impact on HDL-C was observed [61]. The present meta-analysis revealed that melatonin supplementation lowered serum LDL-C and TC levels and increased serum HDL-C values. These differences may reflect the inclusion of several recent trials with longer follow-up periods and participants with borderline or mildly elevated baseline lipid levels, which may have allowed the HDL-C response to be observed more clearly than in earlier reviews.

Regarding BP regulation, a meta-analysis of five RCTs reported that melatonin can reduce both SBP and DBP [54]. Conversely, another meta-analysis found a reduction in DBP only, with no significant change in SBP [55]. However, a meta-analysis reported no significant differences in SBP or DBP after melatonin supplementation [105]. In contrast, the current meta-analysis demonstrated a substantial reduction in SBP but no change in DBP, which indicated a modest but potentially clinically relevant antihypertensive effect of melatonin. A review of clinical studies reported that melatonin supplementation can significantly lower BP in healthy individuals and those with HTN, suggesting its potential as an adjunctive antihypertensive treatment [139]. The reduction in SBP observed in this study may be related to the presence of participants with isolated systolic elevation and the longer duration of interventions in the included trials, which could explain why earlier reviews reported inconsistent patterns across SBP and DBP outcomes.

Previous studies have consistently revealed the antioxidant effects of melatonin in OS. A meta-analysis of 15 RCTs reported that melatonin administration significantly reduced MDA levels and increased TAC [66]. Similarly, other meta-analyses reported significant increases in TAC [64,65] and reductions in MDA [64,67]. The alignment across reviews may be attributed to the relatively homogeneous measurement methods of OS biomarkers and the strong mechanistic basis for melatonin’s antioxidant activity, which reduces the likelihood of divergent findings.

Regarding inflammatory biomarkers, a meta-analysis across diverse age groups (from neonates to older adults) reported that melatonin exerted significant anti-inflammatory effects on IL-6 [41]. However, no substantial impact on CRP levels was observed [41]. A meta-analysis of 13 clinical trials revealed that melatonin supplementation significantly decreased IL-6 and TNF-α expressions [40]. In addition, a meta-analysis of 14 clinical trials displayed that melatonin effectively reduced chronic inflammation, as evidenced by significant reductions in CRP, TNF-α, and IL-6 levels [67]. The findings of the current meta-analysis align closely with these reports, as melatonin supplementation substantially decreased serum CRP, TNF-α, and IL-6 levels, confirming its strong anti-inflammatory potential in diverse clinical populations. Differences across earlier reviews, especially regarding CRP, may reflect variations in baseline inflammatory status and the inclusion of acute versus chronic conditions.

Evidence regarding the effects of melatonin on liver function is limited and inconsistent. A meta-analysis of five RCTs reported that melatonin significantly elevated AST levels and decreased GGT levels in patients with NAFLD, with no substantial effect on serum ALT levels [63]. In contrast, this meta-analysis displayed considerable reductions in ALT levels but no significant changes in AST or GGT. These ALT reductions suggest that melatonin may confer hepatoprotective effects beyond NAFLD, potentially due to the inclusion of adults with milder liver enzyme elevations who may respond more readily to melatonin’s antioxidant and anti-inflammatory properties.

Some discrepancies between the findings of this review and those of previous meta-analyses likely reflect these methodological differences. Several earlier reviews included non-randomized trials, whereas the present analysis was restricted to RCTs and incorporated a larger number of recently published studies. In addition, some prior reviews employed standardized mean differences (SMDs), whereas the use of WMDs in this review facilitated a more direct clinical interpretation of the results. Beyond methodological factors, conflicting findings have also been reported across individual trials and previous reviews, with some studies showing null or weaker effects of melatonin on lipid, glycemic, and inflammatory outcomes. These inconsistencies may reflect variations in the baseline metabolic status, intervention duration, melatonin dosage, adherence, and measurement methods.

### 4.3. Possible Underlying Mechanisms

The effects of melatonin as a modulator of body composition have been discussed in previous studies [140,141]. Melatonin influences appetite regulation [142,143], enhances energy expenditure by stimulating brown adipose tissue activity and thermogenic pathways [144], and modulates the circadian control of metabolism [47,145], thereby contributing to improvements in obesity-related metabolic outcomes [146]. Its antioxidant and endocrine-modulating properties affect lipid metabolism and fat storage [147]. Experimental studies have revealed that melatonin supplementation may prevent weight gain by decreasing lipogenesis, enhancing lipolysis, and reducing inflammation in adipose tissue [148,149,150,151].

The positive effects of melatonin on FBG levels observed in this meta-analysis may be attributed to several mechanisms. Melatonin modulates the endogenous circadian system, which plays a pivotal role in insulin sensitivity and glucose metabolism [149,152]. Low circulating melatonin levels are associated with hyperinsulinemia and glucose intolerance, further emphasizing the circadian component of glucose homeostasis [152]. It also exhibits potent antioxidative and anti-inflammatory properties that mitigate OS and systemic inflammation, which are two major contributors to IR and β-cell dysfunction [153,154,155,156].

The beneficial effects of melatonin on serum TC, LDL-C, and HDL-C levels detected in this meta-analysis may be attributed to several mechanisms. Melatonin reduces intestinal cholesterol absorption, contributing to lower circulating TC levels [157]. In addition, it exhibits antioxidative effects that limit LDL-C oxidation (168), which, together with its membrane-stabilizing properties [158], may collectively account for the decline in LDL-C and TC levels. It may further improve HDL-C levels through its antioxidative and anti-inflammatory properties, which enhance HDL-C functionality and support reverse cholesterol transport [146].

Melatonin regulates BP through both central and peripheral mechanisms. Peripherally, it enhances endothelial function and promotes vasodilation, partly through pathways involving nitric oxide (NO) production [139,159,160,161]. This NO-mediated vasodilatory action contributes to the improvement of vascular reactivity and overall BP reduction in the body. Furthermore, it exhibits strong antioxidant properties, scavenging free radicals and attenuating OS, which are key contributors to endothelial dysfunction and vascular stiffness [139,162,163]. Through these antioxidative and vasoregulatory effects, melatonin supports vascular health and helps maintain normal BP.

Melatonin possesses strong antioxidant and anti-inflammatory properties. These actions reduce OS and lipid peroxidation, contributing to decreased MDA levels and improved TAC [164,165,166]. In addition, it regulates inflammatory signaling pathways, leading to reductions in acute-phase proteins (e.g., CRP) and pro-inflammatory cytokines (e.g., TNF-α and IL-6) [41,167,168,169]. It has a positive effect on liver function biomarkers [63]. By mitigating OS, inflammation, and hepatocyte apoptosis, melatonin helps maintain normal liver enzyme levels and supports overall hepatic functions.

These mechanisms should be interpreted as potential hypothesis-based explanations derived from prior biological and clinical evidence. They are not indicative of causal pathways confirmed by this meta-analysis but rather provide context for how melatonin may influence observed outcomes.

### 4.4. Safety of Melatonin Supplements

A review indicated that short-term melatonin supplementation is safe, even at high doses [170]. Mild adverse effects may occur, including headache, dizziness, nausea, and drowsiness [170]. Another review reported that melatonin supplementation generally has a favorable safety profile [171]. In addition, limited adverse event reporting has been observed in studies involving high-dose melatonin [172]. Based on this limited evidence, melatonin appears to have a good safety profile [172]. Moreover, phase 1 pharmacological trials have demonstrated no toxicity in healthy volunteers receiving melatonin doses of ≤100 mg [173]. However, there is a broad consensus that the long-term effects of high-dose melatonin, both beneficial and adverse, are not yet fully understood and require further investigation [52,174]. Although melatonin is generally considered safe based on short-term and early-phase clinical data, robust long-term safety evidence, particularly for higher doses and in individuals with chronic diseases, remains limited. It should also be noted that adverse event reporting was inconsistent across the included RCTs, which may have led to an underestimation of the true incidence of side effects associated with melatonin supplementation. Given that this meta-analysis did not evaluate adverse events or safety outcomes, no conclusions can be drawn regarding the safety of melatonin supplementation. Future RCTs should prioritize long-term follow-up to establish optimal melatonin supplementation dosing and duration, with systematic assessment of safety and adverse effects across diverse populations.

### 4.5. Clinical Implications

This meta-analysis revealed that melatonin supplementation exerts beneficial effects on multiple CMRFs. Greater improvements were observed in individuals with overweight or obesity and those with mildly elevated metabolic markers. The results may also be relevant for populations with circadian disturbances, such as older adults with reduced endogenous melatonin secretion. These findings highlight the potential clinical relevance of melatonin as part of an integrative strategy for reducing CMD risk. However, key practical considerations, such as optimal dose, timing of administration, formulation differences, and appropriate patient selection, remain insufficiently defined, indicating that further evidence is needed before routine clinical integration can be recommended.

### 4.6. Strengths and Limitations

This is the first comprehensive dose–response meta-analysis to evaluate the impacts of melatonin supplementation on major integrated CMRFs. The outcomes examined covered a wide range of anthropometric, glycemic, and lipid parameters, as well as BP, OS, inflammatory markers, and liver function indicators. Earlier reviews have generally examined individual outcomes, whereas this study synthesized data from 63 RCTs to provide a comprehensive cardiometabolic perspective. These results provided updated and robust evidence for the multifaceted role of melatonin in CMRFs.

This study had several methodological strengths. It employed a systematic search strategy without restrictions on language or publication date, thereby including a substantial number of relevant trials. The analysis included subgroup and sensitivity analyses, linear and nonlinear dose–response evaluations, and assessments of publication bias that enhanced the robustness of the findings. Forty-seven trials were deemed to have low RoB, and only a small proportion showed suboptimal quality. Importantly, only RCTs were included, and based on the GRADE assessment, half of the outcomes demonstrated high-to-moderate certainty of evidence. In addition to these methodological strengths, the inclusion of a wide spectrum of CMRFs offers an important interpretative advantage by enabling a more integrated evaluation of melatonin’s potential cardiometabolic effects.

This study had several limitations. The included trials exhibited methodological heterogeneity and variability in clinical characteristics, including differences in intervention duration, melatonin dosage, sample size, and participants’ health status. The control groups varied across studies, contributing to variability in outcomes. Moreover, there was a lack of RCTs examining specific parameters such as FI, GGT, HC, HOMA-IR, BFP, AST, ALT, IL-6, and HbA1c. These factors should be considered when interpreting these results. 

It is also important to note that several potentially influential variables, including habitual sleep patterns, sleep quality, and circadian timing of melatonin intake, were not consistently reported across the included RCTs. The absence of these data limits the ability to account for their confounding effects, despite their known relevance to melatonin physiological actions. Additionally, the participants included in the RCTs were highly heterogeneous, ranging from individuals with diverse underlying diseases to otherwise healthy participants. This broad clinical diversity introduces substantial variation in the baseline metabolic status, inflammatory and OS levels, disease-specific pathophysiology, and concurrent medication use, all of which may influence the magnitude and direction of melatonin’s effects. Because such heterogeneity cannot be fully resolved through subgroup analyses, particularly when subgroups contain few studies, pooled estimates should be interpreted with caution, as they may not accurately represent the effect of melatonin within any single, clinically defined population. Nevertheless, the use of random-effects models and sensitivity analyses helped account for this variation, and the observed heterogeneity did not materially alter the overall direction of the findings.

While previous meta-analyses have evaluated the effects of melatonin supplementation on various health outcomes, this study is remarkable for its comprehensive synthesis of the evidence and updated consensus on CMRFs. This comprehensive approach provides a refined understanding and valuable insights into the therapeutic capabilities of melatonin.

## 5. Conclusions

This meta-analysis of 63 RCTs revealed that melatonin supplementation produced modest but statistically significant improvements in several CMRFs, including HC, IL-6, SBP, TC, FBG, HDL-C, MDA, TAC, CRP, LDL-C, TNF-α, and ALT. However, no significant effects were observed on BW, WC, BFP, BMI, FI, HOMA-IR, HbA1c, TG, DBP, GGT, or AST. While these findings indicate broad therapeutic potential, the overall effect sizes were small, and important uncertainties persist regarding long-term efficacy, optimal dosing, formulation, and timing of administration. Therefore, the clinical significance of melatonin should be interpreted with caution until stronger evidence becomes available. In addition, because this meta-analysis did not evaluate adverse events, no conclusions can be drawn regarding the safety of melatonin supplementation. Future RCTs should prioritize the long-term evaluation of melatonin supplementation, carefully considering circadian timing, sleep-related factors, and other variables that may influence treatment response, along with systematic assessments of safety outcomes.

## Figures and Tables

**Table 1:** Characteristics of included studies in the meta-analysis.

Reference	Study Region	Study Design	Participants	Sex	Sample Size		Trial Duration (Weeks)	Mean Age		Mean BMI		Intervention	
					IG	CG		IG	CG	IG	CG	Melatonin Dose (mg/Day)	CG
Abood et al. 2020 [75]	Iraq	P, R, DB, PC	Women with MetS	♀	20	15	12	45.8 ± 6.5	48.0 ± 7.4	40.2 ± 6.9	41.8 ± 8.8	10	PL (lactose)
Agahi et al. 2018 [76]	Iran	P, R, DB, PC	Patients treated with antipsychotics	(♀♂)	50	50	8	37.4 ± 10.3	37.4 ± 12.4	NR	NR	3	PL
Akhondzadeh et al. 2022 [77]	Iran	P, R, DB, PC	Women with comorbidities (OW, depression)	♀	21	22	12	35.3 ± 10.6	38.5 ± 8.7	33 ± 5.4	33 ± 5.4	3	PL
Al Lami, 2018 [78]	Iraq	P, R, SB, PC	Patients with CKD	(♀♂)	21	20	12	58.2 ± 15.6	56.1 ± 10.7	NR	NR	5	PL
Alamdari et al. 2015 [79]	Iran	P, R, DB, PC	Women with OB	♀	22	22	6	33.8 ± 6.9	34.8±7.2	34.1 ± 3.2	35.7 ± 4.1	6	PL (excipients)
Alizadeh et al. 2021 [80]	Iran	P, R, DB, PC	Women with PCOS	♀	21	20	8	25.5 ± 4.9	26.2 ± 5.7	28.4 ± 3.8	26.9 ± 3.8	6	PL
Alizadeh et al. 2021 [81]	Iran	P, R, SB, CO	Patients with COVID-19	(♀♂)	14	17	2	37.5 + 8.2	34.5 + 8.2	NR	NR	6	Regular medications
Amstrup et al. 2024 [82]	Denmark	P, R, DB, PC	Postmenopausal women	♀	17	16	12	63 ± 4.5	64 ± 5	26.3 ± 4.1	24.2 ± 5.5	10	PL
Anton et al. 2022 [83]	Romania	P, R, DB, PC	Patients with T2DM and PD	(♀♂)	25	25	8	30–60	30–60	NR	NR	6	PL (excipients)
Azizi et al. 2025 [84]	Iran	P, R, DB, CO	Methamphetamine-dependent men	♂	23	23	4	18–55	18–55	NR	NR	10	NI
Bahrami et al. 2019 [86]	Iran	P, R, DB, PC	Patients with MetS	(♀♂)	36	34	12	42.5± 9.8	42.6± 10.2	31.0 ± 4.9	32.1 ± 4.9	6	PL (starch)
Bahrami et al. 2020 [85]	Iran	P, R, DB, PC	Patients with NAFLD	(♀♂)	24	21	12	44 ± 9.6	37.7± 11.3	29.4 ± 3.6	32.5 ± 6.1	6	PL (starch)
Basu et al. 2025 [87]	India	P, R, SB, PC	Sedentary men	♂	14	14	4	23.2 ± 1.3	22.5 ± 1.0	NR	NR	3	PL (starch)
Bazyar et al. 2019 [88]	Iran	P, R, DB, PC	Patients with T2DM & PD	(♀♂)	22	22	8	53.7 ± 6.6	51.4 ± 5.0	NR	NR	6	PL (excipients)
Bazyar et al. 2021 [89]	Iran	P, R, DB, PC	Patients with T2DM	(♀♂)	25	25	8	53.6 ± 4.8	51.5 ± 6.3	27.3 ± 2.1	27.4 ± 2.0	6	PL (peppermint oil)
Bazyar et al. 2022 [135]	Iran	P, R, DB, PC	Patients with T2DM & PD under NSPT	♀	22	22	8	53.7 ± 6.6	51.4 ± 5.0	27.3 ± 2.1	27.2 ± 2.1	6	PL (starch)
Celinski et al. 2014 [90]	Poland	P, R, PC	Patients with NAFLD	(♀♂)	23	23	56	36.1 ± 5.7	29.3 ± 9.5	NR	NR	10	PL (liver health supplement)
Chojnacki et al. 2011 [93]	Poland	P, R, DB, PC	Patients with UC	(♀♂)	30	30	48	35.6 ± 11.4	33.9 ± 11.7	NR	NR	5	PL (saccharine)
Chojnacki et al. 2015 [92]	Poland	P, R, SB, PC	Postmenopausal women	♀	34	30	24	57.9 ± 5.5	56.1 ± 5.8	30.9 ± 3.1	30.1 ± 3.5	5	PL
Chojnacki et al. 2018 [91]	Poland	P, R, DB, PC	Postmenopausal women	♀	30	30	48	57.3 ± 6.4	56.2 ± 4.1	30.9 ± 3.5	30.7 ± 3.8	8	PL
D’Anna et al. 2017 [94]	Italy	P, R, PC	Women during menopausal transition	♀	16	16	24	49.1 ± 1.7	48.7 ± 1.5	26.7 ± 4.1	25.3 ± 3.7	3	PL (myoinositol)
Esalatmanesh et al. 2021 [95]	Iran	P, R, DB, PC	Patients with RA	(♀♂)	32	32	12	49.3 ± 10.8	49.4 ± 12.7	27.2 ± 5.3	28.4 ± 5.6	6	PL
Farrokhian et al. 2019 [96]	Iran	P, R, TB, PC	Patients with T2DM	(♀♂)	34	36	8	57.7 ± 8.5	57.6 ± 9.1	29.3 ± 4.5	27.6± 5.0	6	PL (cellulose)
Forrest et al. 2007 [97]	UK	P, R, DB, PC	Patients with RA	(♀♂)	37	38	24	65.1± 2.1	60.0 ± 1.8	NR	NR	10	PL
Ganjifard et al. 2025 [98]	Iran	P, R, DB, PC	Patients with COVID-19	(♀♂)	23	23	2	62.5 ± 18.6	52.8 ± 16.1	NR	NR	18	PL (cellulose)
Ghaderi-Zefrehi et al. 2024 [99]	Iran	P, R, DB, PC	Patients with MetS	(♀♂)	31	32	12	>18	>18	30.2 ± 4.0	32.0 ± 5.0	6	PL (starch)
Hoseini et al. 2021 [105]	Iran	P, R, DB, PC	Patients with HFrEF	(♀♂)	42	43	24	62.7 ± 10.3	59.1 ± 11.5	26.7 ± 3.2	27.2 ± 4.3	10	PL
Gonciarz et al. 2012 [100]	Poland	P, R, DB, PC	Patients with NASH	(♀♂)	30	12	24	41.5 ± 4	40.8 ± 3.6	NR	NR	10	PL
Goyal et al. 2014 [101]	USA	P, R, DB, PC	Patients with MetS	(♀♂)	19	20	10	62.7 ± 9.6	57.6 ± 10.1	35.2 ± 7.0	34.1 ± 6.4	8	PL
Grossman et al. 2006 [102]	Israel	P, R, DB, PC	Patients with nocturnal HTN	(♀♂)	19	19	4	62 ± 11	66± 11	27.4 ±4.5	27.3 ± 2.9	2	PL
Hannemann et al. 2024 [103]	Germany	P, R, DB, PC	Night-shift workers	(♀♂)	12	12	12	38.3 ± 11.6	34.8 ± 11.5	26.1 ± 5.1	27.8± 7.7	2	PL
Hasan et al. 2022 [104]	Iraq	P, R, CO	Patients with COVID-19	(♀♂)	82	76	2	56.8 ± 7.5	55.7 ± 8.0	NR	NR	10	NI
Jallouli et al. 2025 [106]	Tunisia	P, R, DB, PC	Patients with MS	(♀♂)	15	12	12	34.6 ± 10.9	36.8 ± 8.0	23.9 ± 4.2	22.9 ± 4.3	3	PL (starch & cellulose)
Javanmard et al. 2016 [107]	Iran	P, R, DB, PC	Patients underwent CABG	(♀♂)	20	19	4	60.1 ± 6.3	58.6 ± 5.8	27.7 ± 3.2	29.2 ± 3.7	10	PL
Zare Javid et al. 2020 [133]	Iran	P, R, DB, PC	Patients with T2DM & PD	(♀♂)	22	22	8	53.7 ± 6.6	51.4 ± 5.0	27.3 ± 2.1	27.2 ± 2.1	6	PL (excipients)
Kari et al. 2019 [108]	Iran	P, R, PC	Postmenopausal women with T2DM	♀	10	8	8	50–60	50–60	28.3 ± 4.1	31.4 ± 4.0	3	PL (MD)
Kim et al. 2021 [109]	South Korea	P, R, DB, PC	Women >55 y with insomnia	♀	19	19	6	61 ± 9.6	61 ± 4.4	24.9 ± 3.2	23.6 ± 4.4	2	PL
Kotlarczyk et al. 2012 [110]	USA	P, R, DB, PC	Perimenopausal women	♀	13	5	24	50.3 ± 3.0	47.5 ± 2.0	25.7 ± 3.7	21.7 ± 3.5	3	PL (lactose)
Larki et al. 2025 [136]	Iran	P, R, DB, PC	Hemodialysis patients	(♀♂)	6	41	41	48.9 ± 9.7	50.0 ± 12.6	27.7 ± 5.4	27.3 ± 4.5	3	PL
Lauritzen et al. 2022 [111]	Denmark	C, R, DB, PC	Patients with T2DM	♂	65	65	12	65 ± 21.5	65 ± 21.5	29 ± 3.5	29 ± 3.5	10	PL
Leonardo-Mendonça et al. 2017 [112]	Spain	P, R, DB, PC	Resistance-trained athletes	♂	12	12	4	19–30	19–30	NR	NR	100	PL (lactose & colloidal silica)
Marzougui et al. 2024 [114]	Tunisia	P, R, DB, PC	Hemodialysis patients	(♀♂)	11	11	12	49.2 ± 10.2	49 ± 12.5	22.3 ± 2.7	23.2 ± 3.9	3	PL
Modabbernia et al. 2014 [115]	Iran	P, R, DB, PC	Patients with schizophrenia	(♀♂)	18	18	8	32.7 ± 7.3	32.8 ± 8.2	23.9 ± 3.7	23.2 ± 3.2	3	PL
Mohammadi et al. 2021 [116]	Iran	P, R, DB, PC	Individuals with OW or OB	(♀♂)	19	19	12	38.9 ± 11.6	37.8 ± 11.3	31.0 ± 2.0	30.4 ± 1.6	3	PL
Mohammadi et al. 2025 (a) [137]	Iran	P, R, DB, PC	Patients underwent CABG surgery	(♀♂)	17	18	8	64.4 ± 7.9	60.2 ± 7.3	26.7 ± 4.7	26.8 ± 3.6	5	PL (cellulose)
Mohammadi et al. 2025 (b) [137]	Iran	P, R, DB, PC	Patients underwent CABG surgery	(♀♂)	17	18	8	61.7 ± 8.9	60.2 ± 7.3	28.3 ± 5.1	26.8 ± 3.6	10	PL (cellulose)
Luz et al. 2025 (a) [113]	Brazil	P, R, DB, PC	Morning-shift workers	♀	7	9	12	49.9 ± 6.6	45.1 ± 4.3	26.0 ± 3.3	28.2 ± 3.8	0.3	PL
Luz et al. 2025 (b) [113]	Brazil	P, R, DB, PC	Afternoon-shift workers	♀	8	7	12	47.1 ± 5.7	48.5 ± 5.8	28.3 ± 6.6	31.5 ± 4.7	0.3	PL
Luz et al. 2025 (c) [113]	Brazil	P, R, DB, PC	Night-shift workers	♀	7	8	12	43.0 ± 3.5	50.0 ± 4.9	27.8 ± 4.5	27.1 ± 5.5	0.3	PL
Mousavi et al. 2022 [117]	Iran	P, R, DB, PC	Women with PCOS	♀	21	20	8	25.5 ± 4.9	26.2± 5.7	28.4 ± 3.8	26.9 ± 3.8	6	PL (MG)
Nabatian-Asl et al. 2021 [118]	Iran	P, R, DB, PC	Patients with SLE	♀	13	12	12	40.6 ± 12.9	39.1 ± 9.0	26.0 ± 5.6	27.5 ± 3.8	10	PL
Nunes et al. 2008 [119]	Brazil	P, R, DB, PC	Patients with COPD	(♀♂)	12	13	3	64.1 ± 9.9	67.3 ± 8.1	23.8 ± 4.2	24.1 ± 4.0	3	PL
Ortiz-Franco et al. 2017 [120]	Spain	P, R, DB, PC	Diabetic hemodialysis patients	♂	7	7	2	26.0 ±6.0	28.4 ± 4.3	25.0 ± 2.2	24.7 ± 1.9	20	PL (lactose)
Pakravan et al. 2017 [121]	Iran	P, R, DB, PC	Patients with NAFLD	(♀♂)	49	48	6	42.5 ± 10.1	40.6 ± 9.8	NR	NR	6	PL
Panah et al. 2019 [122]	Iran	P, R, DB, PC	RT patients with IRI	(♀♂)	20	20	4	39.2 ± 7.4	36.8 ± 8.5	NR	NR	3	PL
Rechciński et al. 2010 [123]	Poland	P, R, PC	Patients with CAD	(♀♂)	40	20	13	61.1 ± 6.7	53.6 ± 13.6	NR	NR	5	PL
Rezvanfar et al. 2017 [124]	Iran	C, R, DB, PC	Patients with T2DM	(♀♂)	64	76	12	52 ± 8	52 ± 8	NR	NR	6	PL
Rigamonti et al. 2024 [125]	Italy	P, R, DB, PC	Adults with OB underwent a BWR	(♀♂)	9	9	2	27.8 ± 5.6	28.8 ± 5	43 ± 4.9	42.8 ± 4	2	PL
Rindone et al. 1997 (a) [126]	USA	C, R, SB, PC	Patients with HC	(♀♂)	8	16	6	68 ± 9	68 ± 9	NR	NR	0.3	PL
Rindone et al. 1997 (b) [126]	USA	C, R, SB, PC	Patients with HC	(♀♂)	8	16	6	68 ± 9	68 ± 9	NR	NR	3	PL
Rondanelli et al. 2018 [127]	Italy	P, R, DB, PC	Sarcopenic elderly patients	(♀♂)	42	44	4	81.6 ± 7.0	81.8± 6.4	24.0 ± 0.8	22.8 ± 0.6	1	PL (MD)
Romo-Nava et al. 2014 (a) [134]	Mexico	P, R, DB, PC	SGA-treated patients (medium risk)	(♀♂)	15	13	8	30.6 ± 7.5	28.6 ± 9	26.1 ± 4.2	26.7 ± 5.4	5	PL
Romo-Nava et al. 2014(b) [134]	Mexico	P, R, DB, PC	SGA-treated patients (high risk)	(♀♂)	5	11	8	30.6 ± 7.5	28.6 ± 9	26.2 ± 5.3	24.6 ± 6.1	5	PL
Sadeghi et al. 2025 [128]	Iran	P, R, DB, PC	Patients with CKD	(♀♂)	20	21	10	64 ± 12	65 ± 12	29.2 ± 3.5	29.9 ± 4.5	10	PL (starch)
Sánchez-López et al. 2018 [129]	Mexico	P, R, DB, PC	Patients with MS	(♀♂)	17	16	24	26–52	29–51	23.8± 3.2	24.1 ± 3.3	25	PL
Seabra et al. 2000 [130]	Brazil	P, R, DB, PC	Healthy men	♂	30	10	4	29 ± 6.3	29 ± 6.3	NR	NR	10	PL
Szewczyk-Golec et al. 2017 [131]	Poland	P, R, DB, PC	Patients with OB (calorie-restricted)	♀	15	15	4	37.7 ± 13.2	36.3 ± 16.2	37.8 ± 5.8	38.2 ± 7.5	10	PL (lactose)
Talari et al. 2022 [132]	Iran	P, R, DB, PC	Patients with DN	(♀♂)	19	13	24	40–85	40–85	NR	NR	10	PL (starch)

Abbreviations: MetS, metabolic syndrome; P, parallel; C, crossover; BMI, body mass index; CO, controlled; IG, intervention group; PC, placebo-controlled; NR, not reported; ♀, female; ♂, male; PL, placebo; R, randomized; OB, obesity; PCOS, polycystic ovary syndrome; COVID-19, coronavirus disease 2019; SB, single-blinded; T2DM, type 2 diabetes mellitus; PD, periodontal disease; OW, overweight; COPD, chronic obstructive pulmonary disease; UC, ulcerative colitis; RA, rheumatoid arthritis; HFrEF, heart failure with reduced ejection fraction; DB, double-blinded; NASH, nonalcoholic steatohepatitis; CG, control group; HTN, hypertension; MS, multiple sclerosis; CABG, coronary artery bypass grafting; NAFLD, nonalcoholic fatty liver disease; SLE, systemic lupus erythematosus; IRI, ischemia and reperfusion injury; RT, renal transplant patients; CAD, coronary artery disease; BWR, body weight reduction; HC, hypercholesterolemia; CKD, chronic kidney disease; DN, diabetic nephropathy; SGAs, second-generation antipsychotics; USA, United states of America; NI, no intervention; TB, triple blinded; UK, United Kingdom; NSPT, nonsurgical periodontal therapy; MG, magnesium; MD, maltodextrin. Excipients include starch, cellulose, silicon dioxide, etc.

**Table 2:** Summary of the impacts of melatonin supplementation on cardiometabolic risk factors.

CMRFs	Effect Sizes (n)	Participants (n)	WMD (95% CI)	*p*-Value	Heterogeneity		Certainty of the Evidence (GRADE)
					I^2^ (%)	*p*-Value	
**Anthropometric parameters**							
BW (kg)	27	1276	−0.49 (−1.18, 0.20)	0.163	9.2	0.328	Moderate (II)
BMI (kg/m^2^)	27	1062	−0.31 (−0.94, 0.32)	0.338	76.6	<0.001	Moderate (II)
WC (cm)	20	908	−0.92 (−1.93, 0.09)	0.073	47.2	0.011	High (I)
HC (cm)	9	396	−1.18 (−2.28, −0.08)	**0.035**	0	0.657	Moderate (II)
BFP (%)	9	227	0.01(−0.01, 0.03)	0.296	0	0.991	High (I)
**Glycemic parameters**							
FBG (mg/dL)	20	958	−11.63 (−19.16, −4.10)	**0.002**	98	<0.001	Low (III)
FI (µIU/mL)	7	296	0.49 (−1.08, 2.05)	0.544	64.2	0.010	Low (III)
HbA1c (%)	5	402	−0.22 (−0.66, 0.21)	0.313	73.3	0.005	Moderate (II)
HOMA-IR	8	280	0.15 (−0.18, 0.48)	0.359	15.7	0.307	High (I)
**Lipid parameters**							
TG (mg/dL)	21	1047	−6.10 (−14.69,2.49)	0.164	66.7	<0.001	Low (III)
TC (mg/dL)	20	1006	−6.97 (−12.20, −1.74)	**0.009**	73.7	<0.001	Low (III)
LDL-C (mg/dL)	20	943	−6.28 (−10.53, −2.03)	**0.004**	64.7	<0.001	Low (III)
HDL-C (mg/dL)	20	1005	2.04 (0.50, 3.57)	**0.009**	72	<0.001	Low (III)
**Blood pressure**							
SBP (mmHg)	23	1157	−2.34 (−4.13, −0.55)	**0.011**	69.7	<0.001	Low (III)
DBP (mmHg)	21	1069	−0.88 (−2.19, 0.43)	0.186	73.3	<0.001	Low (III)
**Oxidative stress parameters**							
MDA (μmol/L)	16	671	−1.54 (−2.07, −1.01)	**<0.001**	95.5	<0.001	Very low (IV)
TAC (mmol/L)	12	524	0.15 (0.08, 0.22)	**<0.001**	96.2	<0.001	Low (III)
**Inflammatory parameters**							
CRP (mg/L)	21	1108	−0.59 (−0.94, −0.23)	**<0.001**	93.5	<0.001	Moderate (II)
IL−6 (pg/mL)	8	351	− 6.43 (−10.72, −2.15)	**0.003**	98.7	<0.001	Low (III)
TNF-α (pg/mL)	11	488	−1.61 (−2.31, −0.90)	**<0.001**	96.1	<0.001	Low (III)
**Liver function markers**							
AST (IU/L)	8	345	−2.64 (−6.63, 1.35)	0.194	85.5	<0.001	Low (III)
ALT (IU/L)	9	383	−2.61 (−4.87, −0.34)	**0.024**	71.4	<0.001	Low (III)
GGT (IU/L)	5	188	−7.21 (−15.20, 0.79)	0.077	88.7	<0.001	Low (III)

Abbreviations: BMI, body mass index; BFP, body fat percentage; WC, waist circumference; BW, body weight; SBP, systolic blood pressure; DBP, diastolic blood pressure; HC, hip circumference; MDA, malondialdehyde; ALT, alanine aminotransferase; AST, aspartate aminotransferase; TG, triglycerides; TC, total cholesterol, LDL-C, low-density lipoproteins cholesterol; HDL-C, high-density lipoprotein cholesterol; FBG, fasting blood glucose; HbA1c, hemoglobin A1c; HOMA-IR, homeostatic model assessment of insulin resistance; TAC, total antioxidant capacity; CRP, C-reactive protein; GGT, gamma-glutamyl transferase; IL-6, interleukin-6; TNF-α, tumor necrosis factor-alpha; WMD, weighted mean difference; CI, confidence interval; FI, fasting insulin; CMRFs: cardiometabolic risk factors. Bold numbers indicate statistical significance (*p* < 0.05).

## Data Availability

The original contributions presented in this study are included in the article/Supplementary Materials. Further inquiries can be directed to the corresponding authors.

## Supplementary Materials

The following supporting information can be downloaded at https://www.mdpi.com/article/10.3390/nu18010134/s1. Figure S1: Funnel plots for the effects of melatonin supplementation on CMRFs; Figure S2: Nonlinear dose–response association between dose of melatonin supplementation and mean changes in CMRFs; Figure S3: Nonlinear association between duration of melatonin supplementation and mean changes in CMRFs; Figure S4: Linear dose–response association between dose of melatonin supplementation and mean changes in CMRFs; Figure S5: Linear association between duration of melatonin supplementation and mean changes in CMRFs; Table S1: Search strategy in PubMed (MEDLINE); Table S2: Subgroup analyses of the impacts of melatonin supplementation on CMRFs; Table S3: Risk of bias assessment; Table S4: GRADE assessment.

## Abbreviations

The following abbreviations are used in this manuscript:

BMI	Body mass index
WC	Waist circumference
SBP	Systolic blood pressure
DBP	Diastolic blood pressure
HC	Hip circumference
NO	Nitric oxide
MDA	Malondialdehyde
ALT	Alanine aminotransferase
AST	Aspartate aminotransferase
TG	Triglycerides
TC	Total cholesterol
LDL-C	Low-density lipoprotein cholesterol
HDL-C	High-density lipoprotein cholesterol
FBG	Fasting blood glucose
HbA1c	Hemoglobin A1c
HOMA-IR	Homeostatic model assessment of insulin resistance
TAC	Total antioxidant capacity
CRP	C-reactive protein
GGT	Gamma-glutamyl transferase
IL-6	Interleukin-6
TNF-α	Tumor necrosis factor-alpha
WMD	Weighted mean difference
CI	Confidence interval
MetS	Metabolic syndrome
PCOS	Polycystic ovary syndrome
COVID-19	Coronavirus disease 2019
T2DM	Type 2 diabetes mellitus
NAFLD	Nonalcoholic fatty liver disease
COPD	Chronic obstructive pulmonary disease
UC	Ulcerative colitis
RA	Rheumatoid arthritis
HFrEF	Heart failure with reduced ejection fraction
NASH	Nonalcoholic steatohepatitis
HTN	Hypertension
MS	Multiple sclerosis
CABG	Coronary artery bypass grafting
OB	Obesity
SLE	Systemic lupus erythematosus
IRI	Ischemia and reperfusion injury
CAD	Coronary artery disease
DN	Diabetic nephropathy
SGAs	Second-generation antipsychotics
RCT	Randomized controlled trial
OW	Overweight
OS	Oxidative stress
BW	Body weight
BP	Blood pressure
PROSPERO	Prospective Register of Systematic Reviews
PRISMA	Preferred Reporting Items for Systematic Reviews and Meta-analyses
USA	United States of America
GRADE	Grading of recommendations assessment, development, and evaluation
RoB	Risk of bias
ROS	Reactive oxygen species
CMRF	Cardiometabolic risk factor
CVD	Cardiovascular disease
CMH	Cardiometabolic health
CMR	Cardiometabolic risk
CMD	Cardiometabolic disease
FI	Fasting insulin
IR	Insulin resistance
SD	Standard deviation
UK	United Kingdom
