# Supplementary material for: Comprehensive Effects of Melatonin Supplementation on Cardiometabolic Risk Factors: A Systematic Review and Dose–Response Meta-Analysis
Source: Nutrients. 2025 Dec 31;18(1):134. doi: 10.3390/nu18010134 (PMC12787795; doi:10.3390/nu18010134)
Supplement: Supplementary file 1 [file nutrients-18-00134-s001.zip › nutrients-4006151-supplementary.pdf]

Supplementary Materials

Table S1. Search strategy in PubMed (MEDLINE)

	Keywords	Search strategy
#1	**Melatonin**	(“melatonin” [tiab] OR “N-acetyl-5-methoxytryptamine” [tiab] OR “Pineal hormone” [tiab])
#2	**Cardiometabolic risk factors**	(“body weight”[tiab] OR “waist circumference”[tiab] OR “WC”[tiab] OR “hip circumference”[tiab] OR “HC”[tiab] OR “body mass index”[tiab] OR “BMI”[tiab] OR “body fat percentage”[tiab] OR “BFP”[tiab] OR “gamma-glutamyl transferase”[tiab] OR “GGT”[tiab] OR “aspartate transaminase”[tiab] OR “aspartate aminotransferase”[tiab] OR “AST”[tiab] OR “alanine aminotransferase”[tiab] OR “ALT”[tiab] OR “fasting Insulin”[tiab] OR “homeostatic model assessment for Insulin Resistance”[tiab] OR “HOMA-IR”[tiab] OR “hemoglobin A1C”[tiab] OR “HbA1c”[tiab] OR “fasting blood sugar”[tiab] OR “FBS”[tiab] OR “fasting blood glucose”[tiab] OR “FBG”[tiab] OR “triglycerides”[tiab] OR “TG”[tiab] OR “total cholesterol”[tiab] OR “TC”[tiab] OR “low-density lipoprotein”[tiab] OR “LDL”[tiab] OR “high-density lipoprotein”[tiab] OR “HDL”[tiab] OR “total antioxidant capacity”[tiab] OR “TAC”[tiab] OR “malondialdehyde”[tiab] OR “MDA”[tiab] OR “C-reactive protein”[tiab] OR “CRP”[tiab] OR “tumor necrosis factor”[tiab] OR “TNF-alpha”[tiab] OR “Interleukin-6”[tiab] OR “IL-6”[tiab] OR “diastolic blood pressure”[tiab] OR “DBP”[tiab] OR “systolic blood pressure”[tiab] OR “SBP”[tiab])
#3	**Study design**	("Intervention"[tiab] OR "Intervention Study"[tiab] OR "Intervention Studies"[tiab] OR "controlled trial"[tiab] OR "randomized"[tiab] OR "randomised"[tiab] OR "random"[tiab] OR "randomly"[tiab] OR "placebo"[tiab] OR "clinical trial"[tiab] OR "randomized controlled trial"[tiab] OR "randomized clinical trial"[tiab] OR "RCT"[tiab] OR "blinded"[tiab] OR "double blind"[tiab] OR "double blinded"[tiab] OR "trial"[tiab] OR "trials"[tiab] OR "Pragmatic Clinical Trial"[tiab] OR "Cross-Over Studies"[tiab] OR "Cross-Over"[tiab] OR "Cross-Over Study"[tiab] OR "parallel"[tiab] OR "parallel study"[tiab] OR "parallel trial"[tiab])
#4	**Final search combination**	#1 AND #2 AND #3

Table S2. Subgroup analyses of the impacts of melatonin supplementation on cardiometabolic risk factors (CMRFs)

Sub-groups	Effect Sizes (n)	WMD (95% CI)	P-value	Heterogeneity		
				P-value	I^2 (%)	P-value between sub-groups
**Impacts of melatonin on BW** (kg)						
Overall effect	27	-0.49 (-1.18,0.20)	0.163	0.328	9.2	
Trial duration (weeks)						
≤12	27	-0.49 (-1.18,0.20)	0.163	0.328	9.2	
Melatonin dose (mg/day)						
≤6	23	-0.80 (-1.68,0.07)	0.071	0.190	20.3	0.613
>6	4	-0.19 (-2.39,2.00)	0.863	0.839	0	
Baseline BMI						
Normal	4	-0.55 (-2.30,1.19)	0.534	0.055	60.6	
OW	15	-1.34 (-2.51,-0.17)	**0.024**	0.786	0	0.715
OB	8	-0.70 (-2.58,1.17)	0.461	0.981	0	
Sex						
Female	10	-1.46 (-3.01,0.09)	0.065	0.909	0	
Male	2	0.11 (-2.20,2.43)	0.922	0.894	0	0.521
Both	15	-0.74 (-1.84,0.36)	0.188	0.152	27.6	
**Impacts of melatonin on BMI** (kg/m^2)						
Overall effect	27	-0.31 (-0.94,0.32)	0.338	<0.001	76.6	
Trial duration (weeks)						
≤12	24	0.07 (-0.38,0.53)	0.747	0.011	44.2	0.007
>12	3	-2.42 (-4.17,-0.66)	**0.007**	0.003	83.2	
Melatonin dose (mg/day)						
≤6	23	-0.29 (-0.99,0.40)	0.414	<0.001	80	0.935
>6	4	-0.23 (-1.39,0.92)	0.692	0.792	0	
Baseline BMI						
Normal	4	0.57 (-0.45,1.59)	0.274	0.207	34.1	
OW	12	-0.00 (-0.82, 0.82)	0.995	0.001	65.1	0.174
OB	11	-0.86 (-1.96, 0.24)	0.126	<0.001	82.1	
Sex						
Female	10	-1.46 (-3.01,0.09)	0.065	0.909	0	
Male	2	0.11 (-2.20, 2.43)	0.922	0.894	0	0.521
Both	15	-0.74 (-1.84, 0.36)	0.188	0.152	27.6	
**Impacts of melatonin on WC** (cm)						
Overall effect	20	-0.92 (-1.93,0.09)	0.073	0.011	47.2	
Trial duration (weeks)						
≤12	19	-0.74 (-1.71, 0.22)	0.132	0.029	41.9	0.050
>12	1	-5.60 (-10.36,-0.83)	**0.021**	-	-	
Melatonin dose (mg/day)						
≤6	16	-0.70 (-1.74, 0.34)	0.190	0.034	43.2	0.084
>6	4	-2.47 (-4.20,-0.75)	**0.005**	0.562	0	
Baseline BMI						
Normal	2	-0.48 (-4.42,3.46)	0.811	0.028	79.2	
OW	9	-1.81 (-2.88, -0.74)	**0.001**	0.576	0	0.272
OB	9	-0.41 (-1.79, 0.97)	0.558	0.333	12.2	
Sex						
Female	7	-0.44 (-2.50,1.61)	0.673	0.093	44.7	0.563
Both	13	-1.15 (-2.37, 0.07)	0.066	0.014	52.3	
**Impacts of melatonin on HC (cm)**						
Overall effect	9	-1.18 (-2.28,-0.08)	**0.035**	0.657	0	
Trial duration (weeks)						
≤12	9	-1.18 (-2.28, -0.08)	**0.035**	0.657	0	
Melatonin dose (mg/day)						
≤6	9	-1.18 (-2.28, -0.08)	**0.035**	0.657	0	
Baseline BMI						
Normal	1	-2.30 (-5.44, 0.84)	0.152	-	-	
OW	4	-2.64 (-4.58,-0.70)	**0.007**	0.795	0	0.092
OB	4	-0.09 (-1.56, 1.38)	0.903	0.990	0	
Sex						
Female	2	-2.00 (-4.75, 0.75)	0.154	0.242	27.0	0.496
Both	7	-0.95 (-2.19,0.29)	0.133	0.684	0	
**Impacts of melatonin on BFP (%)**						
Overall effect	9	0.01 (-0.01,0.03)	0.296	0.991	0	
Trial duration (weeks)						
≤12	9	0.01 (-0.01,0.03)	0.296	0.991	0	
Melatonin dose (mg/day)						
≤6	9	0.01 (-0.01,0.03)	0.296	0.991	0	

Table S2. *Cont.*

Sub-groups	Effect Sizes (n)	WMD (95% CI)	P-value	Heterogeneity		
				P-value	I² (%)	P-value between Sub-groups
Baseline BMI						
OW	6	0.24 (-1.73, 2.22)	0.806	0.915	0	0.814
OB	3	0.01 (-0.00, 0.02)	0.297	0.966	0	
Sex						
Female	4	0.59 (-1.68, 2.87)	0.607	0.775	0	0.613
Both	5	0.01 (-0.00, 0.02)	0.298	0.993	0	
**Impacts of melatonin on serum FBG** (mg/dL)						
Overall effect	20	-11.63 (-19.16,-4.10)	**0.002**	<0.001	98	
Baseline serum FBG						
≤100	10	-2.36 (-4.12, -0.59)	**0.009**	0.024	53.1	0.277
>100	10	-19.96 (-51.61,11.69)	0.217	<0.001	98.9	
Trial duration (weeks)						
≤12	19	-11.28 (-19.01, -3.54)	**0.004**	<0.001	98.1	0.344
>12	1	-18.52 (-31.37, -5.66)	**0.005**	-	-	
Melatonin dose (mg/day)						
≤6	15	-14.14 (-26.08,-2.21)	**0.020**	<0.001	98.5	0.069
>6	5	-2.36 (-6.69,1.96)	0.284	0.083	51.4	
Baseline BMI						
Normal	5	-32.87 (-54.52,-11.23)	**0.003**	<0.001	99.6	
OW	10	-2.52 (-5.68, 0.64)	0.119	0.130	34.8	0.015
OB	5	-0.48 (-4.89,3.93)	0.831	0.107	47.4	
Sex						
Female	5	-2.41 (-4.95,0.11)	0.061	0.593	0	
Male	2	-1.63 (-2.64,-0.62)	**0.001**	0.924	0	0.158
Both	13	-15.24 (-29.62, -0.86)	**0.038**	<0.001	98.7	
**Impacts of melatonin on FI** (μIU/mL)						
Overall effect	7	0.49 (-1.08,2.05)	0.544	0.010	64.2	
Trial duration (weeks)						
≤12	6	0.51 (-1.22,2.26)	0.562	0.006	69.1	0.927
>12	1	0.70 (-2.80,4.20)	0.695	-	-	
Melatonin dose (mg/day)						
≤6	6	1.23 (-0.49, 2.95)	0.161	0.157	37.4	0.004
>6	1	-1.69 (-2.66,-0.71)	**0.001**	-	-	
Baseline BMI						
Normal	1	4.60 (-1.04, 10.24)	0.110	-	-	
OW	4	0.77 (-1.15, 2.70)	0.430	0.170	40.3	0.323
OB	2	-0.43 (-3.79, 2.92)	0.800	0085	66.2	
Sex						
Female	3	-1.50 (-2.43,-0.57)	**0.002**	0.412	0	0.016
Both	4	1.80 (-0.72,4.32)	0.163	0.047	62.2	
**Impacts of melatonin on HbA1c (%)**						
Overall effect	5	-0.22 (-0.66,0.21)	0.313	0.005	73.3	
Trial duration (weeks)						
≤12	5	-0.22 (-0.65,0.21)	0.313	0.005	73.3	
Melatonin dose (mg/day)						
≤6	4	0.00 (-0.78,0.78)	0.303	0.002	80.0	0.573
>6	1	-0.27 (-0.78, 0.24)	1.000	-	-	
Baseline BMI						
Normal	1	-0.19 (-1.63,1.25)	0.796	-	-	
OW	3	-0.44 (-0.81,-0.06)	**0.021**	0.205	36.8	0.018
OB	1	0.20 (-0.04, 0.44)	0.104	-	-	
Sex						
Female	1	-0.78 (-1.27,-0.28)	**0.002**	-	-	
Male	1	0.00 (-0.78,0.78)	1.000	-	-	0.082
Both	3	-0.06 (-0.53, 0.40)	0.773	0.044	68.0	
**Impacts of melatonin on HOMA-IR**						
Overall effect	8	0.15 (-0.18,0.48)	0.359	0.307	15.7	
Trial duration (weeks)						
≤12	8	0.15 (-0.18,0.48)	0.359	0.307	15.7	
Melatonin dose (mg/day)						
≤6	7	0.22 (-0.15, 0.60)	0.242	0.230	26.1	0.508
>6	1	-0.27 (-1.68,1.14)	0.709	-	-	
Baseline BMI						
Normal	2	0.08 (-0.84,1.01)	0.862	0.165	48.0	
OW	4	0.44 (-0.08,0.97)	0.097	0.871	0.0	0.800
OB	2	0.35 (-0.41,1.12)	0.365	0.305	5.1	
Sex						
Female	4	-0.17 (-0.47,0.11)	0.232	0.915	0	0.007
Both	4	0.57 (0.11, 1.04)	**0.015**	0.914	0	

Table S2. *Cont.*

**Sub-groups**	**Effect Sizes** (n)	**WMD** (95% CI)	**P-value**	**Heterogeneity**		
				**P-value**	**I²** (%)	**P-value between Sub-groups**
**Impacts of melatonin on serum TG** (mg/dL)						
Overall effect	21	-6.10 (-14.69,2.49)	0.164	<0.001	66.7	
Baseline TG						
≤150	6	-0.40 (-12.94,11.67)	0.947	0.005	69.9	0.256
>150	15	-10.63 (-23.50,2.40)	0.105	<0.001	67.7	
Trial duration (weeks)						
≤12	19	-2.88 (-10.65,4.88)	0.467	0.001	57.3	<0.001
>12	2	-50.13 (71.17,-29.08)	**<0.001**	0.510	0.0	
Melatonin dose (mg/day)						
≤6	14	-0.28 (-9.36,8.81)	0.950	0.014	51.3	0.079
>6	7	-19.30 (-38.42,-0.17)	**0.048**	<0.001	77.2	
Baseline BMI						
Normal	5	-18.88 (-34.22,-3.55)	**0.016**	**<0.001**	82.9	
OW	11	3.53(-7.37,14.43)	0.526	**0.038**	47.9	0.058
OB	5	-10.06 (-32.20,12.08)	0.373	0.144	41.7	
Sex						
Female	3	-4.61 (-24.50,15.27)	0.649	0.108	55.1	
Male	3	-5.18 (-26.11,15.74)	0.628	0.007	80.1	0.947
Both	15	-7.10 (-19.50,5.29)	0.262	<0.001	69.0	
**Impacts of melatonin on serum TC** (mg/dL)						
Overall effect	20	-6.97 (-12.20,-1.74)	**0.009**	<0.001	73.7	
Baseline TC						
≤200	13	-4.07 (-9.53,1.39)	0.144	<0.001	68.5	0.175
>200	7	-13.59 (-26.20,-0.97)	**0.035**	<0.001	78.8	
Trial duration (weeks)						
≤12	18	-4.75 (-9.40,-0.09)	**0.045**	<0.001	65.5	0.098
>12	2	-35.75 (-72.22, 0.71)	0.055	0.016	82.9	
Melatonin dose (mg/day)						
≤6	15	-4.53 (-9.94, 0.86)	0.100	0.002	58.6	0.135
>6	5	-15.68 (-29.27, -2.10)	**0.024**	<0.001	88.8	
Baseline BMI						
Normal	6	-16.63 (-27.12,-6.13)	**0.002**	<0.001	84.4	
OW	10	1.95 (-1.83, 5.74)	0.311	0.483	0	<0.001
OB	4	-12.26 (-19-87,-4.65)	**0.002**	0.505	0	
Sex						
Female	4	-2.36 (-11.55, 6.81)	0.614	0.185	37.8	
Male	2	-3.67 (-18.04,10.69)	0.617	<0.001	92.9	0.519
Both	14	-8.97 (-16.44, -1.51)	**0.018**	<0.001	74.2	
**Impacts of melatonin on serum LDL-C** (mg/dL)						
Overall effect	20	-6.28 (-10.53,-2.03)	**0.004**	<0.001	64.7	
Baseline LDL-C						
≤100	5	2.04 (-3.11,7.20)	0.437	0.525	0	0.002
>100	15	-8.91 (-13.58,-4.24)	**<0.001**	0.001	62.8	
Trial duration (weeks)						
≤12	19	-5.10 (-8.22,-1.98)	**0.001**	0.068	34.9	<0.001
>12	1	-60.00 (-80.64,-39.35)	**<0.001**	-	-	
Melatonin dose (mg/day)						
≤6	14	-5.85 (-9.38,-2.32)	**0.001**	0.133	30.5	0.468
>6	6	-10.88 (-24.03,2.52)	0.104	<0.001	85.5	
Baseline BMI						
Normal	5	-15.80 (-28.09,-3.51)	**0.012**	<0.001	83.1	
OW	10	-1.94 (-7.05,3.16)	0.456	0.012	57.4	0.055
OB	5	-9.04 (-15.18,-2.91)	**0.004**	0.815	0	
Sex						
Female	4	-1.40 (-7.88,5.07)	0.672	0.298	18.6	
Male	3	-0.08 (-10.94,10.32)	0.987	0.030	71.5	0.123
Both	13	-9.16 (-14.74,-3.59)	**0.001**	0.001	65.0	

Table S2. *Cont.*

Sub-groups	Effect sizes (n)	WMD (95% CI)	P-value	Heterogeneity		
				P-value	I^2 (%)	P-value between Sub-groups
**Impacts of melatonin on serum HDL-C** (mg/dL)						
Overall effect	20	2.04 (0.50,3.57)	**0.009**	<0.001	72	
Baseline HDL-C						
≤50	19	2.25 (0.65,3.84)	**0.006**	<0.001	67.5	0.009
>50	1	-0.60 (-2.02,0.82)	0.408	-	-	
Trial duration (weeks)						
≤12	19	2.25 (0.74,3.75)	**0.003**	<0.001	71.4	0.020
>12	1	-10.00 (-20.17,0.17)	0.054	-	-	
Melatonin dose (mg/day)						
≤6	14	2.42 (0.53,431)	**0.012**	<0.001	72.6	0.394
>6	6	1.01 (-1.63,3.65)	0.454	0.022	62.1	
Baseline BMI						
Normal	5	-0.53 (-3.56,2.49)	0.728	<0.001	81.1	
OW	10	3.70 (1.84,5.63)	**<0.001**	0.043	48.3	0.056
OB	5	1.93 (0.37,3.49)	**0.015**	0.546	0	
Sex						
Female	3	3.46 (0.76,6.16)	**0.012**	0.289	19.5	
Male	3	1.31 (-2.04,4.67)	0.443	0.028	72.0	0.532
Both	14	1.79 (-0.26,3.85)	0.088	<0.001	74.4	
**Impacts of melatonin on SBP** (mmHg)						
Overall effect	23	-2.34(-4.13,-0.55)	**0.011**	<0.001	69.7	
Baseline SBP						
≤130	16	-1.60 (-3.70,0.50)	0.135	<0.001	71.5	0.080
>130	6	-4.58 (-7.17,-1.98)	**0.001**	0.178	34.5	
Trial duration (weeks)						
≤12	19	-2.97 (-4.93,-1.01)	**0.003**	<0.001	71.6	0.007
>12	4	1.73 (-1.09,4.57)	0.229	0.632	0	
Melatonin dose (mg/day)						
≤6	18	-2.86 (-4.83,-0.88)	**0.005**	<0.001	71.3	0.267
>6	5	-0.13 (-4.51,4.23)	0.950	0.031	62.4	
Baseline BMI						
Normal	5	-0.35 (-2.76, 2.05)	0.775	0.145	41.4	
OW	14	-2.04 (-4.65, 0.57)	0.126	<0.001	74.4	0.008
OB	4	-5.69 (-8.12, -3.25)	**<0.001**	0.391	0.1	
Sex						
Female	5	-1.47 (-6.79, 3.84)	0.587	0.002	76.5	
Male	2	-0.99 (-3.63, 1.64)	0.462	0.998	0	0.568
Both	16	-2.81 (-4.98, -0.64)	**0.011**	<0.001	71.7	
**Impacts of melatonin on DBP (mmHg)**						
Overall effect	21	-0.88 (-2.19,0.43)	0.186	<0.001	73.3	
Baseline DBP						
≤80	14	-0.72 (-2.29,0.83)	0.362	<0.001	77.1	0.671
>80	6	-1.42 (-4.20,1.36)	0.317	<0.001	68.0	
Trial duration (weeks)						
≤12	17	-1.02 (-2.55, 0.50)	0.188	<0.001	78.0	0.406
>12	4	0.00 (-1.88,1.90)	0.994	0.783	0	
Melatonin dose (mg/day)						
≤6	16	-1.60 (-3.03, -0.17)	**0.027**	<0.001	73.9	0.030
>6	5	1.72 (-0.91,4.40)	0.199	0.065	54.8	
Baseline BMI						
Normal	4	-0.11 (-1.68,1.45)	0.886	0.195	36.2	
OW	13	-0.41 (-2.44,1.61)	0.688	<0.001	79.2	0.021
OB	3	-3.06 (-4.64,-1.48)	**<0.001**	0.404	0	
Sex						
Female	4	-1.35 (-4.54, 1.83)	0.405	0.072	57	
Male	2	1.58 (-0.12,3.28)	0.070	0.328	0	0.045
Both	15	-1.18 (-2.74,0.37)	0.136	<0.001	75.7	

Table S2. *Cont.*

Sub-groups	Effect sizes (n)	WMD (95% CI)	P-value	Heterogeneity		
				P-value	I^2 (%)	P-value between Sub-groups
**Impacts of melatonin on MDA**(μmol/L)						
Overall effect	16	-1.54 (-2.07,-1.01)	**<0.001**	<0.001	95.5	
Trial duration (weeks)						
≤12	15	-1.54 (-2.07,-1.00)	**<0.001**	<0.001	95.8	0.901
>12	1	-1.82 (-6.19, 2.55)	0.415	-	-	
Melatonin dose (mg/day)						
≤6	11	-1.23 (-1.75,-0.71)	**<0.001**	<0.001	95.9	0.149
>6	5	-4.11 (-7.98, -0.24)	**0.037**	<0.001	93.6	
Baseline BMI						
Normal	4	-1.37 (-2.47,-0.27)	**0.014**	<0.001	93.0	
OW	10	-3.31 (-4.81,-1.81)	**<0.001**	<0.001	96.0	<0.001
OB	2	-0.31 (-0.43,-0.18)	**<0.001**	0.222	32.9	
Sex						
Female	4	-0.56 (-1.46, 0.32)	0.212	<0.001	92.9	
Male	1	-0.89 (-3.02,1.24)	0.414	-	-	0.021
Both	11	-2.23 (-3.04,-1.43)	**<0.001**	<0.001	96.1	
**Impacts of melatonin on TAC** (mmol/L)						
Overall effect	12	0.15 (0.08,0.22)	**<0.001**	<0.001	96.2	
Trial duration (weeks)						
≤12	11	0.16 (0.09, 0.23)	**<0.001**	<0.001	96.5	0.019
>12	1	0.01(-0.09, 0.11)	0.854	-	-	
Melatonin dose (mg/day)						
≤6	6	0.14 (0.02,0.26)	**0.023**	<0.001	93.6	0.871
>6	6	0.15 (0.05,0.26)	**0.003**	<0.001	97.3	
Baseline BMI						
Normal	2	0.07 (-0.07,0.21)	0.339	<0.001	94.2	
OW	9	0.18 (0.08, 0.28)	**<0.001**	<0.001	97.0	0.324
OB	1	0.08 (-0.04,0.20)	0.218	-	-	
Sex						
Female	3	0.16 (0.06,0.26)	**0.001**	0.244	29.2	
Male	2	0.23 (-0.22,0.69)	0.324	<0.001	98.5	0.801
Both	7	0.12 (0.02,0.22)	**0.011**	<0.001	97.1	
**Impacts of melatonin on CRP**(mg/L)						
Overall effect	21	-0.59 (-0.94,-0.23)	**<0.001**	<0.001	93.5	
Trial duration (weeks)						
≤12	18	-0.56 (-0.87, -0.25)	**<0.001**	<0.001	91.7	0.036
>12	3	-22.12 (-42.29, -1.95)	**0.032**	<0.001	98.0	
Melatonin dose (mg/day)						
≤6	13	-0.52 (-0.97,-0.07)	**0.023**	<0.001	94.3	0.275
>6	8	-1.21 (-2.37,-0.05)	**0.039**	<0.001	92.4	
Baseline BMI						
Normal	10	-1.91 (-2.88,-0.94)	**<0.001**	<0.001	96.0	
OW	8	-0.33 (-0.61,-0.05)	**0.017**	0.003	68.2	0.008
OB	3	-0.31 (-1.08,0.45)	0.418	<0.001	89.4	
Sex						
Female	4	-0.07 (-0.23,0.09)	0.382	0.295	19.0	
Male	1	-0.86 (-1.63,-0.08)	**0.029**	-	-	0.003
Both	16	-0.83 (-1.33,-0.34)	**0.001**	<0.001	94.5	
**Impacts of melatonin on IL-6** (pg/mL)						
Overall effect	8	-6.43 (-10.72,-2.15)	**0.003**	<0.001	98.7	
Trial duration (weeks)						
≤12	5	-3.92 (-8.89, 1.05)	0.123	<0.001	99.2	0.183
>12	3	-17.83 (-37.71, 2.04)	0.079	<0.001	90.5	
Melatonin dose (mg/day)						
≤6	4	-3.67 (-9.61,2.26)	0.225	<0.001	99.4	0.161
>6	4	-9.45 (-14.95,-3.95)	**<0.001**	<0.001	86.1	
Baseline BMI						
Normal	4	-5.88 (-12.70,0.94)	0.091	<0.001	99.4	
OW	2	-16.37 (-39.98, 7.23)	0.174	<0.001	94.9	0.532
OB	2	-2.25 (-11.83,7.31)	0.644	<0.001	97.3	
Sex						
Female	1	-7.16 (-9.51,-4.80)	**<0.001**	-	-	0.764
Both	7	-6.35 (-11.06,-1.64)	**0.008**	<0.001	98.9	

Table S2. *Cont.*

Sub-groups	Effect sizes (n)	**WMD** (95% CI)	P-value	Heterogeneity		
				**P-value**	I^2 (%)	**P-value between Sub-groups**
**Impacts of melatonin on TNF-α** (pg/mL)						
Overall effect	11	-1.61 (-2.31,-0.90)	**<0.001**	<0.001	96.1	
Trial duration (weeks)						
≤12	8	-0.87 (-1.66, -0.09)	**0.029**	<0.001	94	0.490
>12	3	-2.88 (-8.53,2.76)	0.317	<0.001	98.5	
Melatonin dose (mg/day)						
≤6	7	-0.66 (-1.37, 0.03)	0.063	<0.001	93.3	0.040
>6	4	-6.63 (-12.28,-0.99)	**0.021**	<0.001	98.1	
Baseline BMI						
Normal	5	-1.90 (-2.78,-1.02)	**<0.001**	<0.001	97.1	
OW	4	-12.58 (-22.58,-2.58)	**0.014**	<0.001	93.7	0.015
OB	2	-0.21 (-1.71,1.28)	0.778	<0.001	97.8	
Sex						
Female	2	-1.28 (-2.58,0.27)	0.055	0.224	32.3	0.557
Both	9	-1.75 (-2.61,-0.88)	**<0.001**	<0.001	96.8	
**Impacts of melatonin on AST** (IU/L)						
Overall effect	8	-2.64 (-6.63,1.35)	0.194	<0.001	85.5	
Trial duration (weeks)						
≤12	6	-1.95 (-5.00,1.08)	0.207	0.003	72.3	0.813
>12	2	-5.47(-34.38,23.42)	0.710	<0.001	96.5	
Melatonin dose (mg/day)						
≤6	3	-3.69 (-6.04, -1.33)	**0.002**	0.519	0	0.721
>6	5	-2.49 (-8.60,3.60)	0.423	<0.001	88.7	
Baseline BMI						
Normal	5	-0.77 (-5.61,4.07)	0.755	<0.001	86.0	0.299
OW	3	-7.13 (-18.11, 3.85)	0.203	<0.001	89.2	
Sex						
Male	3	-0.71 (-4.36,2.93)	0.701	0.006	80.2	0.287
Both	5	-6.02 (-15.07,3.03)	0.192	<0.001	89.1	
**Impacts of melatonin on ALT** (IU/L)						
Overall effect	9	-2.61 (-4.87,-0.34)	**0.024**	<0.001	71.4	
Trial duration (weeks)						
≤12	7	-2.45 (-4.85, -0.66)	**0.044**	0.001	78.3	0.485
>12	2	-6.39 (-17.16, 4.37)	0.245	0.845	0	
Melatonin dose (mg/day)						
≤6	5	-2.83 (-5.28,-0.39)	**0.023**	0.046	62.5	0.901
>6	4	-3.15 (-7.65,1.33)	0.168	0.095	49.5	
Baseline BMI						
Normal	6	-3.08 (-5.69,-0.47)	**0.020**	<0.001	80.8	0.297
OW	3	-0.30 (-4.82, 4.12)	0.895	0.926	0	
Sex						
Female	1	-1.00 (-4.11,2.11)	0.530	-	-	
Male	3	-2.23 (-5.76,1.29)	0.214	<0.001	89.8	0.504
Both	5	-4.39 (-9.16,0.38)	0.071	0.201	33.1	
**Impacts of melatonin on GGT (IU/L)**						
Overall effect	5	-7.21 (-15.20,0.79)	0.077	<0.001	88.7	
Trial duration (weeks)						
≤12	3	-0.69 (-3.16, 1.77)	0.581	0.260	25.7	< 0.001
>12	2	-36.21(-48.14,-24.28)	**<0.001**	0.868	0	
Melatonin dose (mg/day)						
≤6	2	1.02 (-1.97,4.02)	0.504	0.968	0	0.128
>6	3	-22.29 (-52.13,7.54)	0.143	<0.001	93.3	
Baseline BMI						
Normal	3	-10.30 (-27.69,7.08)	0.245	<0.001	94	0.993
OW	2	-10.47 (-41.42,20.48)	0.507	0.189	42	
Sex						
Female	1	1.00 (-2.23, 4.23)	0.545	-	-	
Male	1	-2.30 (-4.90, 0.30)	0.083	-	-	0.138
Both	3	-21.42 (-53.69,10.84)	0.193	<0.001	92.3	

Abbreviations**:** BMI, body mass index; BFP, body fat percentage; WC, waist circumference; SBP, systolic blood pressure; DBP, diastolic blood pressure; HC, hip circumference; BW, body weight; MDA, malondialdehyde; ALT, alanine aminotransferase; AST, aspartate aminotransferase; TG, triglycerides; TC, total cholesterol, LDL-C, low-density lipoproteins cholesterol; HDL-C, high-density lipoprotein cholesterol; FBG, fasting blood glucose; HbA1c, hemoglobin A1c; HOMA-IR, homeostatic model assessment of insulin resistance; TAC, total antioxidant capacity; CRP, C-reactive protein; GGT, gamma-glutamyl transferase; IL-6, interleukin-6; TNF-α, tumor necrosis factor alpha; WMD, weighted mean difference; CI, confidence interval; OW, overweight; OB, obesity; FI, fasting insulin. Bold numbers indicate statistical significance ($p < 0.05$).

Table S3. Risk of bias assessment

**Reference**	**Bias arising from the randomization process** (Allocation bias)	**Bias due to deviations from the intended interventions** (Performance bias)	**Bias due to missing outcome data** (Attrition bias)	**Bias in the measurement of the Outcome** (Detection bias)	**Bias in the selection of reported results** (Reporting bias)	**The overall risk of bias**
Abood et al. 2020	U	L	L	L	L	U
Agahi et al.2018	L	L	L	L	L	L
Akhondzadeh et al.2022	L	L	L	L	L	L
Al Lami, 2018	U	U	L	L	U	H
Alamdari et al.2015	L	L	L	L	L	L
Alizadeh et al.2021	L	L	L	L	L	L
Alizadeh et al.2021	U	U	L	L	U	H
Amstrup et al.2024	L	L	L	L	L	L
Anton et al.2022	L	L	L	L	L	L
Azizi et al.2025	L	L	L	L	L	L
Bahrami et al.2019	L	L	L	L	L	L
Bahrami et al.2020	L	L	L	L	L	L
Basu et al.2025	U	U	L	L	U	H
Bazyar et al.2019	L	L	L	L	L	L
Bazyar et al.2021	L	L	L	L	L	L
Bazyar et al.2022	L	L	L	L	L	L
Celinski et al.2014	H	U	L	L	U	H
Chojnacki et al.2011	L	L	L	L	L	L
Chojnacki et al. 2015	U	U	L	L	U	H
Chojnacki et al.2018	L	L	L	L	L	L
D'Anna et al.2017	H	U	L	L	U	H
Esalatmanesh et al.2021	L	L	L	L	L	L
Farrokhian et al.2019	L	L	L	L	L	L
Forrest et al.2007	L	L	L	L	L	L
Ganjifard et al. 2025	L	L	L	L	L	L
Ghaderi-Zefrehi et al.2024	L	L	L	L	L	L
Hoseini et al.2021	L	L	L	L	L	L
Gonciarz et al.2012	U	L	L	L	L	U
Goyal et al.2014	L	L	L	L	L	L
Grossman et al.2006	L	L	L	L	L	L
Hannemann et al. 2024	L	L	L	L	L	L
Hasan et al.2022	H	U	L	L	U	H
Jallouli et al.2025	L	L	L	L	L	L

Table S3. *Cont.*

**Reference**	**Bias arising from the randomization process** (Allocation bias)	**Bias due to** deviations from the intended interventions (Performance bias)	**Bias due to missing outcome data** (Attrition bias)	**Bias in the measurement of the Outcome** (Detection bias)	**Bias in the selection of reported results** (Reporting bias)	**The overall risk of bias**
Javanmard et al.2016	L	L	L	L	L	L
Zare Javid et al.2020	L	L	L	L	L	L
Kari et al.2019	H	U	L	L	U	H
Kim et al.2021	L	L	L	L	L	L
Kotlarczyk et al.2012	U	L	L	L	L	U
Larki et al.2025	L	L	L	L	L	L
Lauritzen et al.2022	L	L	L	L	L	L
Leonardo-Mendonça et al.2017	L	L	L	L	L	L
Luz et al.2025	L	L	L	L	L	L
Marzougui et al.2024	L	L	L	L	L	L
Modabbernia et al.2014	L	L	L	L	L	L
Mohammadi et al.2021	L	L	L	L	L	L
Mohammadi et al. 2025	L	L	L	L	L	L
Mousavi et al.2022	L	L	L	L	L	L
Nabatian-Asl et al.2021	L	L	L	L	L	L
Nunes et al.2008	L	L	L	L	L	L
Ortiz-Franco et al.2017	L	L	L	L	L	L
Pakravan et al.2017	L	L	L	L	L	L
Panah et al. 2019	L	L	L	L	L	L
Rechciński et al.2010	H	U	L	L	U	H
Rezvanfar et al.2017	U	L	L	L	L	U
Rigamonti et al.2024	L	L	L	L	L	L
Rindone et al.1997	U	U	L	L	U	H
Rondanelli et al.2018	L	L	L	L	L	L
Romo-Nava et al.2014	L	L	L	L	L	L
Sadeghi et al.2025	L	L	L	L	L	L
Sánchez-López et al.2018	L	L	L	L	L	L
Seabra et al.2000	U	L	L	L	L	U
Szewczyk-Golec et al.2017	L	L	L	L	L	L
Talari et al.2022	U	L	L	L	L	U

Abbreviations: L, low risk of bias; H, high risk of bias; U, unclear risk of bias (some concerns)

Table S4. GRADE assessment

Outcomes	Risk of bias	Inconsistency	Indirectness	Imprecision	Publication bias	Quality of evidence
**BW**	No serious limitation	No serious limitation	No serious limitation	No serious limitation	Serious limitation[3]	⊕⊕⊕⊖ Moderate
**BMI**	No serious limitation	Serious limitation[1]	No serious limitation	No serious limitation	No serious limitation	⊕⊕⊕⊖ Moderate
**WC**	No serious limitation	No serious limitation	No serious limitation	No serious limitation	No serious limitation	⊕⊕⊕⊕ High
**HC**	No serious limitation	No serious limitation	No serious limitation	Serious limitation[2]	No serious limitation	⊕⊕⊕⊖ Moderate
**BFP**	No serious limitation	No serious limitation	No serious limitation	No serious limitation	No serious limitation	⊕⊕⊕⊕ High
**FBG**	No serious limitation	Serious limitation[1]	No serious limitation	Serious limitation[2]	No serious limitation	⊕⊕⊖⊖ Low
**HbA1c**	No serious limitation	Serious limitation[1]	No serious limitation	No serious limitation	No serious limitation	⊕⊕⊕⊖ Moderate
**FI**	No serious limitation	Serious limitation[1]	No serious limitation	Serious limitation[2]	No serious limitation	⊕⊕⊖⊖ Low
**HOMA-IR**	No serious limitation	No serious limitation	No serious limitation	No serious limitation	No serious limitation	⊕⊕⊕⊕ High
**TG**	No serious limitation	Serious limitation[1]	No serious limitation	Serious limitation[2]	No serious limitation	⊕⊕⊖⊖ Low
**TC**	No serious limitation	Serious limitation[1]	No serious limitation	Serious limitation[2]	No serious limitation	⊕⊕⊖⊖ Low
**LDL-C**	No serious limitation	Serious limitation[1]	No serious limitation	Serious limitation[2]	No serious limitation	⊕⊕⊖⊖ Low
**HDL-C**	No serious limitation	Serious limitation[1]	No serious limitation	Serious limitation[2]	No serious limitation	⊕⊕⊖⊖ Low
**SBP**	No serious limitation	Serious limitation[1]	No serious limitation	Serious limitation[2]	No serious limitation	⊕⊕⊖⊖ Low
**DBP**	No serious limitation	Serious limitation[1]	No serious limitation	Serious limitation[2]	No serious limitation	⊕⊕⊖⊖ Low
**MDA**	No serious limitation	Serious limitation[1]	No serious limitation	Serious limitation[2]	Serious limitation[3]	⊕⊖⊖⊖ Very low
**TAC**	No serious limitation	Serious limitation[1]	No serious limitation	No serious limitation	Serious limitation[3]	⊕⊕⊖⊖ Low
**CRP**	No serious limitation	Serious limitation[1]	No serious limitation	No serious limitation	No serious limitation	⊕⊕⊕⊖ Moderate
**IL-6**	No serious limitation	Serious limitation[1]	No serious limitation	Serious limitation[2]	No serious limitation	⊕⊕⊖⊖ Low
**TNF-α**	No serious limitation	Serious limitation[1]	No serious limitation	Serious limitation[2]	No serious limitation	⊕⊕⊖⊖ Low

Table S4. *Cont.*

Outcomes	Risk of bias	Inconsistency	Indirectness	Imprecision	Publication bias	Quality of evidence
**AST**	No serious limitation	Serious limitation[1]	No serious limitation	Serious limitation[2]	No serious limitation	⊕⊕⊖⊖ Low
**ALT**	No serious limitation	Serious limitation[1]	No serious limitation	Serious limitation[2]	No serious limitation	⊕⊕⊖⊖ Low
**GGT**	No serious limitation	Serious limitation[1]	No serious limitation	Serious limitation[2]	No serious limitation	⊕⊕⊖⊖ Low

Abbreviations: BMI, body mass index; BFP, body fat percentage; WC, waist circumference; SBP, systolic blood pressure; BW, body weight; DBP, diastolic blood pressure; HC, hip circumference; MDA, malondialdehyde; ALT, alanine aminotransferase; AST, aspartate aminotransferase; TG, triglycerides; TC, total cholesterol, LDL-C, low-density lipoprotein cholesterol; HDL-C, high-density lipoprotein cholesterol; FBG, fasting blood glucose; HbA1c, hemoglobin A1c; HOMA-IR, homeostatic model assessment of insulin resistance; TAC, total antioxidant capacity; FI, fasting insulin; CRP, C-reactive protein; GGT, gamma- glutamyl transferase; IL-6, interleukin- 6; TNF-α, tumor necrosis factor alpha.

[1] It was downgraded due to the presence of significant heterogeneity (I^2>50%).
[2] It was downgraded because the confidence interval for the effect size range was > 2.
[3] It was downgraded due to publication bias based on Begg's or Egger's test.

A) BW

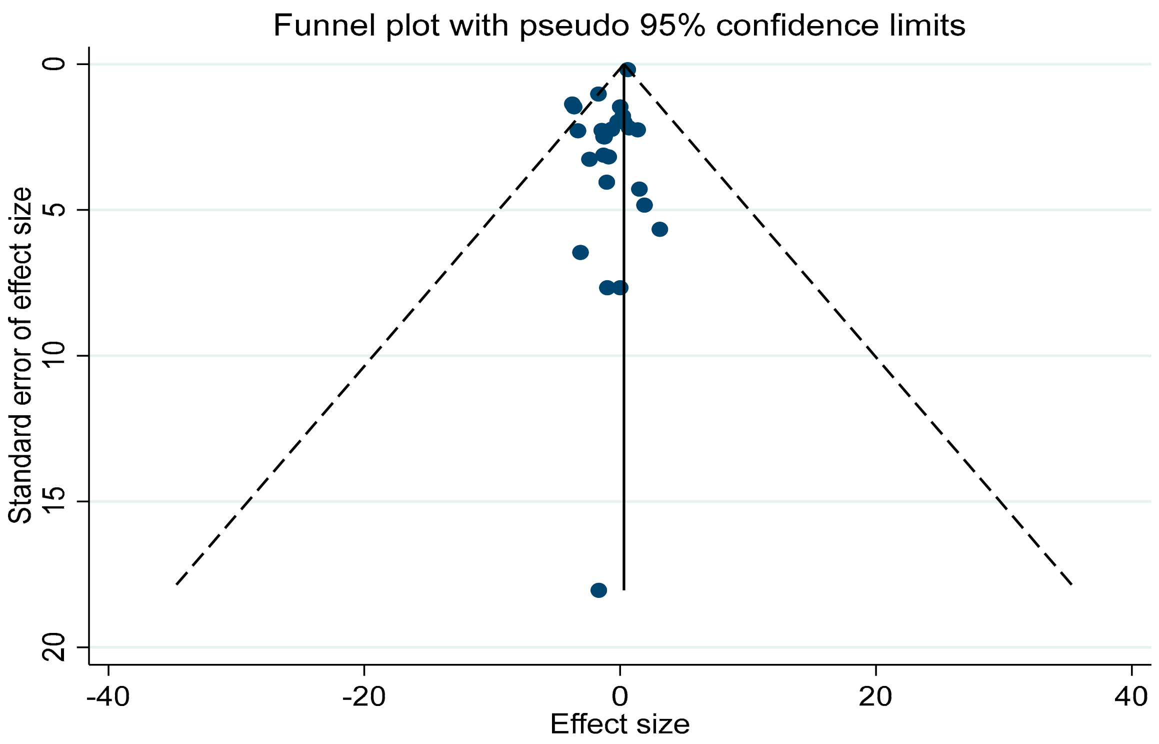

B) BMI

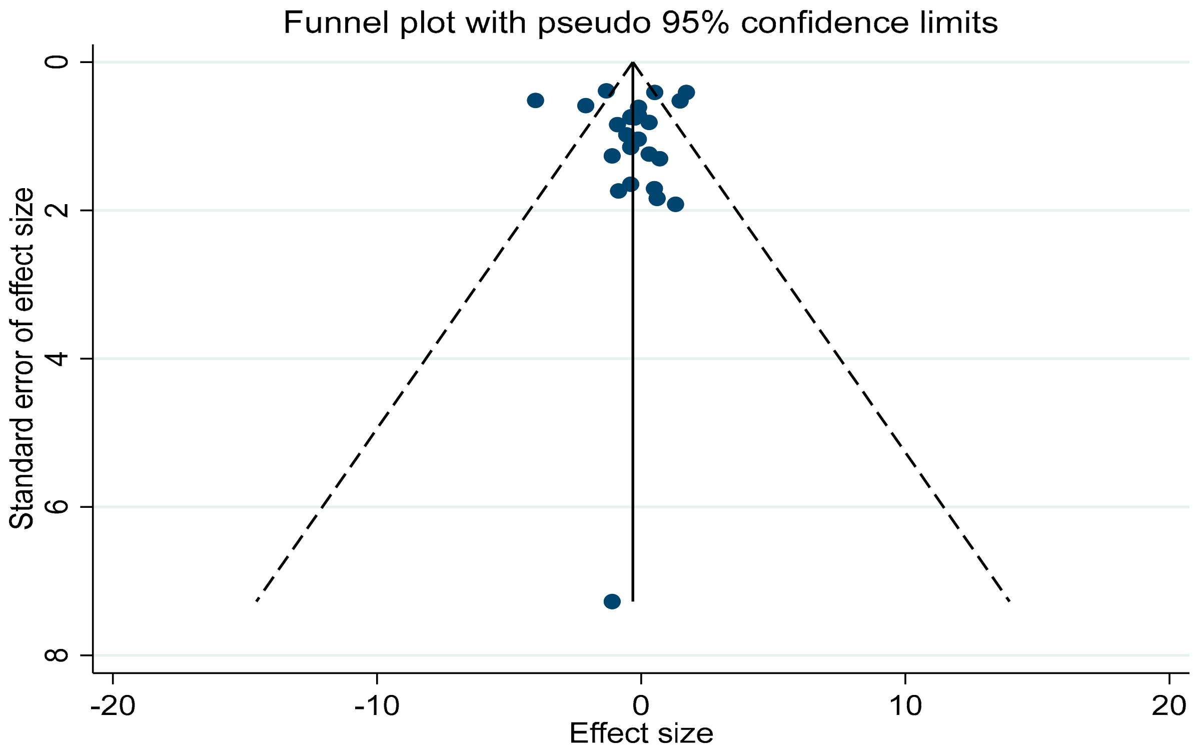

Figure S1. *Cont.*

C) WC

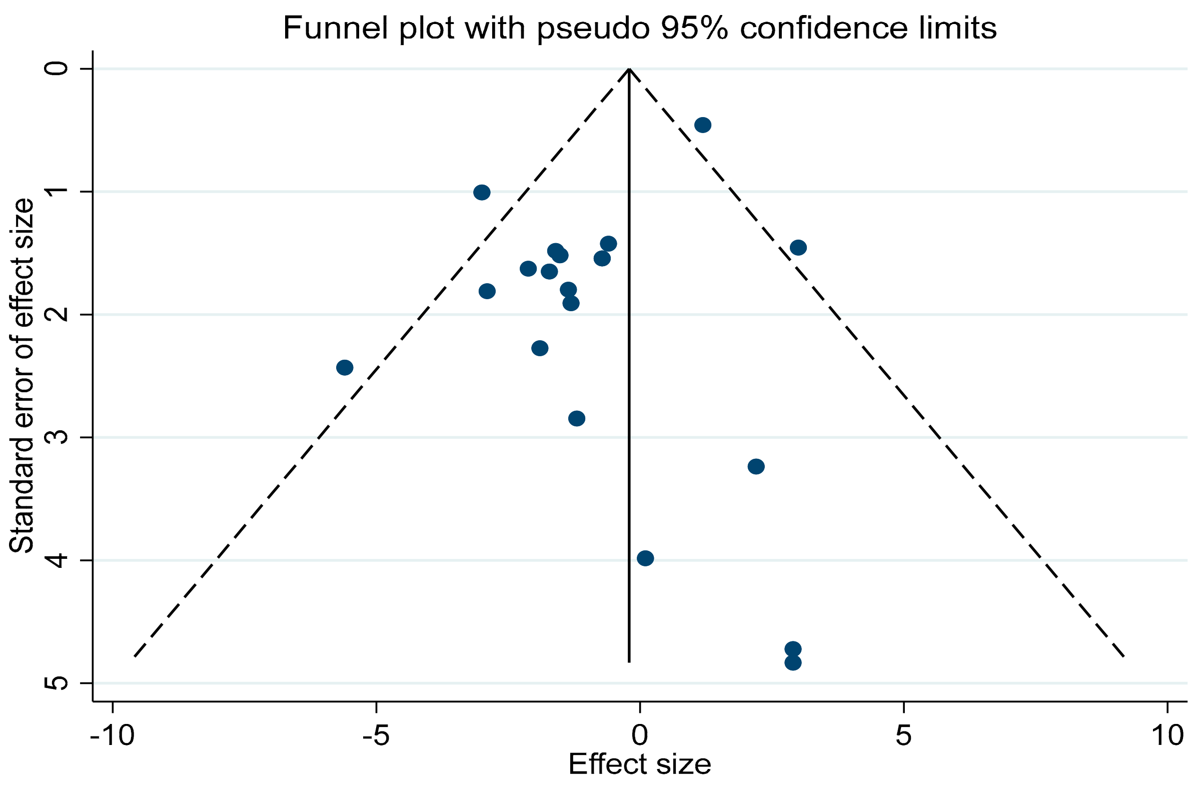

D) HC

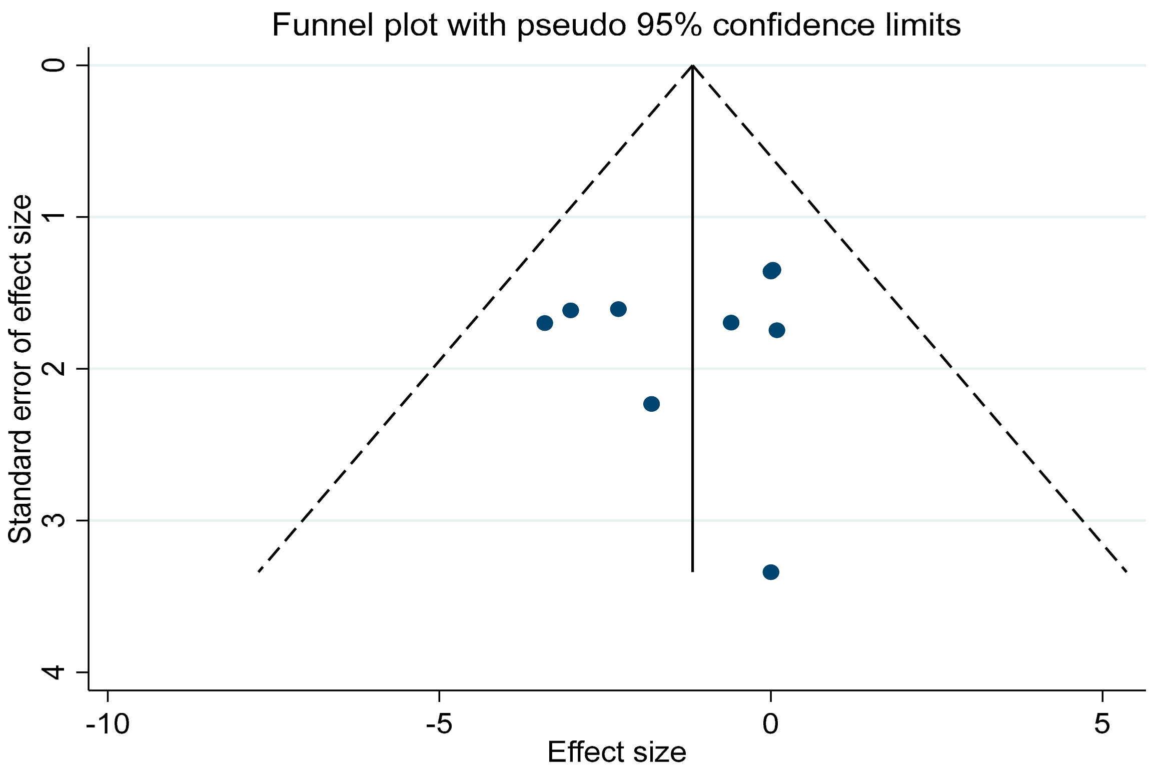

Figure S1. *Cont.*

E) BFP

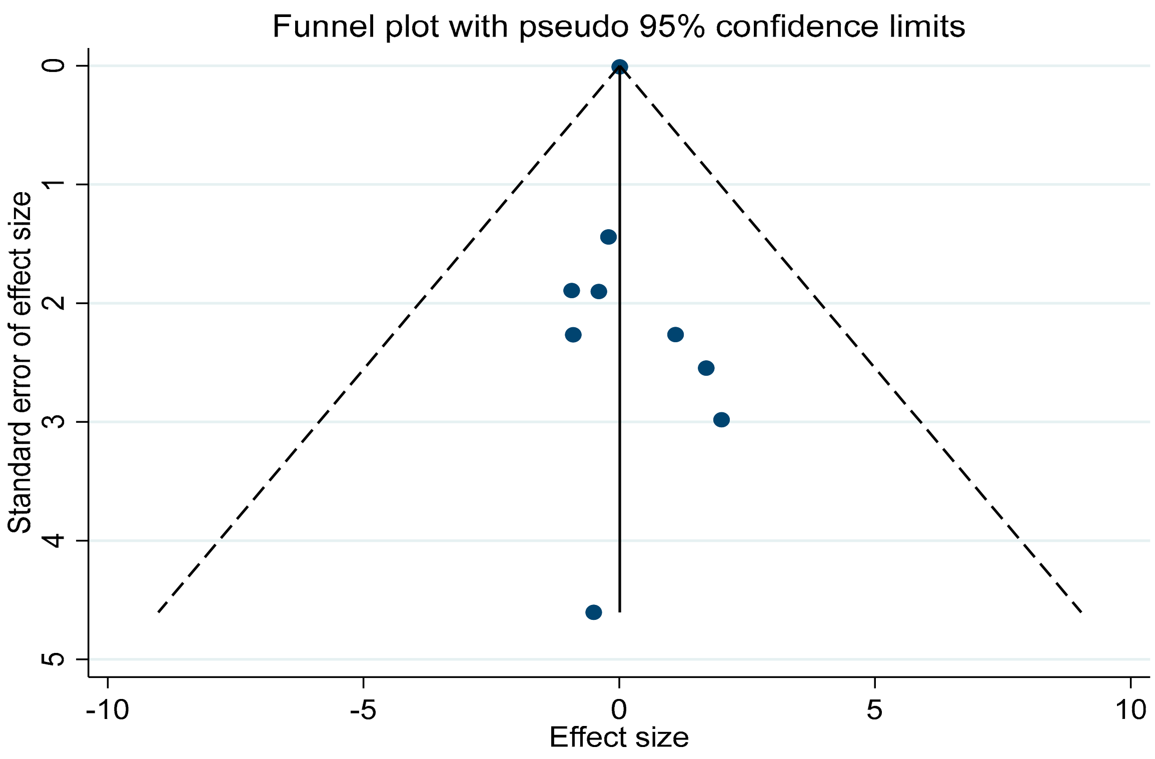

F) FBG

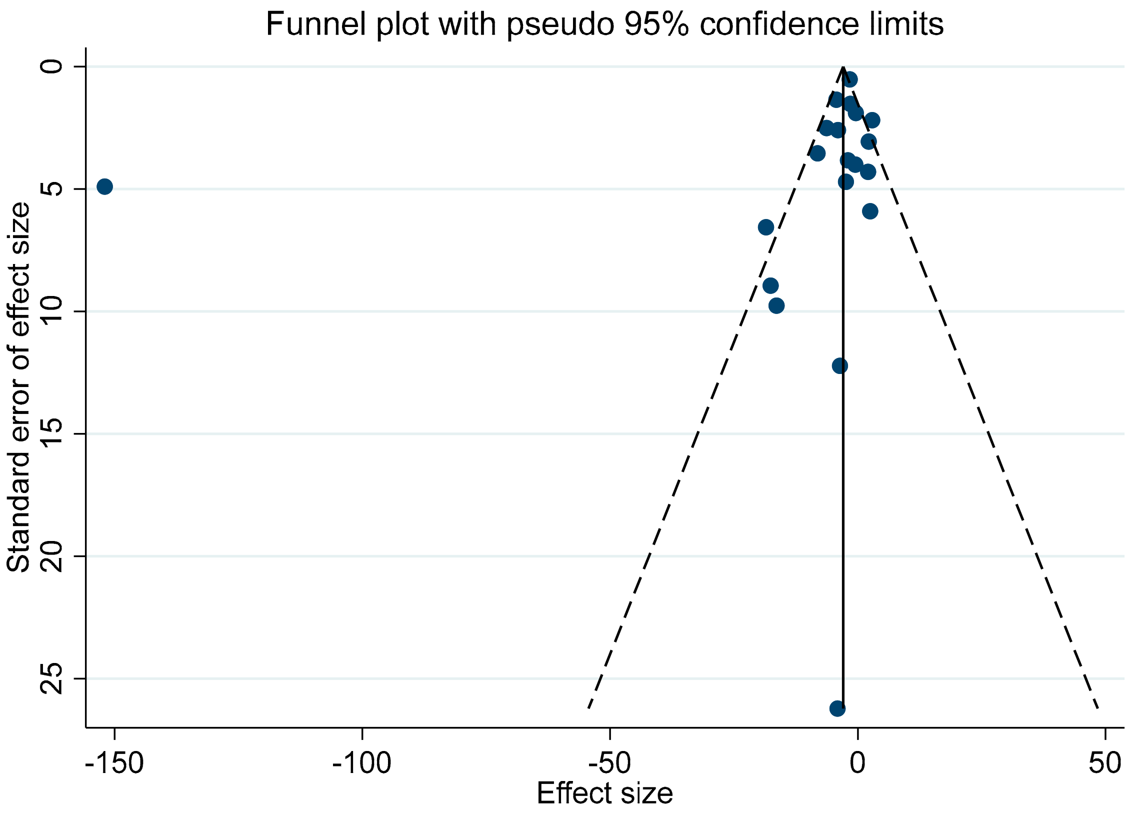

Figure S1. *Cont.*

G) HbA1c

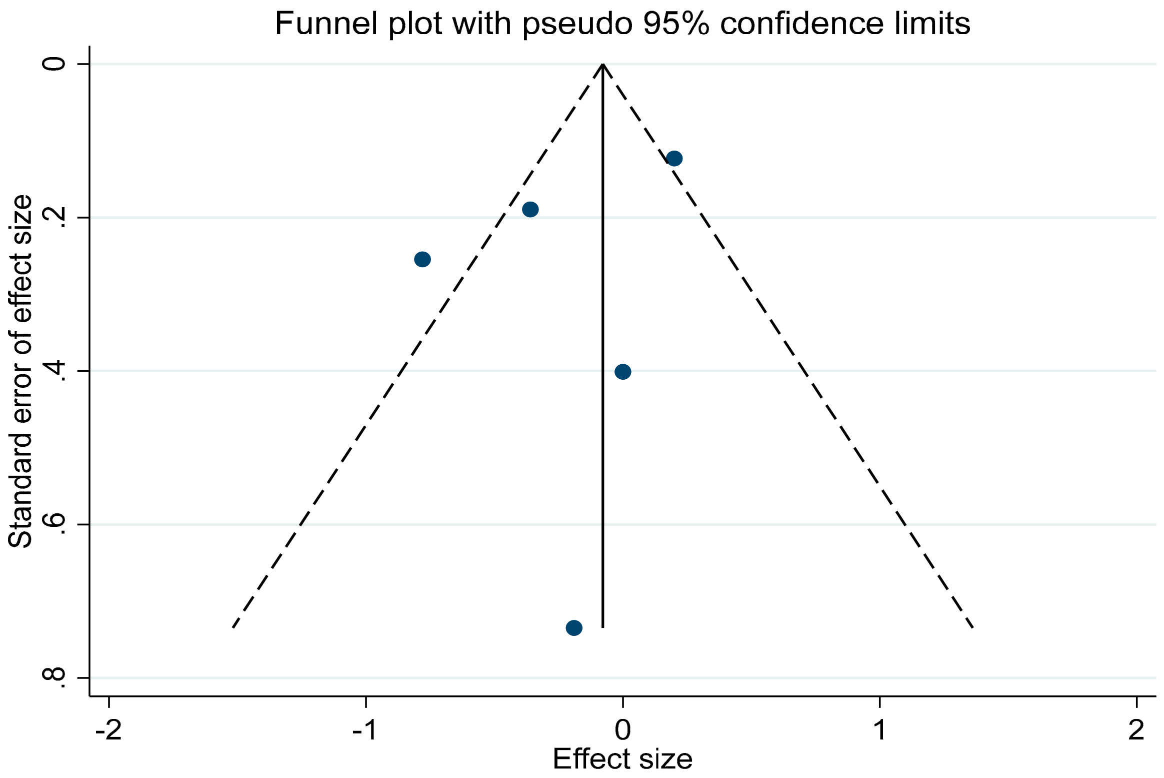

H) FI

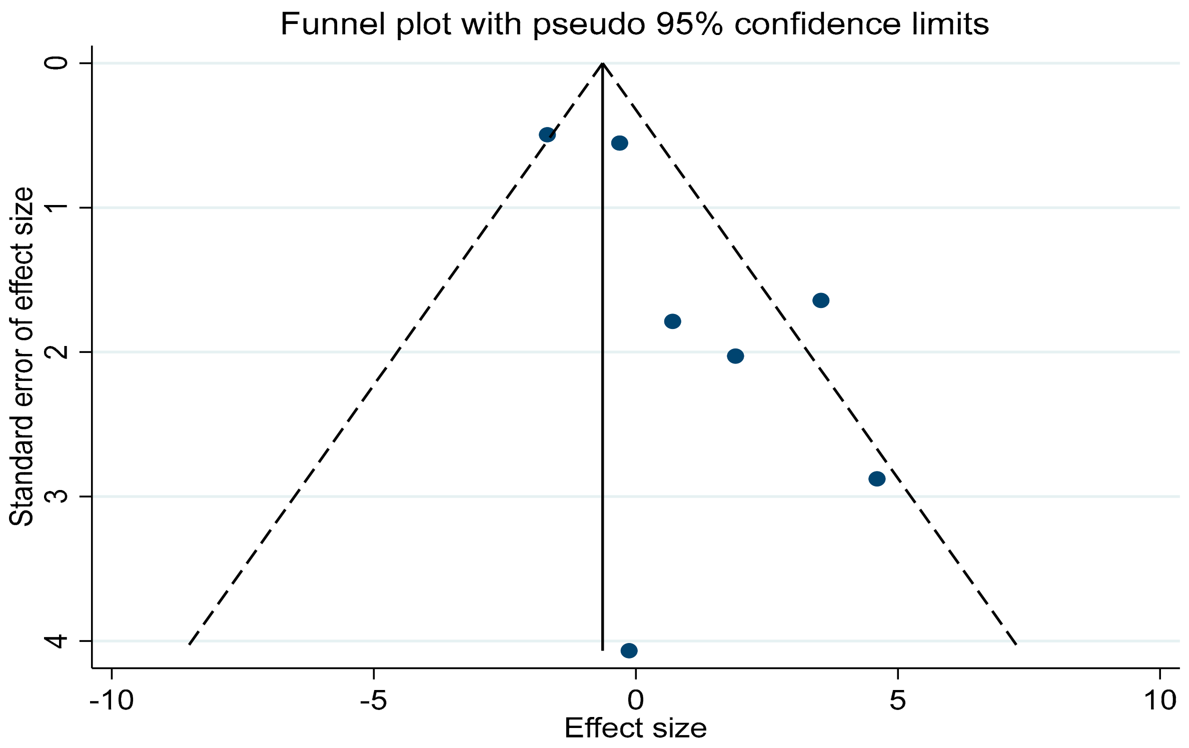

Figure S1. *Cont.*

I) HOMA-IR

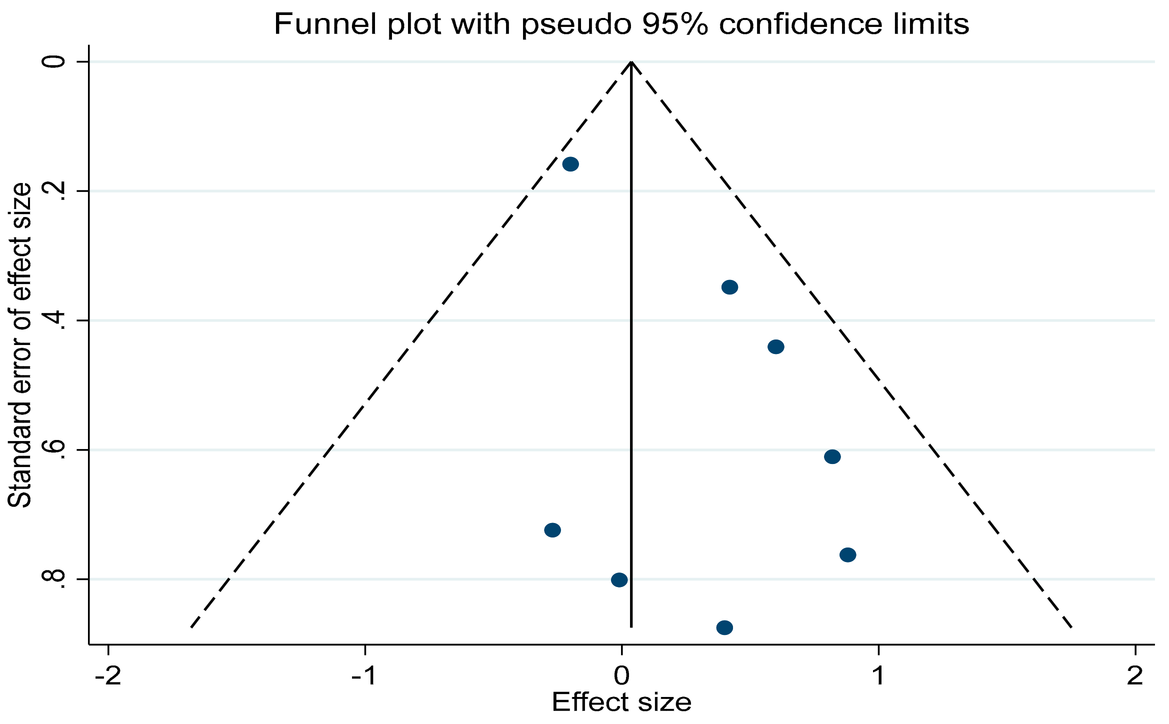

J) TG

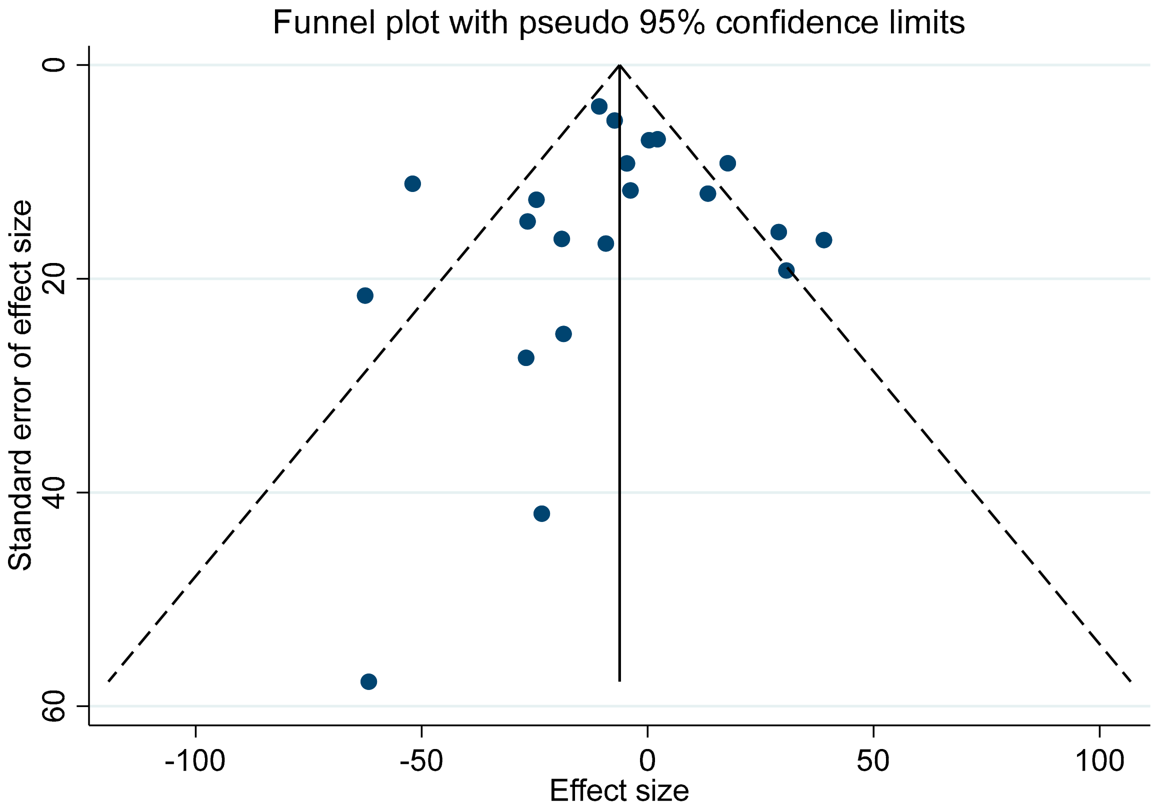

Figure S1. *Cont.*

K) TC

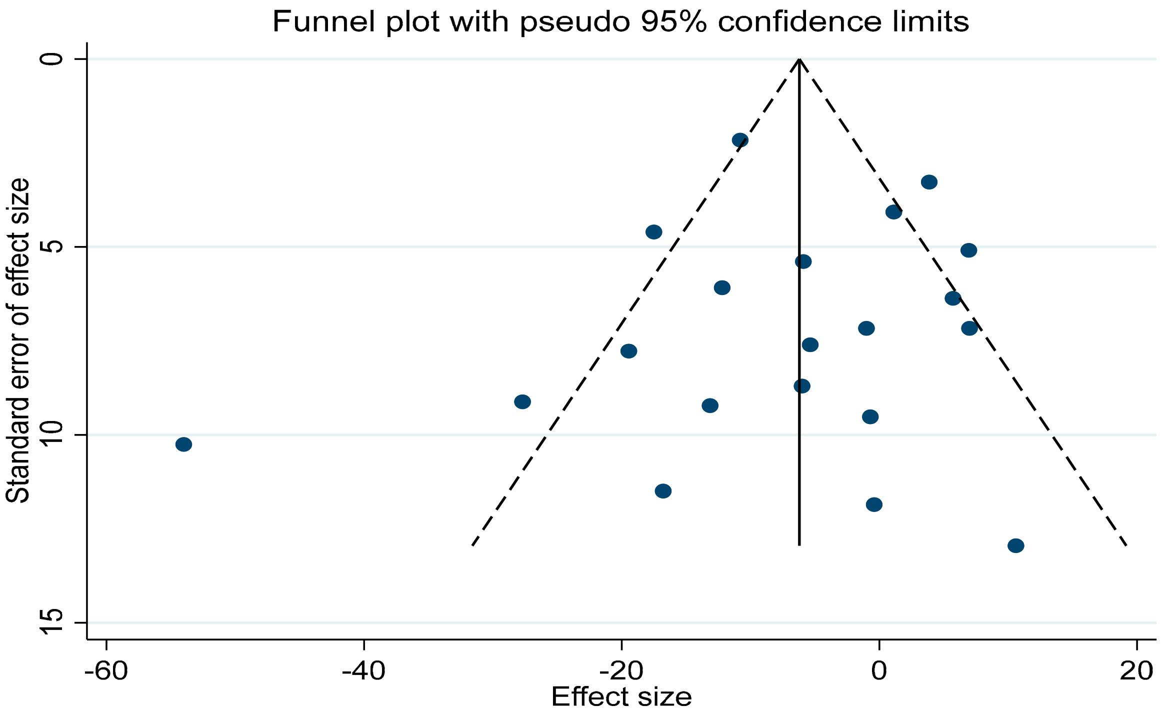

L) LDL-C

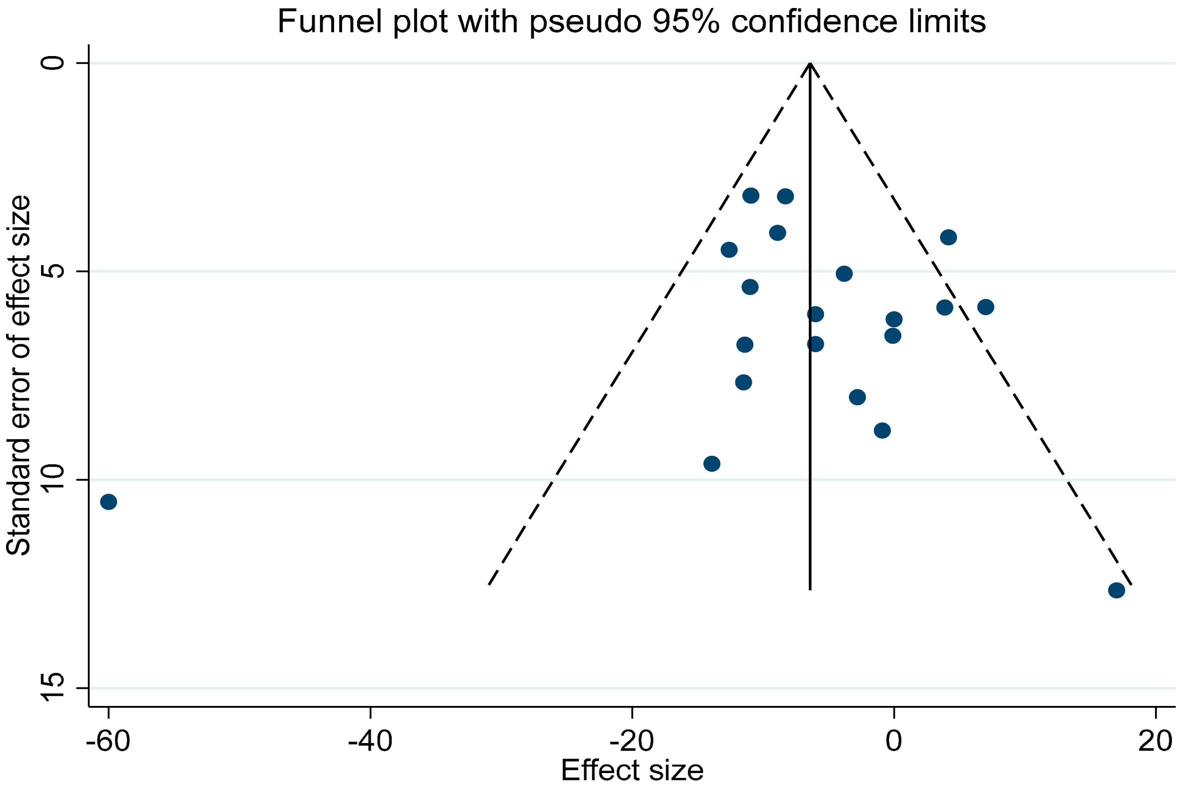

Figure S1. *Cont.*

M) HDL-C

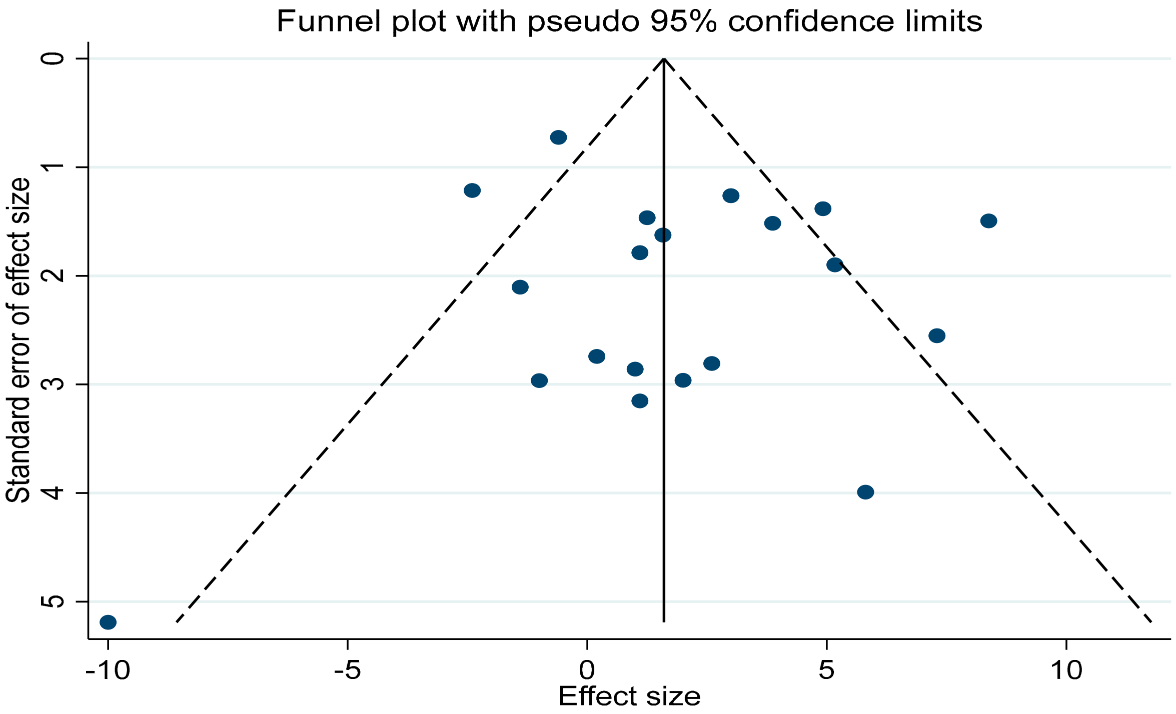

N) SBP

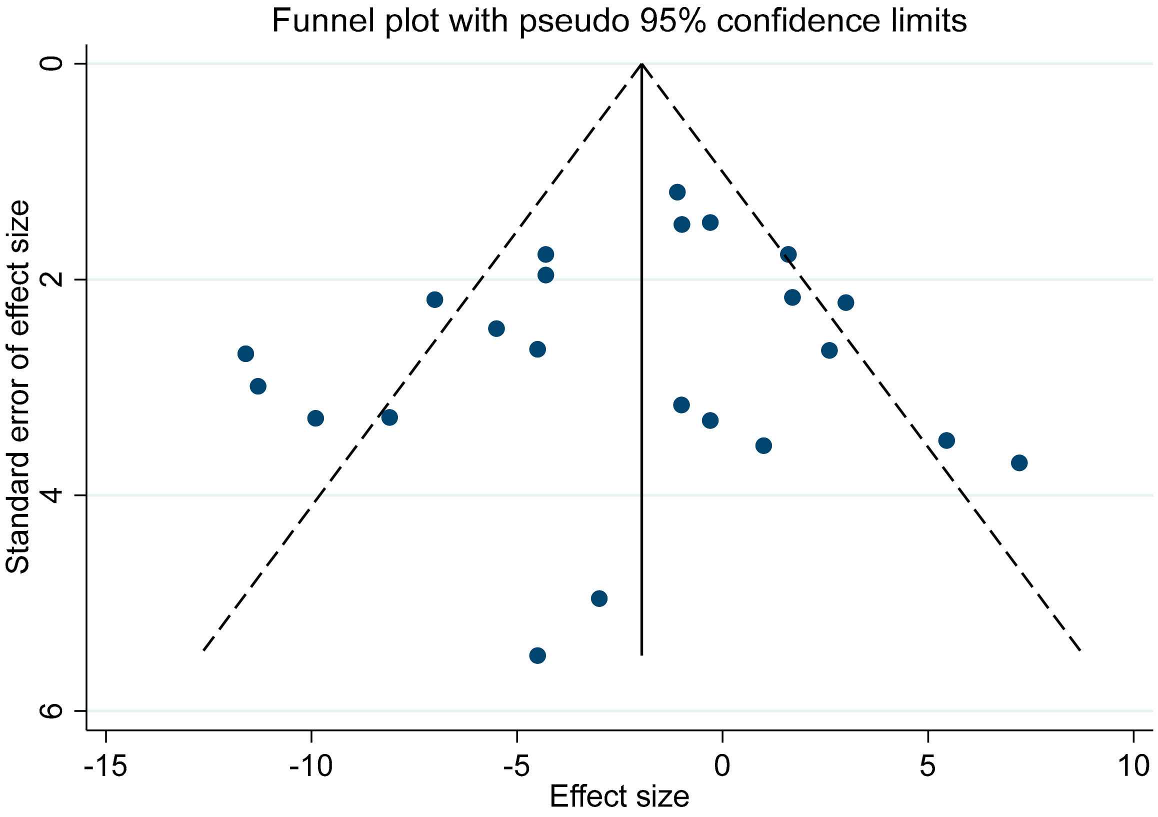

Figure S1. *Cont.*

O) DBP

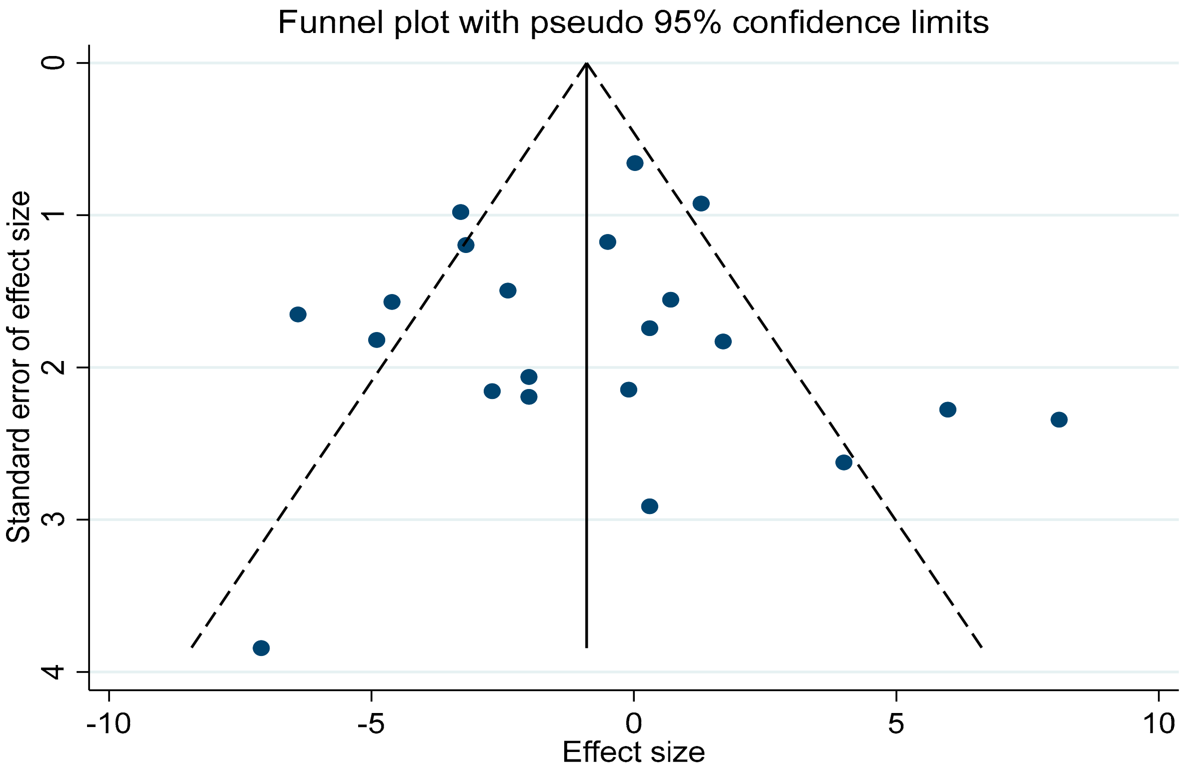

P) MDA

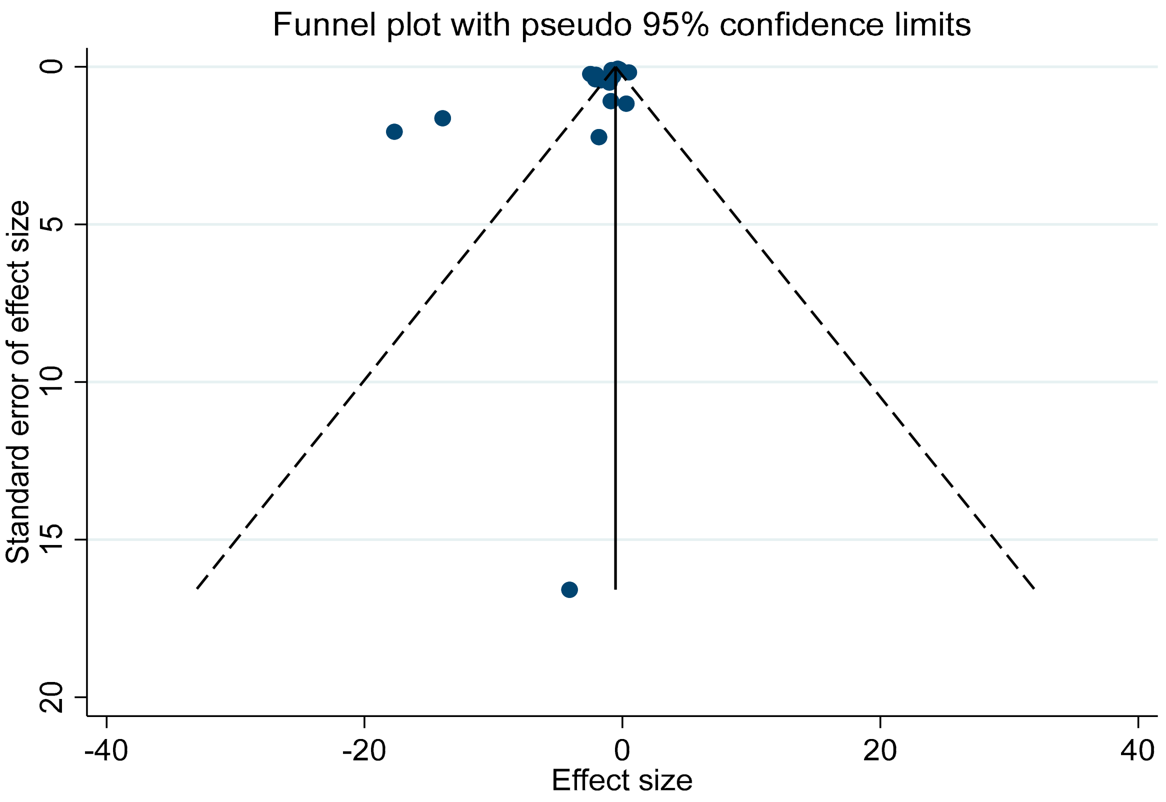

Figure S1. *Cont.*

Q) TAC

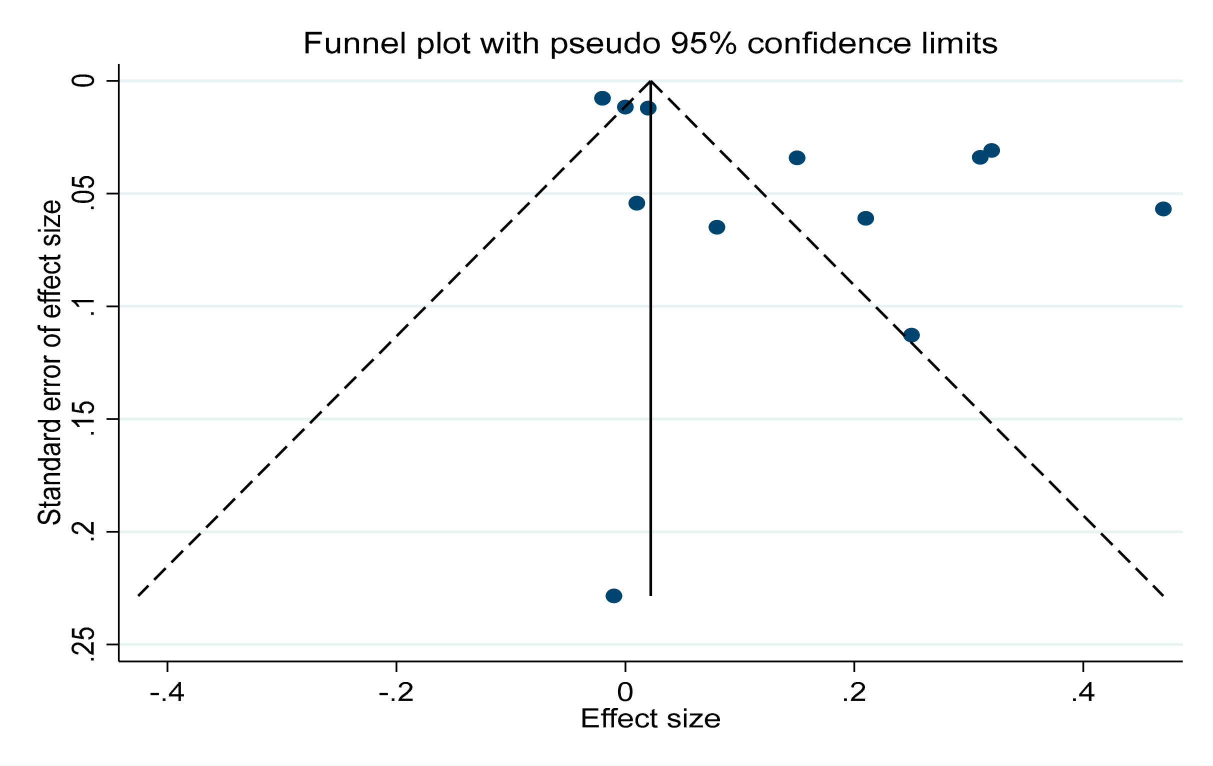

R) CRP

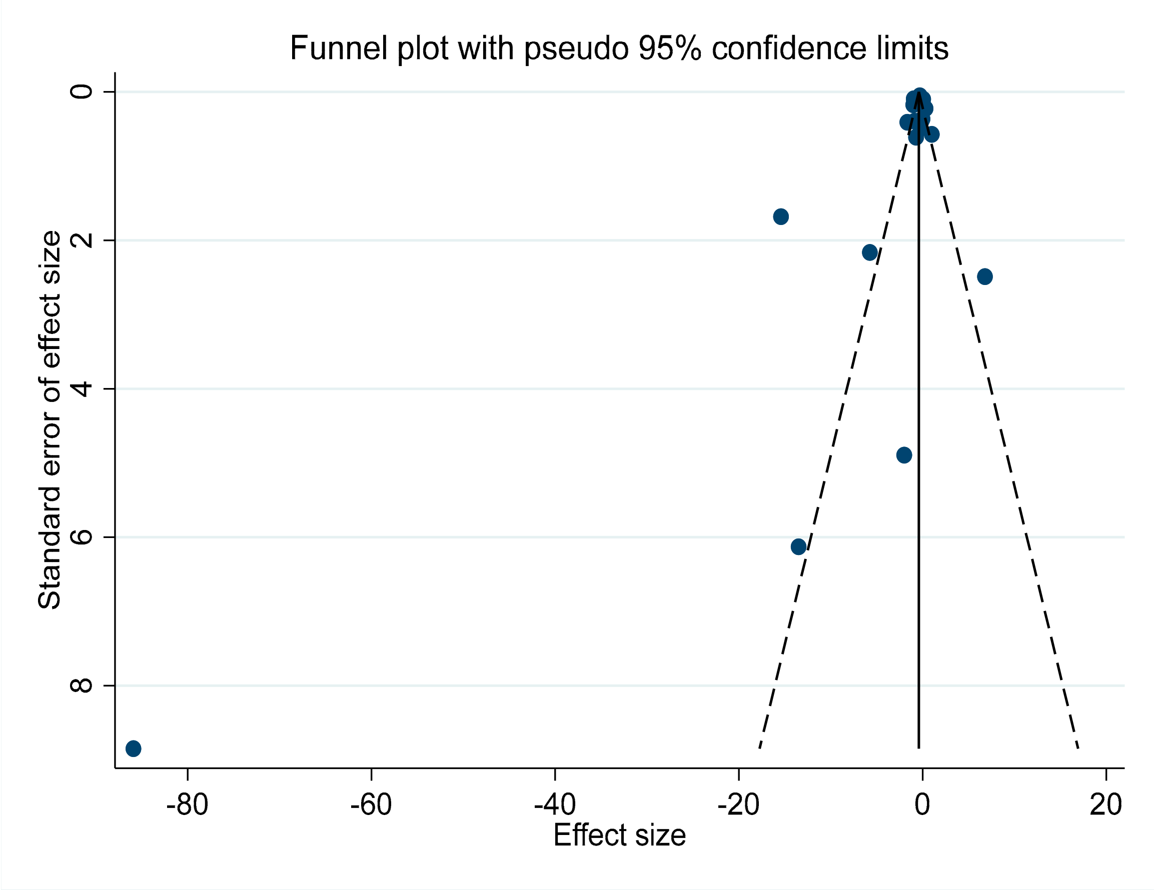

Figure S1. *Cont.*

S) IL-6

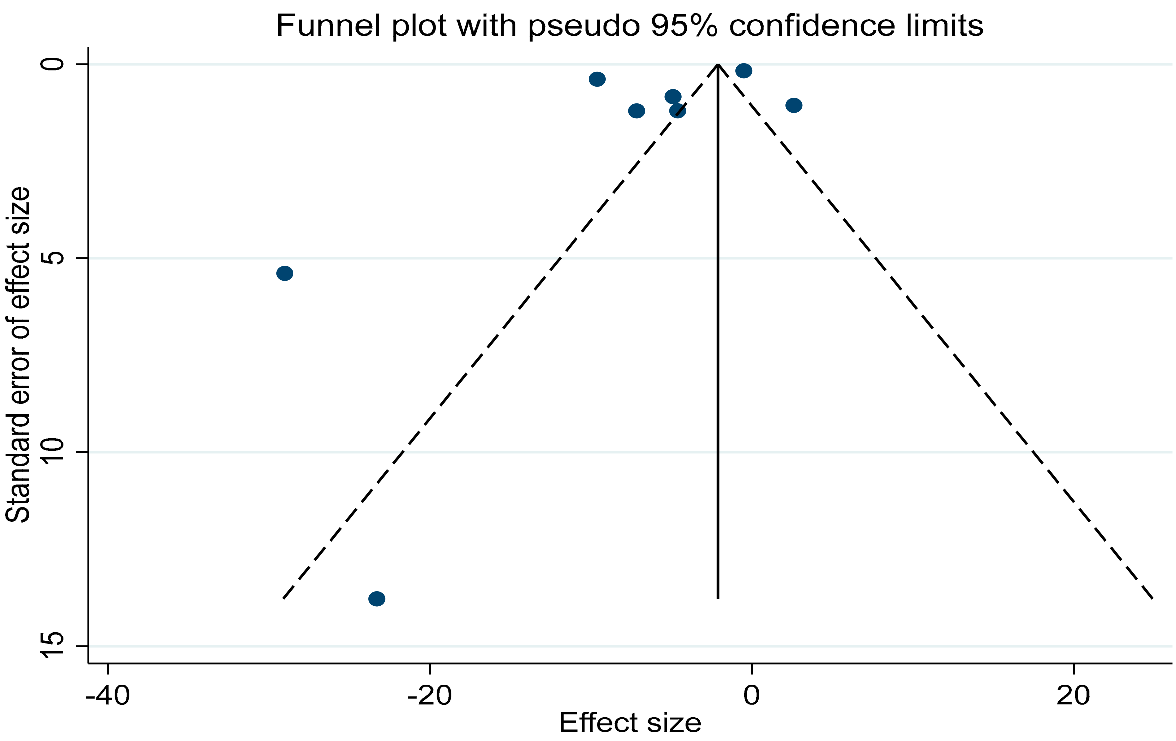

T) TNF-α

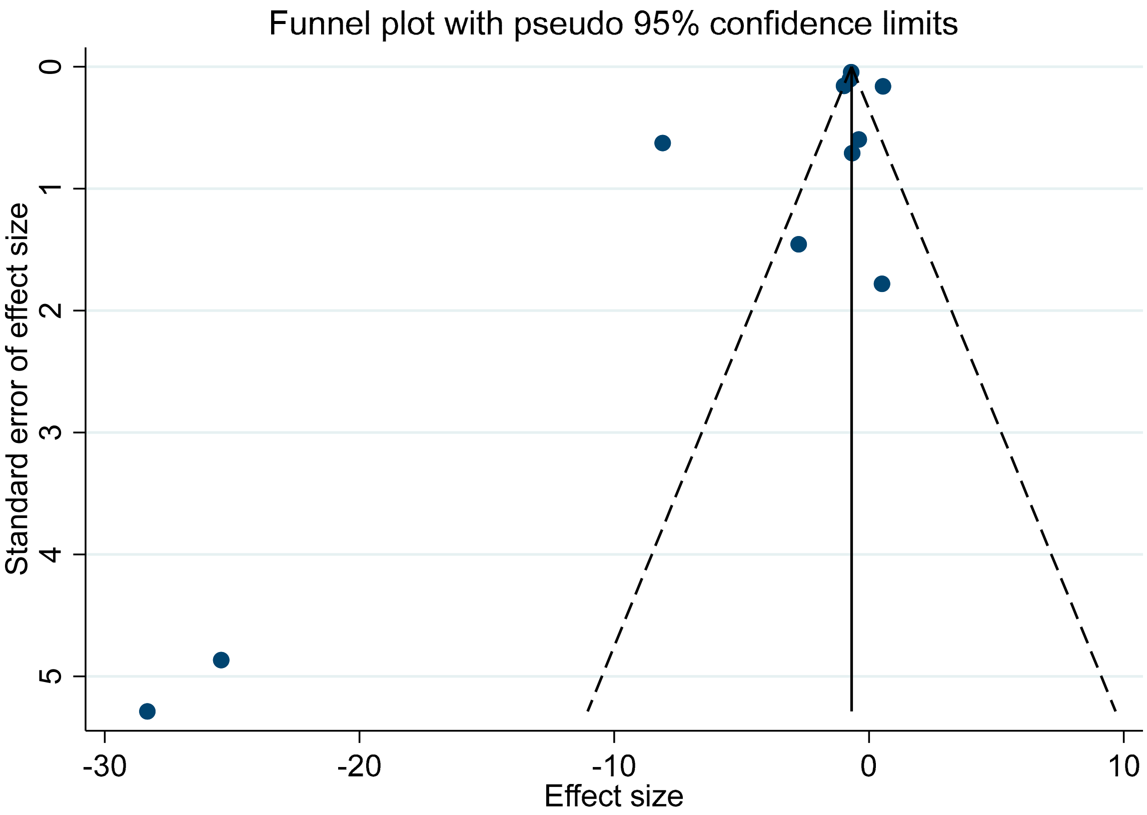

Figure S1. *Cont.*

U) AST

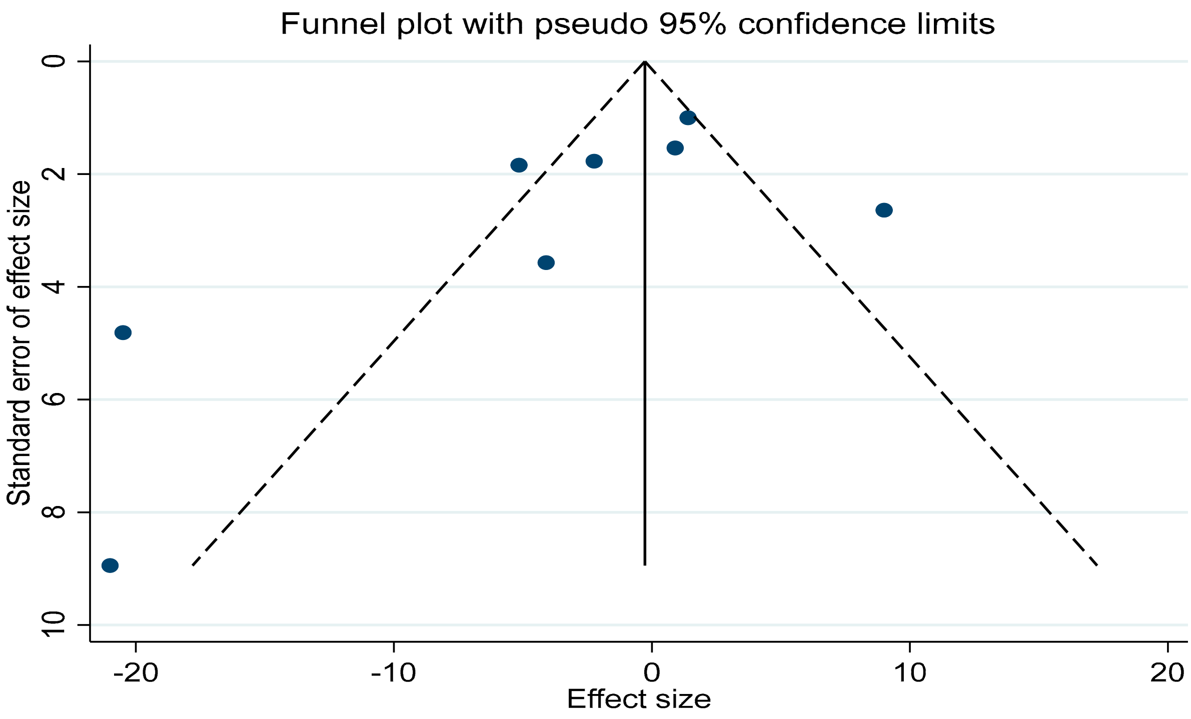

V) ALT

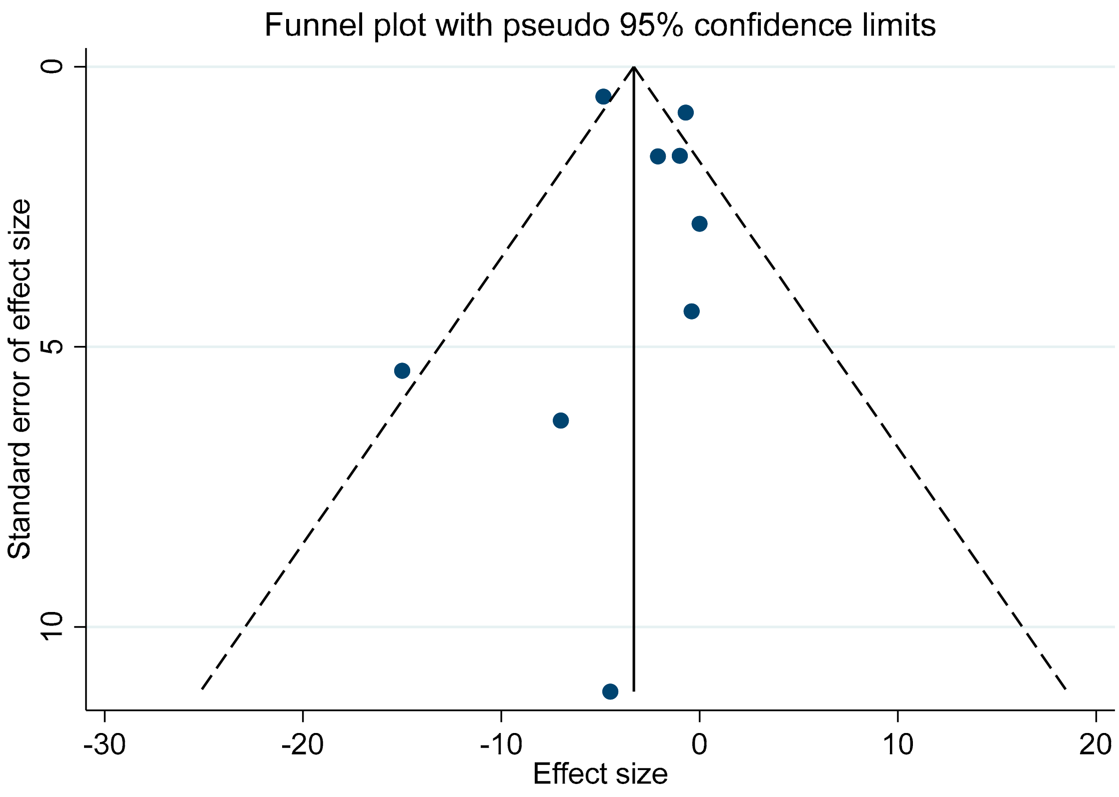

Figure S1. *Cont.*

W) GGT

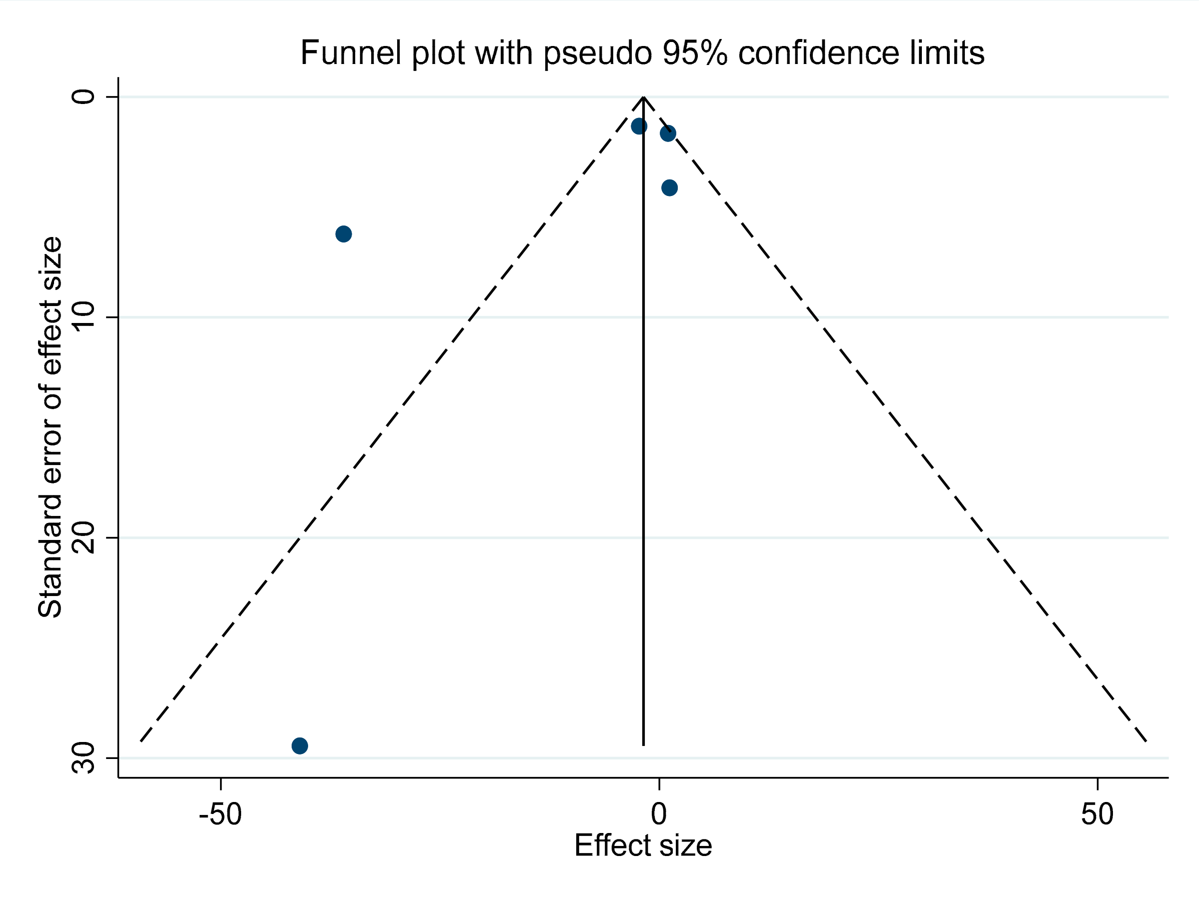

Figure S1. Funnel plots for the effects of melatonin supplementation on cardiometabolic risk factors (CMRFs), including **(A)** BW, **(B)** BMI, **(C)** WC, **(D)** HC, **(E)** BFP, **(F)** FBG, **(G)** HbA1c, **(H)** FI, **(I)** HOMA-IR, **(J)** TG, **(K)** TC, **(L)** LDL-C, **(M)** HDL-C, **(N)** SBP, **(O)** DBP, **(P)** MDA, **(Q)** TAC, **(R)** CRP, **(S)** IL-6, **(T)** TNF-α, **(U)** AST, **(V)**ALT, and **(W)** GGT.

A) BW

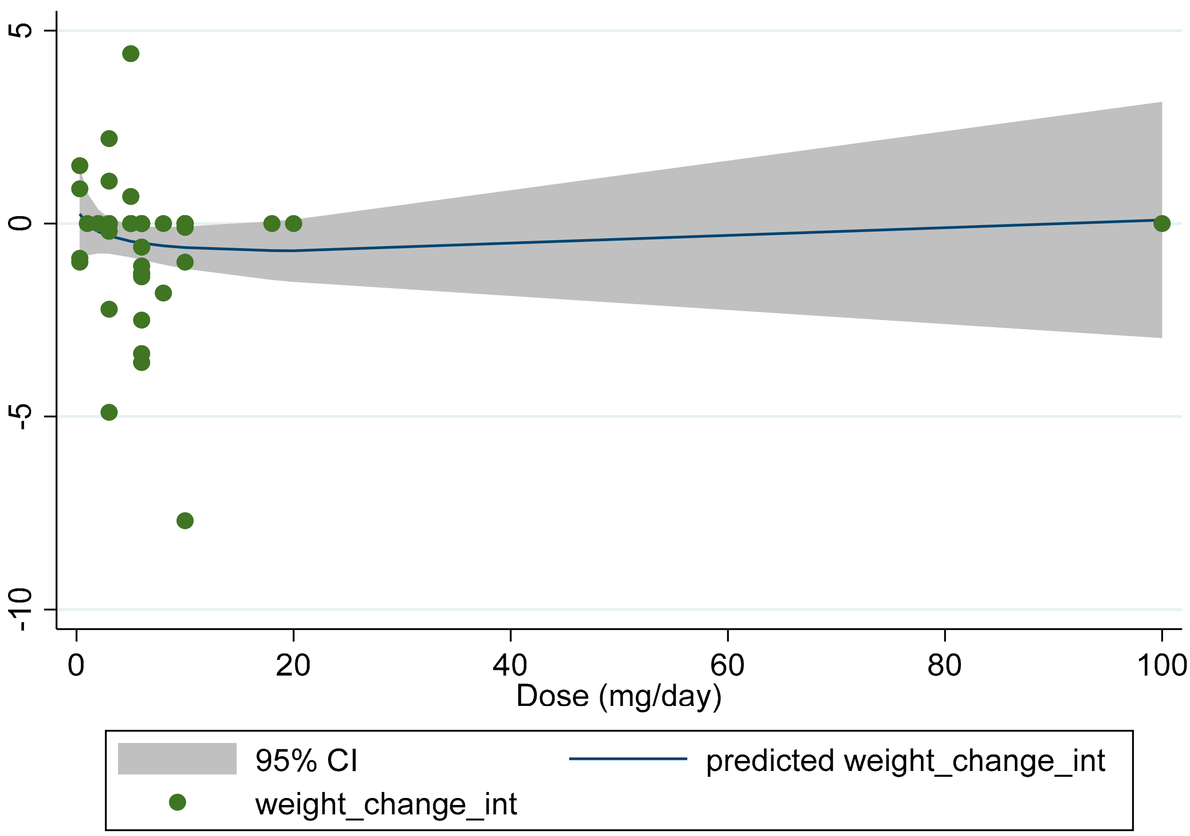

B) BMI

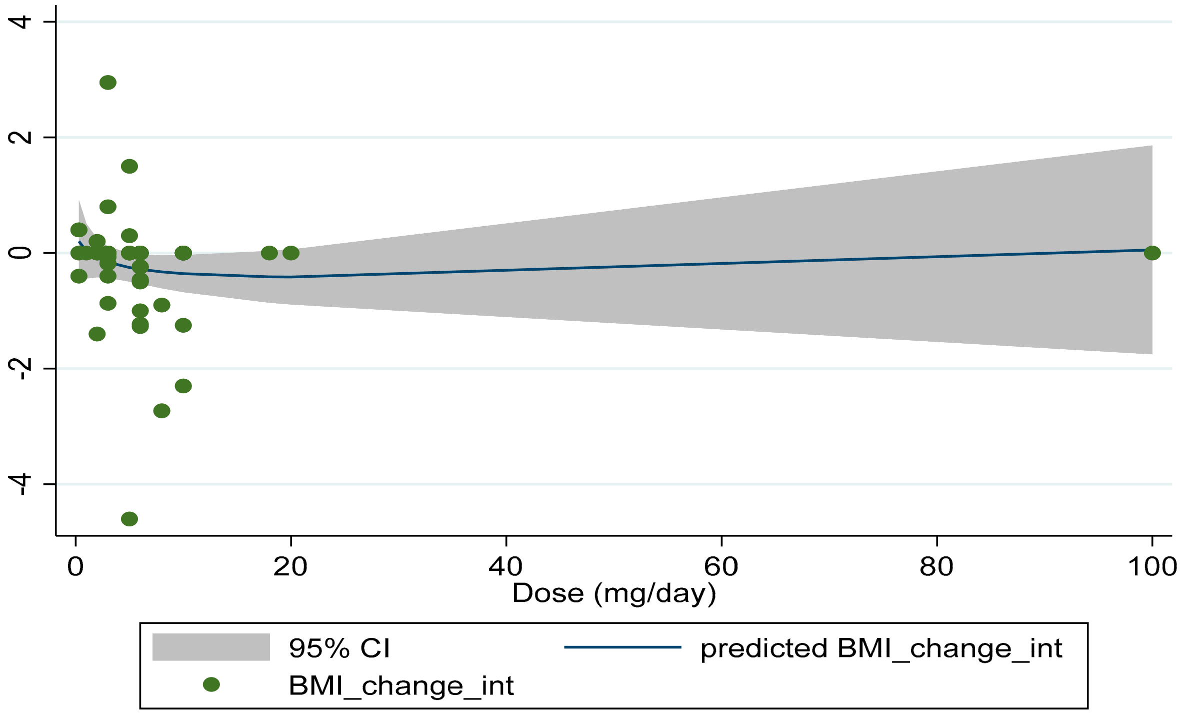

Figure S2. *Cont.*

C) WC

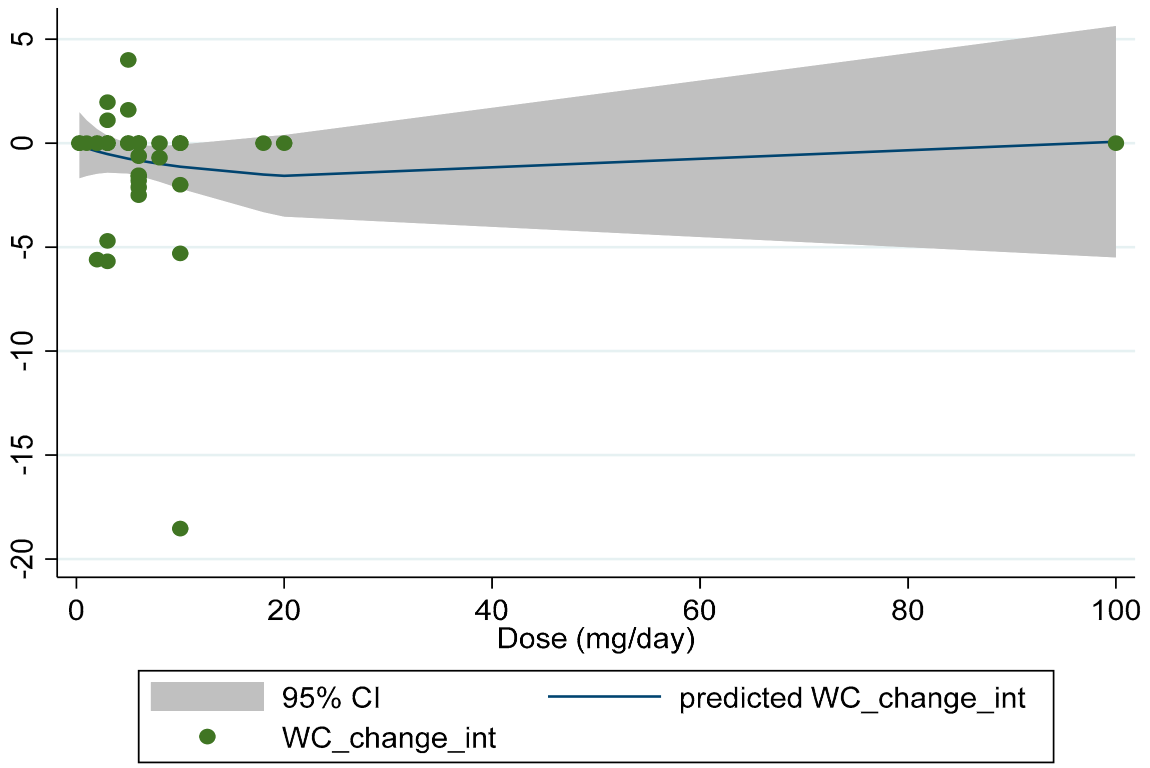

D) HC

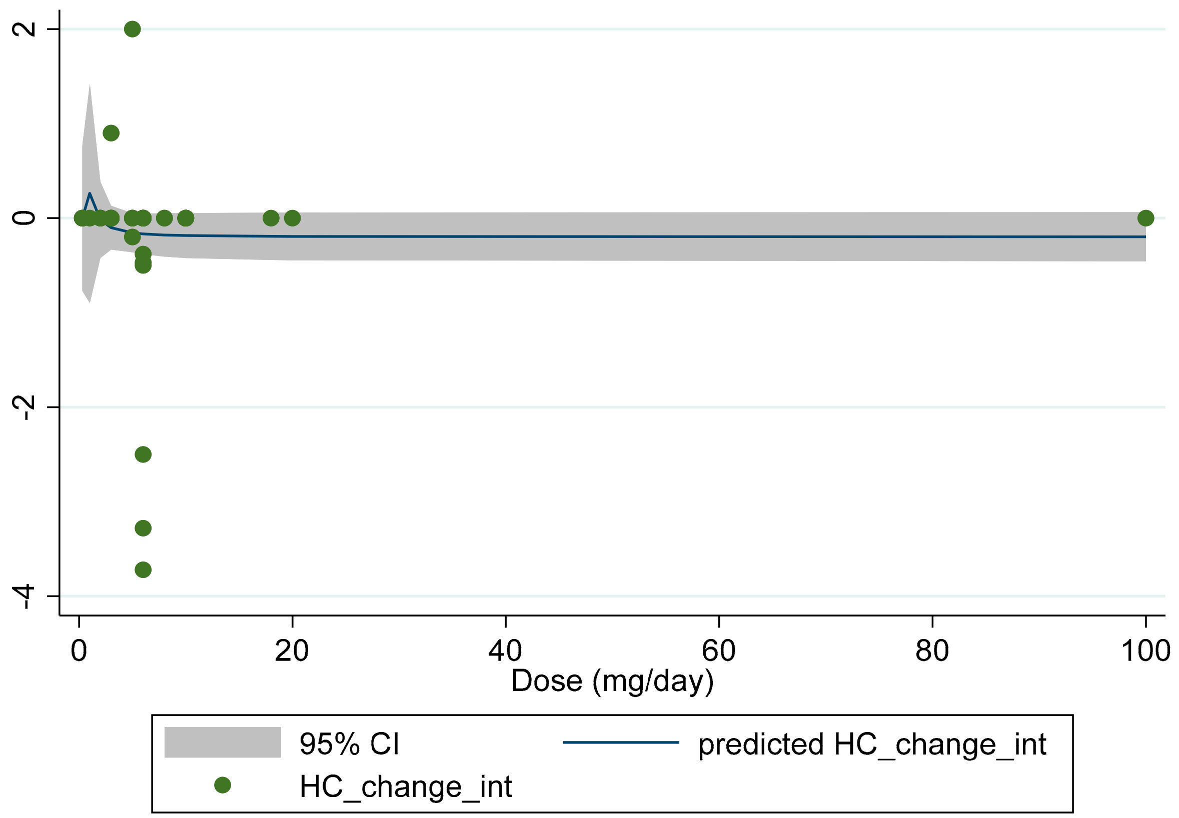

Figure S2. *Cont.*

E) BFP

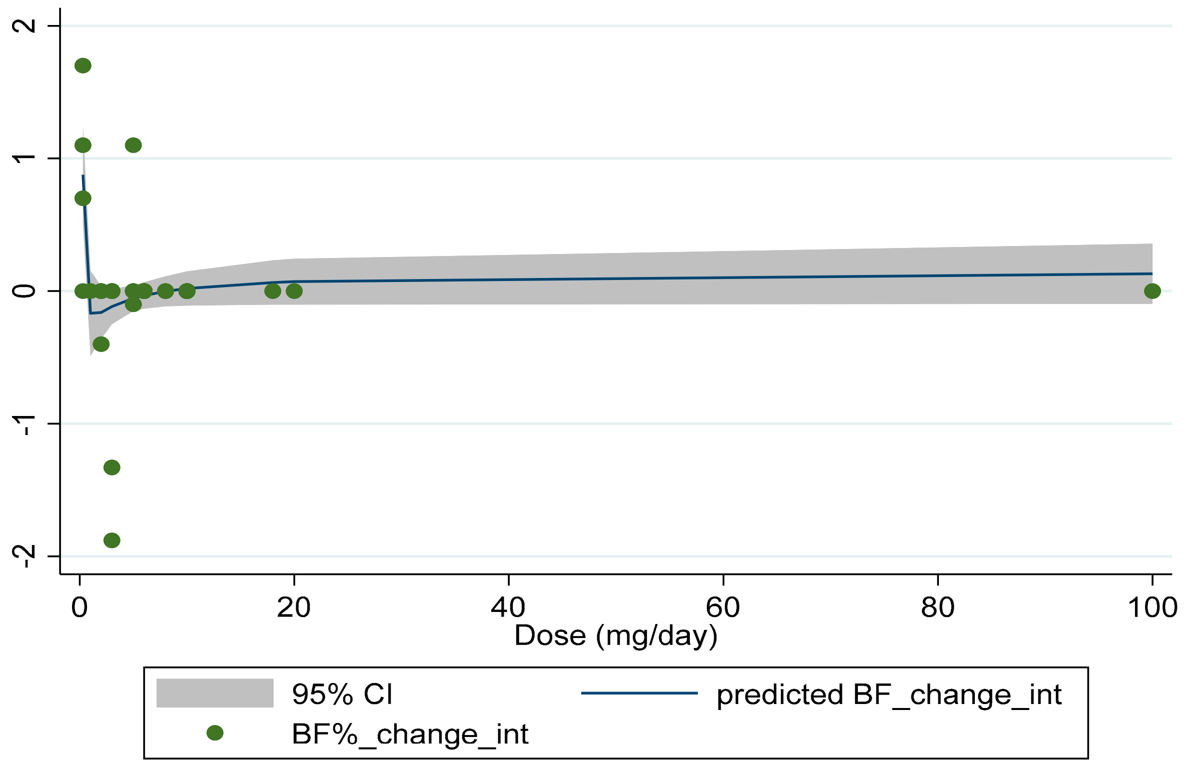

F) FBG

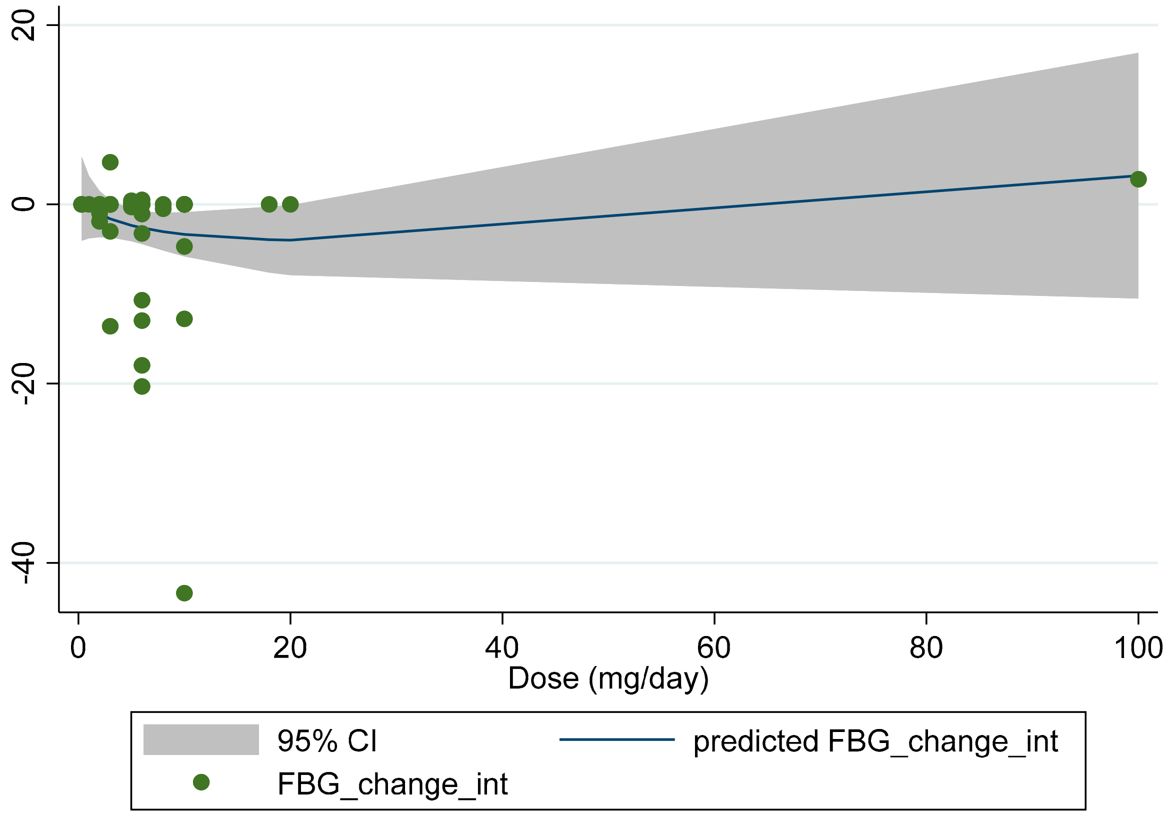

Figure S2. *Cont.*

G) HbA1c

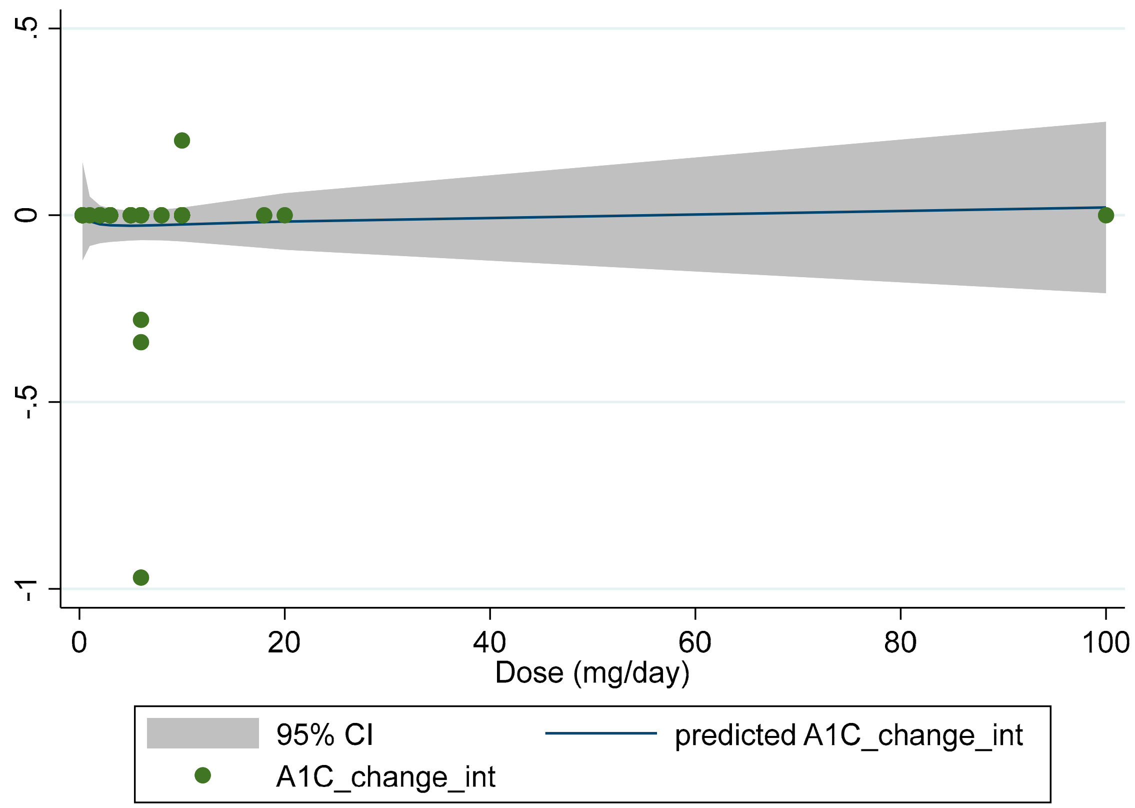

H) FI

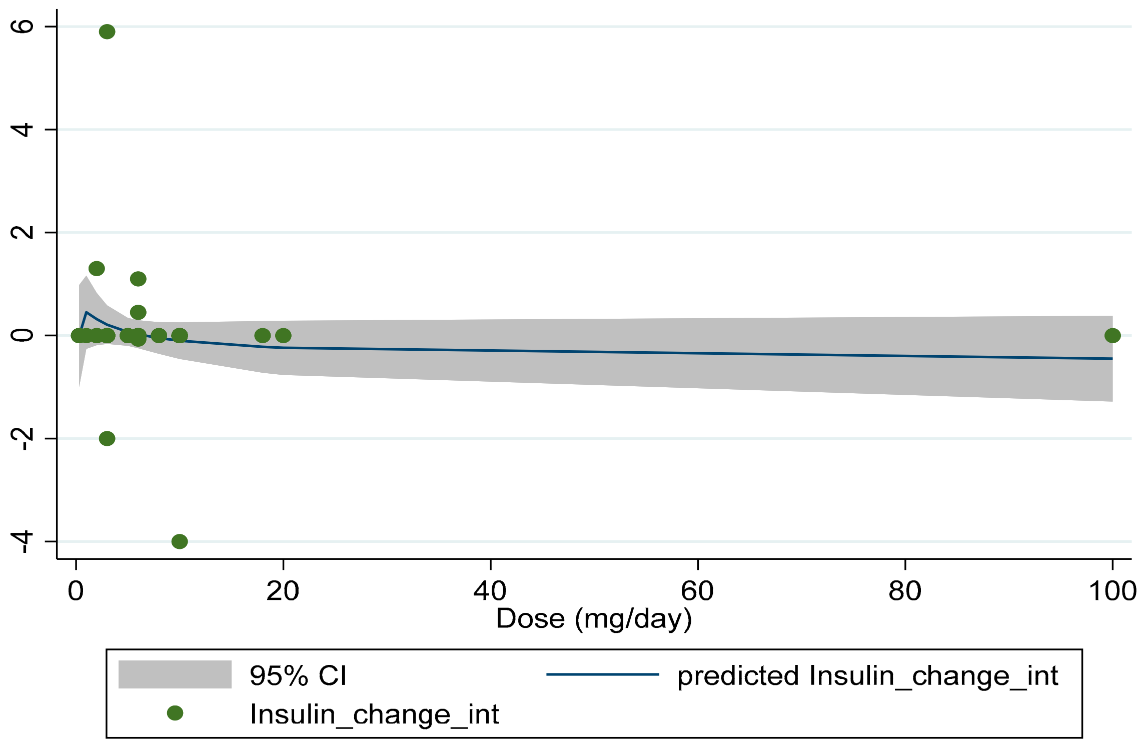

Figure S2. *Cont.*

I) HOMA-IR

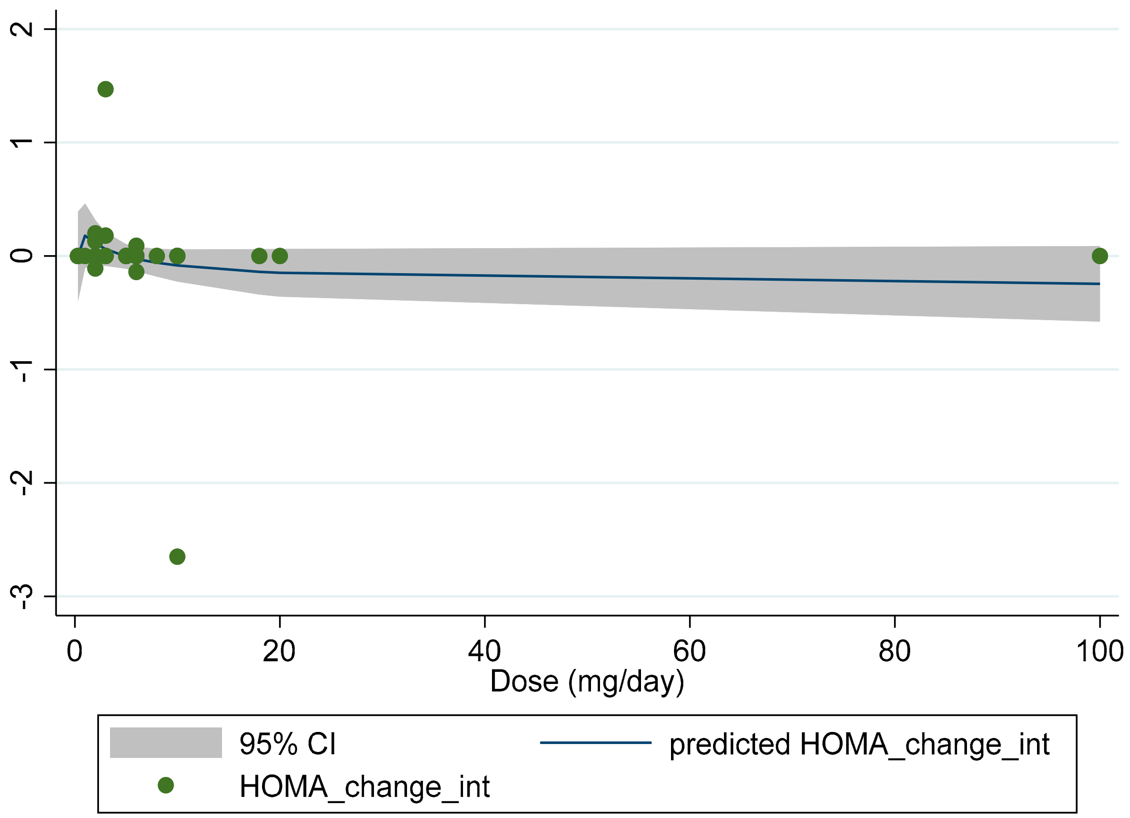

J) TG

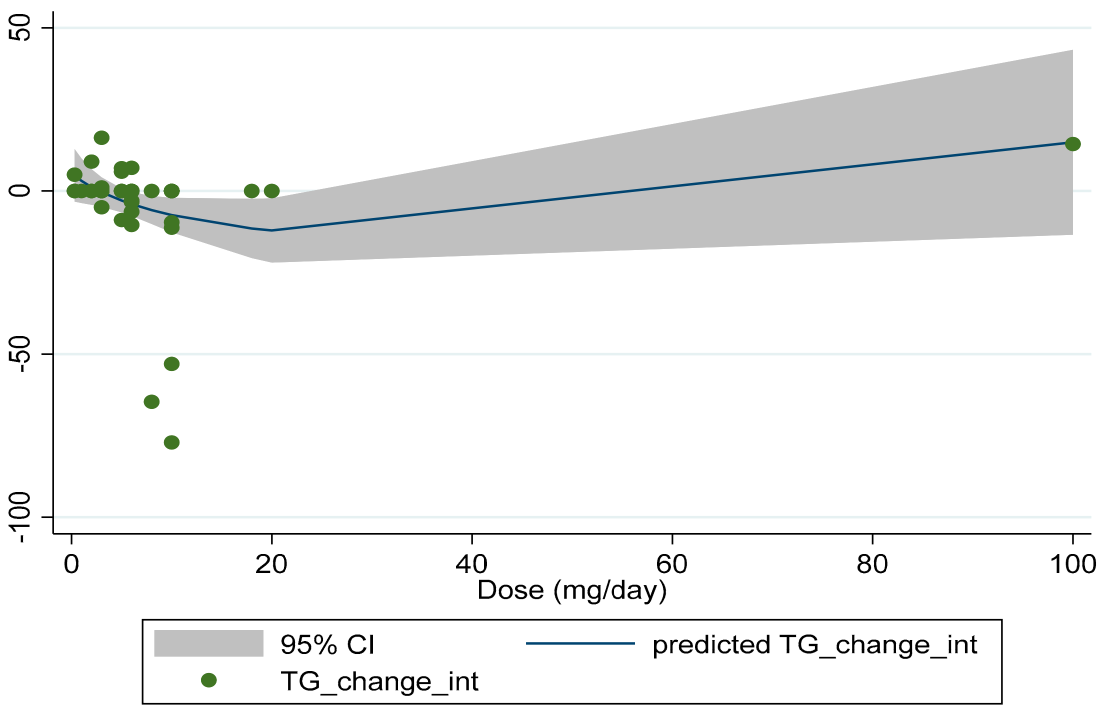

Figure S2. *Cont.*

K) TC

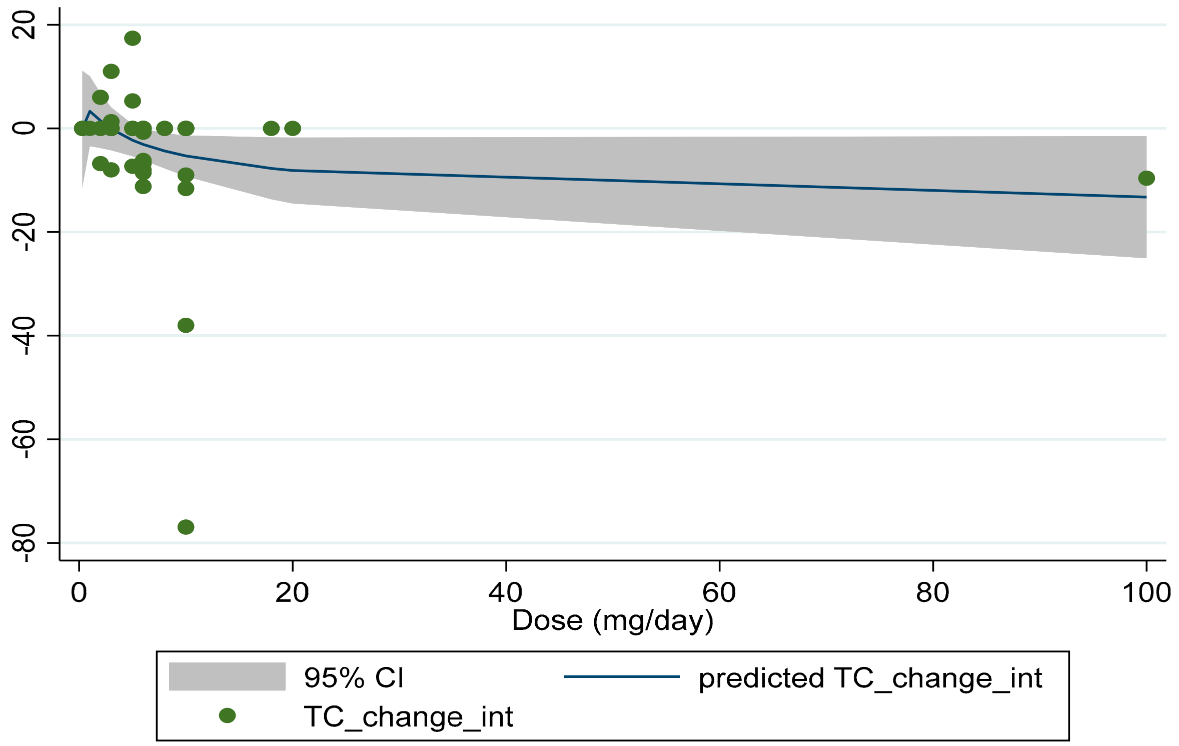

L) LDL-C

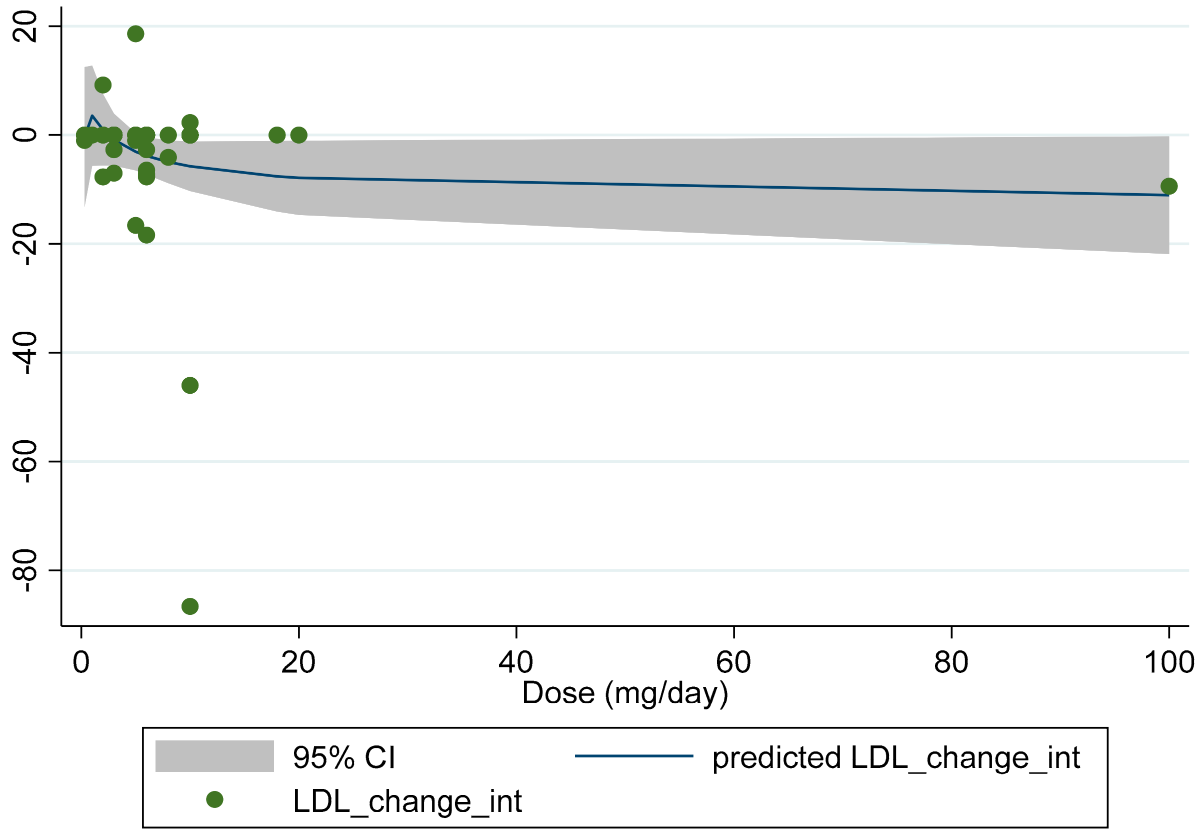

Figure S2. *Cont.*

M) HDL-C

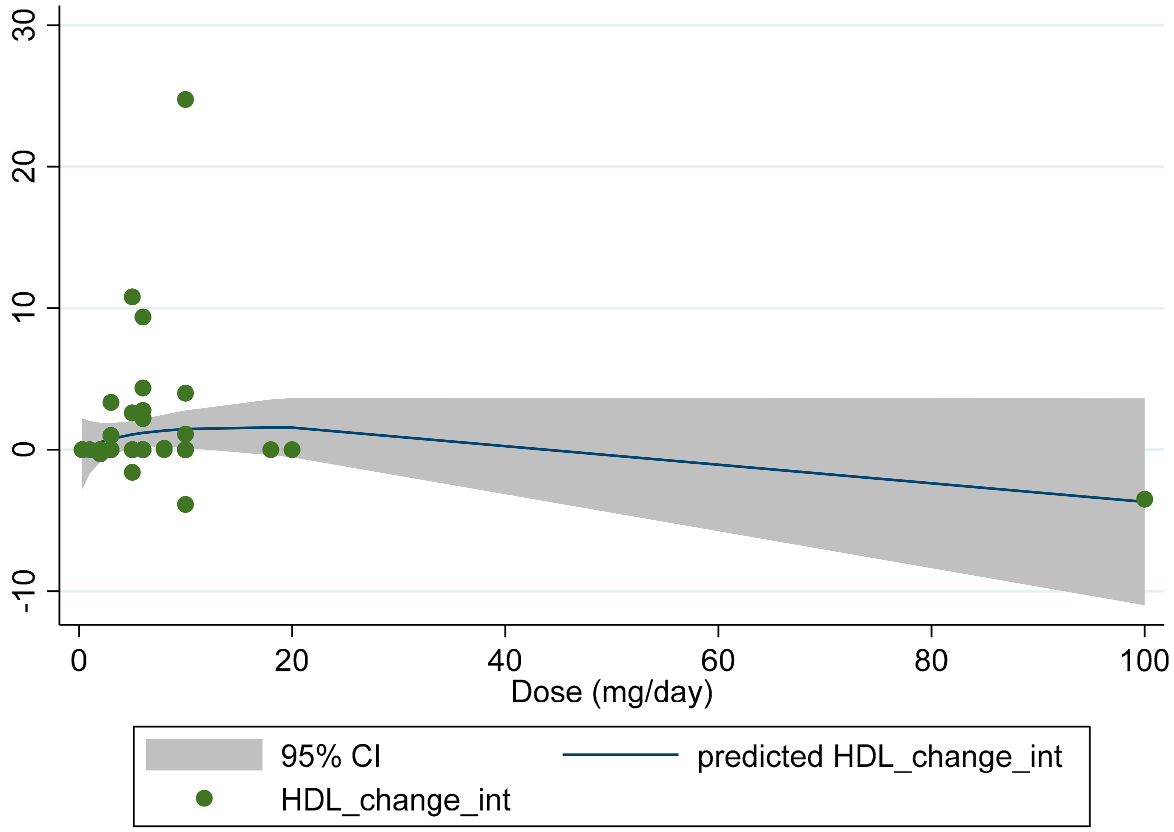

N) SBP

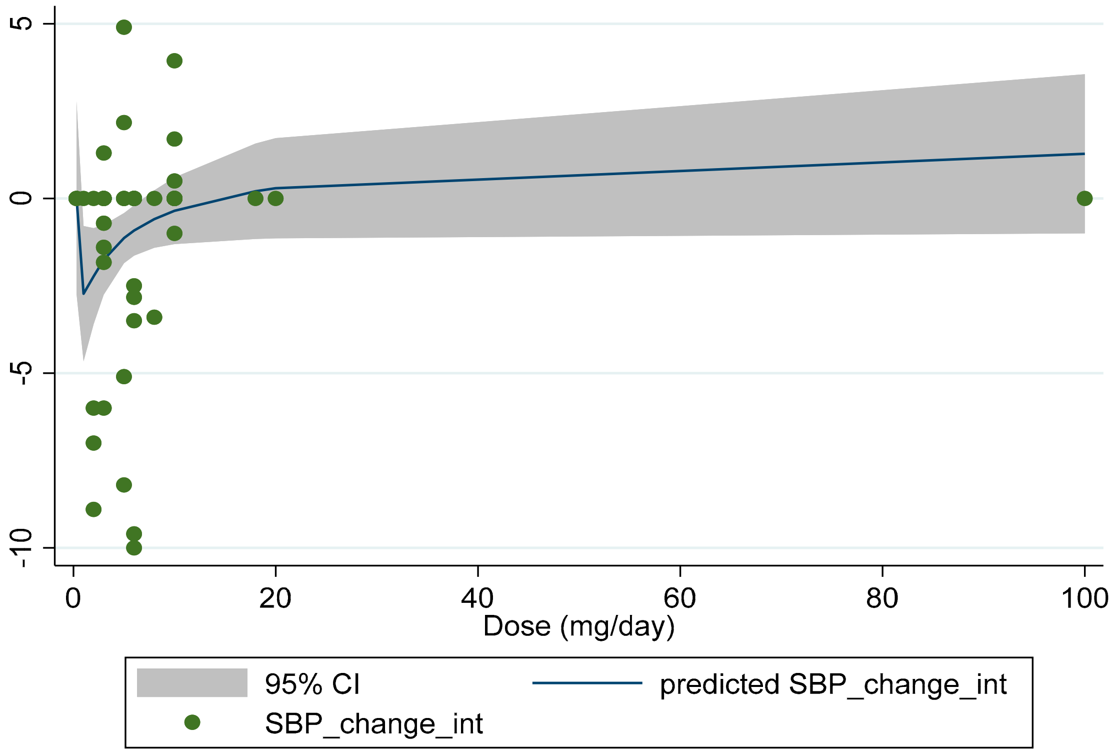

Figure S2. *Cont.*

O) DBP

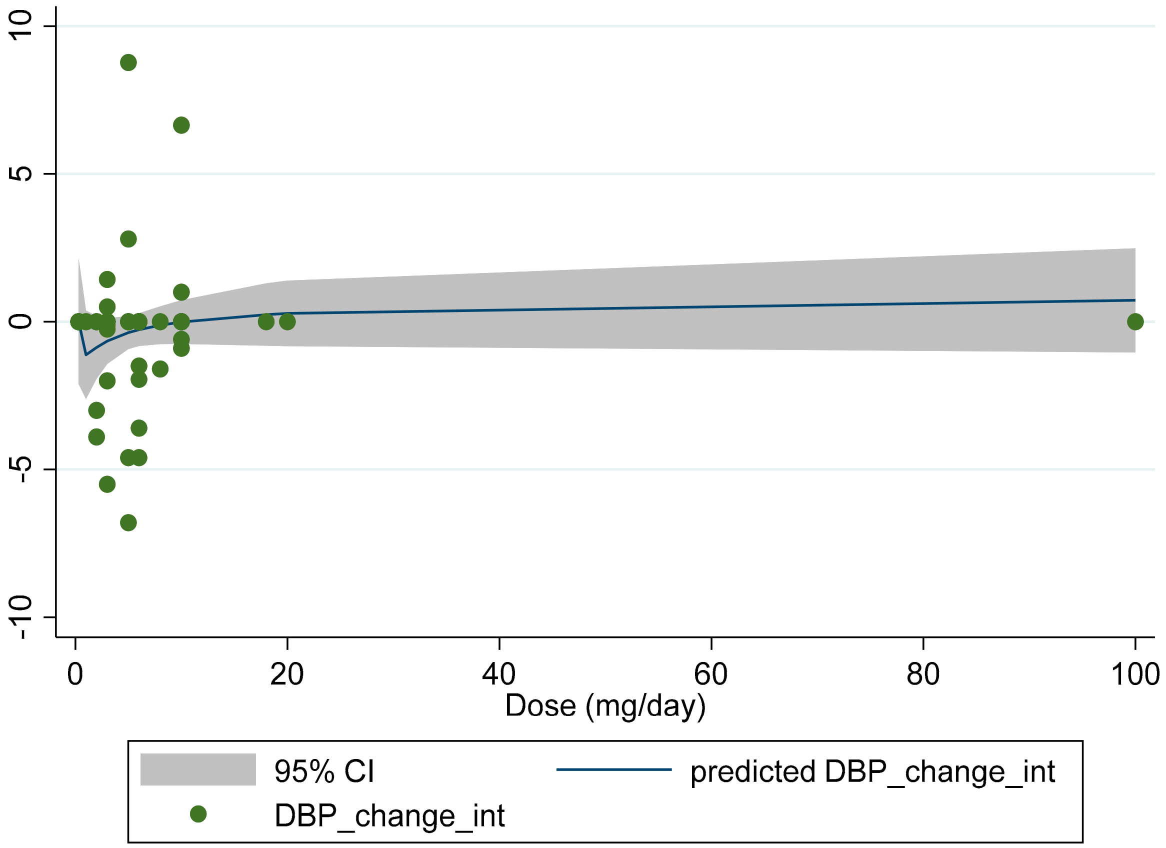

P) MDA

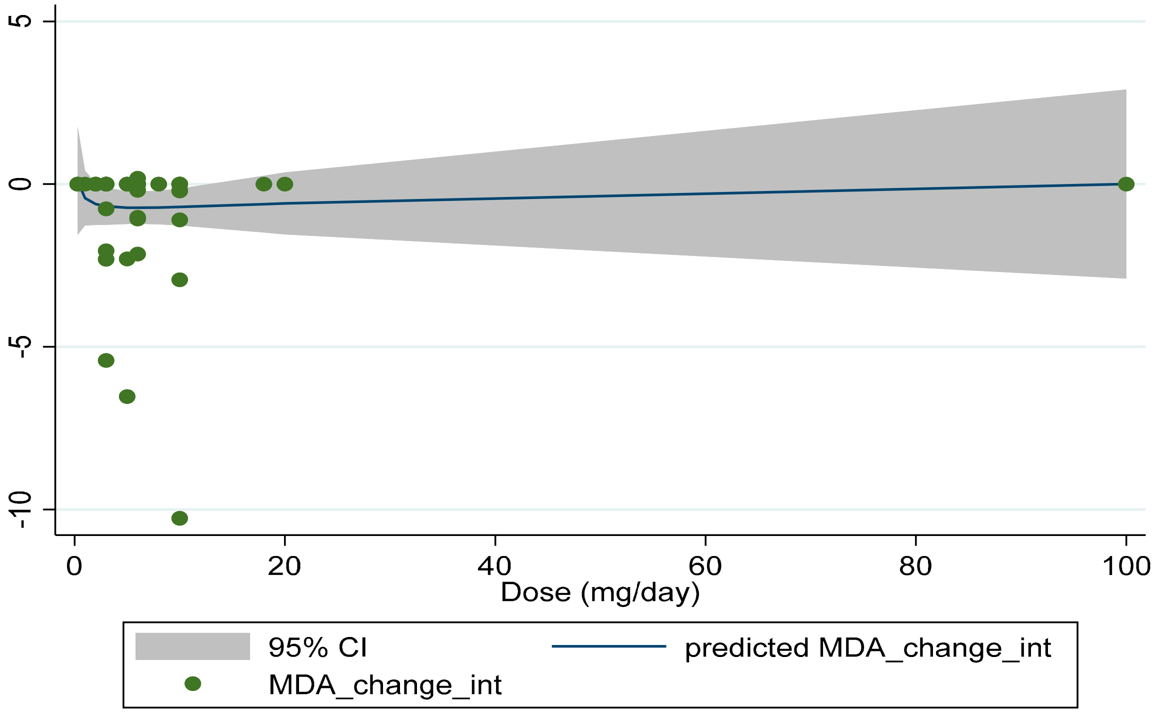

Figure S2. *Cont.*

Q) TAC

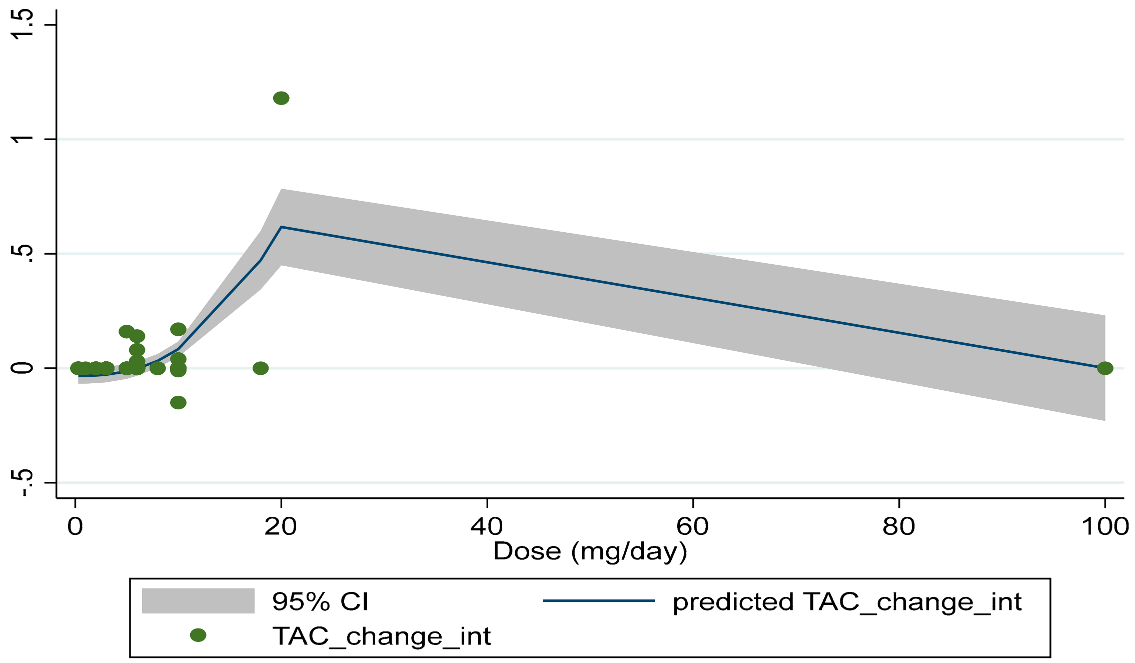

R) CRP

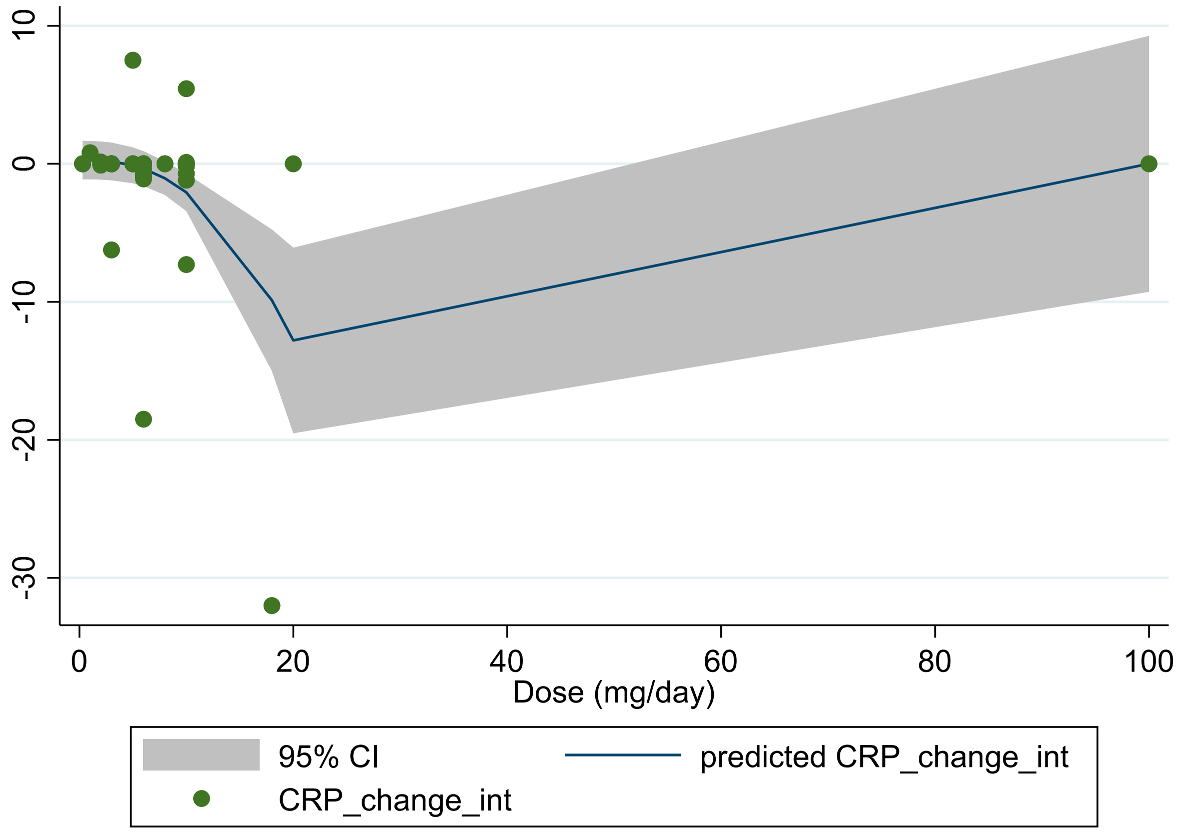

Figure S2. *Cont.*

S) IL-6

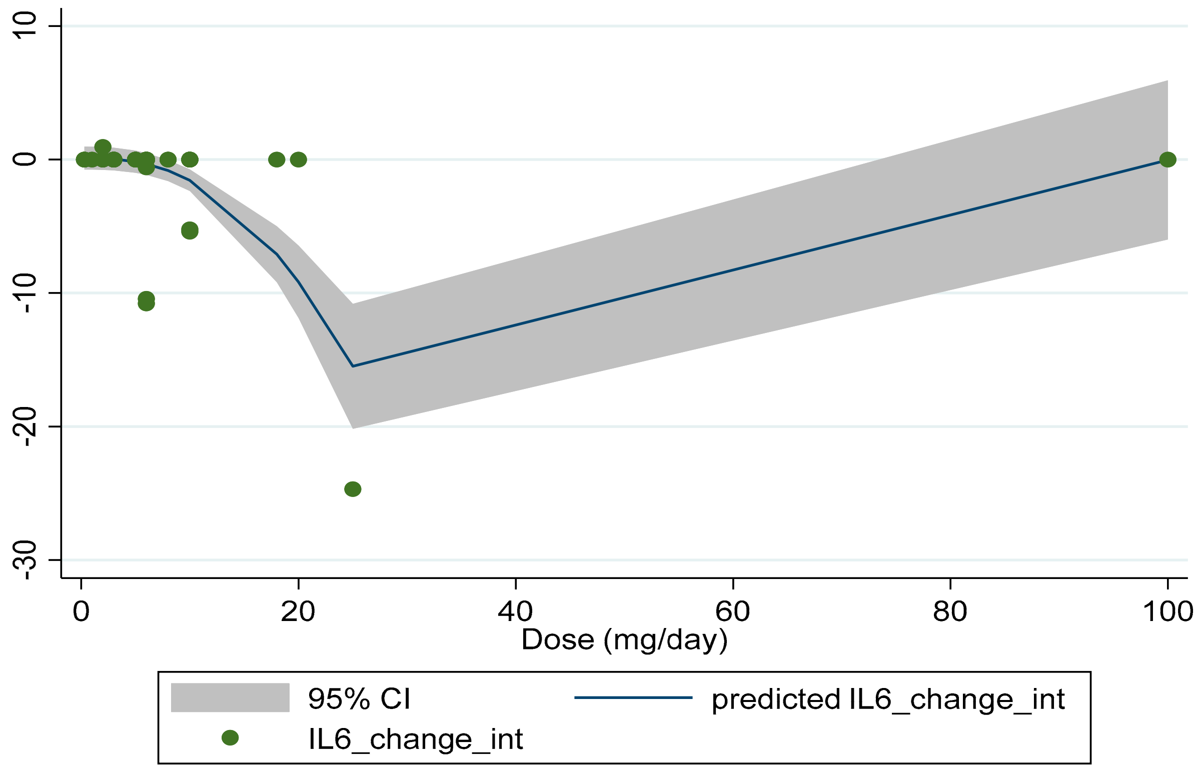

T) TNF-α

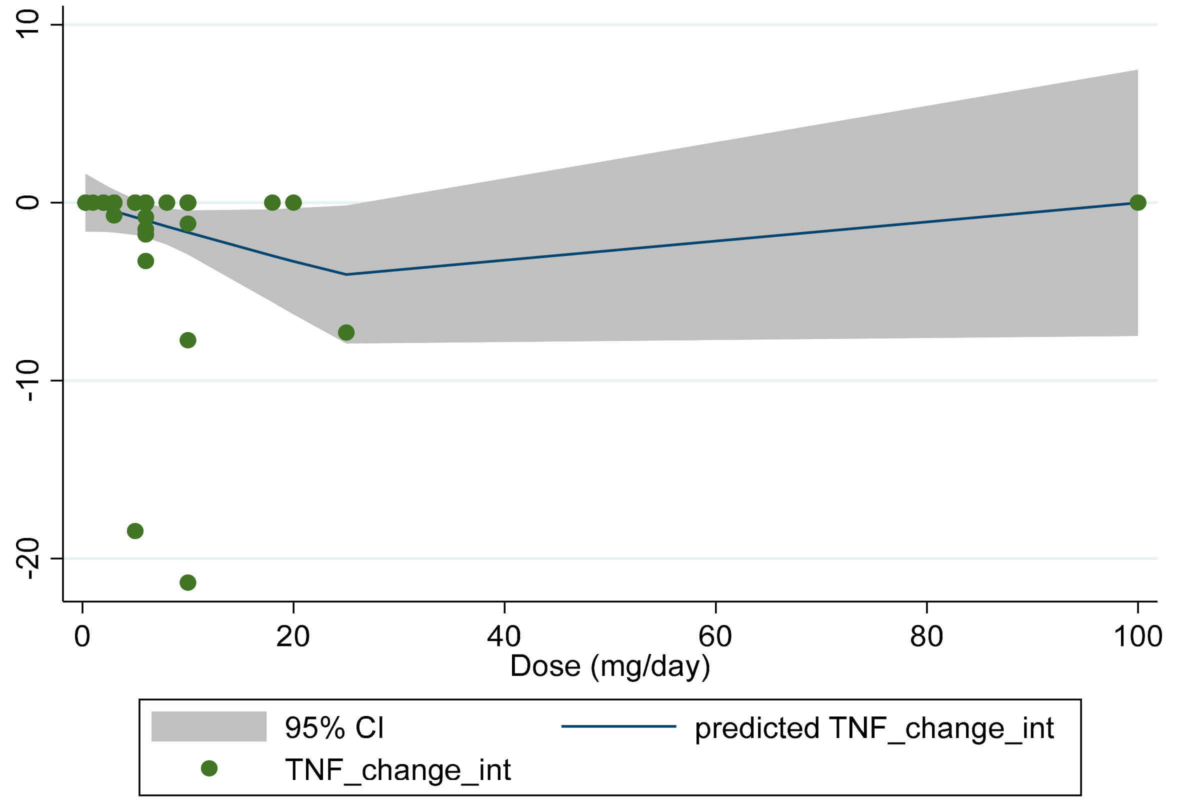

Figure S2. *Cont.*

U) AST

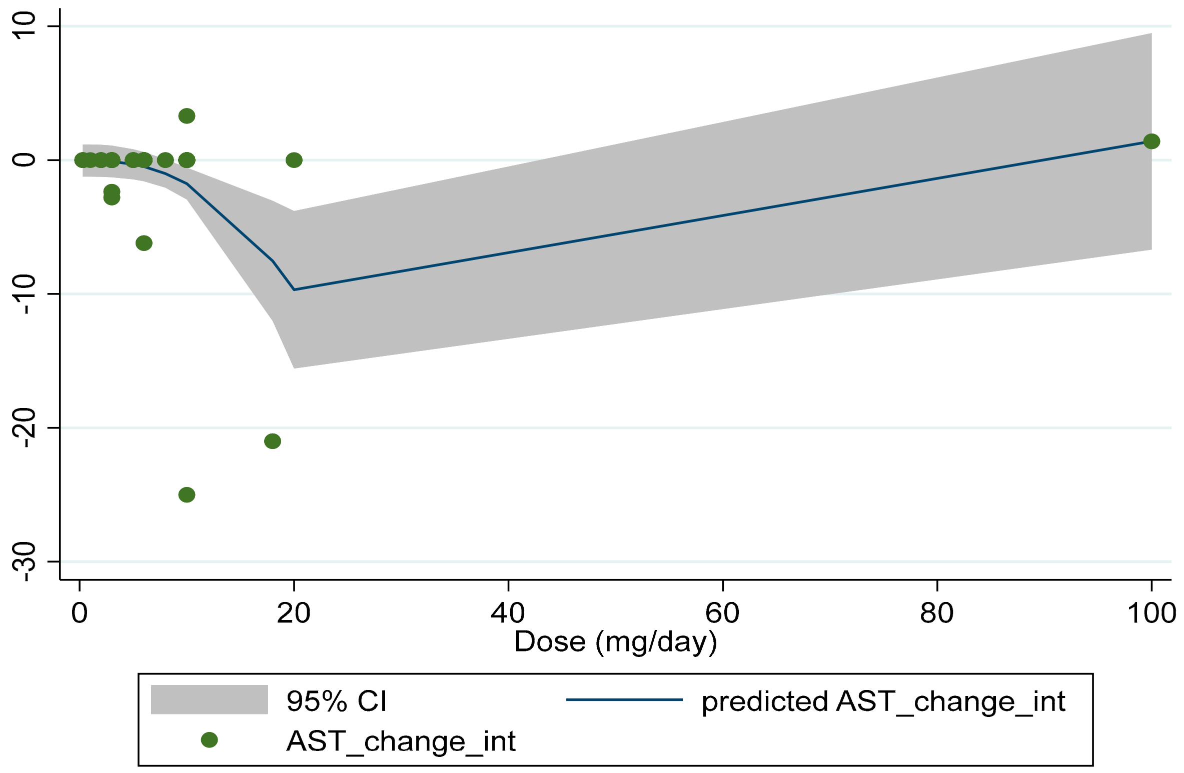

V) ALT

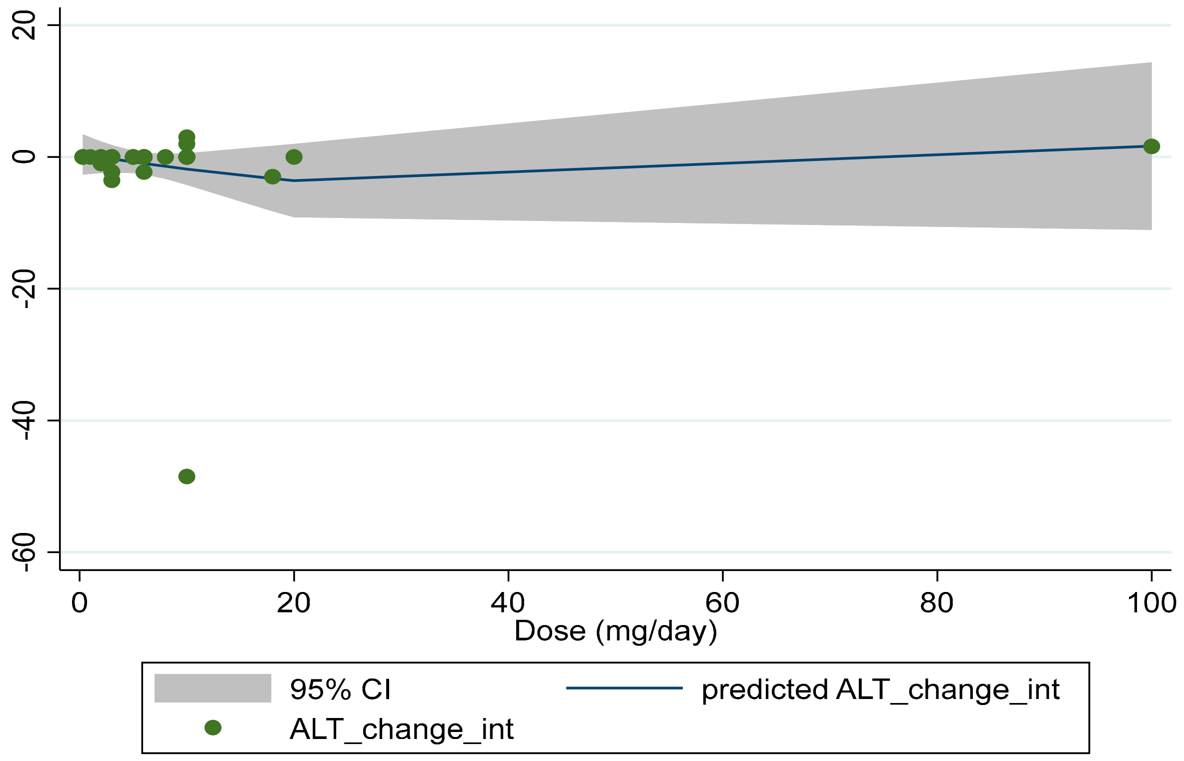

Figure S2. *Cont.*

W) GGT

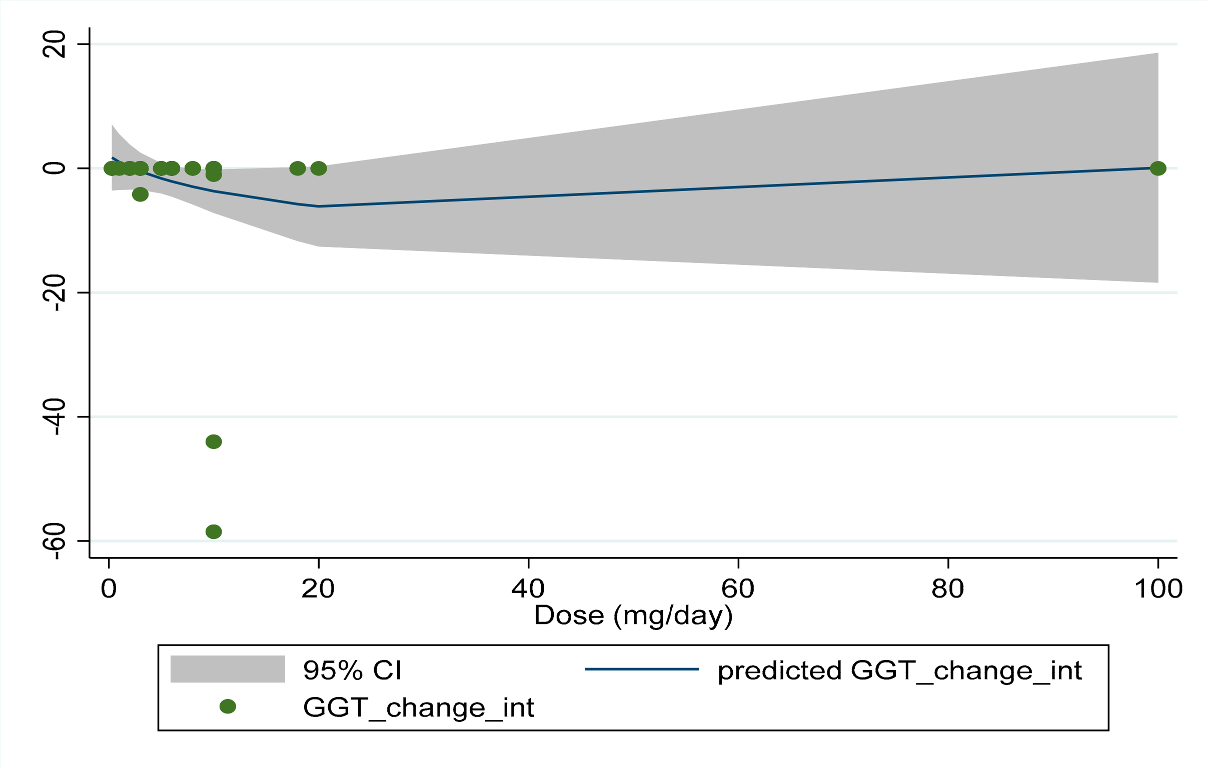

Figure S2. Non-linear dose-response association between dose (mg/day) of melatonin supplementation and mean changes in cardiometabolic risk factors (CMRFs), including **(A)** BW (kg), **(B)** BMI (kg/m²), **(C)** WC (cm), **(D)** HC (cm), **(E)** BFP (%), **(F)** FBG (mg/dL), **(G)** HbA1c (%), **(H)** FI (μIU/mL), **(I)** HOMA-IR, **(J)** TG (mg/dL), **(K)** TC (mg/dL), **(L)** LDL-C (mg/dL), **(M)** HDL-C (mg/dL), **(N)** SBP (mmHg), **(O)** DBP (mmHg), **(P)** MDA (μmol/L), **(Q)** TAC (mmol/L), **(R)** CRP (mg/L), **(S)** IL-6 (pg/mL), **(T)** TNF-*α* (pg/mL), **(U)** AST (IU/L), **(V)** ALT (IU/L), and **(W)** GGT (IU/L).

A) BW

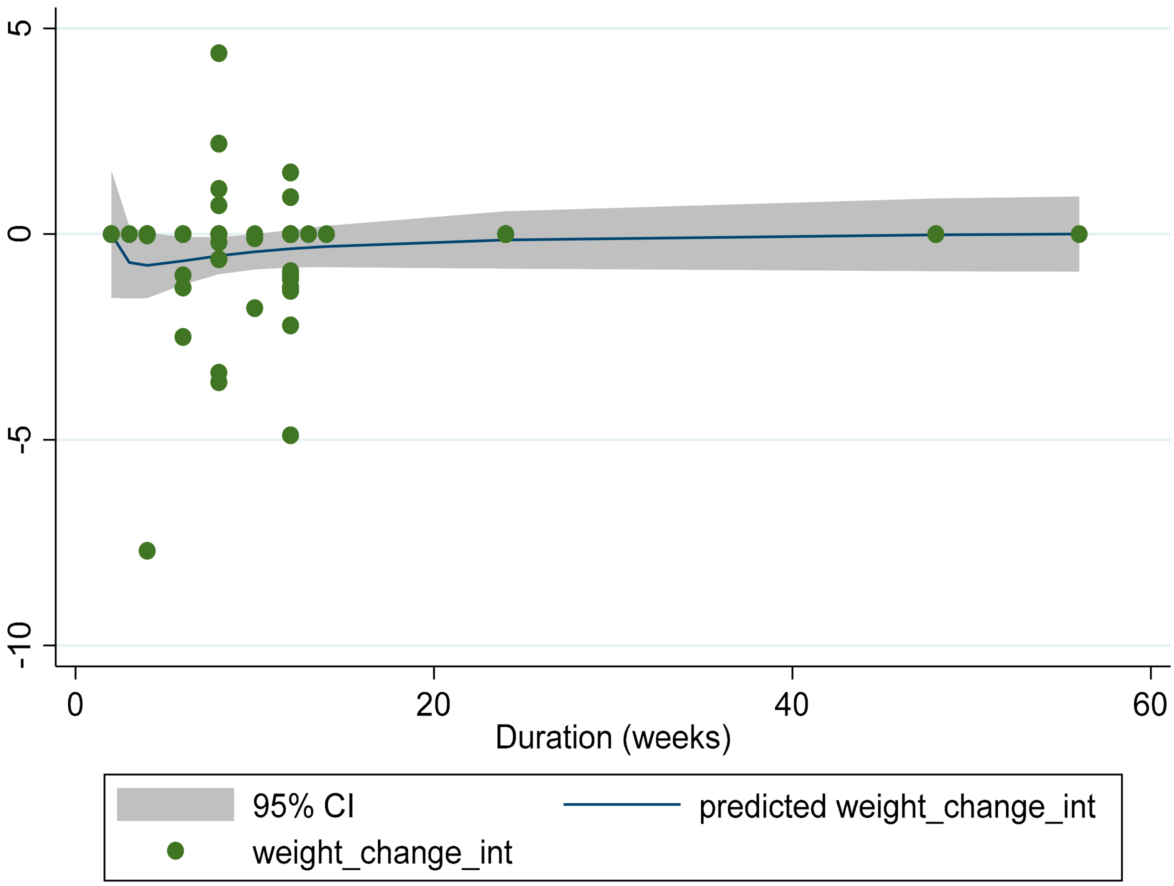

B) BMI

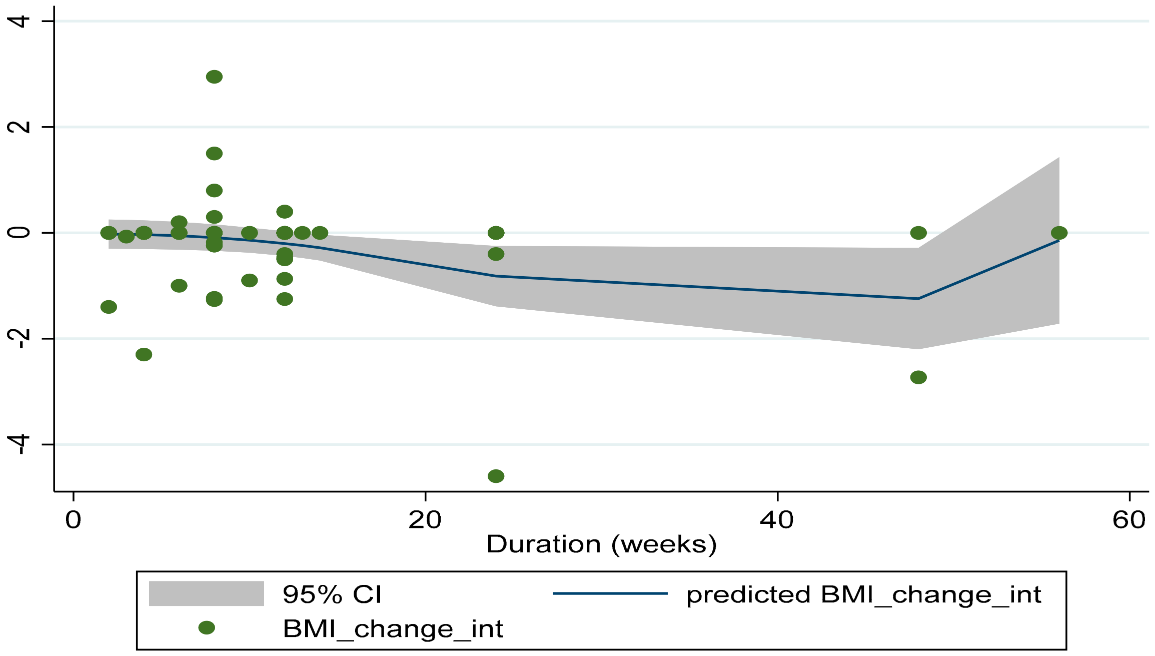

Figure S3. *Cont.*

C) WC

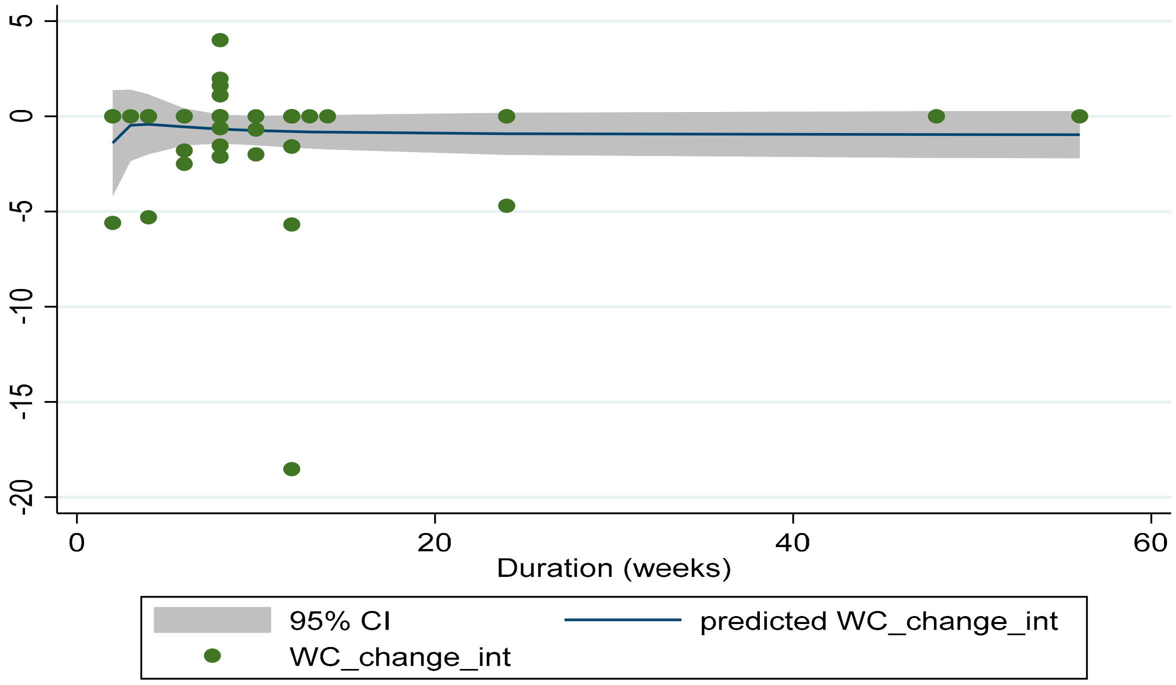

D) HC

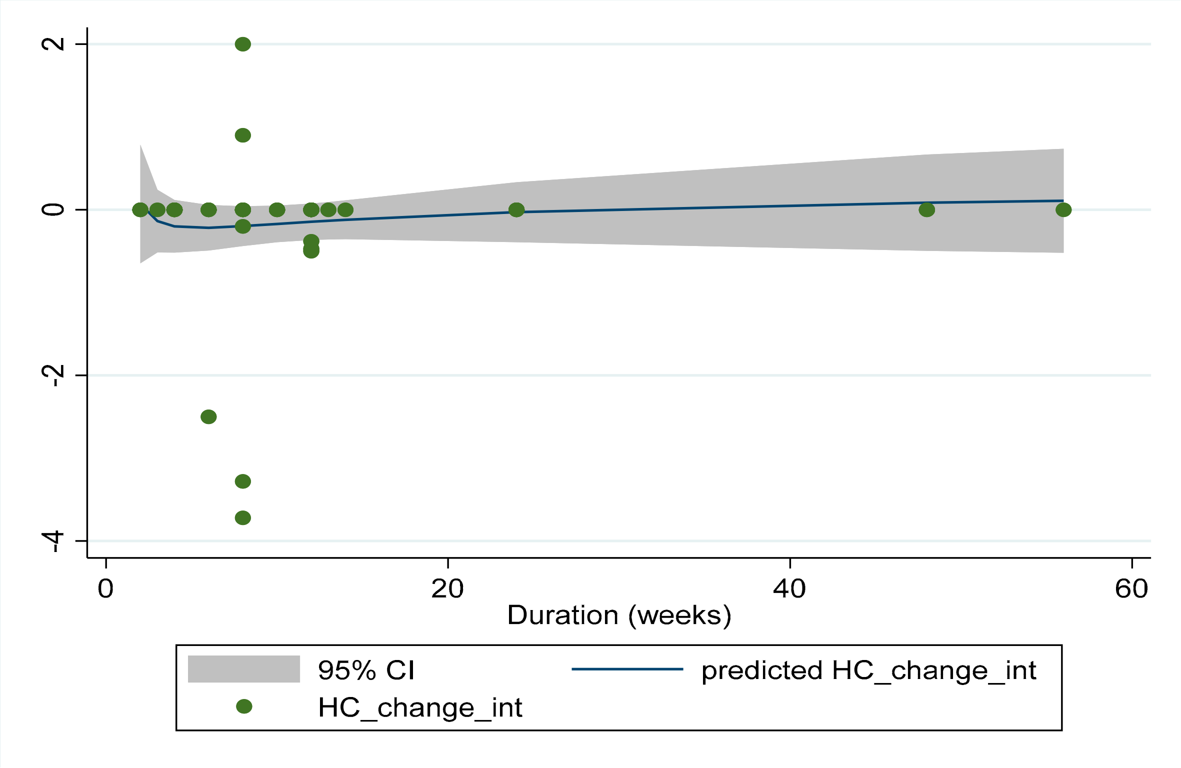

Figure S3. *Cont.*

E) BFP

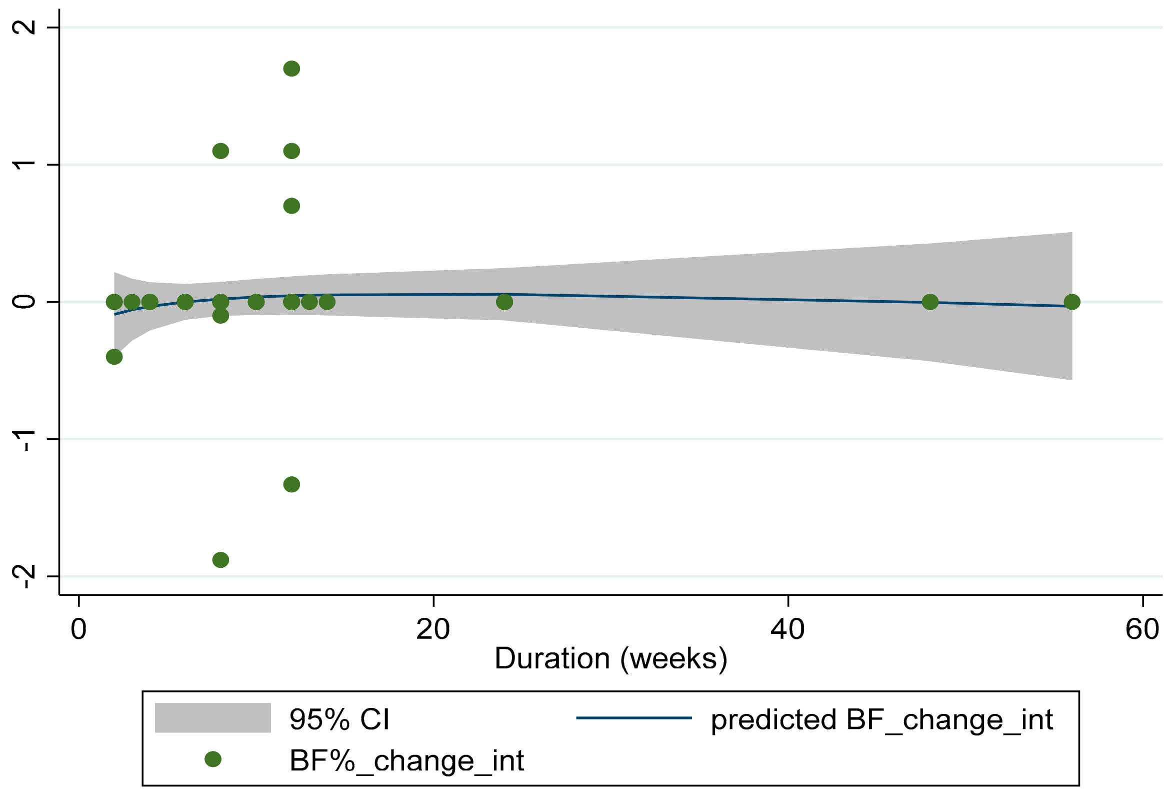

F) FBG

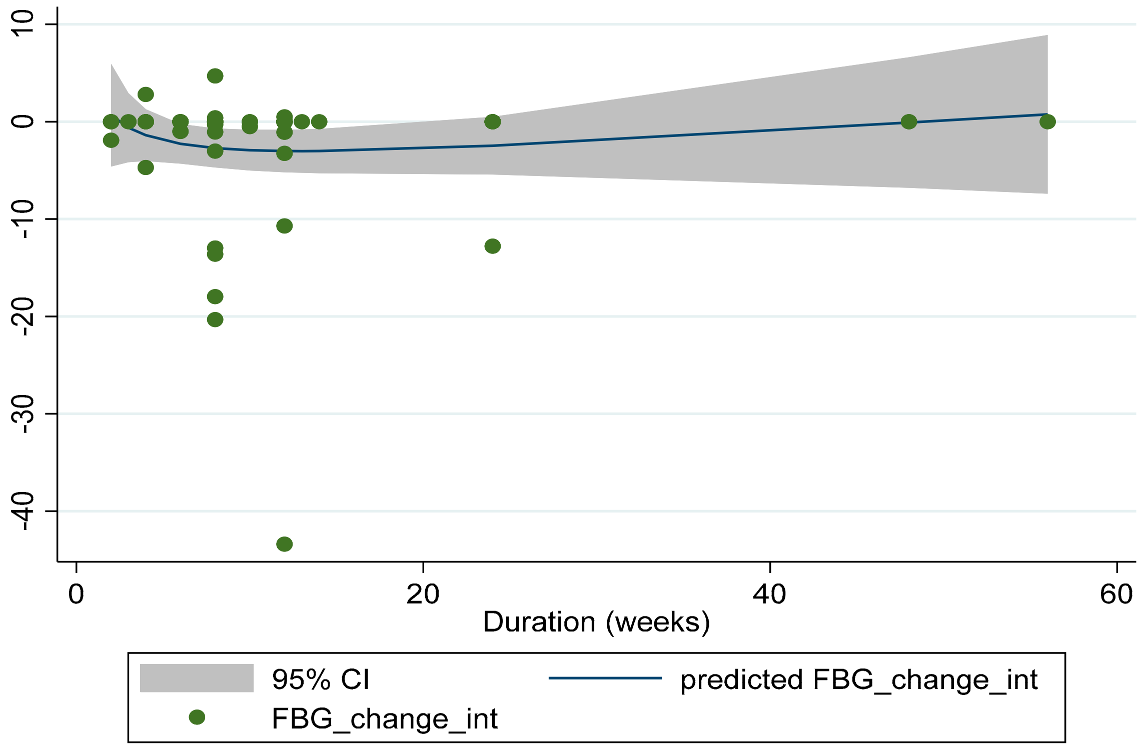

Figure S3. *Cont.*

G) HbA1c

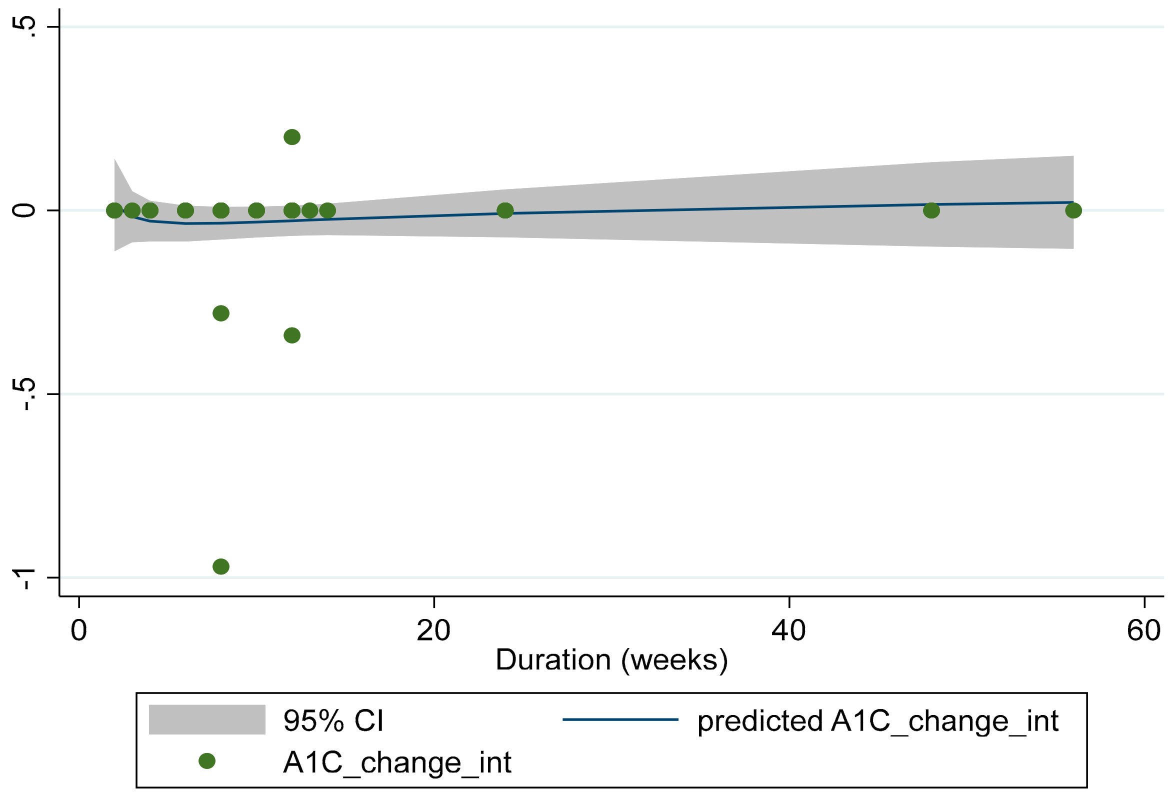

H) FI

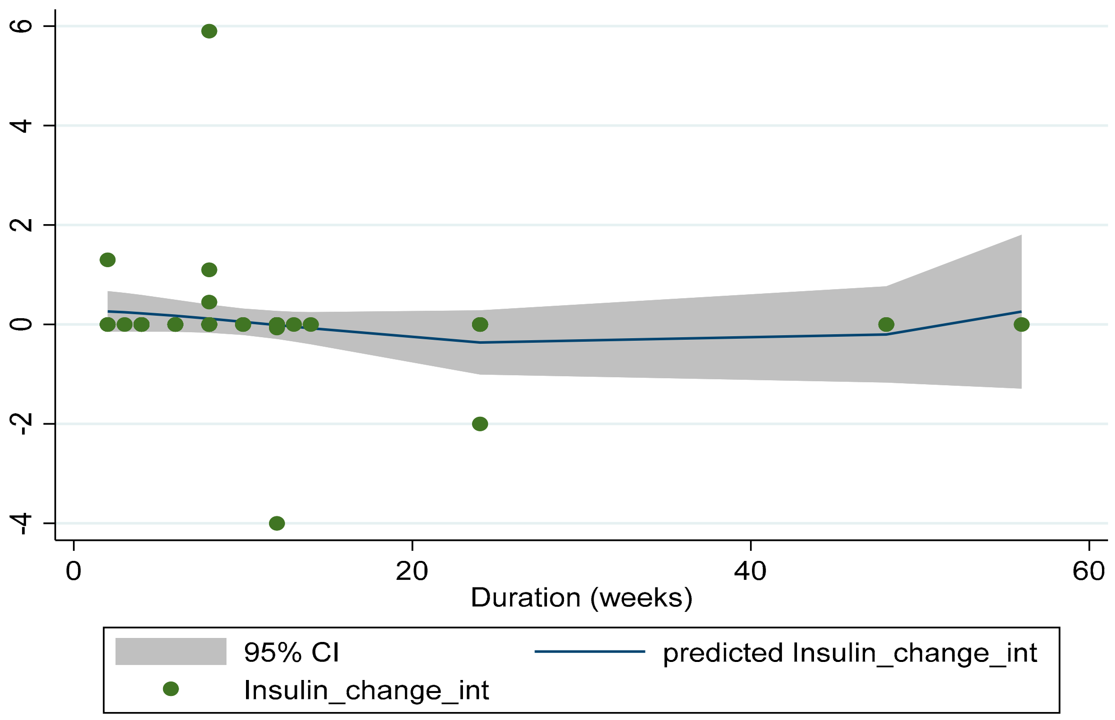

Figure S3. *Cont.*

I) HOMA-IR

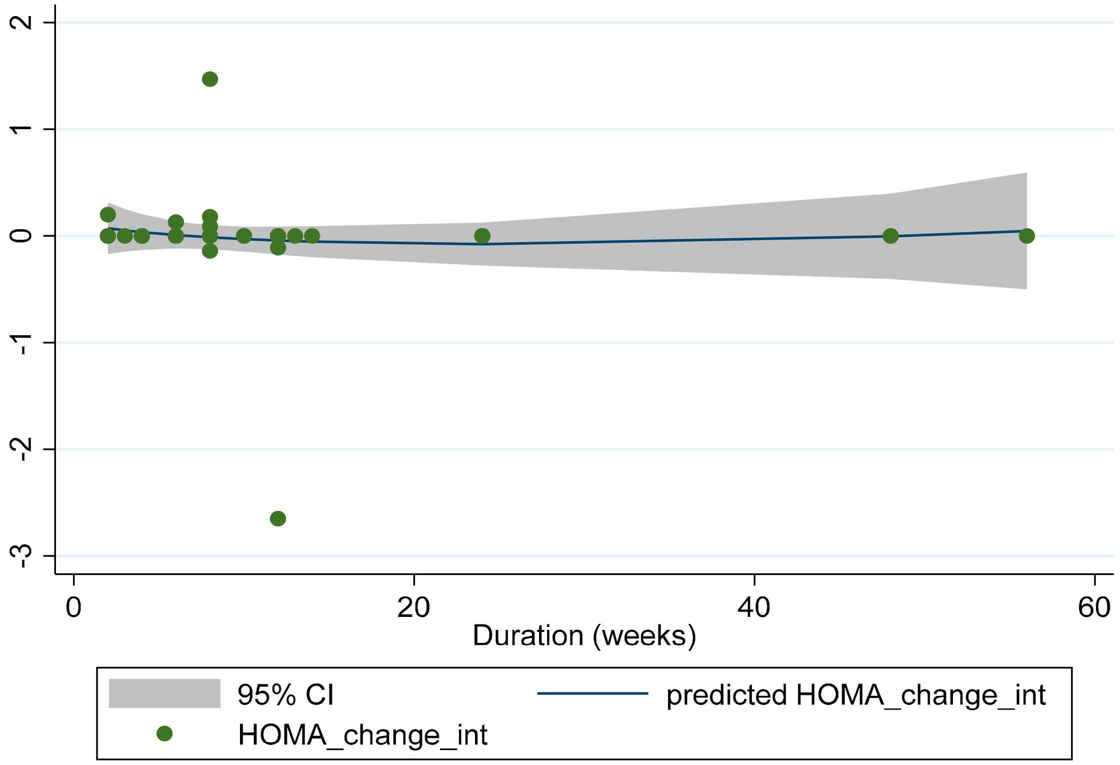

J) TG

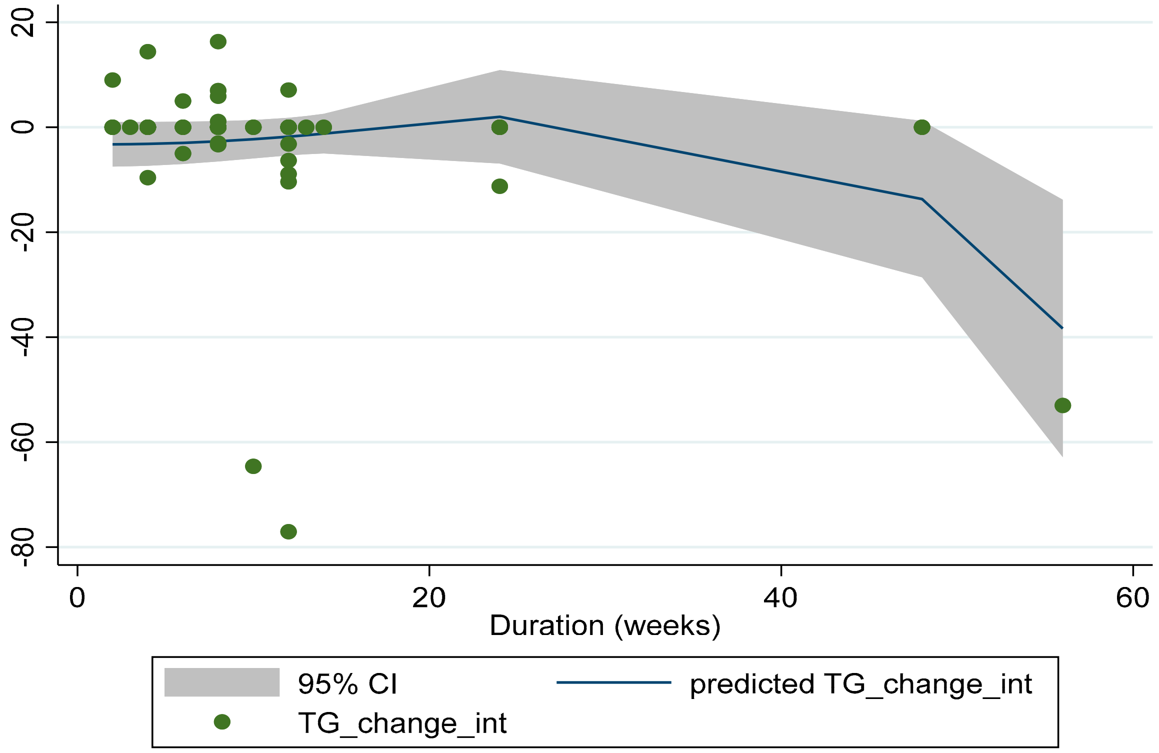

Figure S3. *Cont.*

K) TC

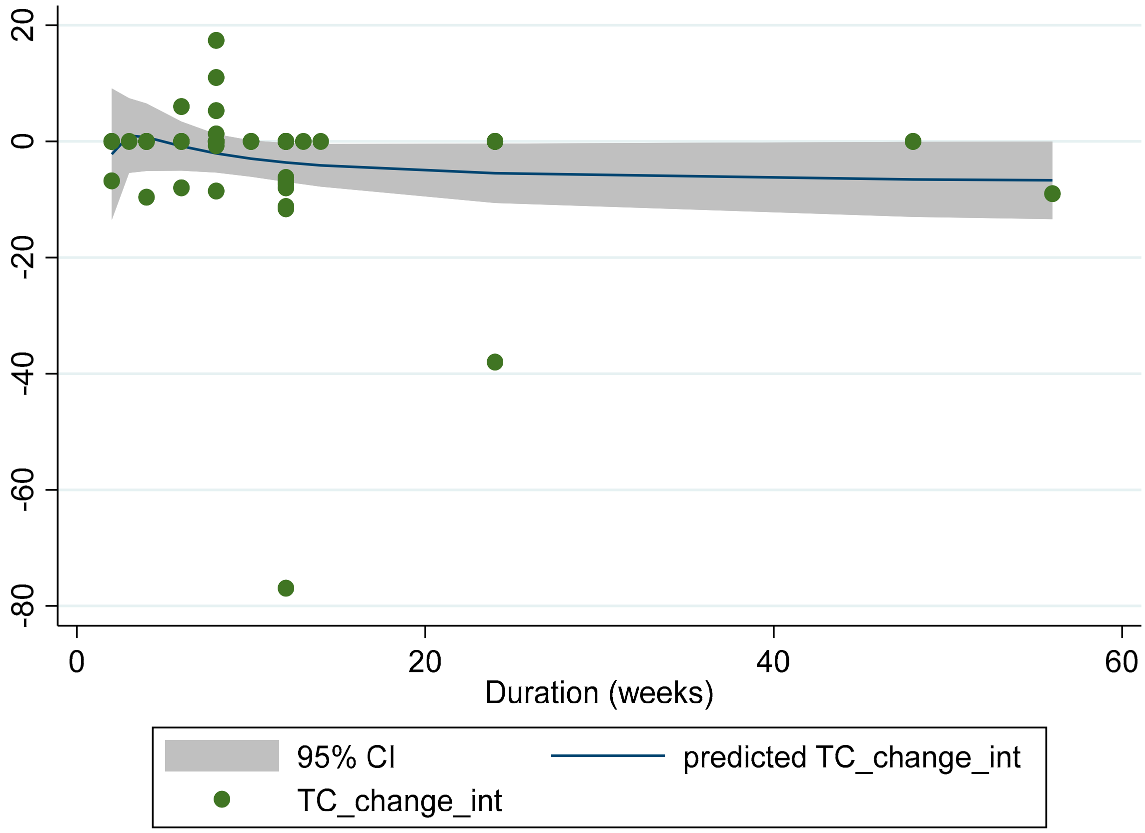

L) LDL-C

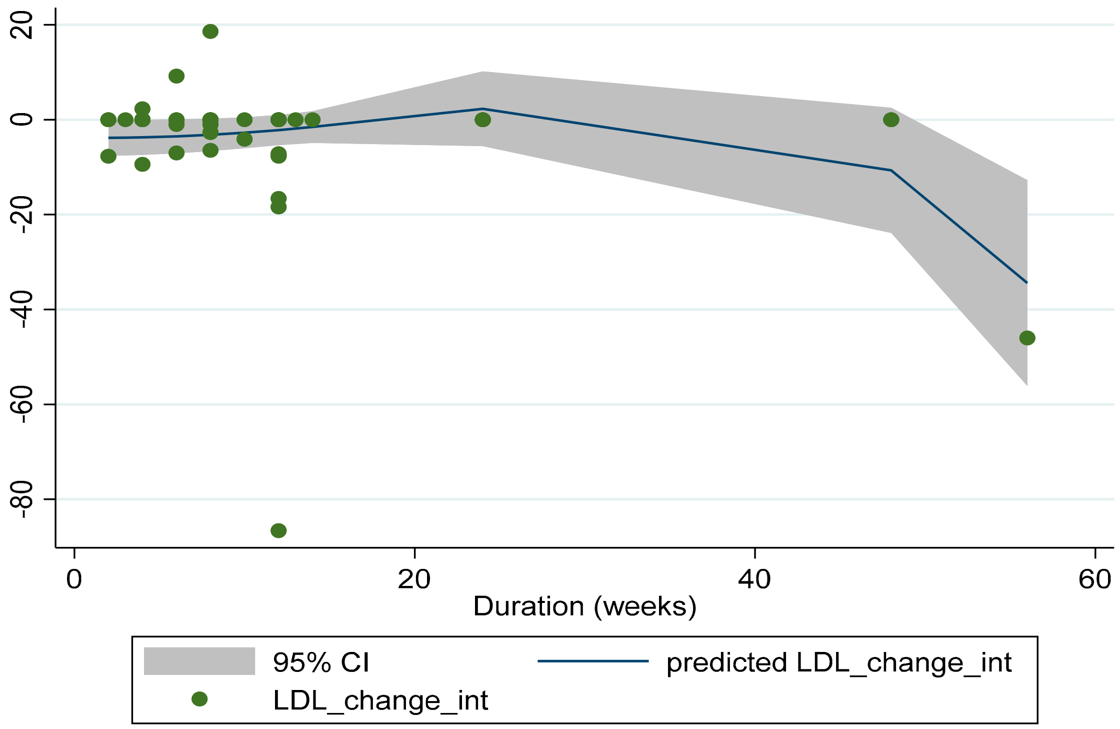

Figure S3. *Cont.*

M) HDL-C

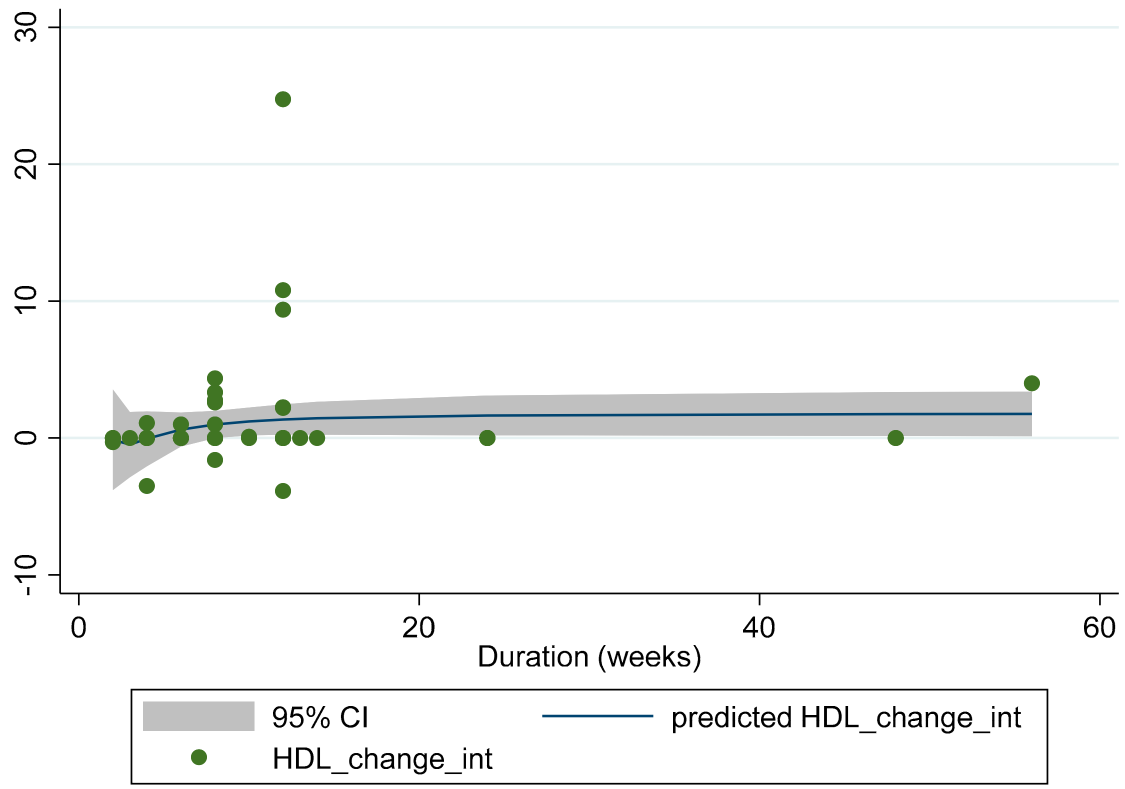

N) SBP

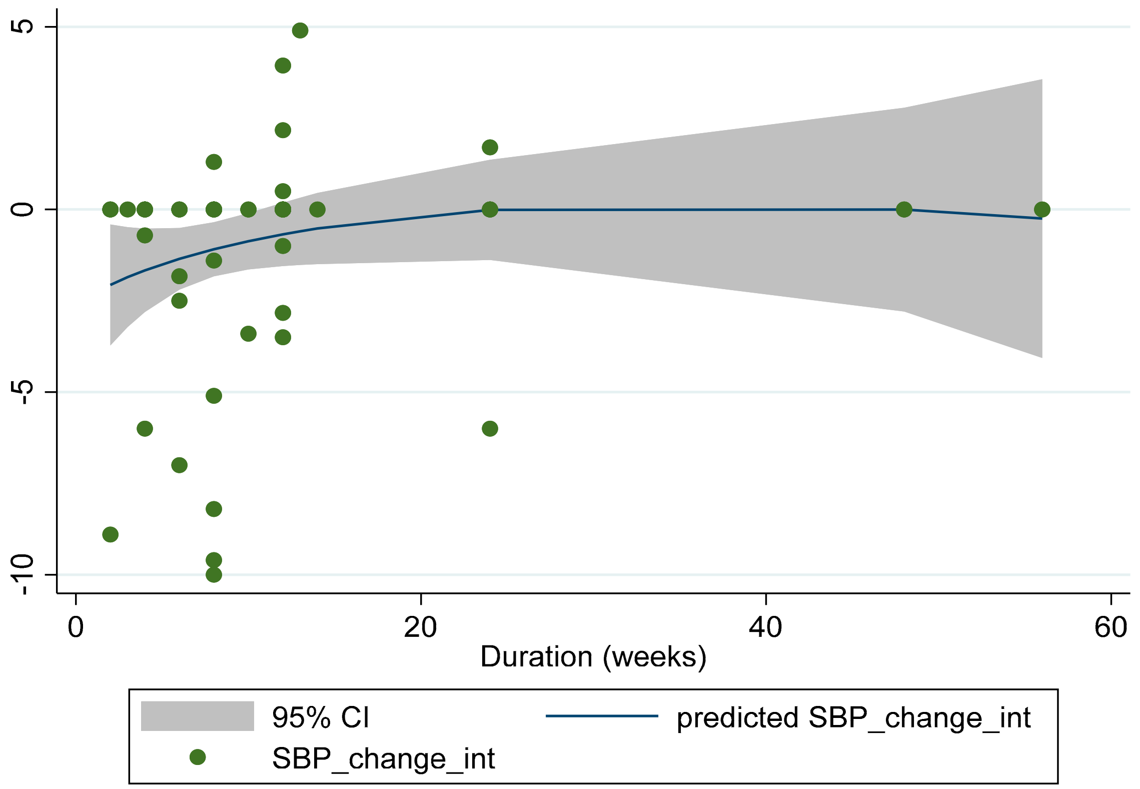

Figure S3. *Cont.*

O) DBP

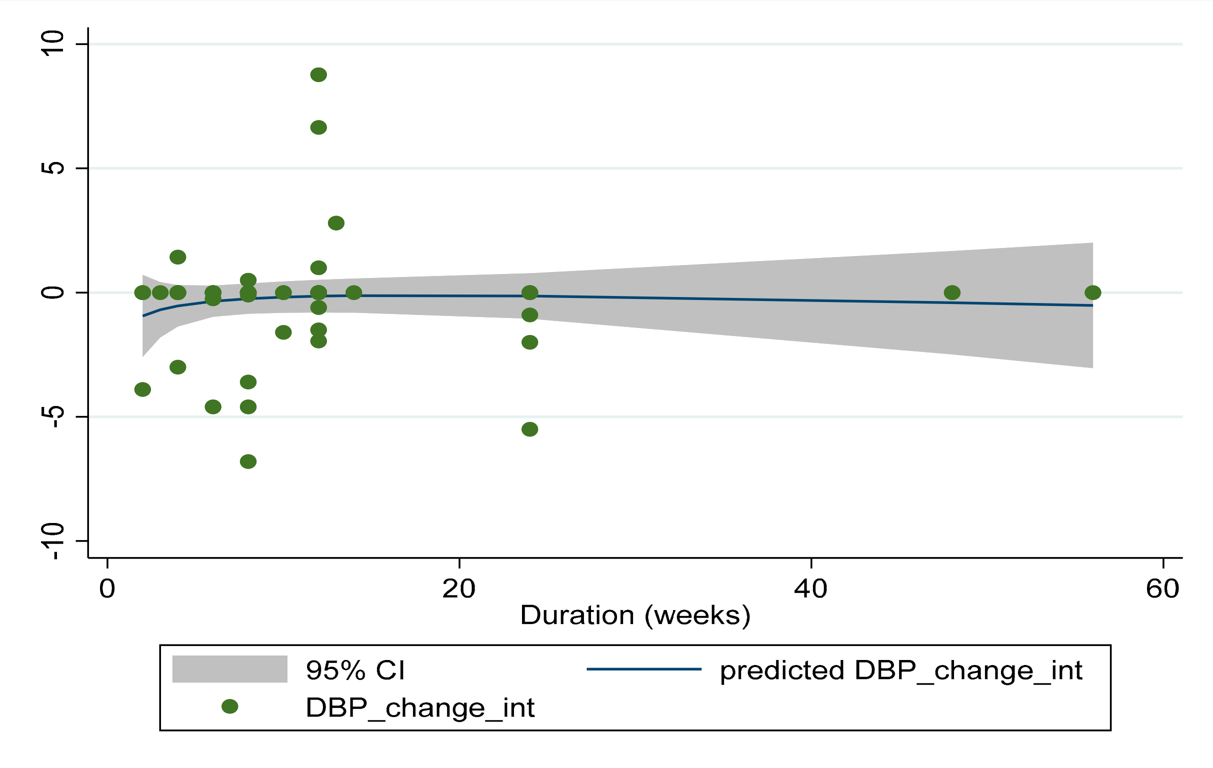

P) MDA

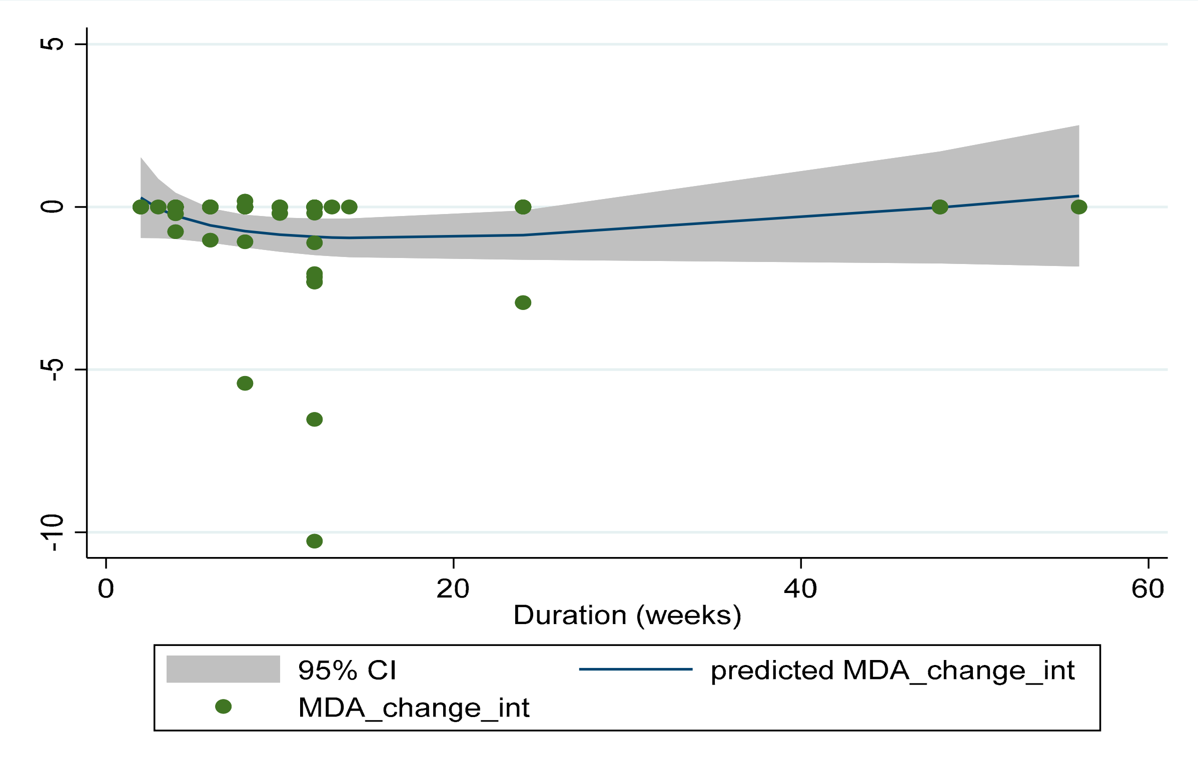

Figure S3. *Cont.*

Q) TAC

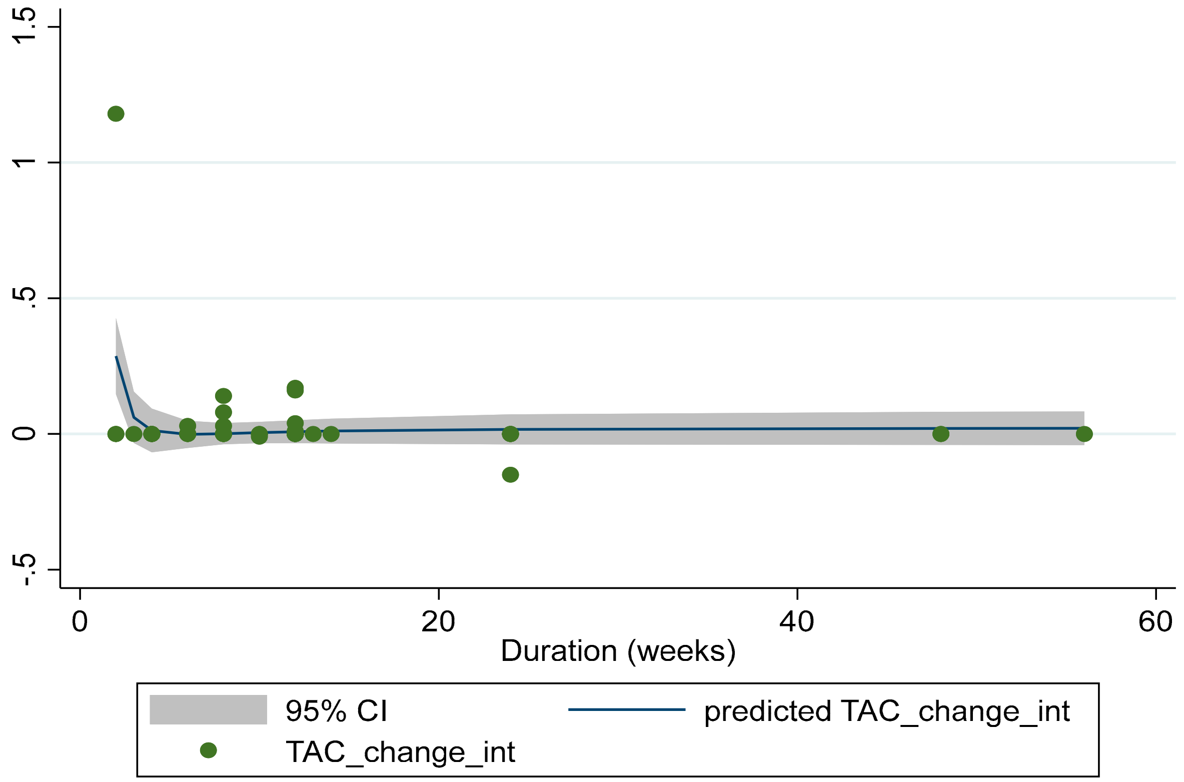

R) CRP

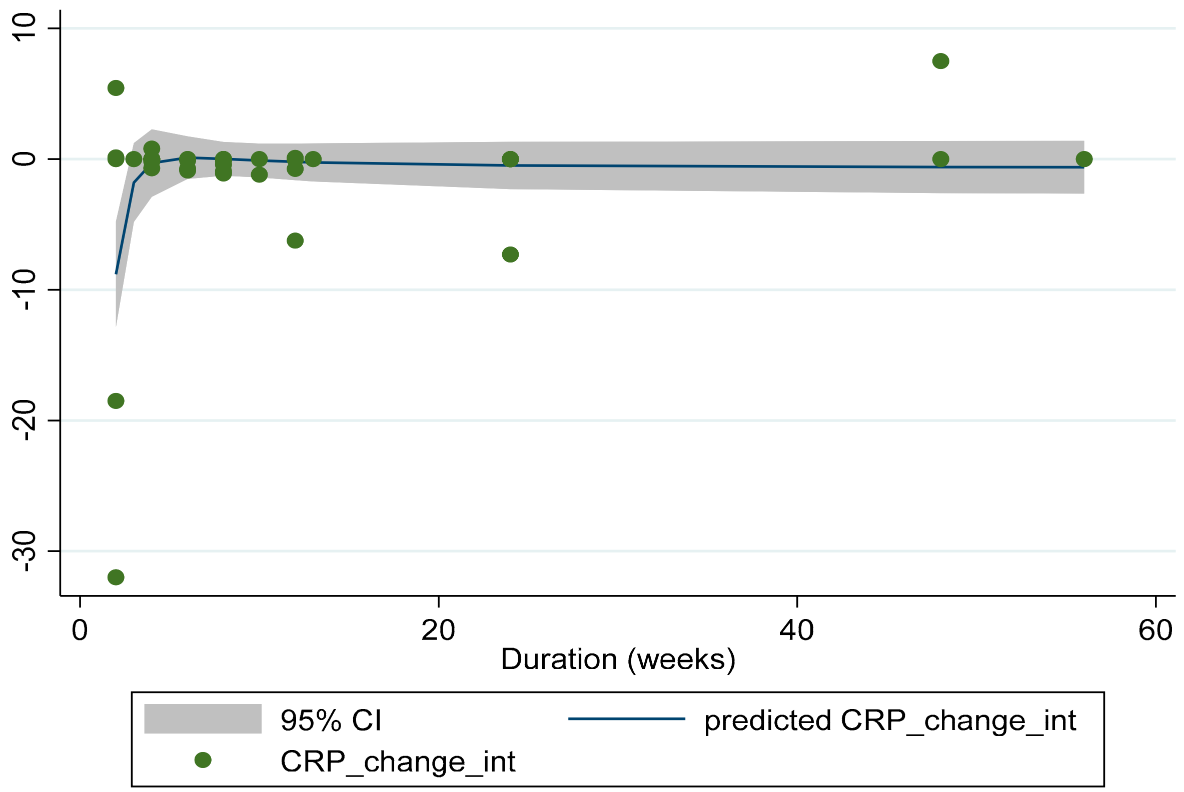

Figure S3. *Cont.*

S) **IL-6**

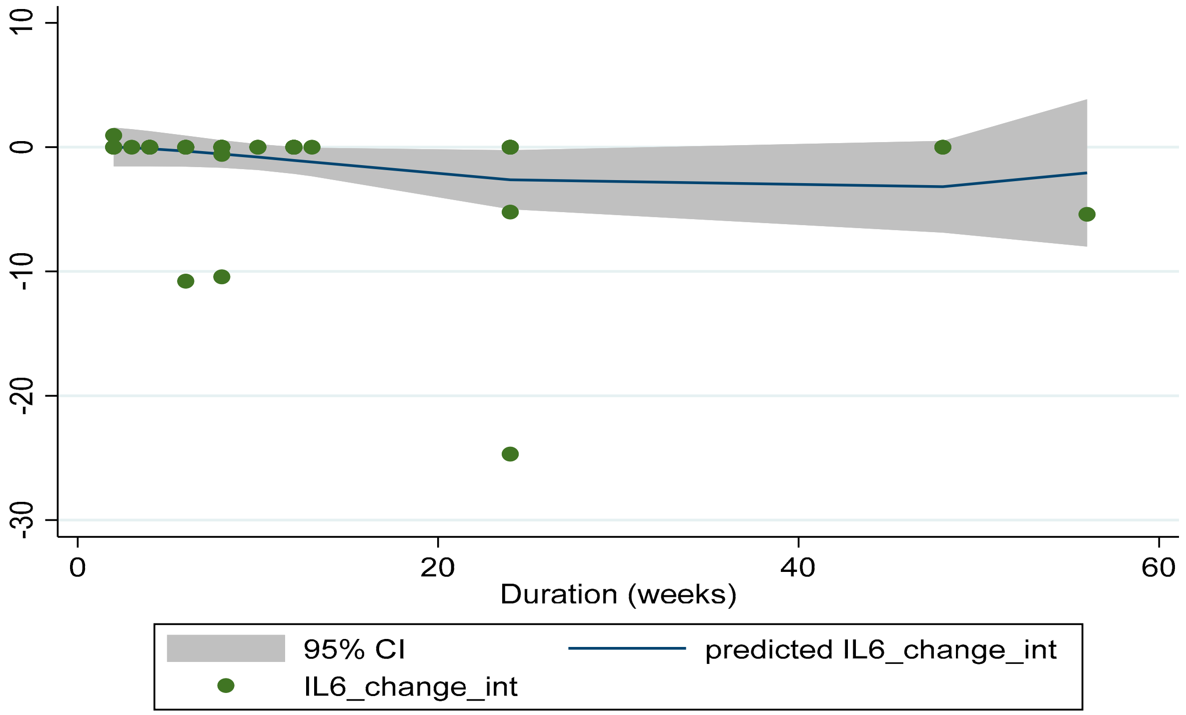

T) **TNF-α**

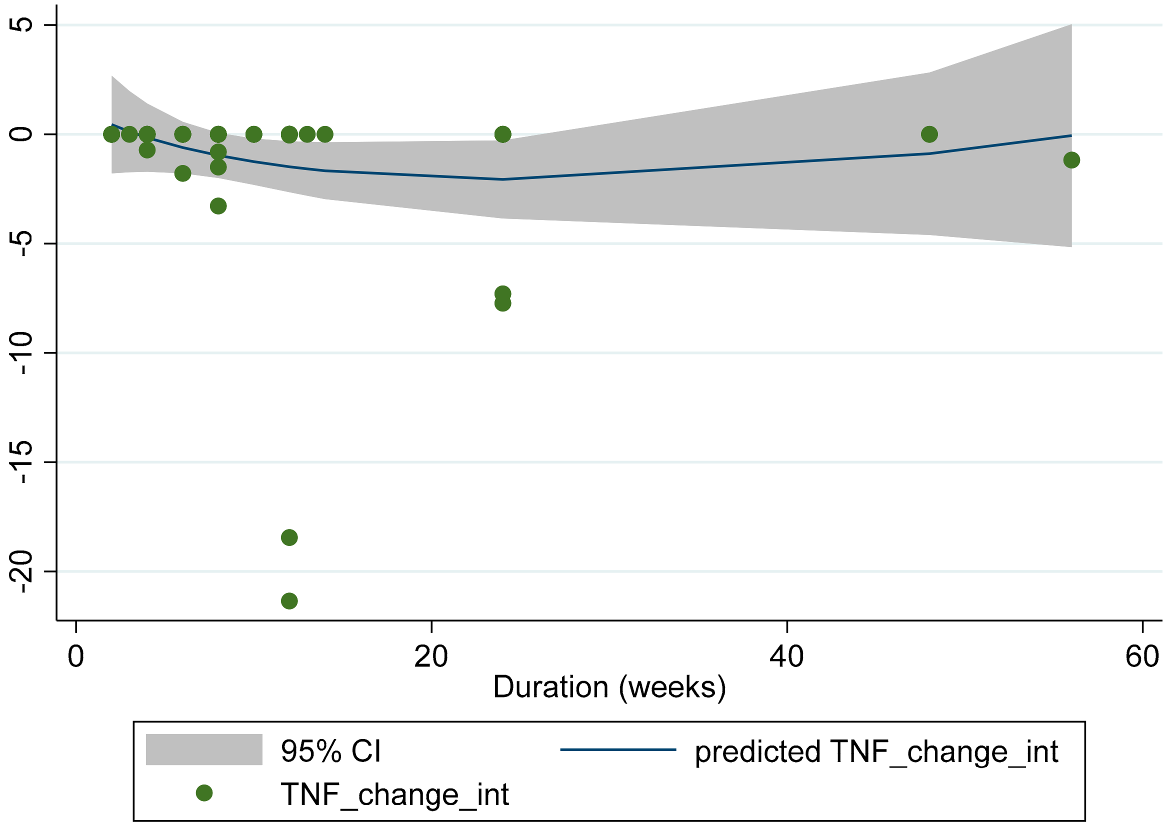

Figure S3. *Cont.*

U) AST

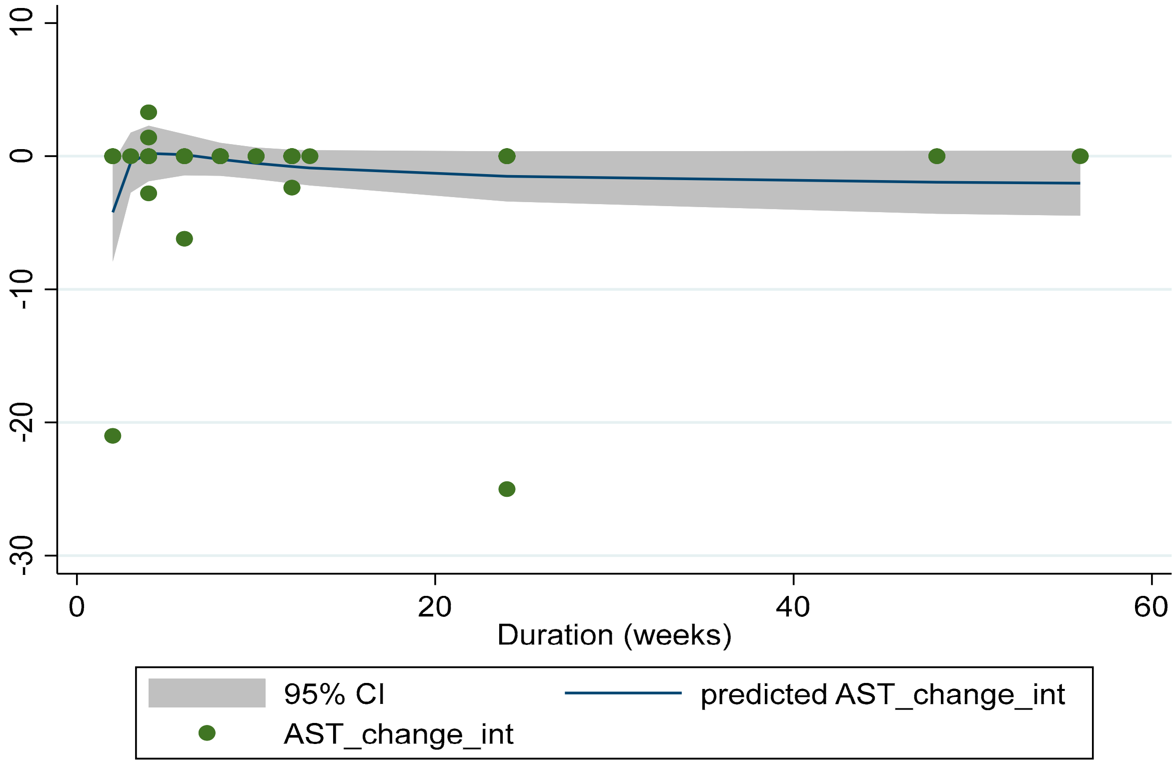

V) ALT

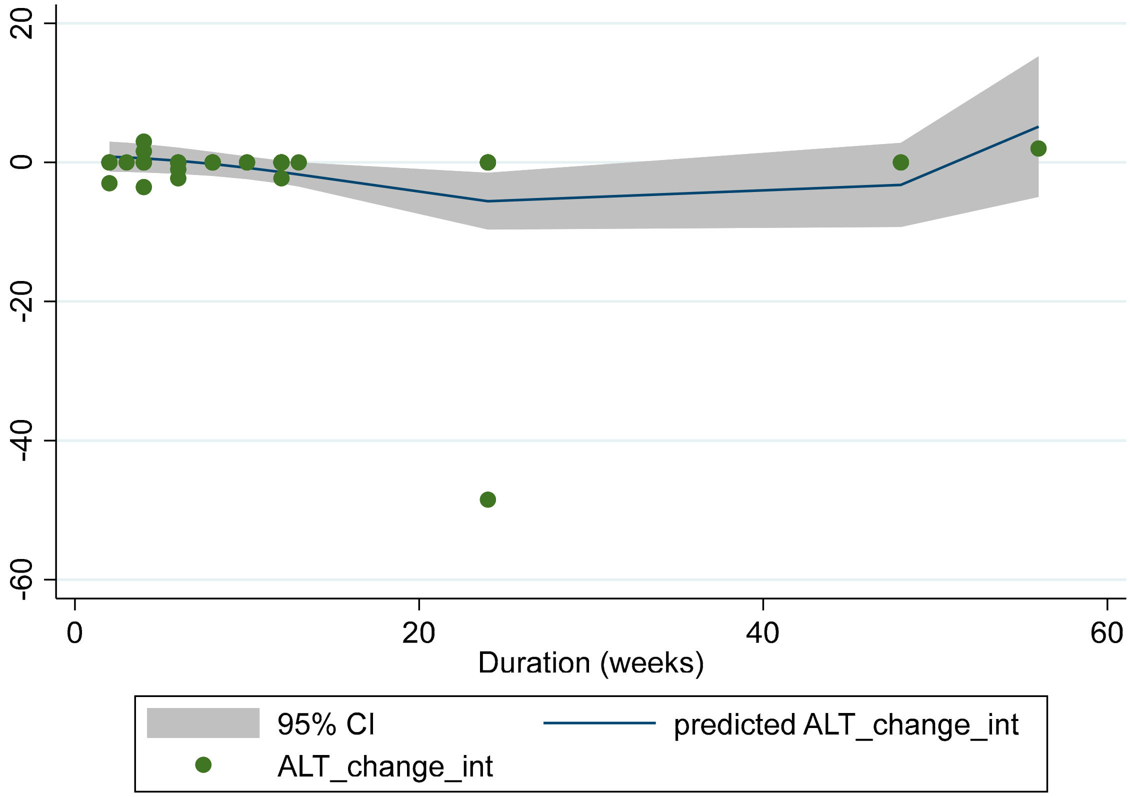

Figure S3. *Cont.*

W) GGT

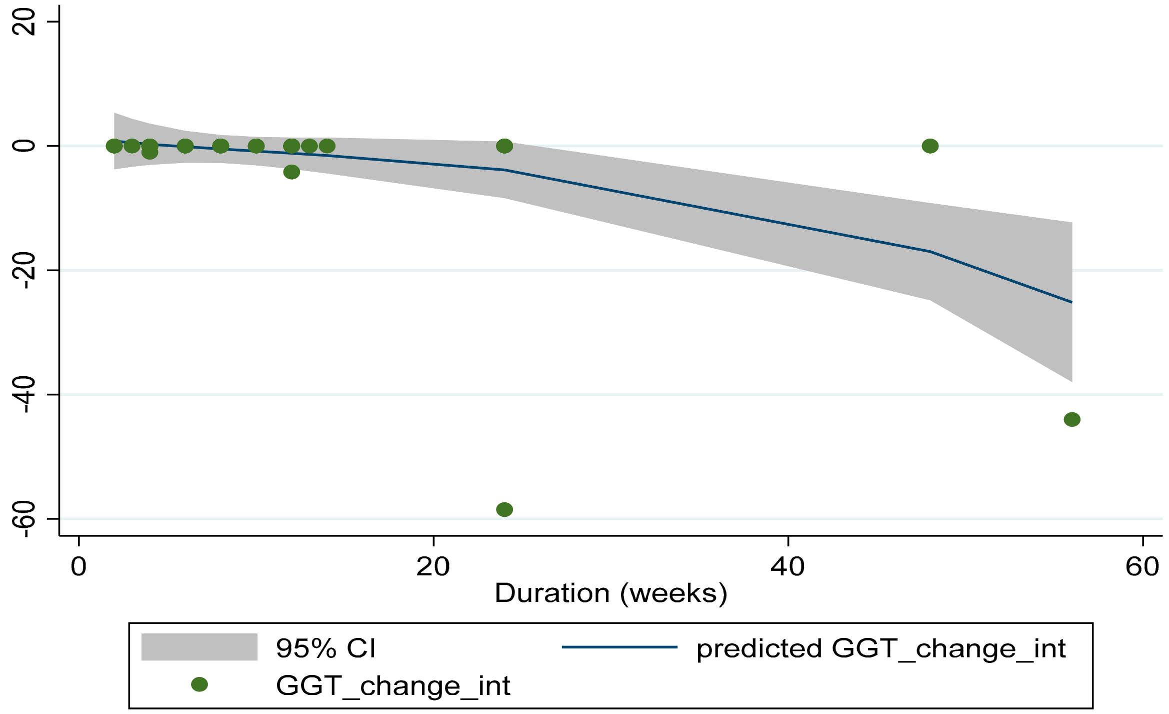

Figure S3. Non-linear association between duration (weeks) of melatonin supplementation and mean changes in cardiometabolic risk factors (CMRFs), including **(A)** BW (kg), **(B)** BMI (kg/m^2), **(C)** WC (cm), **(D)** HC (cm), **(E)** BFP (%), **(F)** FBG (mg/dL), **(G)** HbA1c (%), **(H)** FI (μIU/mL), **(I)** HOMA-IR, **(J)** TG (mg/dL), **(K)** TC (mg/dL), **(L)** LDL-C (mg/dL), **(M)** HDL-C (mg/dL), **(N)** SBP (mmHg), **(O)** DBP (mmHg), **(P)** MDA (μmol/L), **(Q)** TAC (mmol/L), **(R)** CRP (mg/L), **(S)** IL-6 (pg/mL), **(T)** TNF-α (pg/mL), **(U)** AST (IU/L), **(V)** ALT (IU/L), and **(W)** GGT (IU/L).

A) BW

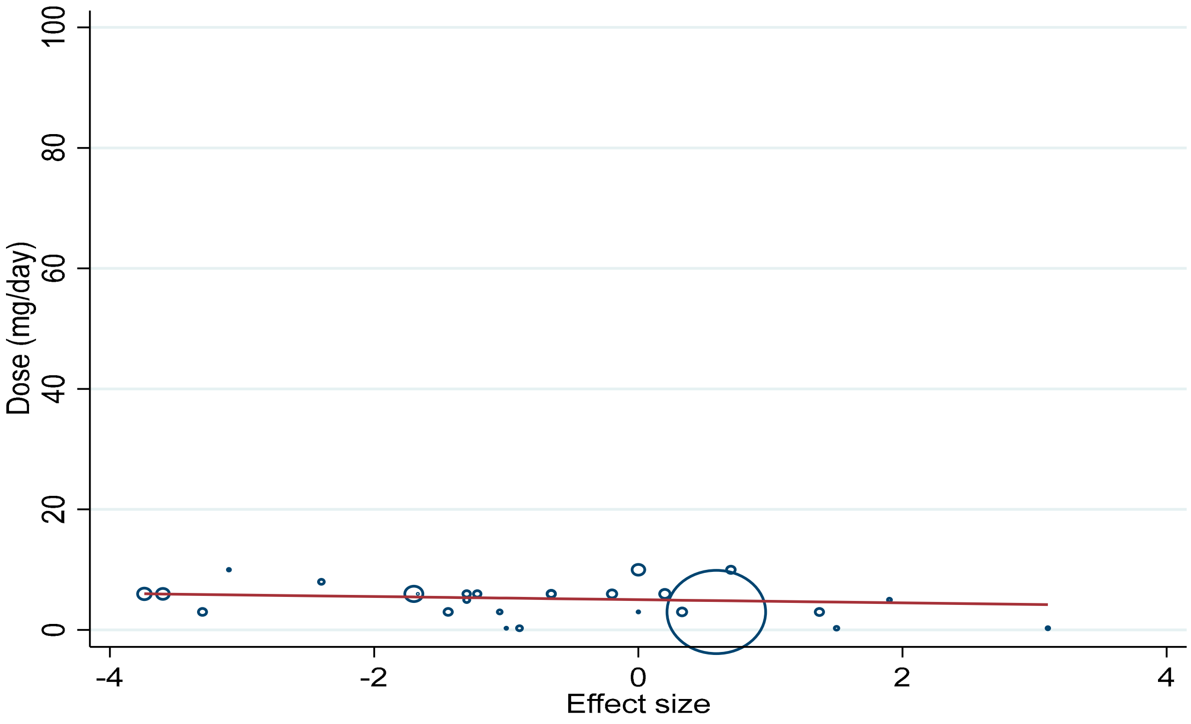

B) BMI

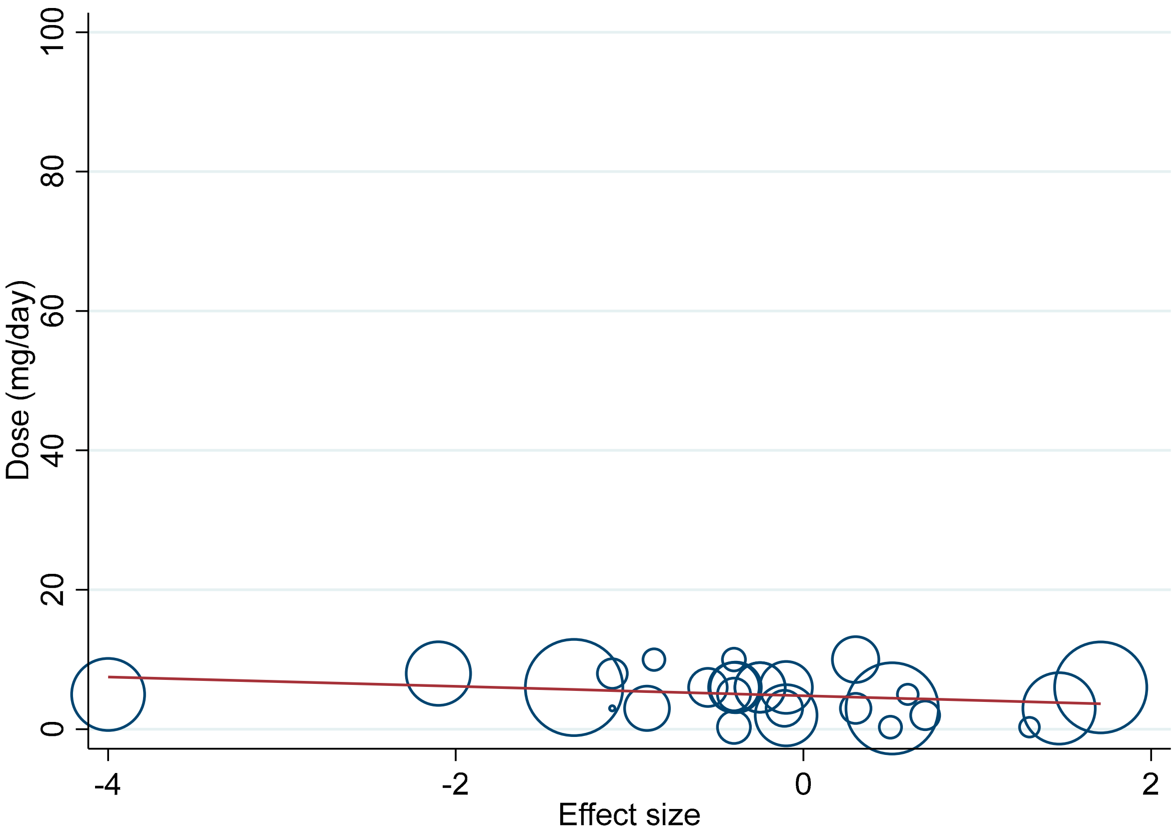

Figure S4. *Cont.*

C) WC

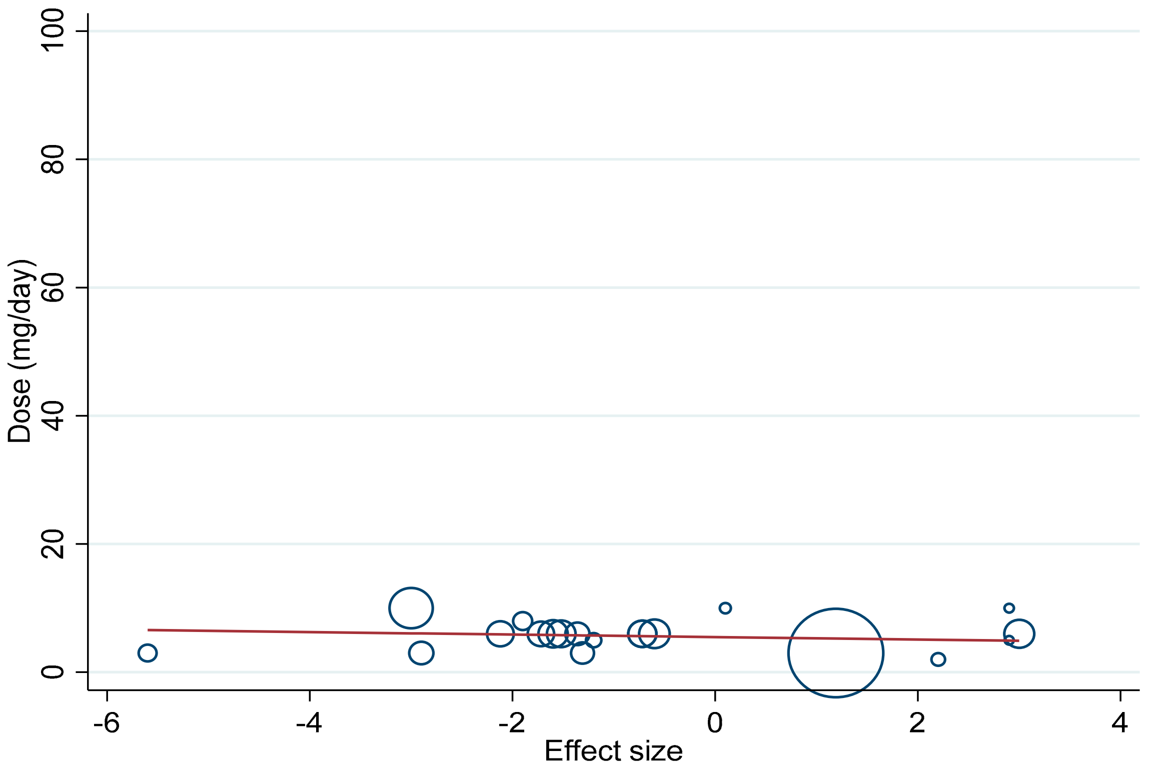

D) HC

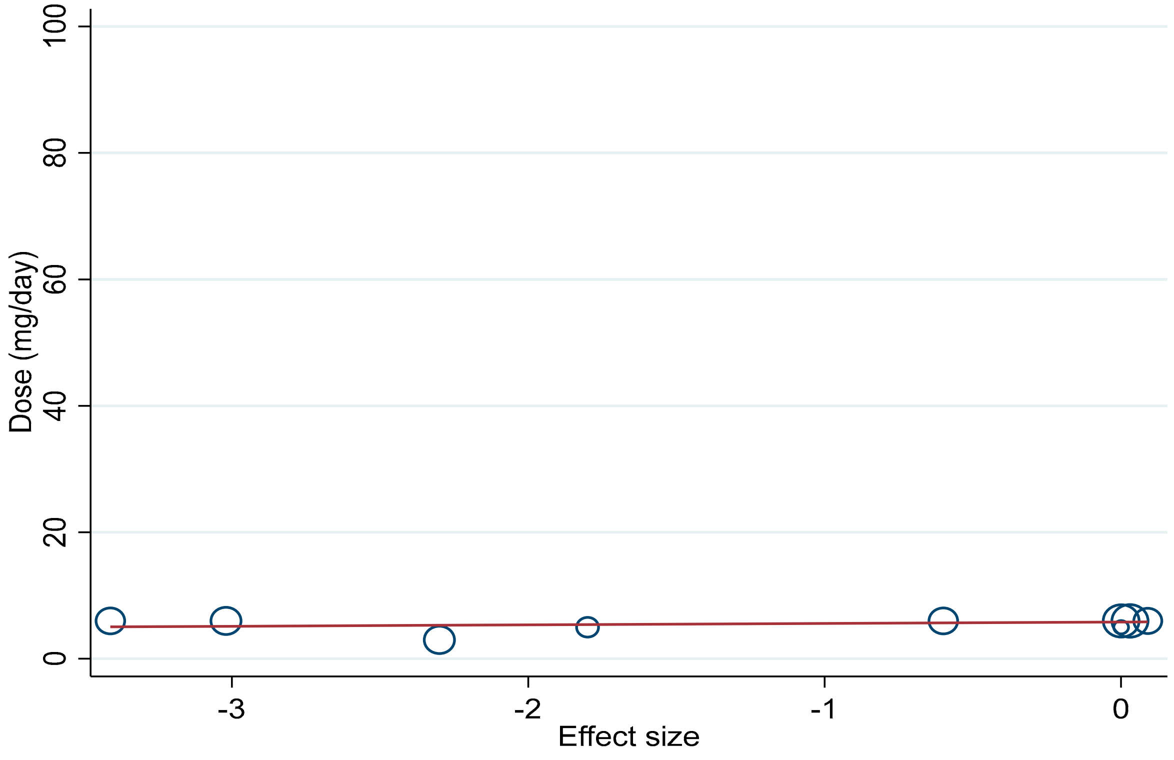

Figure S4. *Cont.*

E) BFP

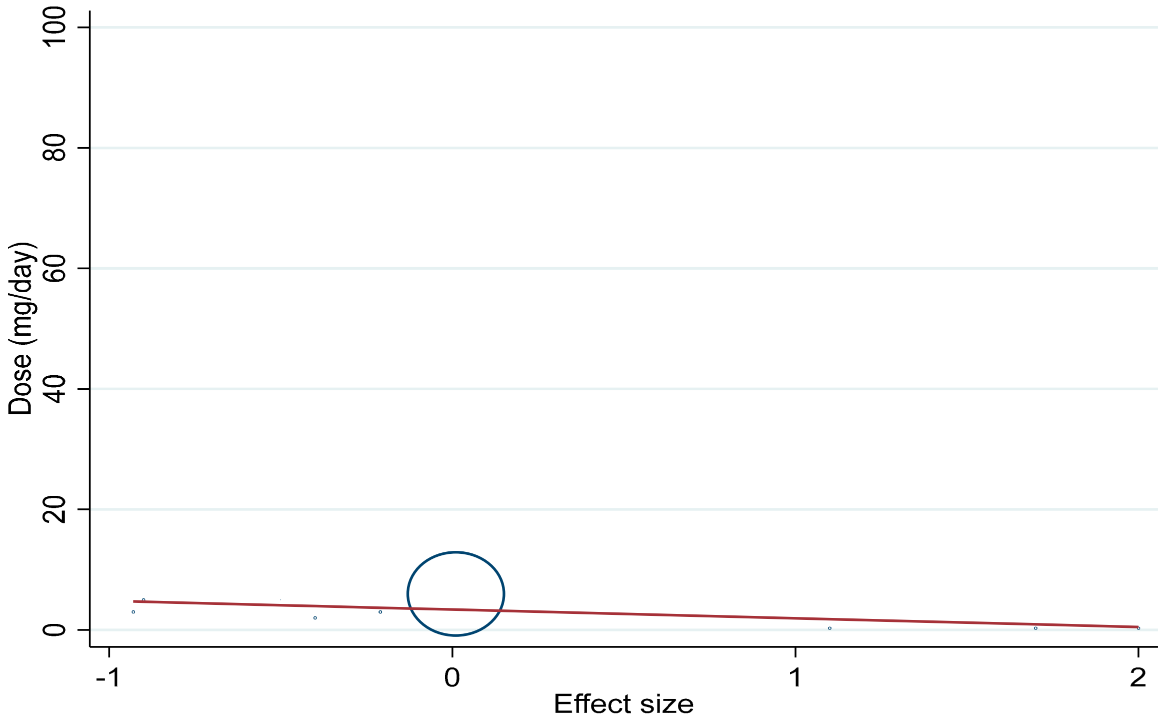

F) FBG

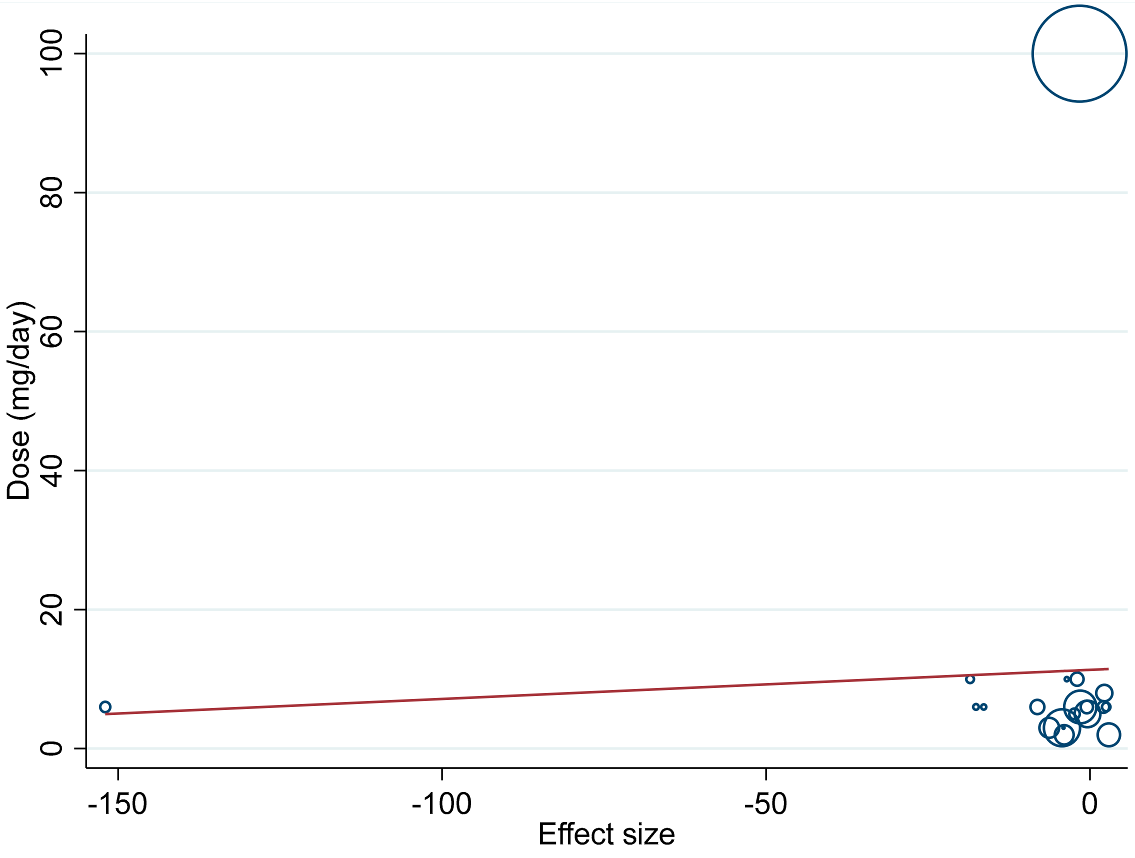

Figure S4. *Cont.*

G) HbA1c

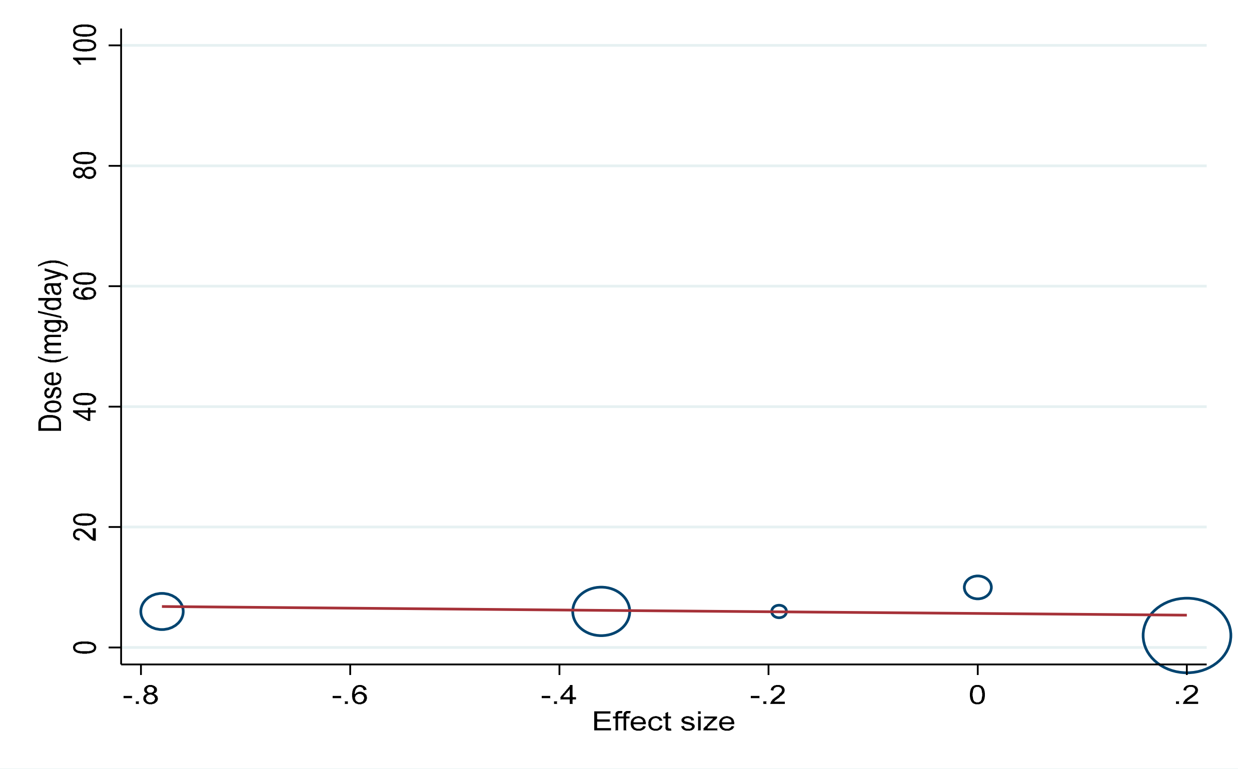

H) FI

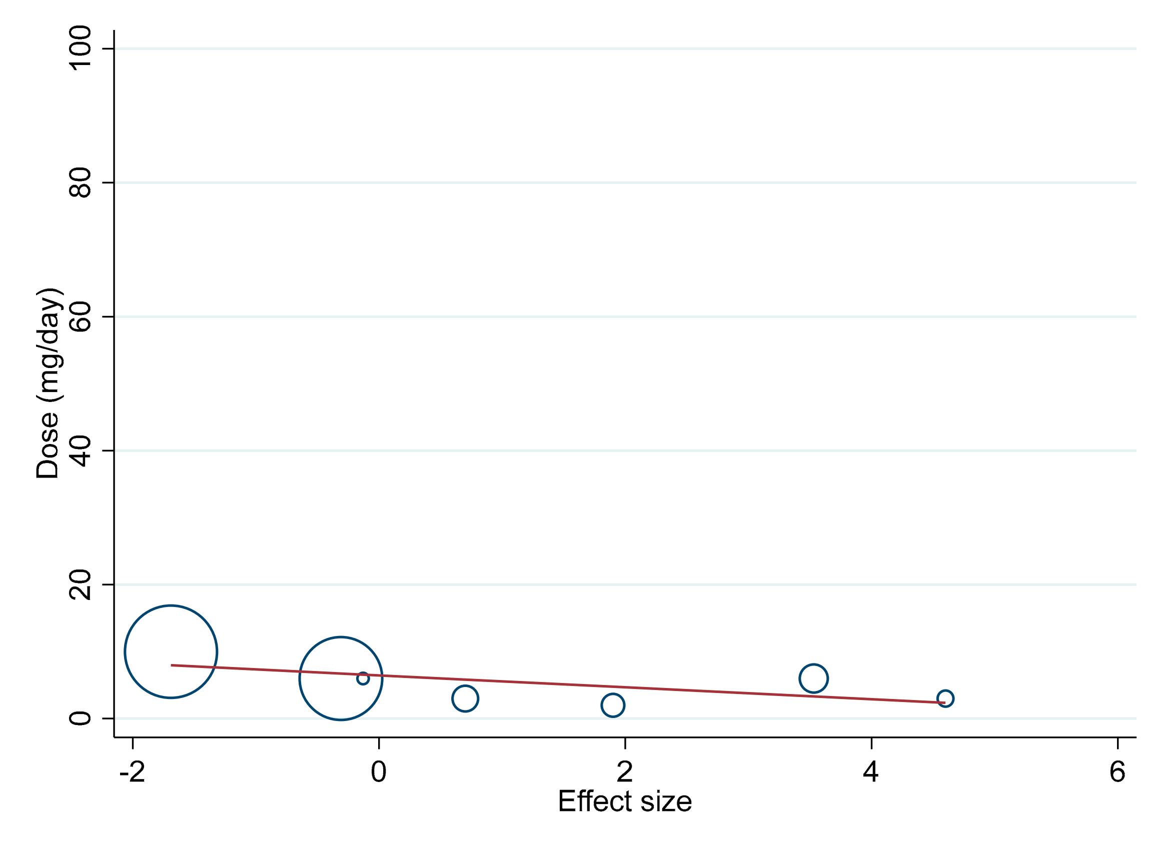

Figure S4. *Cont.*

I) HOMA-IR

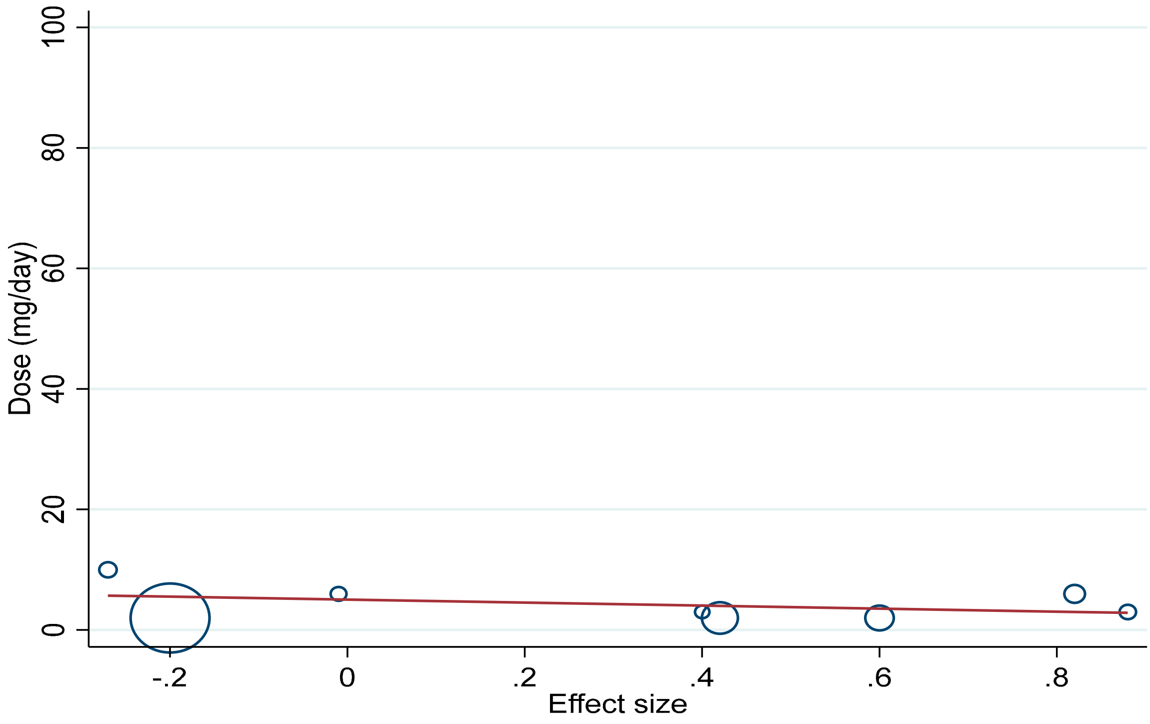

J) TG

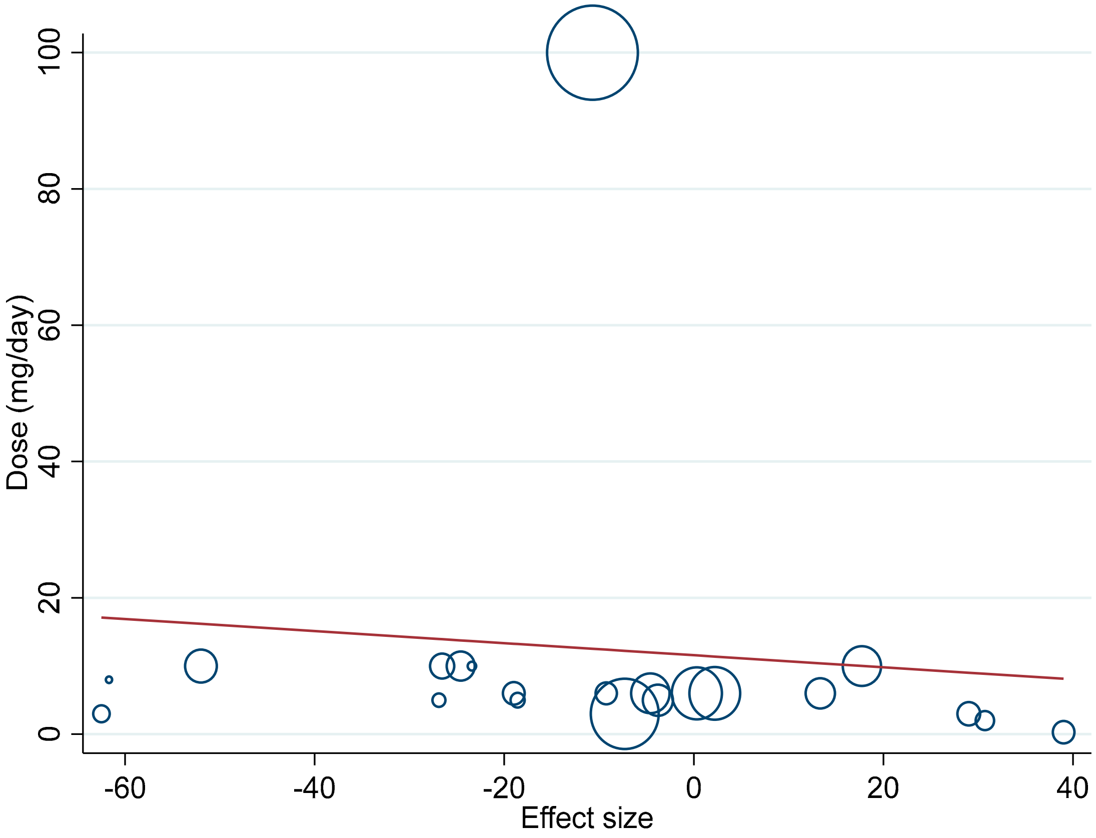

Figure S4. *Cont.*

K) TC

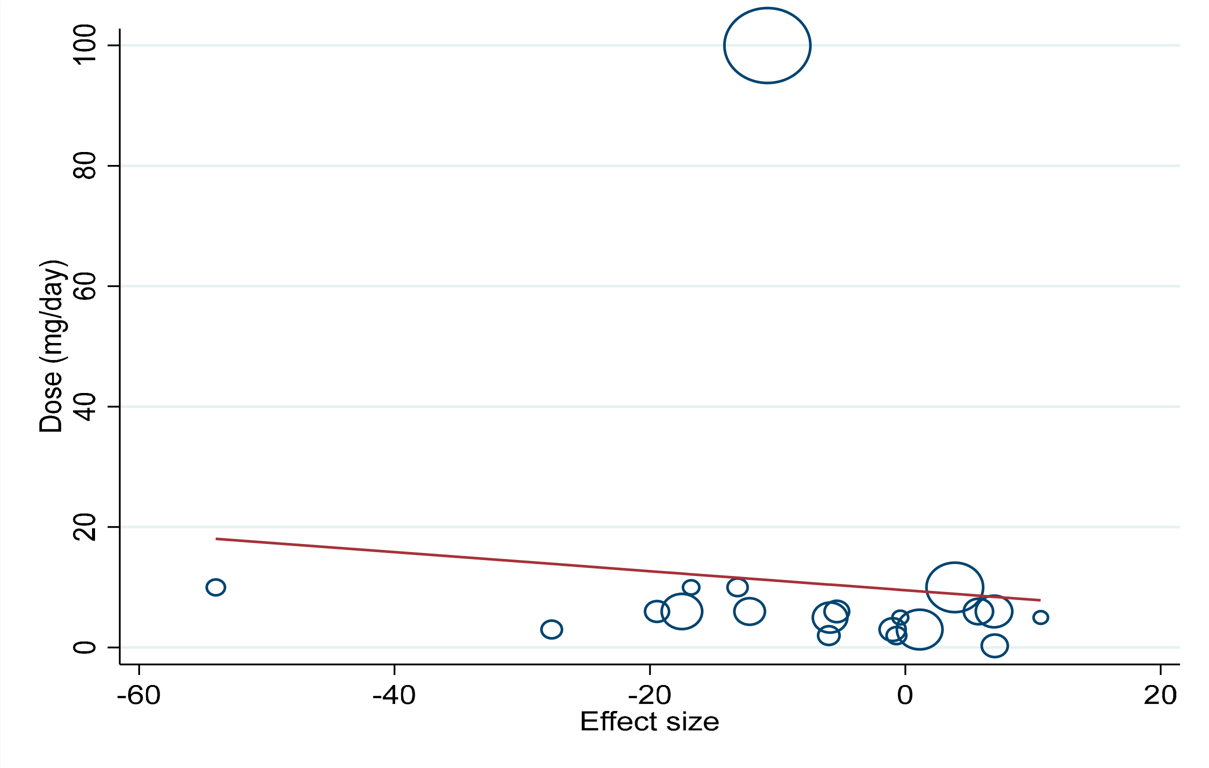

L) LDL-C

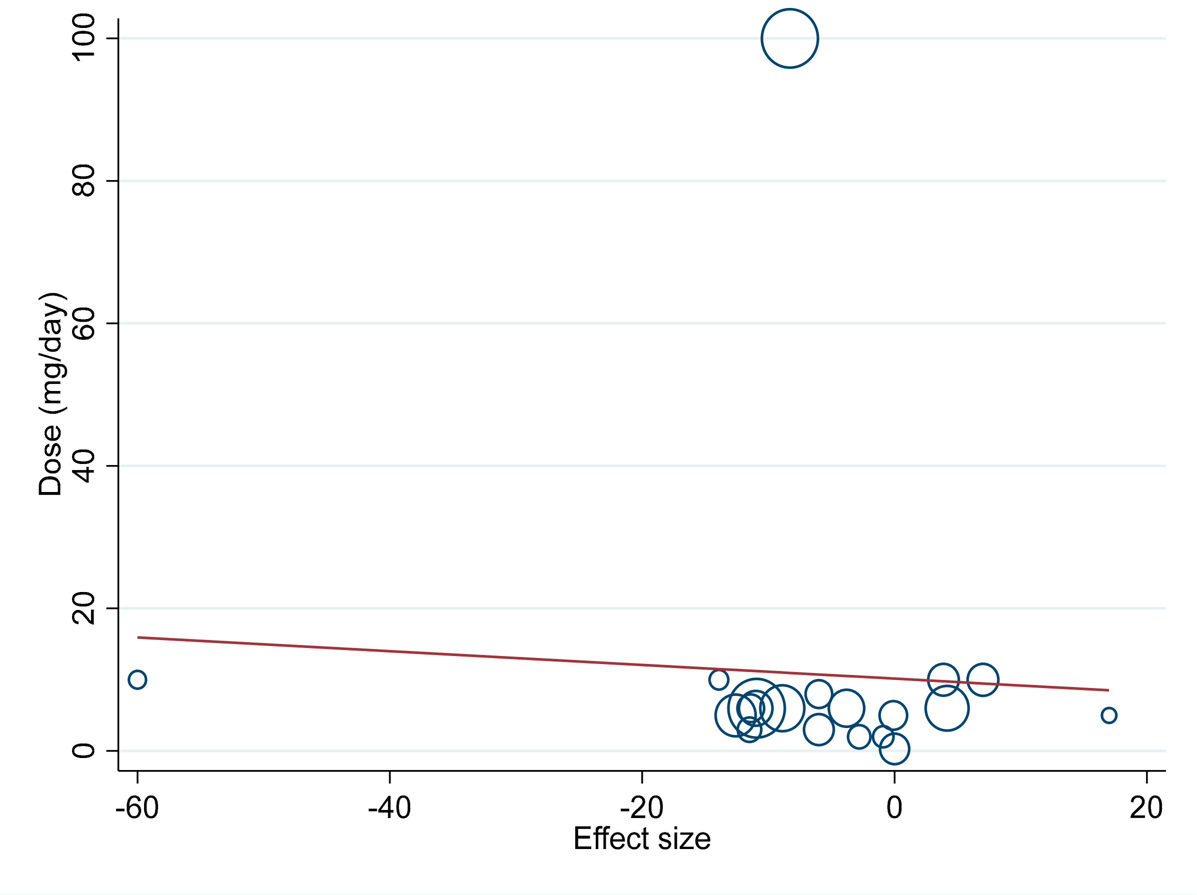

Figure S4. *Cont.*

M) HDL-C

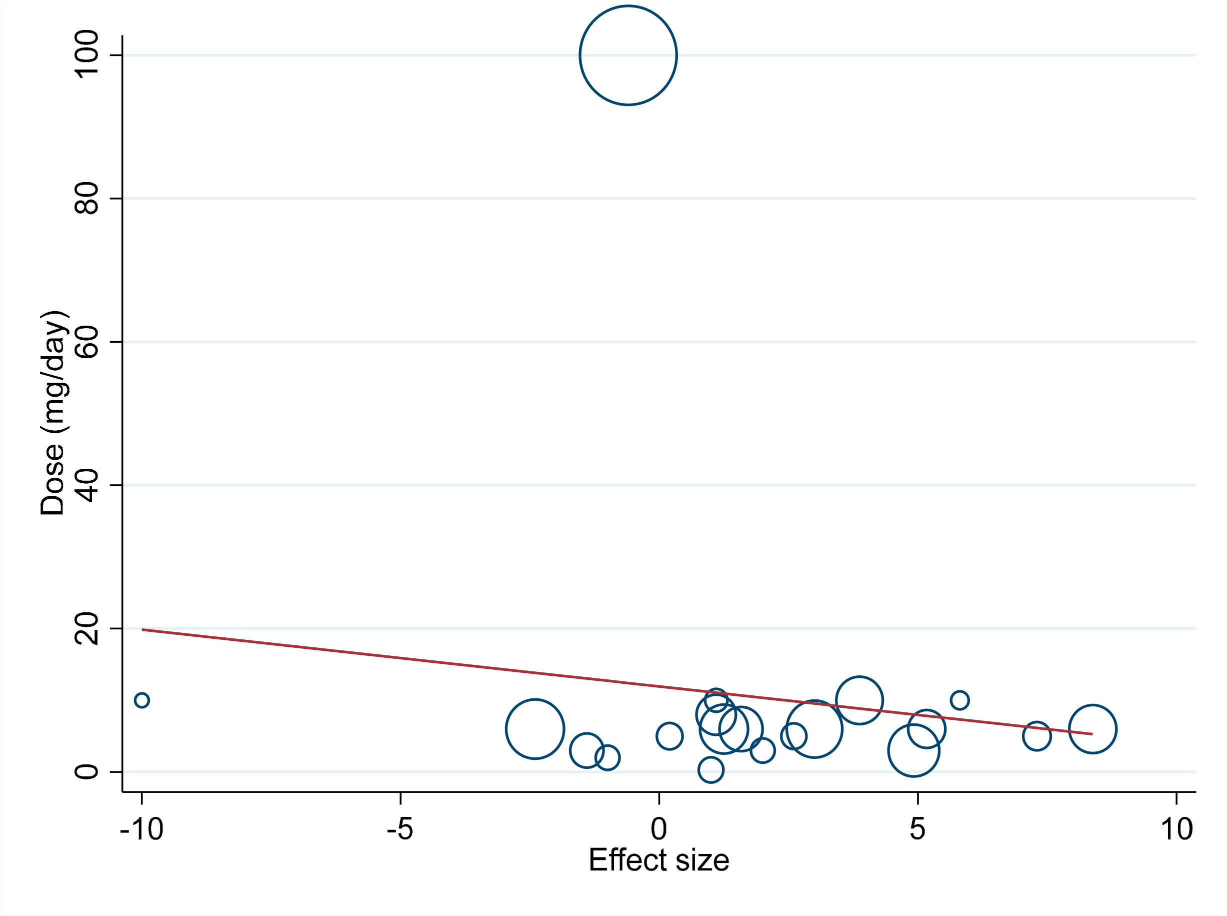

N) SBP

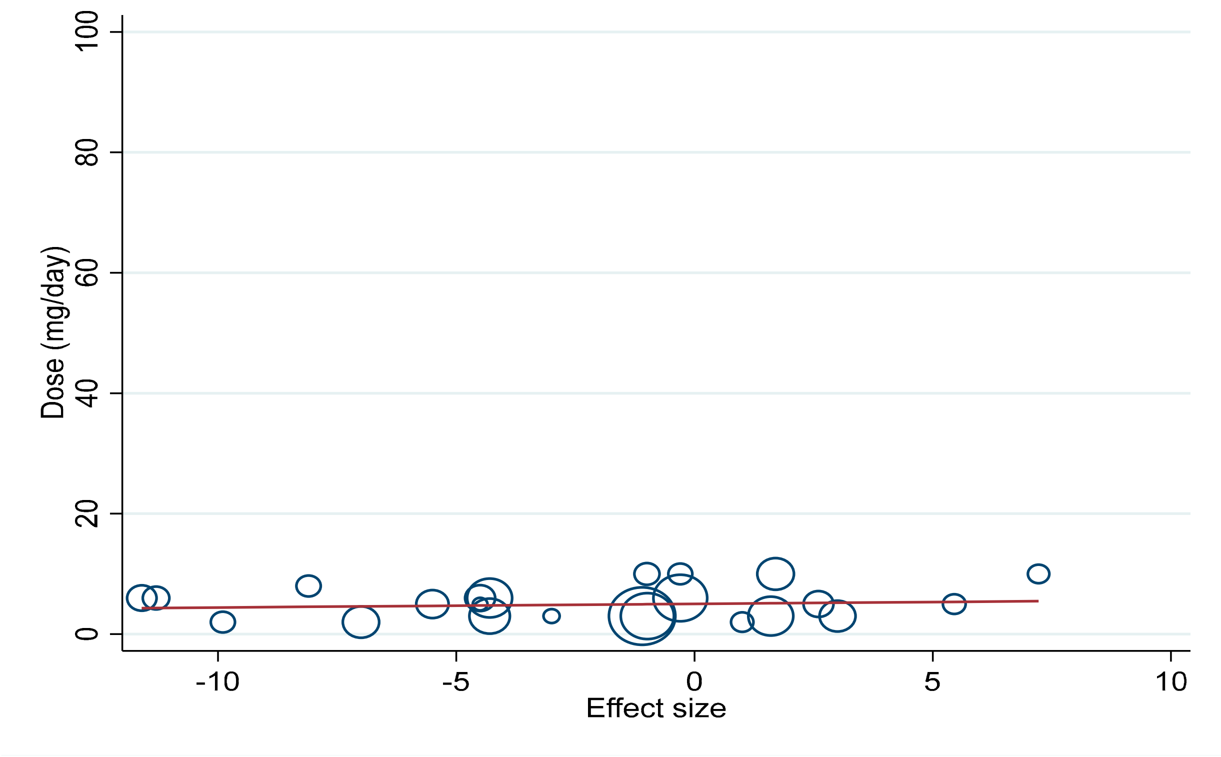

Figure S4. *Cont.*

O) DBP

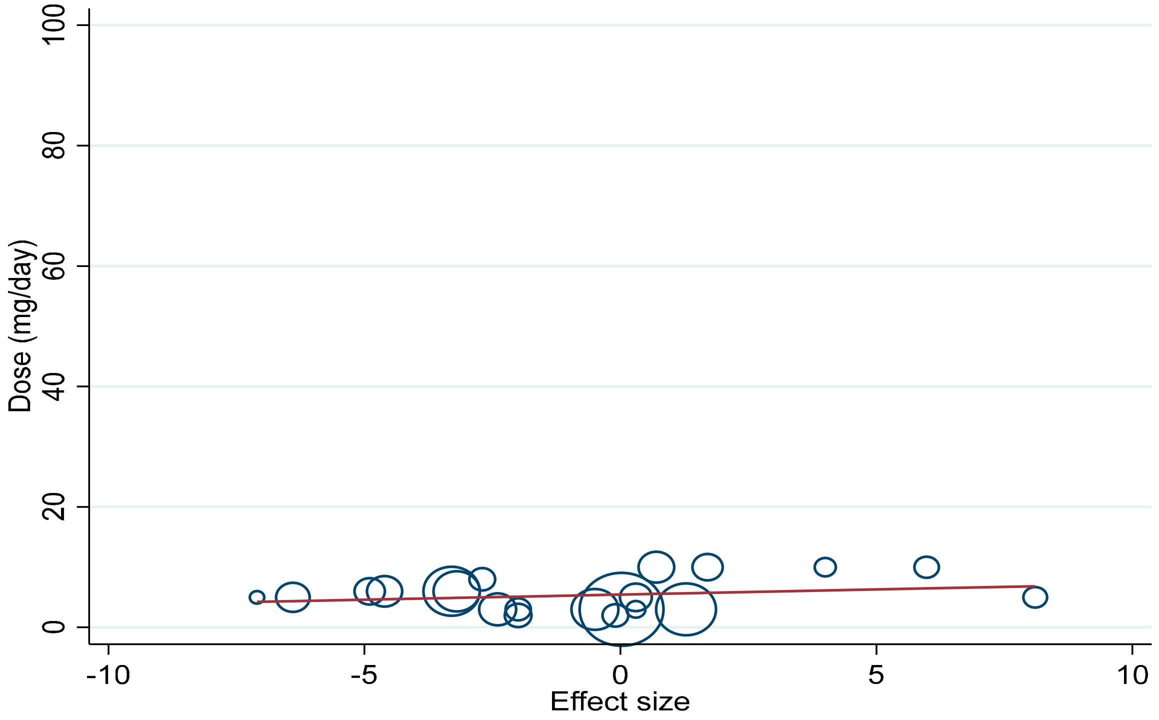

P) MDA

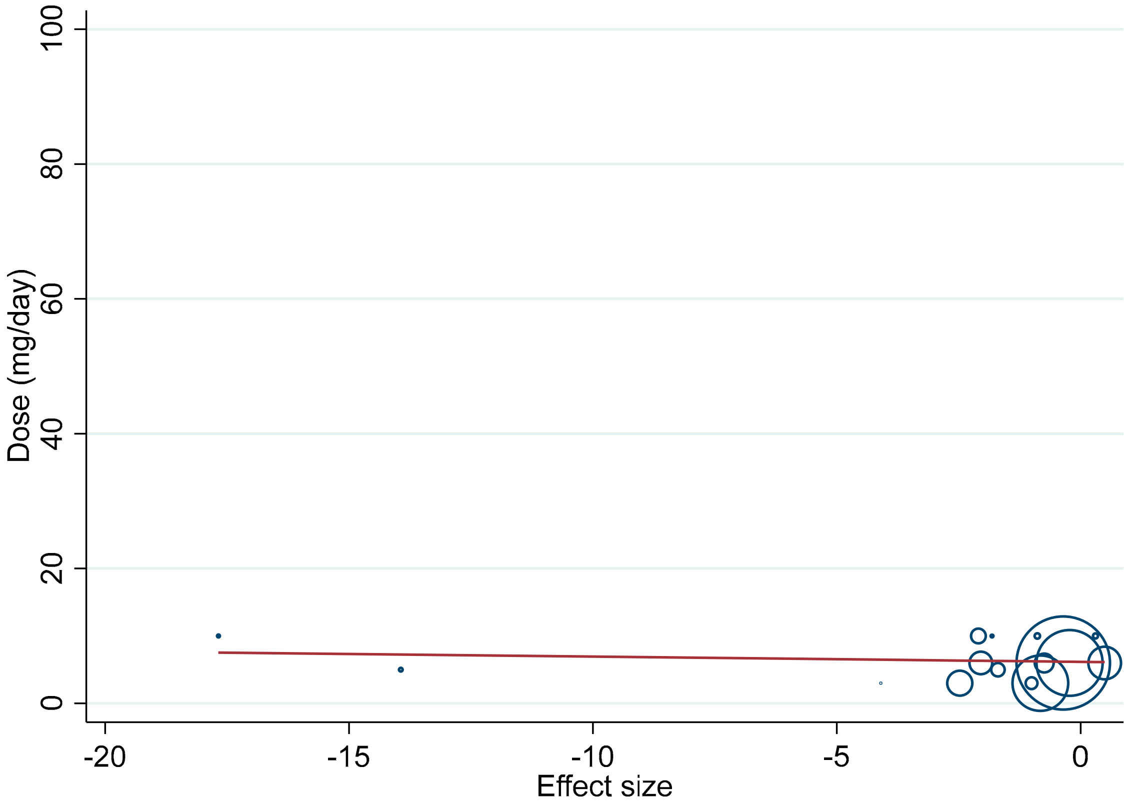

Figure S4. *Cont.*

Q) TAC

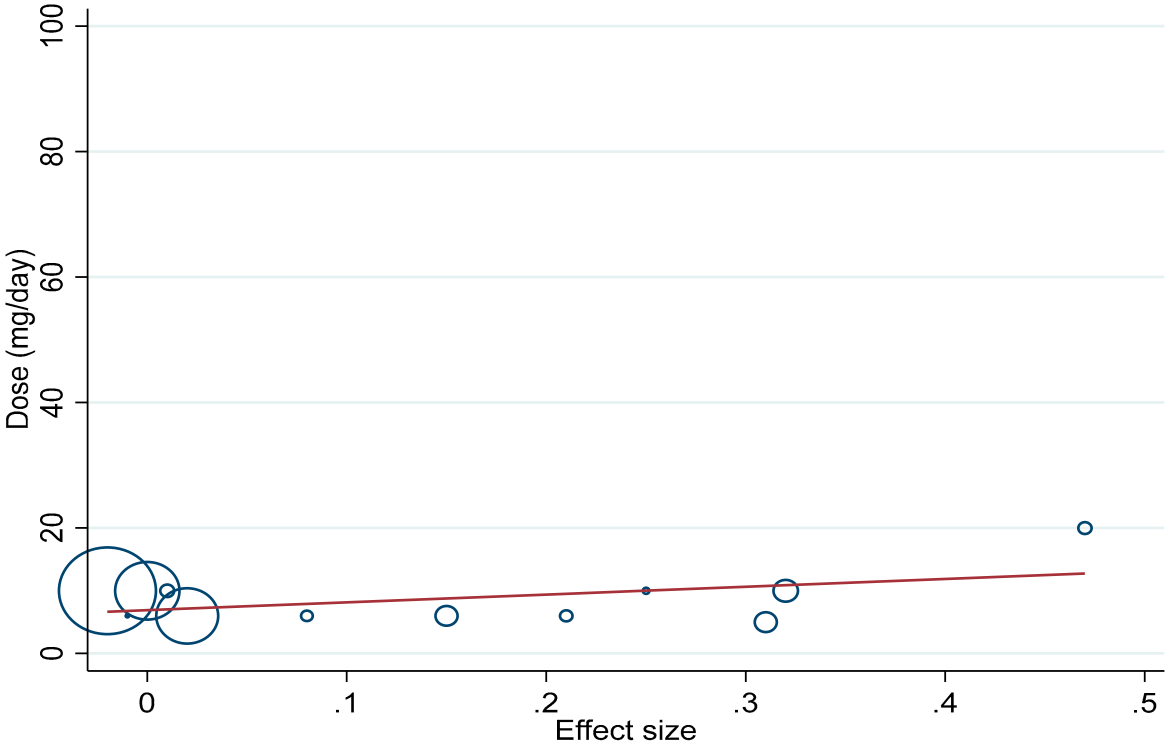

R) CRP

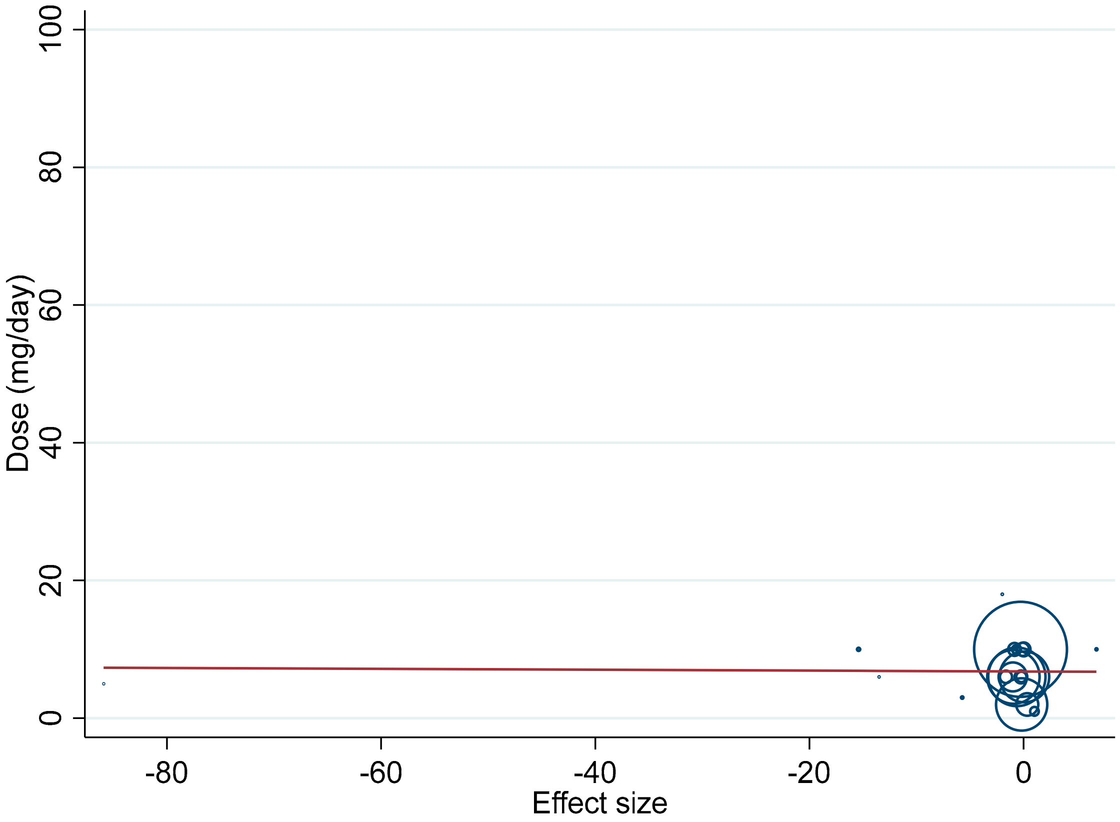

Figure S4. *Cont.*

S) **IL-6**

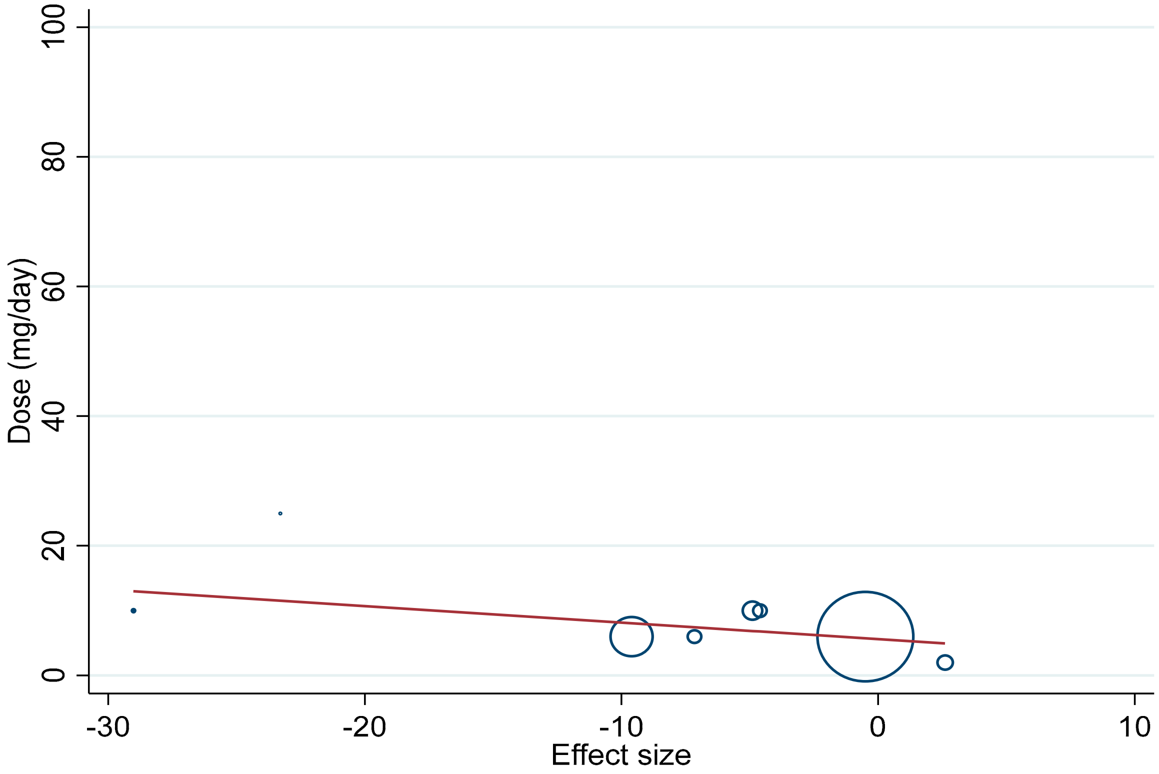

T) **TNF-*α***

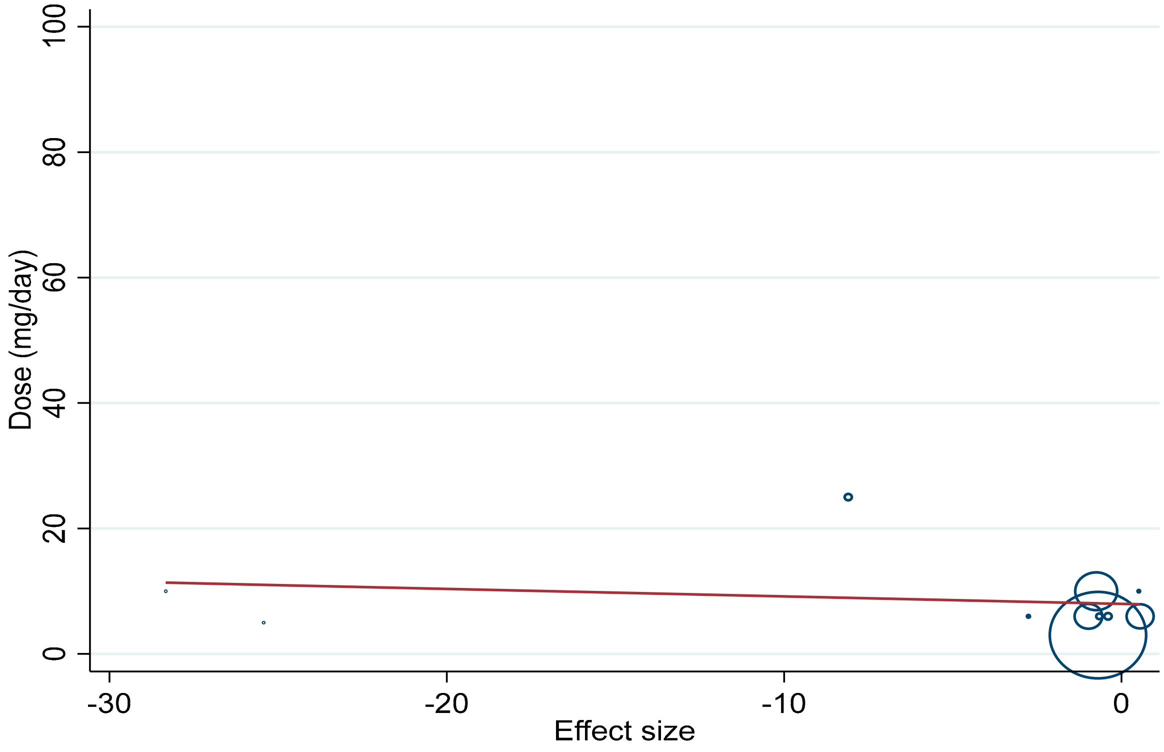

Figure S4. *Cont.*

U) AST

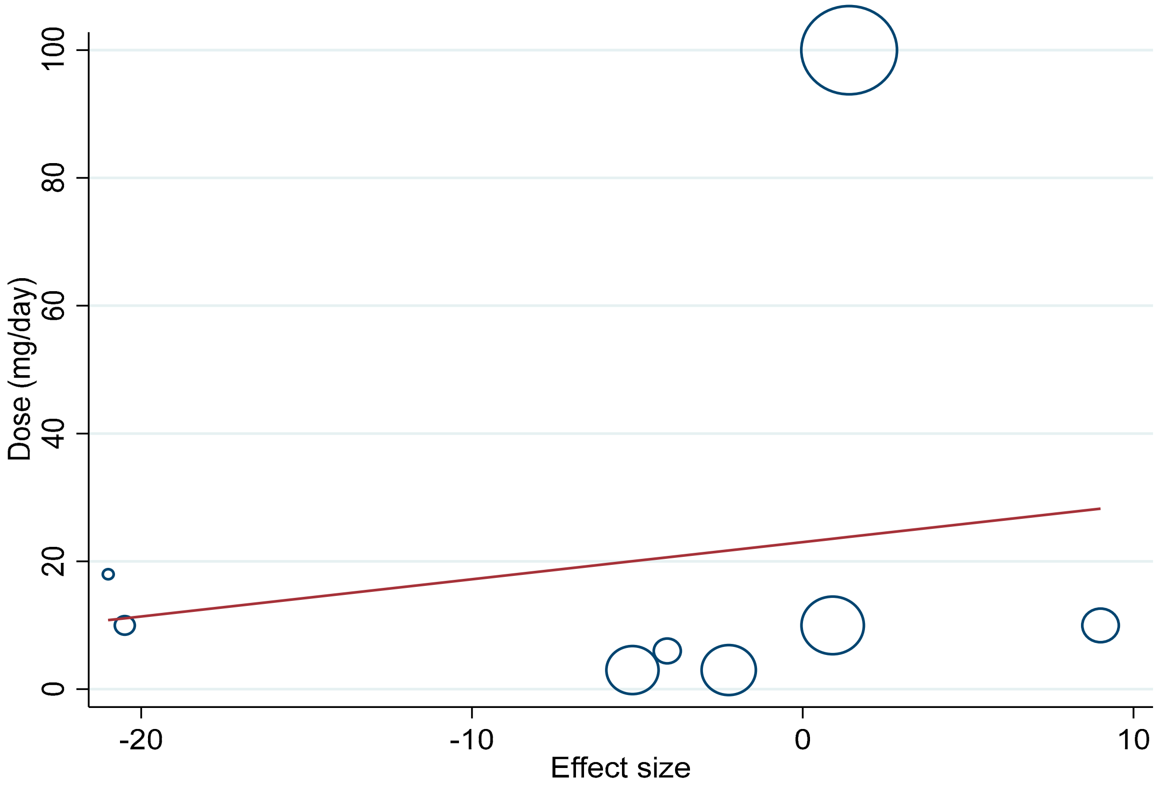

V) ALT

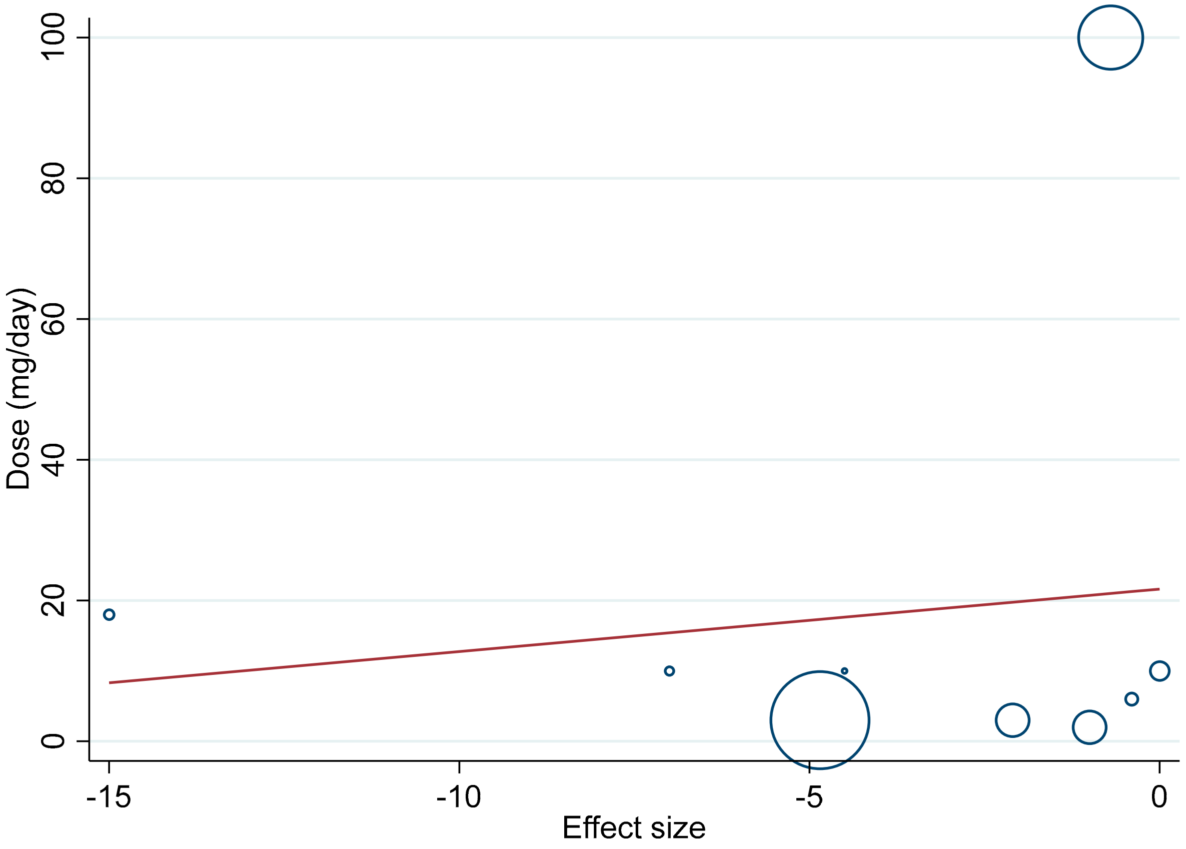

Figure S4. *Cont.*

W) GGT

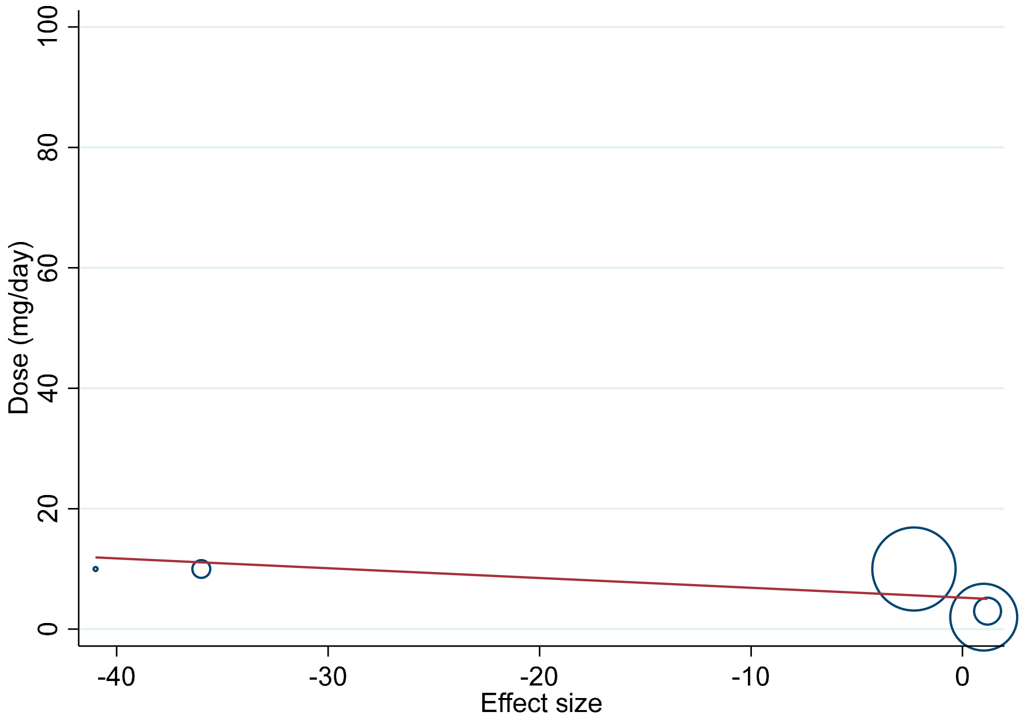

Figure S4. Linear dose-response association between dose (mg/day) of melatonin supplementation and mean changes in cardiometabolic risk factors (CMRFs), including **(A)** BW (kg), **(B)** BMI (kg/m²), **(C)** WC (cm), **(D)** HC (cm), **(E)** BFP (%), **(F)** FBG (mg/dL), **(G)** HbA1c (%), **(H)** FI (μIU/mL), **(I)** HOMA-IR, **(J)** TG (mg/dL), **(K)** TC (mg/dL), **(L)** LDL-C (mg/dL), **(M)** HDL-C (mg/dL), **(N)** SBP (mmHg), **(O)** DBP (mmHg), **(P)** MDA (μmol/L), **(Q)** TAC (mmol/L), **(R)** CRP (mg/L), **(S)** IL-6 (pg/mL), **(T)** TNF-α (pg/mL), **(U)** AST (IU/L), **(V)** ALT (IU/L), and **(W)** GGT (IU/L).

A) BW

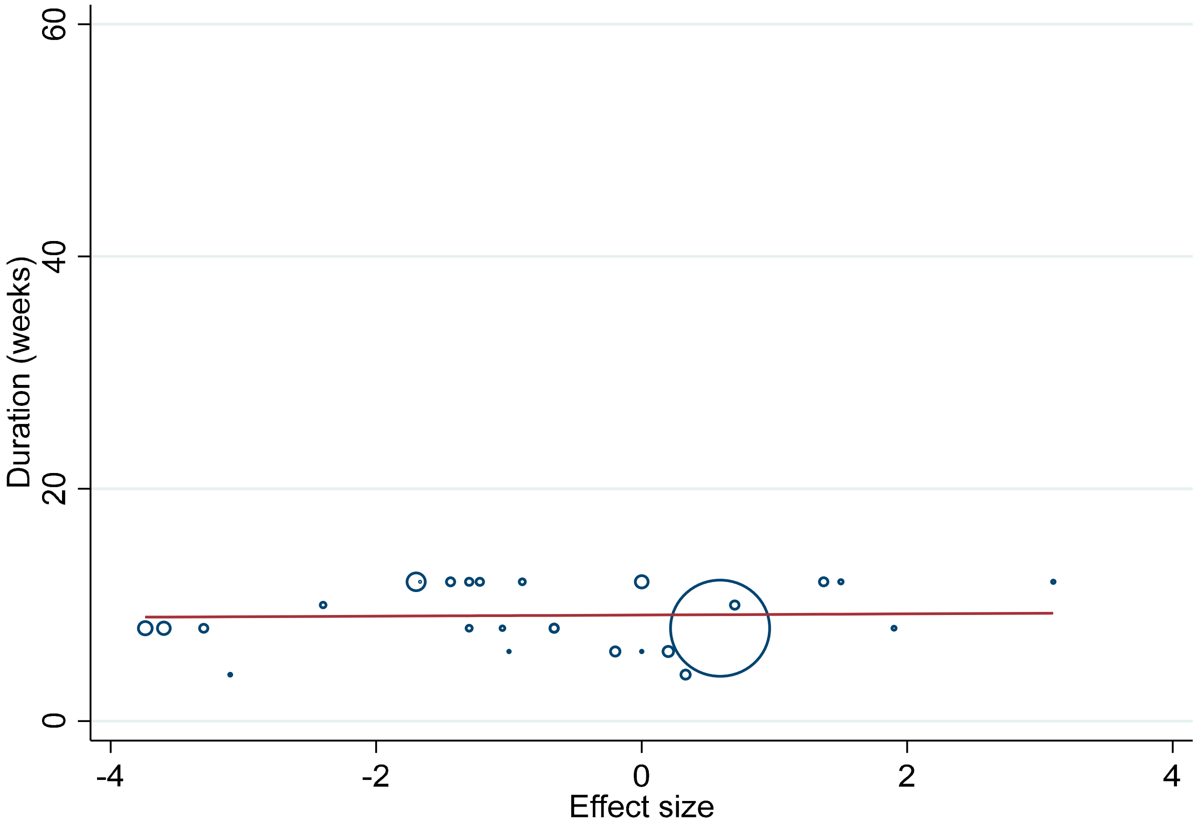

B) BMI

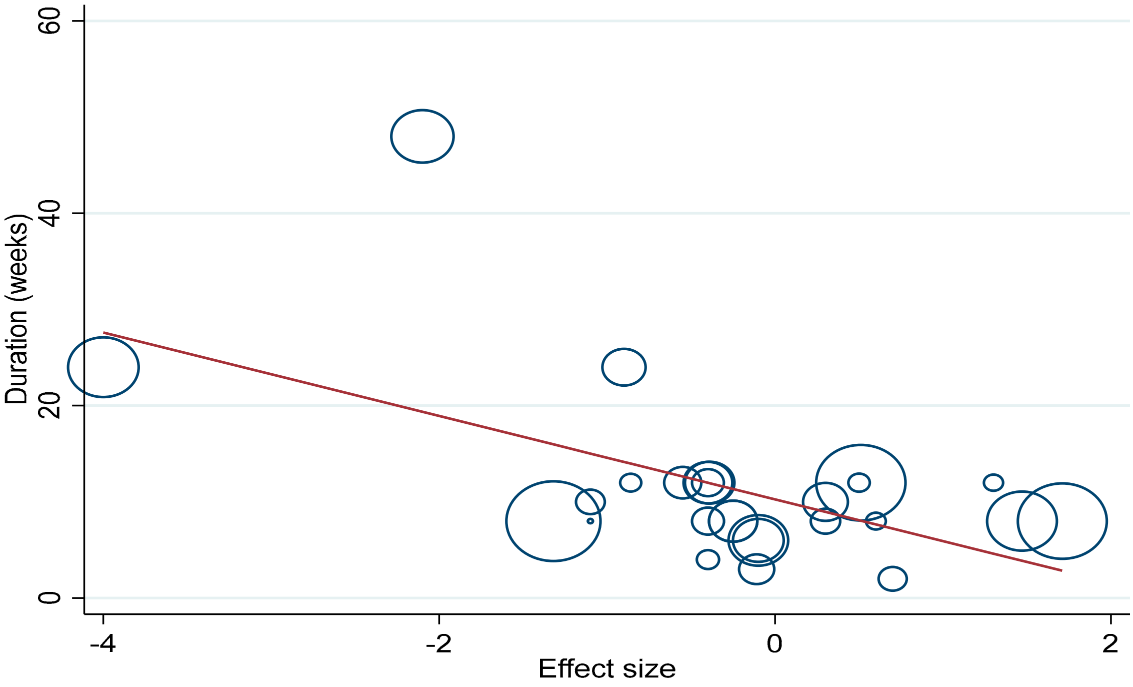

Figure S5. *Cont.*

C) WC

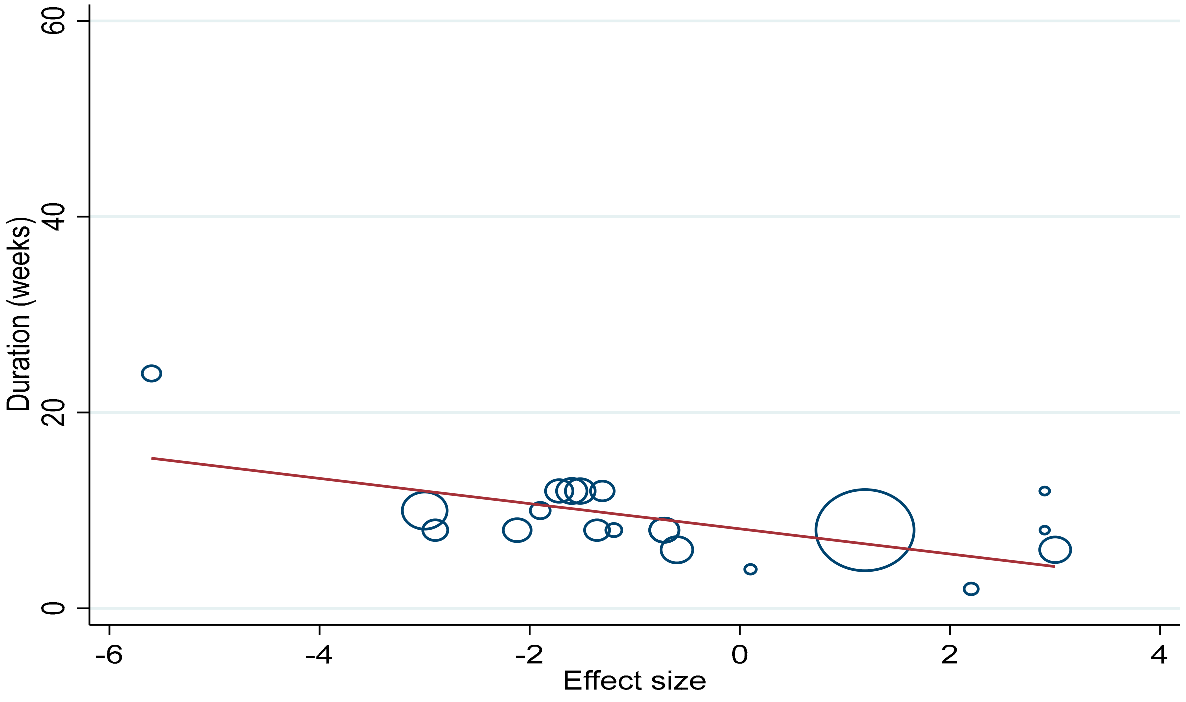

D) HC

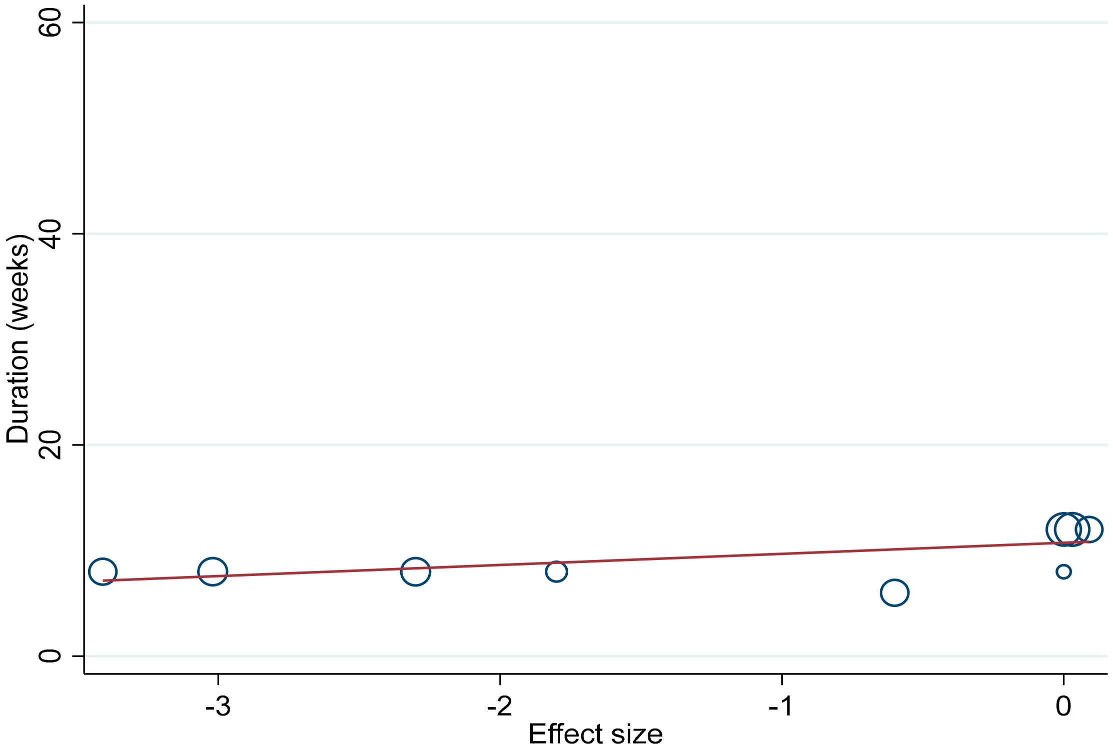

Figure S5. *Cont.*

E) BFP

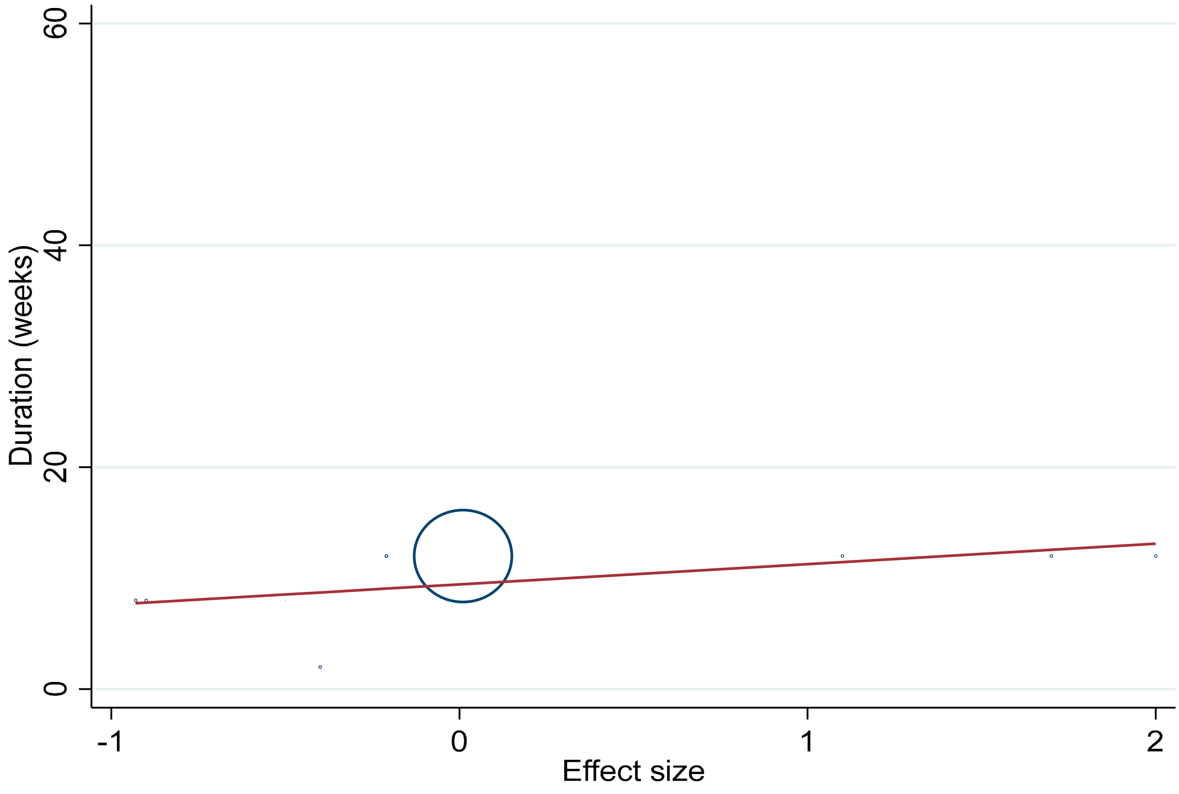

F) FBG

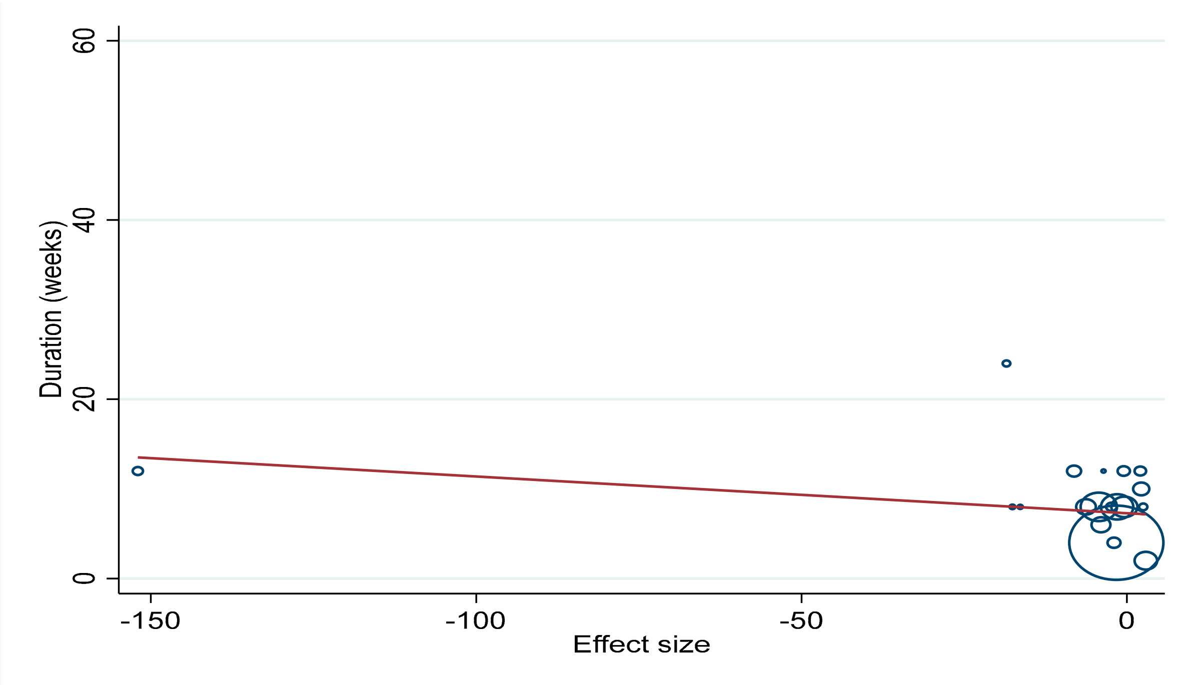

Figure S5. *Cont.*

G) HbA1c

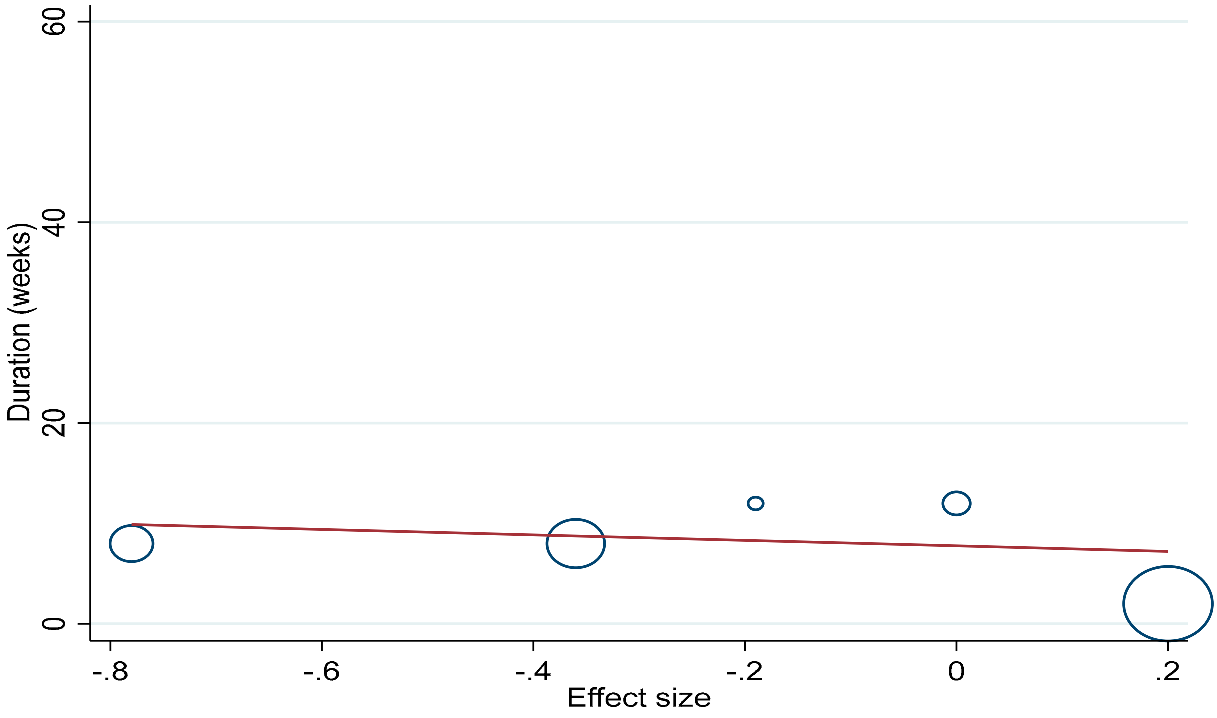

H) FI

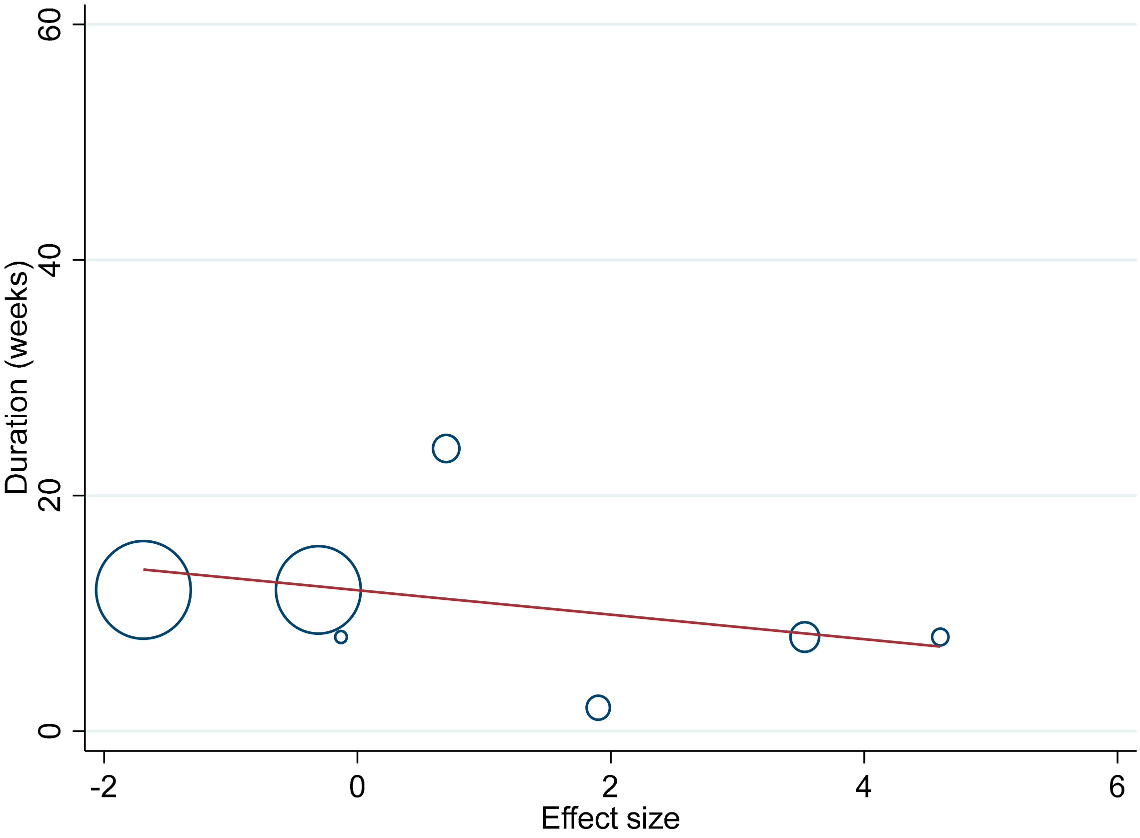

Figure S5. *Cont.*

I) HOMA-IR

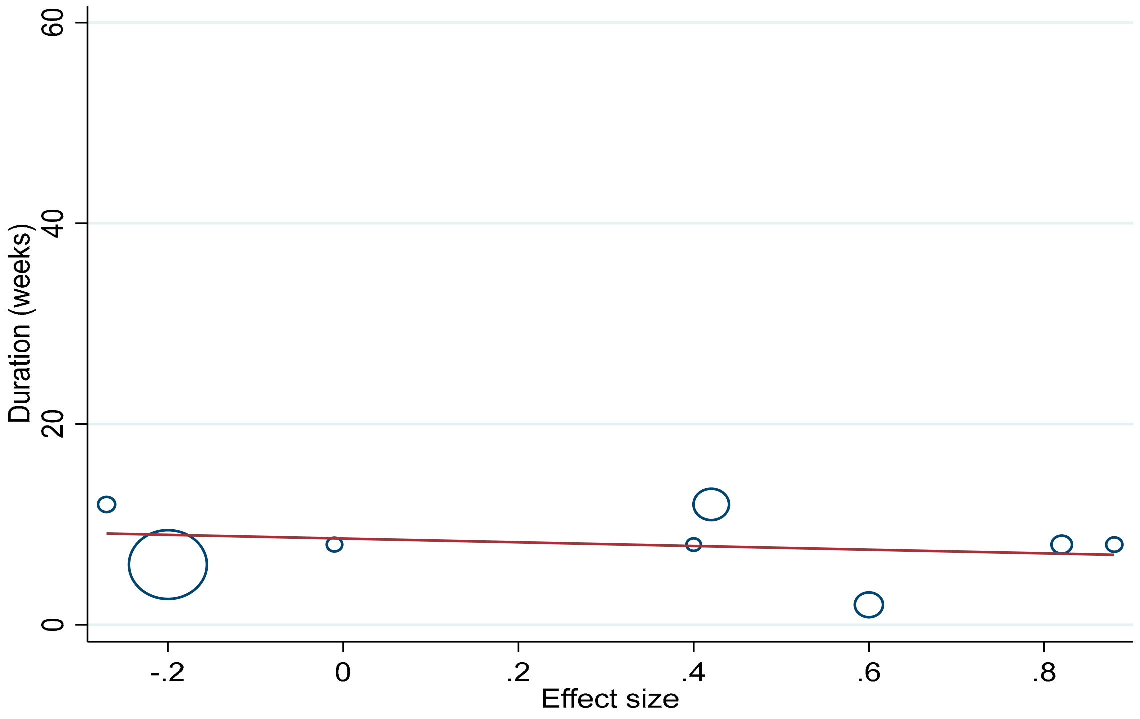

J) TG

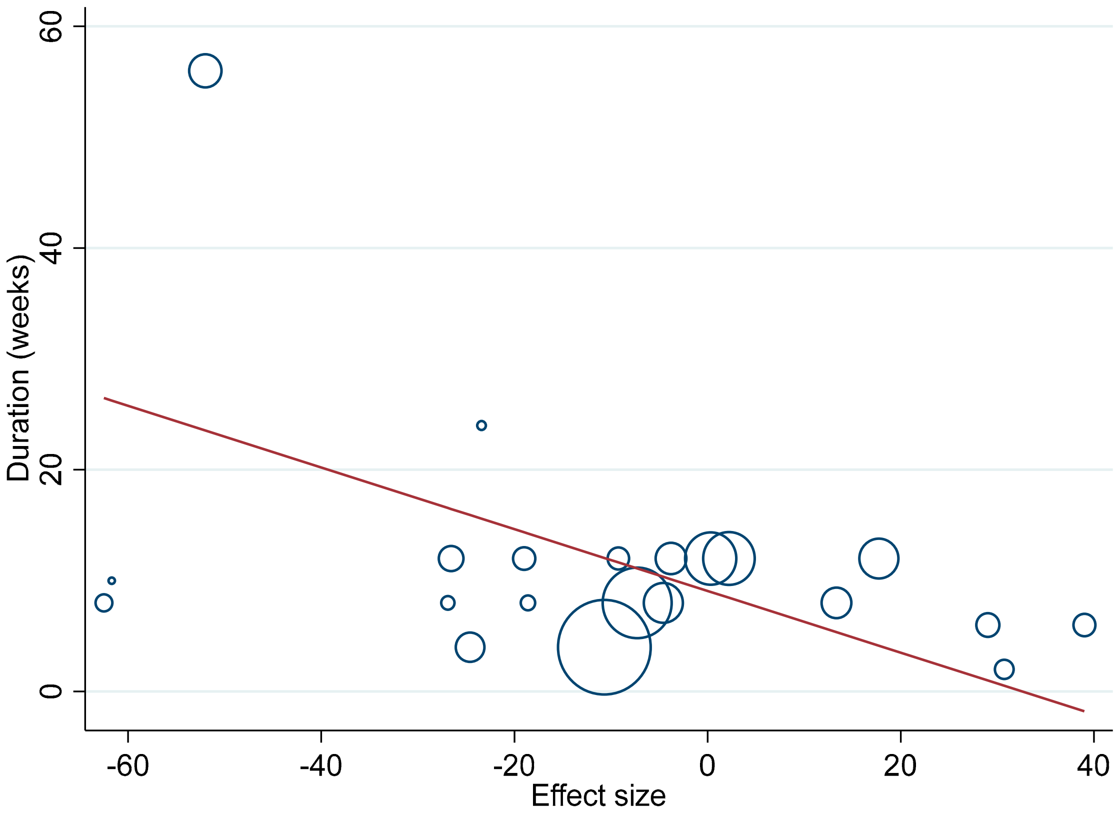

Figure S5. *Cont.*

K) TC

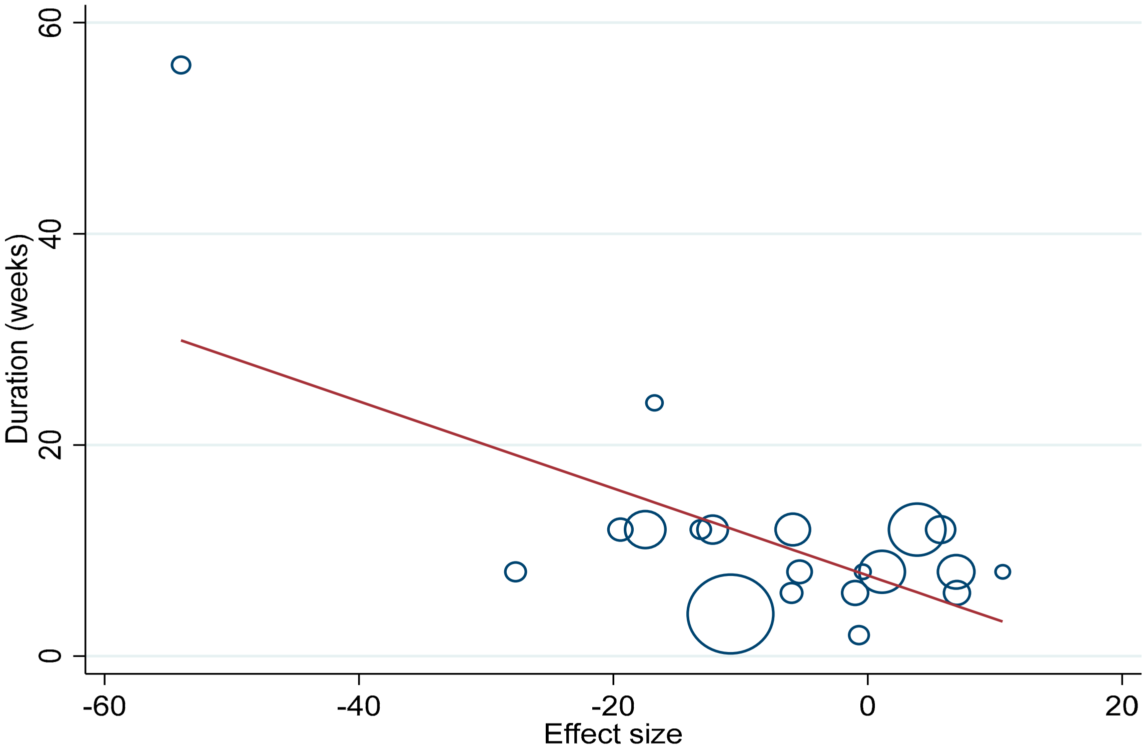

L) LDL-C

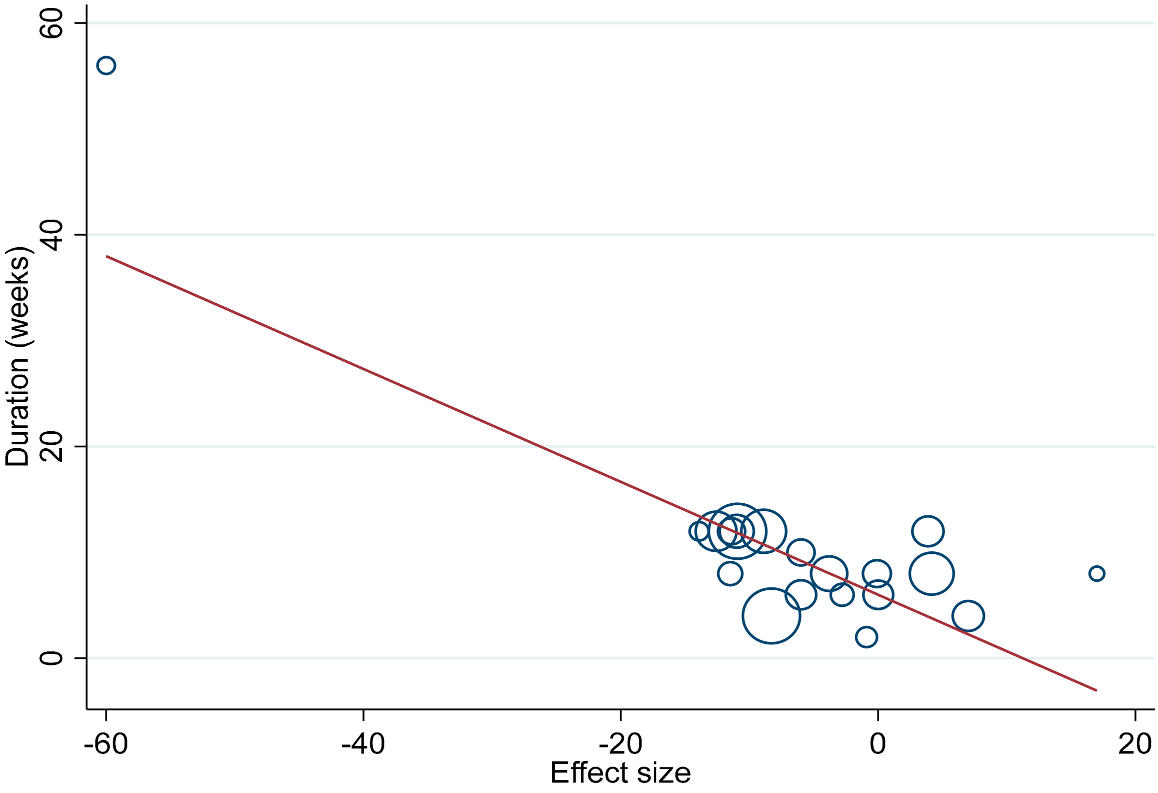

Figure S5. *Cont.*

M) HDL-C

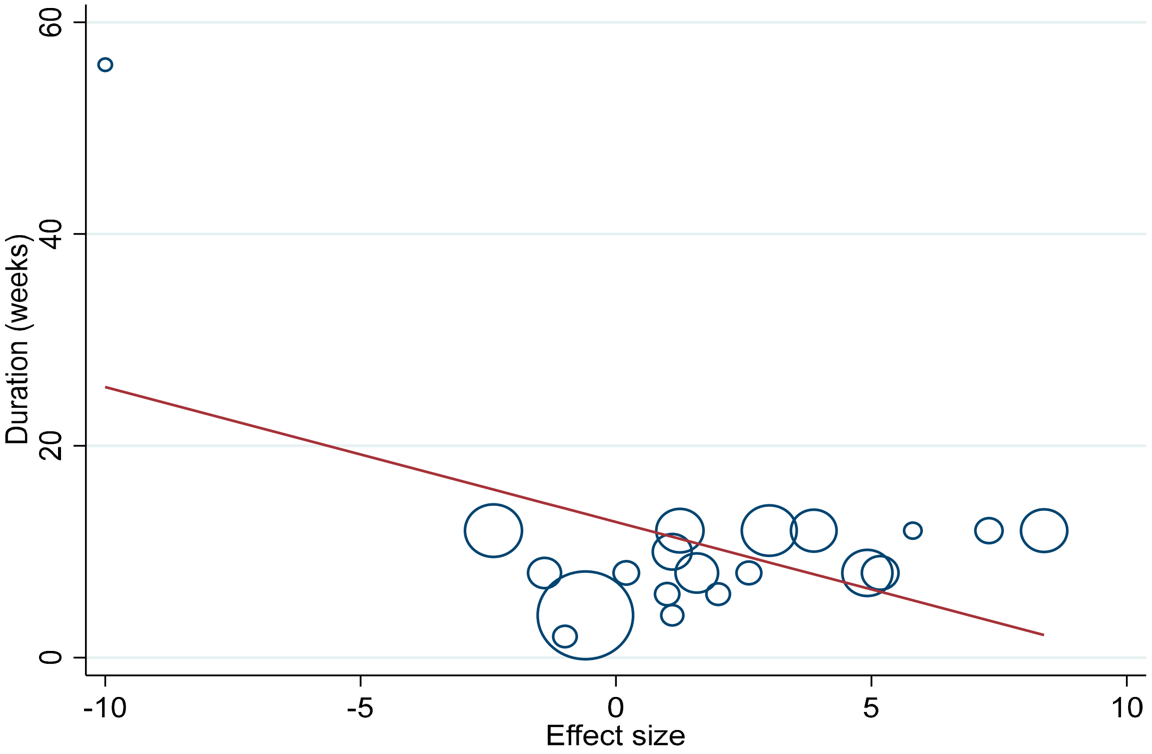

N) SBP

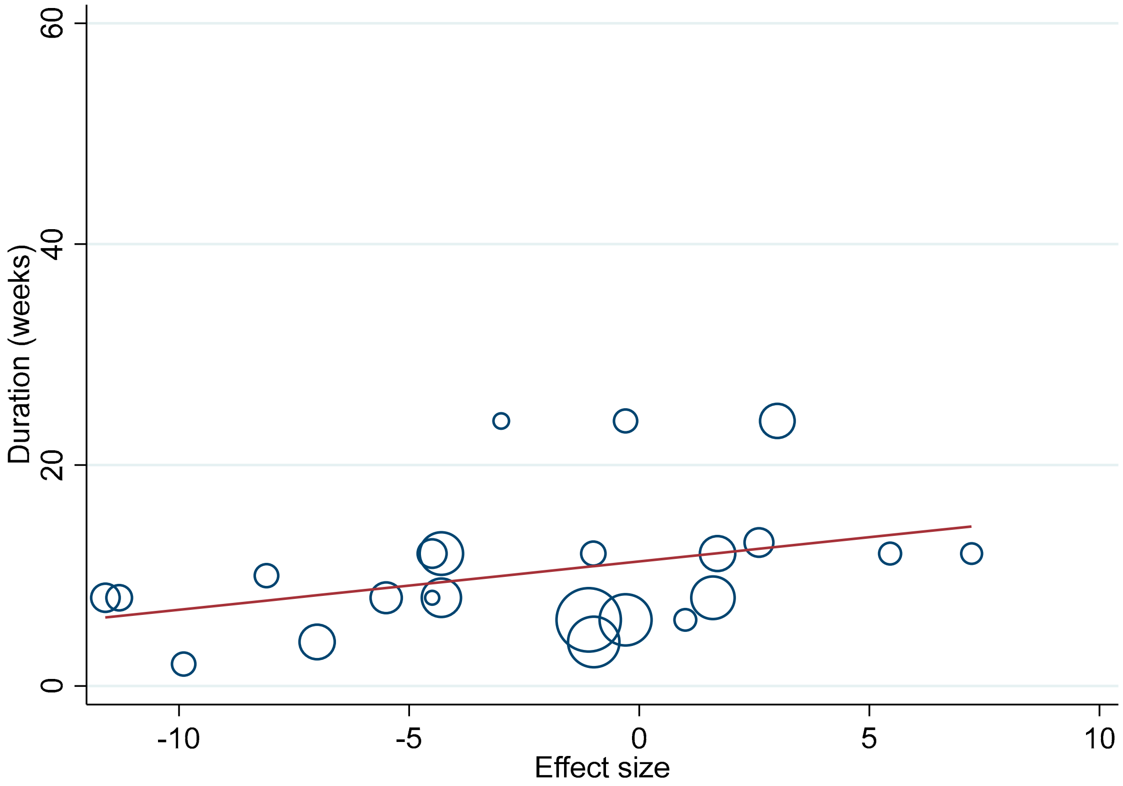

Figure S5. *Cont.*

O) DBP

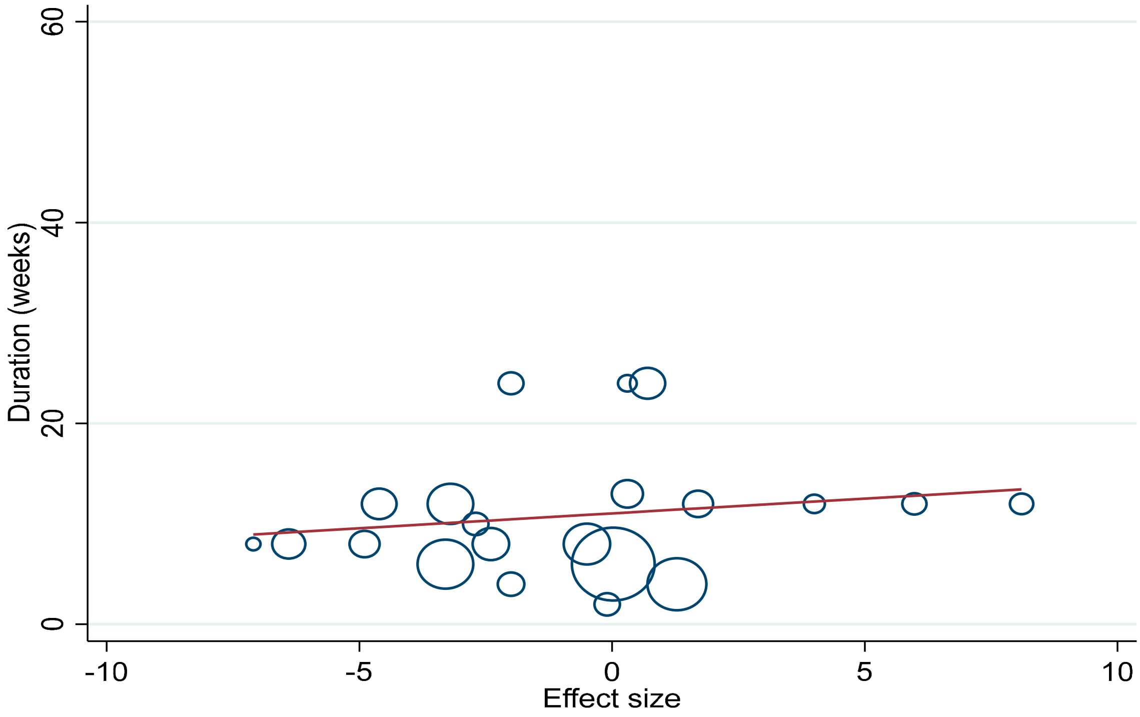

P) MDA

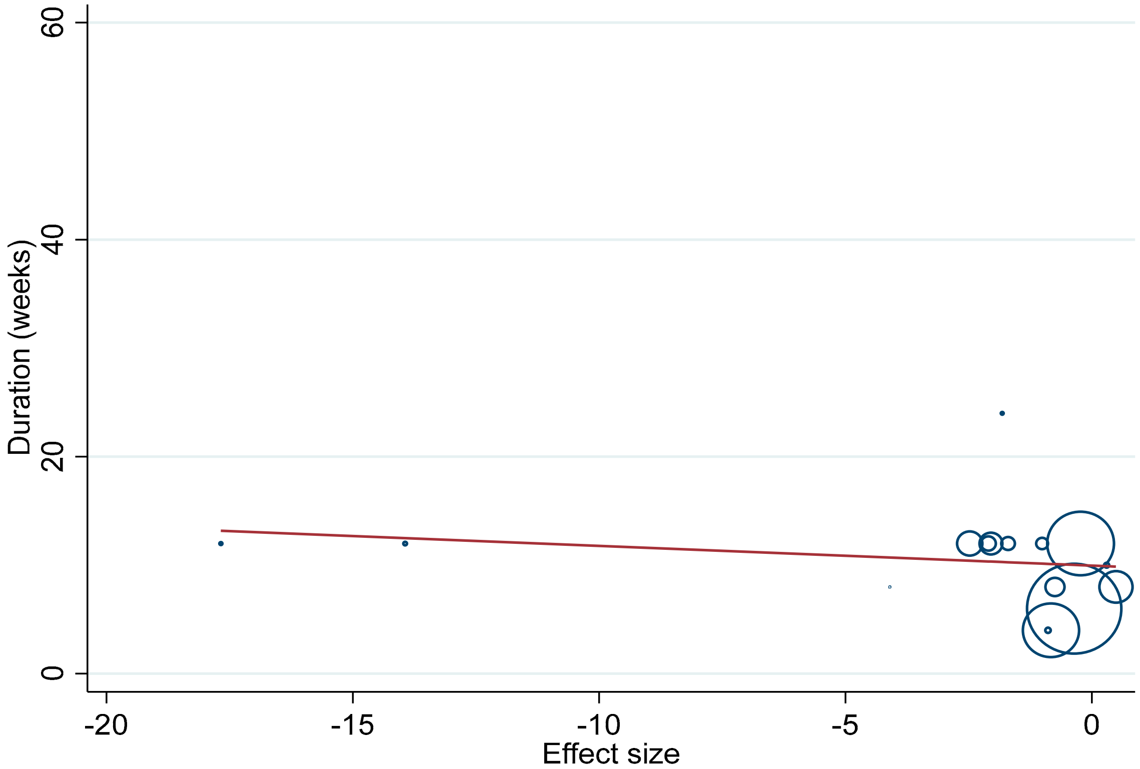

Figure S5. *Cont.*

Q) TAC

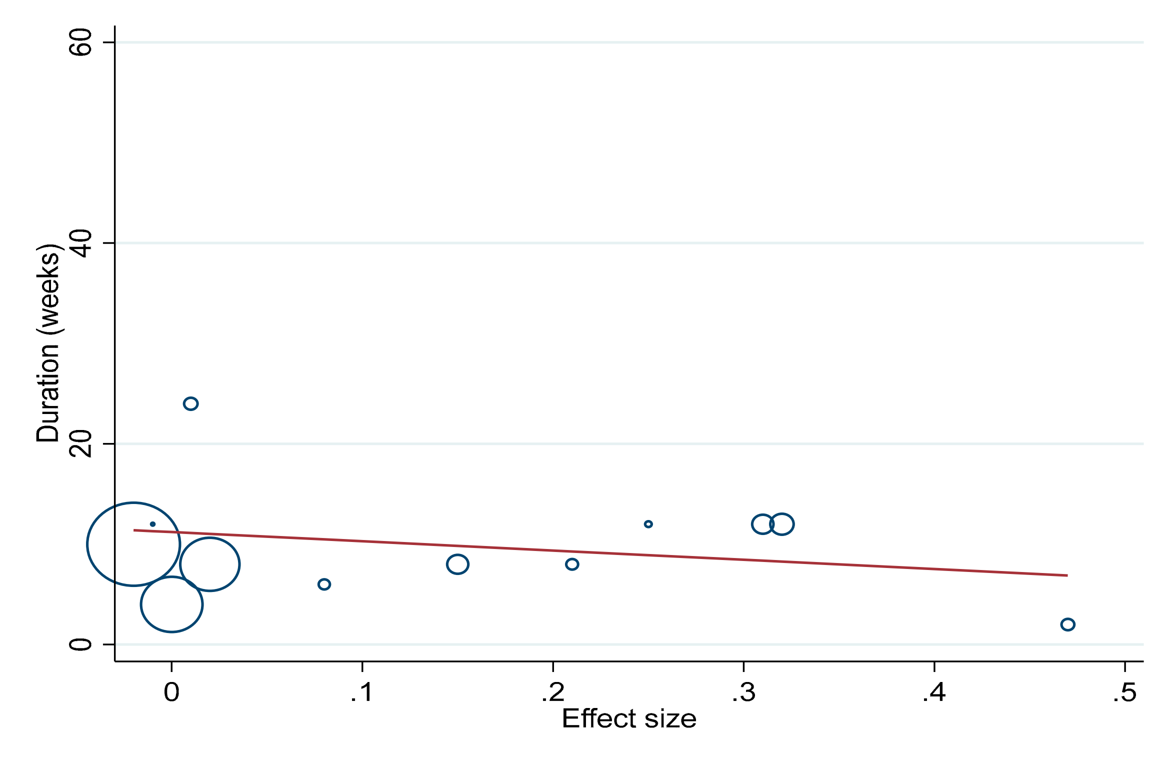

R) CRP

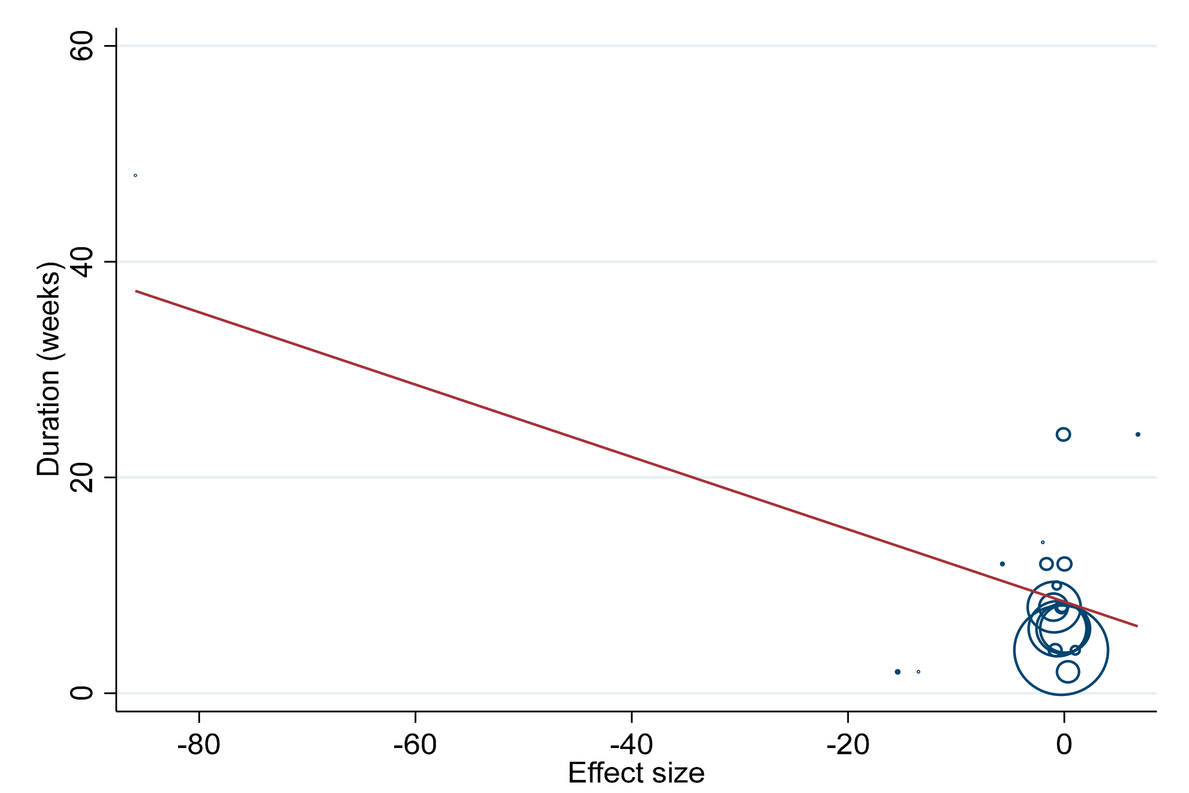

Figure S5. *Cont.*

S) IL-6

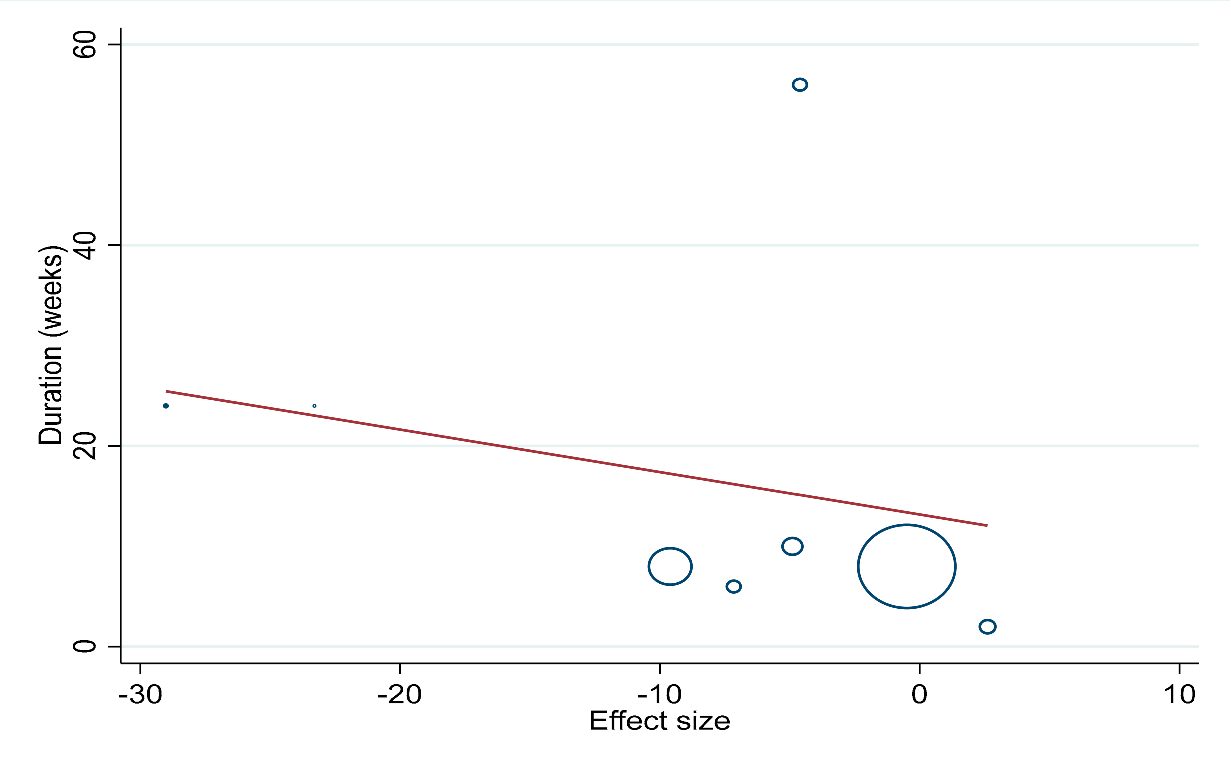

T) TNF-*α*

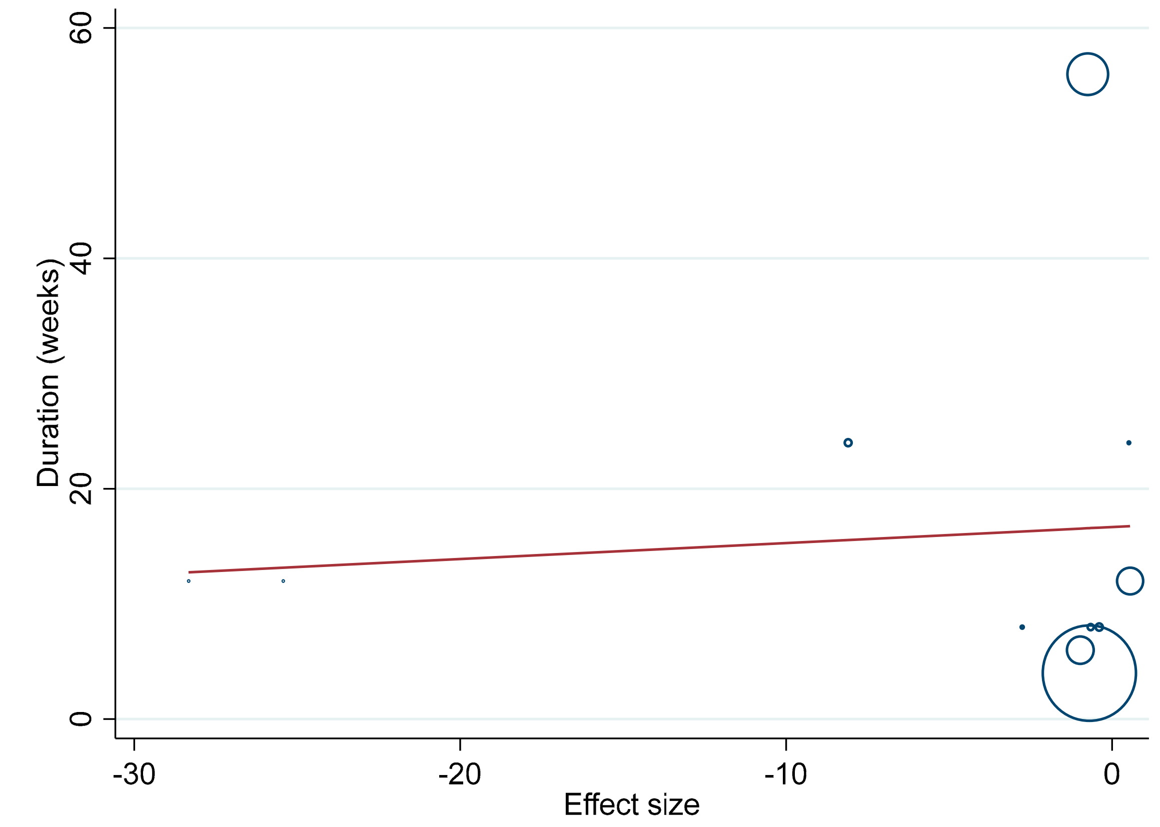

Figure S5. *Cont.*

U) AST

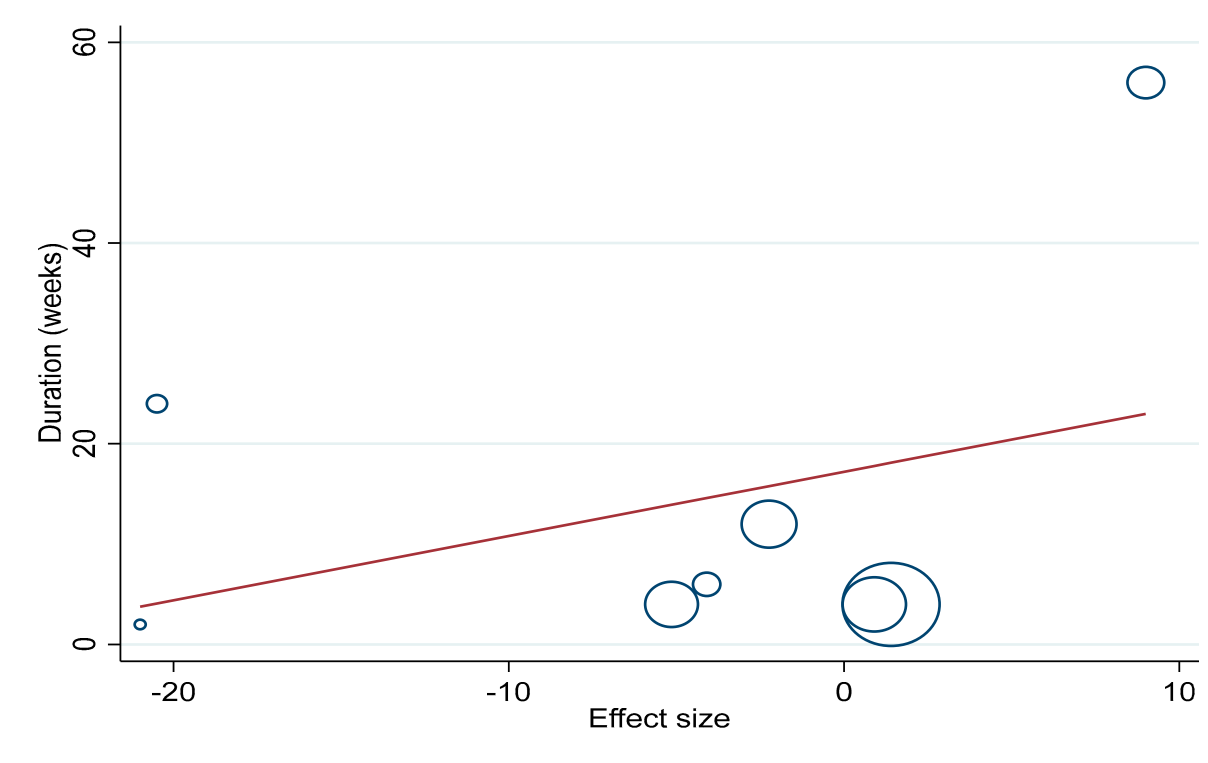

V) ALT

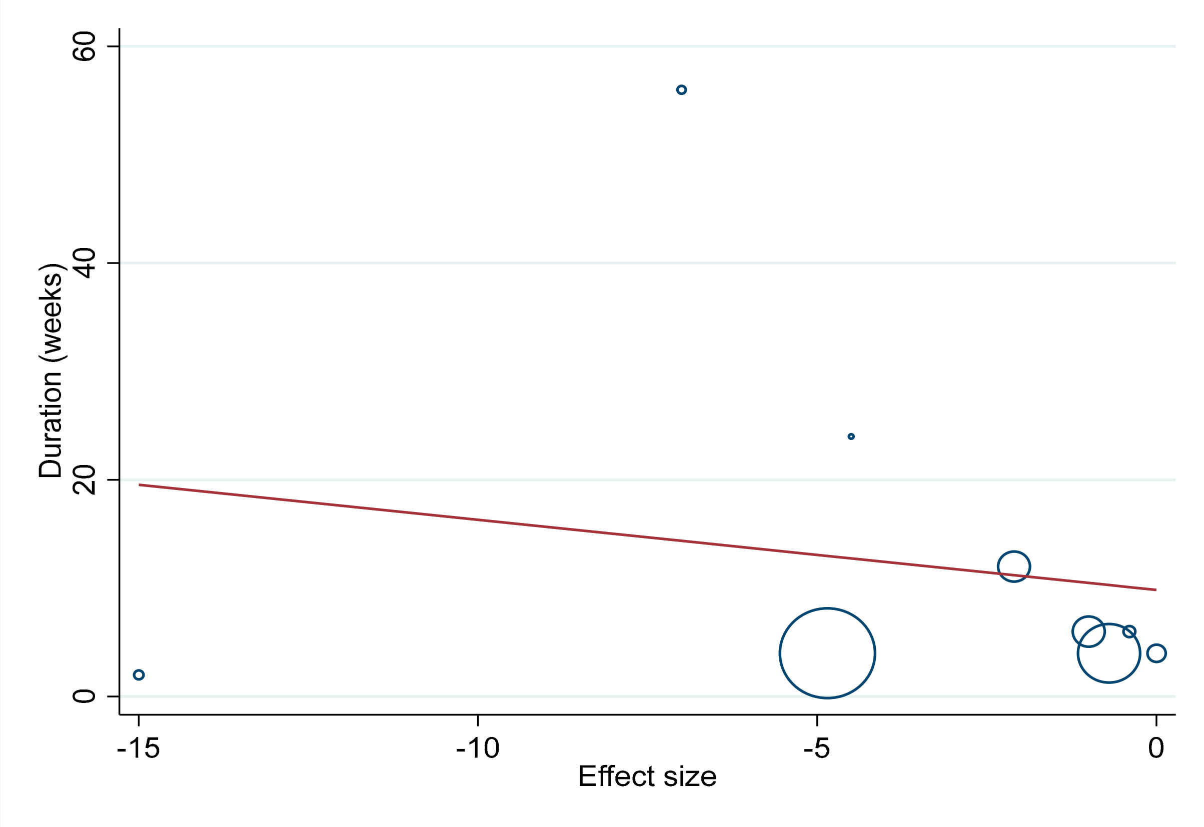

Figure S5. *Cont.*

W) GGT

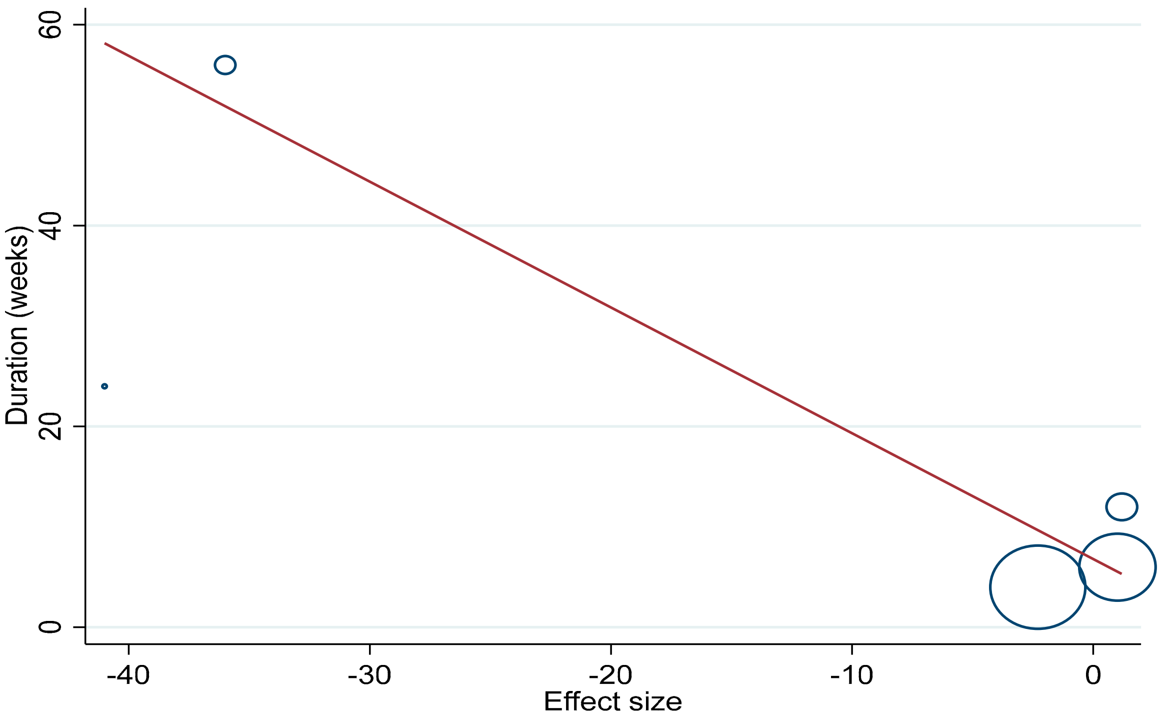

Figure S5. Linear association between duration (weeks) of melatonin supplementation and mean changes in cardiometabolic risk factors (CMRFs), including **(A)** BW (kg), **(B)** BMI (kg/m^2), **(C)** WC (cm), **(D)** HC (cm), **(E)** BFP (%), **(F)** FBG (mg/dL), **(G)** HbA1c (%), **(H)** FI (µIU/mL), **(I)** HOMA-IR, **(J)** TG (mg/dL), **(K)** TC (mg/dL), **(L)** LDL-C (mg/dL), **(M)** HDL-C (mg/dL), **(N)** SBP (mmHg), **(O)** DBP (mmHg), **(P)** MDA (µmol/L), **(Q)** TAC (mmol/L), **(R)** CRP (mg/L), **(S)** IL-6 (pg/mL), **(T)** TNF-α (pg/mL), **(U)** AST (IU/L), **(V)** ALT (IU/L), and **(W)** GGT (IU/L).
